# Effects of a high protein diet and liver disease in an in silico model of human ammonia metabolism

**DOI:** 10.1186/s12976-019-0109-1

**Published:** 2019-07-31

**Authors:** Jeddidiah W. D. Griffin, Patrick C. Bradshaw

**Affiliations:** 1 Department of Natural Sciences, Mars Hill University, Mars Hill, NC USA; 2 Department of Biomedical Sciences, Quillen College of Medicine, East Tennessee State University, Johnson City, TN USA

**Keywords:** Ammonia, Hepatic encephalopathy, Liver cirrhosis, Carbamoyl phosphate synthetase 1, Nitrogen, Urea cycle, Dietary protein

## Abstract

**Background:**

After proteolysis, the majority of released amino acids from dietary protein are transported to the liver for gluconeogenesis or to peripheral tissues where they are used for protein synthesis and eventually catabolized, producing ammonia as a byproduct. High ammonia levels in the brain are a major contributor to the decreased neural function that occurs in several pathological conditions such as hepatic encephalopathy when liver urea cycle function is compromised. Therefore, it is important to gain a deeper understanding of human ammonia metabolism. The objective of this study was to predict changes in blood ammonia levels resulting from alterations in dietary protein intake, from liver disease, or from partial loss of urea cycle function.

**Methods:**

A simple mathematical model was created using MATLAB SimBiology and data from published studies. Simulations were performed and results analyzed to determine steady state changes in ammonia levels resulting from varying dietary protein intake and varying liver enzyme activity levels to simulate liver disease. As a toxicity reference, viability was measured in SH-SY5Y neuroblastoma cells following differentiation and ammonium chloride treatment.

**Results:**

Results from control simulations yielded steady state blood ammonia levels within normal physiological limits. Increasing dietary protein intake by 72% resulted in a 59% increase in blood ammonia levels. Simulations of liver cirrhosis increased blood ammonia levels by 41 to 130% depending upon the level of dietary protein intake. Simulations of heterozygous individuals carrying a loss of function allele of the urea cycle carbamoyl phosphate synthetase I (*CPS1*) gene resulted in more than a tripling of blood ammonia levels (from roughly 18 to 60 μM depending on dietary protein intake). The viability of differentiated SH-SY5Y cells was decreased by 14% by the addition of a slightly higher amount of ammonium chloride (90 μM).

**Conclusions:**

Data from the model suggest decreasing protein consumption may be one simple strategy to decrease blood ammonia levels and minimize the risk of developing hepatic encephalopathy for many liver disease patients. In addition, the model suggests subjects who are known carriers of disease-causing *CPS1* alleles may benefit from monitoring blood ammonia levels and limiting the level of protein intake if ammonia levels are high.

**Electronic supplementary material:**

The online version of this article (10.1186/s12976-019-0109-1) contains supplementary material, which is available to authorized users.

## Background

Protein is an abundant part of the human diet. It is recommended that humans consume 0.8 g of protein per kg body mass per day. For a male of average weight (88.7 kg) [1], this is equivalent to 71 g of protein per day. When amino acids are consumed at a faster rate than they are used for protein synthesis, they are metabolized as an energy source, typically accounting for roughly 15–20% of the energy supply. The liver breaks down nearly half of the amino acids in the human diet as substrates for gluconeogenesis [2]. Amino acid catabolism first relies upon the transfer of the amino group by aminotransferases to a ketoacid, often to alpha-ketoglutarate to form glutamate, and then by the deamination of glutamate by glutamate dehydrogenase which produces ammonia (NH_3_). Roughly 12.5% of nitrogen intake is excreted from the digestive tract [3]. Because ammonia is relatively toxic [4], systems such as the urea cycle are in place primarily in the liver to convert it into a less toxic form that can be readily removed from the circulation and excreted.

The liver is the main organ responsible for filtering ammonia and other nitrogen sources such as glutamine from the blood to synthesize urea, the major form of excreted nitrogen in mammals. Urea is a relatively nontoxic waste product that safely stores nitrogen until it can be removed from the body. However, when ammonia is not successfully removed from the blood due to impaired or overwhelmed removal mechanisms, the plasma ammonia concentration increases, which may cause deleterious effects such as neural impairment [5]. As part of the process of nitrogenous waste removal, nitrogen-rich blood enters through the hepatic portal vein and is eventually filtered through the acinus, the functional unit of the liver, before draining out of the central vein. The acinus is divided into three zones [6]. Zone 1 is the closest to the hepatic portal vein, and zone 3 is the closest to the central vein. The hepatocytes spanning these three zones do not all perform the same metabolic functions [7]; rather, different branches of nitrogen metabolism are localized to specific zones. Zones 1 and 2 contain the enzymes of the urea cycle [8] as well as glutaminase [9], an enzyme that removes nitrogen from glutamine to yield ammonia and glutamate. However, zone 2 has less glutaminase activity than zone 1. Zone 3 contains glutamine synthetase [10], an enzyme that combines ammonia and glutamate to produce glutamine and is also called glutamate-ammonia ligase (GLUL).

Liver disease can change the activities of several key enzymes involved in nitrogen metabolism. For example, liver cirrhosis results in decreased expression of GLUL and the urea cycle enzyme CPS1 [11, 12]. Mutations in the *CPS1* gene can lead to individuals born with a deficiency in mitochondrial carbamoyl phosphate synthetase activity [13]. Because CPS1 catalyzes the first committed step of the urea cycle, this can have serious consequences on nitrogen metabolism. The increase in the number of individuals with liver disease in recent years [14] combined with an average protein intake in the U.S. that is about 40% above the recommended value [15] creates the need for understanding the effects of increased protein intake on blood ammonia levels.

Increased blood ammonia levels are a causative agent in hepatic encephalopathy (HE) [16], but increased ammonia levels have also been implicated in other neural disorders such as Alzheimer’s disease [17], amyotrophic lateral sclerosis [18], and Huntington’s disease [19]. HE results from liver damage leading to cognitive impairment. Liver disease also increases the blood levels of other potentially neurotoxic factors such as manganese and pro-inflammatory cytokines [20] that may contribute to the encephalopathy as well. Between 30 and 45% [21] of the more than 600,000 patients [22] with liver cirrhosis each year will develop hepatic encephalopathy, resulting in a cost of nearly $1 billion per year [21]. Most treatments aim to reduce the level of circulating ammonia [16]. Due to challenges in reliably assaying ammonia due to its reactivity [23, 24], there are not many studies that measured the effects of dietary alterations on blood or tissue ammonia levels. Our model provides further insight into how changes in dietary protein intake may affect blood ammonia levels to better direct these treatment strategies.

We used data from the literature to create a computational model that simulates ammonia metabolism and predicts blood ammonia levels based upon the amount of protein consumed and the degree of liver function. Results from the model agree relatively well with measured physiological and pathophysiological steady state metabolite levels, and several insights were made from varying our initial conditions to investigate the role of key enzymes in human organismal nitrogen metabolism. Cell culture studies were used to extend the model and establish the toxicity of pathophysiological concentrations of ammonia on differentiated SH-SY5Y neuroblastoma cells in culture.

## Methods

### Description of the model

This model describes the changes in ammonia, urea, and glutamine in the blood with the following ordinary differential equations:

1 $$ \frac{d\left[{NH}_3\right]}{dt}={V}_{NH3\  abs}+{V}_{GLS}-{V}_{Urea\ for\ Balance}-{V}_{CPS1}-{V}_{NH3\  ex}-{V}_{GLUL} $$

2 $$ \frac{d\left[ Urea\right]}{dt}={V}_{CPS1}-{V}_{Urea\  ex} $$

3 $$ \frac{d\left[ Gln\right]}{dt}={V}_{GLUL}-{V}_{GLS} $$

**Fig. 1:**
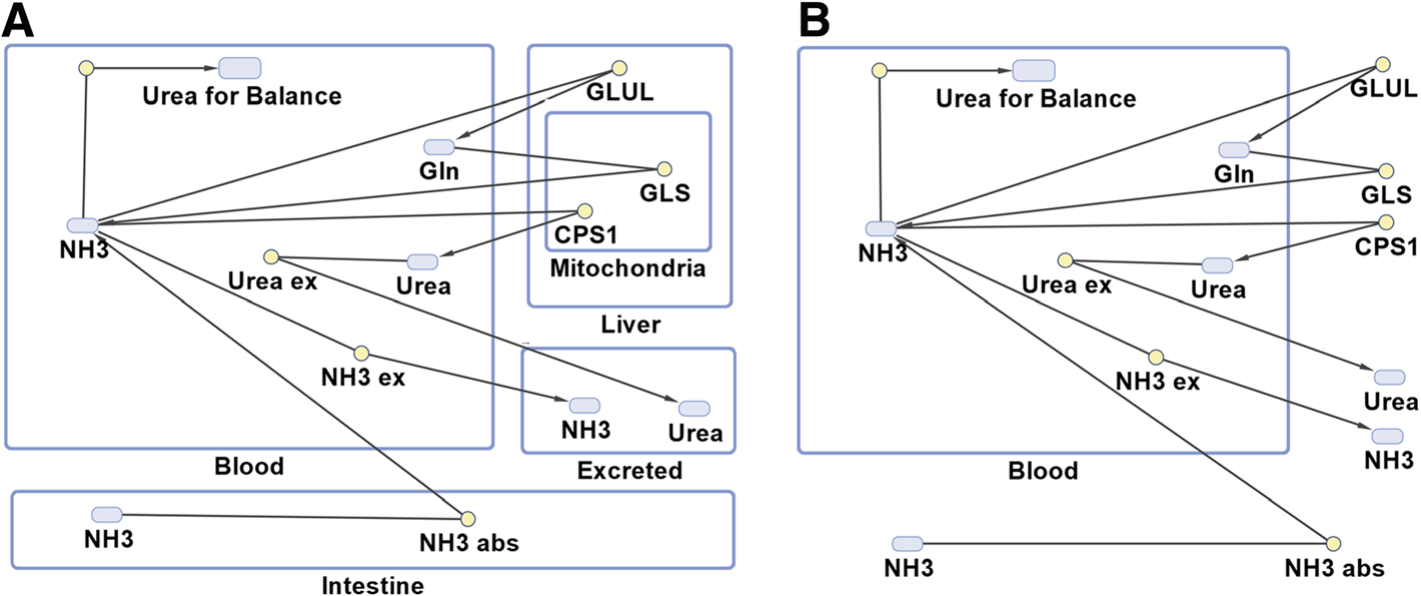
Model of nitrogen metabolism and excretion. NH3 in the figure includes both ammonia and ammonium ions. Circles represent reactions (see Table 1 for reaction equations) and ovals represent reactants and products. **a** Conceptual framework of the in vivo physiology simulated including the many tissues and subcellular compartments involved. **b** The system is modeled in silico considering only the concentrations of metabolites in the blood. Abbreviations are as follows: GLUL, glutamine synthetase; Gln, glutamine; GLS, glutaminase; CPS1, carbamoyl phosphate synthetase 1; Urea ex, urea excreted; NH3 abs, ammonia absorbed; NH3 ex, ammonia excreted

The overall reaction scheme is shown in Fig. 1. Equations for reaction velocities in Eqs. 1, 2, and 3 are shown in Table 1 [25–31]. Carbamoyl phosphate synthetase 1 (CPS1) catalyzes the first committed step in the urea cycle, so this is the only enzyme of the cycle incorporated into the model for simplicity. Other model parameters are shown in Table 2 [32–35]. Mammalian enzymes from liver tissue were used when the data was available (see Table 1). No distinction is made between NH_3_ and NH_4_^+^ in this study unless otherwise noted. N-acetyl-glutamate (NAG) is a CPS1 activator that increases in concentration when more protein is consumed [36]. To model the effects of NAG on CPS1 activity, we interpolated data on CPS1 activity changes from a study that included the effects of changes in hepatic mitochondrial NAG levels due to diet [36] (about 11% in this study). The maximally activated activity of CPS1 [27] was adjusted to reflect the reduced activity at physiological NAG concentrations [37, 38] for the different protein content in the diets and liver conditions used in the study. The adjusted CPS1 values for individuals on the three diets of differing protein content are as follows: 71 g protein per day, 8.05 mmoles/min; 100 g protein per day, 8.47 mmoles/min; 122 g protein per day, 8.78 mmoles/min. Adjustments for liver conditions are described below.

**Table 1:** Values for reaction velocities scaled to average male liver mass with recommended protein diet

Reaction Velocities	Parameters from the Literature	Organism and Tissue	Parameters Scaled to Liver
V_NH3 abs_ = 0.492 mmol/min^a^			
V_GLS_ = (V_max GLS_[Gln])/(K_m Gln_ + [Gln])	V_max GLS_ = 91.4 nmole/min per mg [25]	Rat Liver	V_max GLS_ = 28.54 mmol/min
	K_m Gln_ = 4.0 mM [26]	Human Recombinant	K_m Gln_ = 4.0 mM
V_NH3 ex_ = 0.004 mmol/min^a^			
V_CPS1_ = (V_max CPS1_[NH_3_])/(K_m NH3_ + [NH_3_])	V_max CPS1_ = 45 nmole/min per mg [27]	Human Liver	V_max CPS1_ = 8.05 mmol/min^ab^
	K_m NH3_ = 0.35 mM [28]	Human Recombinant	K_m NH3_ = 0.35 mM
V_GLUL_ = (V_max GLUL_[NH_3_])/(K_m NH3_{1 + ([Gln]/K_i Gln_)} + [NH_3_])	V_max GLUL_ = 0.47 μmole/15 min per mg [29]	Rat Liver	V_max GLUL_ = 12.3 mmol/min^b^
	K_m NH3_ = 0.15 mM [30]	Human Recombinant	K_m NH3_ = 0.15 mM
	K_i Gln_ = 0.6 mM [31]	*Bacillis subtilis*	K_i Gln_ = 0.6 mM
V_Urea ex_ = 0.244 mmol/min^a^			

^a^These values change with protein diet. See methods and discussion for details

^b^These values change with liver condition. See methods and discussion for details

**Table 2:** Parameter values used in nitrogen metabolism model

Parameter Name	Value	References
Blood Volume	6.59 L^a^	
Average Liver Mass	1561 g	[32]
Average Human Mass, Male	88.7 kg	[1]
Time Through Sinusoid	4.3 s	[33]
Initial Urea	5.5 mM	
Initial Ammonia	0 μM	
Initial Glutamine	0 mM	
Recommended Daily Protein	71 g	[34]

^a^Blood volume is about 5.2 L for a 70 kg individual [35]. The value used above was determined by assuming a linear relationship of blood volume with body mass for an individual weighing 88.7 kg. This is the volume of the blood compartment used to calculate metabolite concentrations

Because all the chemical species under consideration are present in the blood compartment, it is the only compartment where the volume affects simulation results. The other compartments in Fig. 1 are used to organize the model components for conceptualization. We assume free diffusion across membranes. The volume was determined by assuming a linear relationship between body mass and blood volume and taking 5.2 L to be the blood volume of a 70 kg male [35]. Blood in the model is assumed to be well mixed. Published enzyme activities were scaled up to the average liver size (1561 g, see Table 2) by adjusting the units to mmol/(min*1561 g). For example, McGivan et al. [25] report glutaminase activity as 91.4 nmoles per minute per mg protein, which converts to 0.0914 mmoles per minute per g protein. If we assume 20% protein content in the cultured hepatocytes, it takes 5 g of tissue to yield 1 g protein. Adjusting for protein content and multiplying by a liver weight of 1561 g yields an enzyme activity of 28.54 mmoles/min/liver. Because of the slightly higher protein content of liver tissue compared to isolated hepatocytes [39], we used a tissue protein content value of 25% when scaling up parameters from a study that reports enzyme activity from liver tissue. Similar calculations were performed for each of the liver enzymes. Changes in enzyme activity due to diet and liver conditions are summarized in Table 3.

**Table 3:** Parameter changes with altered protein intake and liver condition

Parameter Name	Protein in Diet (g per day)	Normal Enzyme Activity (mmoles/min)	Enzyme Activity in Liver Cirrhosis (mmoles/min)
Urea ex	71	0.244	0.244
	100	0.343	0.343
	122	0.417	0.417
NH3 ex	71	0.004	0.004
	100	0.008	0.008
	122	0.012	0.012
NH3 abs	71	0.492	0.492
	100	0.693	0.693
	122	0.845	0.845
V_max CPS1_	71	8.05	5.64
	100	8.47	5.93
	122	8.78	6.146
V_max GLUL_	No change with diet	12.3	2.46

The complete urea cycle uses two nitrogen atoms to synthesize each molecule of urea in one turn of the cycle. However, CPS1 incorporates one nitrogen atom per turn of the cycle. A second nitrogen enters into the cycle from aspartate when argininosuccinate synthetase catalyzes the reaction of aspartate with citrulline. To balance the stoichiometry of the reaction series and to simplify the model, a second reaction equal to CPS1 was created with the product not considered in the simulation results (“Urea for Balance” in Fig. 1). Therefore, two nitrogen atoms per unit time are used to produce one urea molecule, satisfying the stoichiometry of the overall reaction series for this simplified model. The rate of nitrogen absorption is based upon the daily amount of nitrogen consumed. The mass of protein ingested is adjusted to the molar amount of nitrogen ingested (16% of the mass of protein ingested) and for the 12.7% loss of nitrogen in feces [3]. The remaining molar amount of nitrogen is assumed to be absorbed linearly over a period of 24 h. For example, if 71 g of protein is ingested, 16% of that amount (11.36 g) is nitrogen. Assuming a 12.7% loss in feces, this leaves 9.92 g of nitrogen available for absorption. Because there are 14 g of nitrogen per mole, there are 0.708 mol of nitrogen available for absorption per day, or 0.49 millimoles of nitrogen per minute. The absorption rate was recalculated using the same method for each of the three protein diets modeled. The ranges of the amounts of ammonia and urea excreted in urine over a 24 h period have been reported [40, 41], and in the model we assumed the average values to be excreted linearly over the 24 h period. When modeling altered protein intake, the rates of ammonia and urea excretion were adjusted as well. For the high protein diet, we used the upper values of the reference ranges reported for the amounts of ammonia and urea excreted instead of the average values. This was accomplished by assuming daily nitrogen balance and using calculations similar to those used above to equate molar amounts of nitrogen to grams of protein. For example, using the lower values for the reference ranges of daily ammonia and urea excretion and assuming daily nitrogen balance suggest a dietary intake of about 60 g of protein per day. Ammonia and urea excretion rates were scaled to the dietary protein intake by assuming a linear relationship between the two. Excretion rates that were used in the model are as follows: for the 71 g per day protein diet, 0.004 mmoles ammonia are excreted per minute and 0.244 mmoles urea are excreted per minute; for the 100 g per day protein diet, 0.008 mmoles ammonia are excreted per minute and 0.343 mmoles urea are excreted per minute; and for the 122 g per day protein diet, 0.012 mmoles ammonia are excreted per minute and 0.417 mmoles urea are excreted per minute. Changes with altered protein content in the diet are summarized in Table 3.

**Fig. 2:**
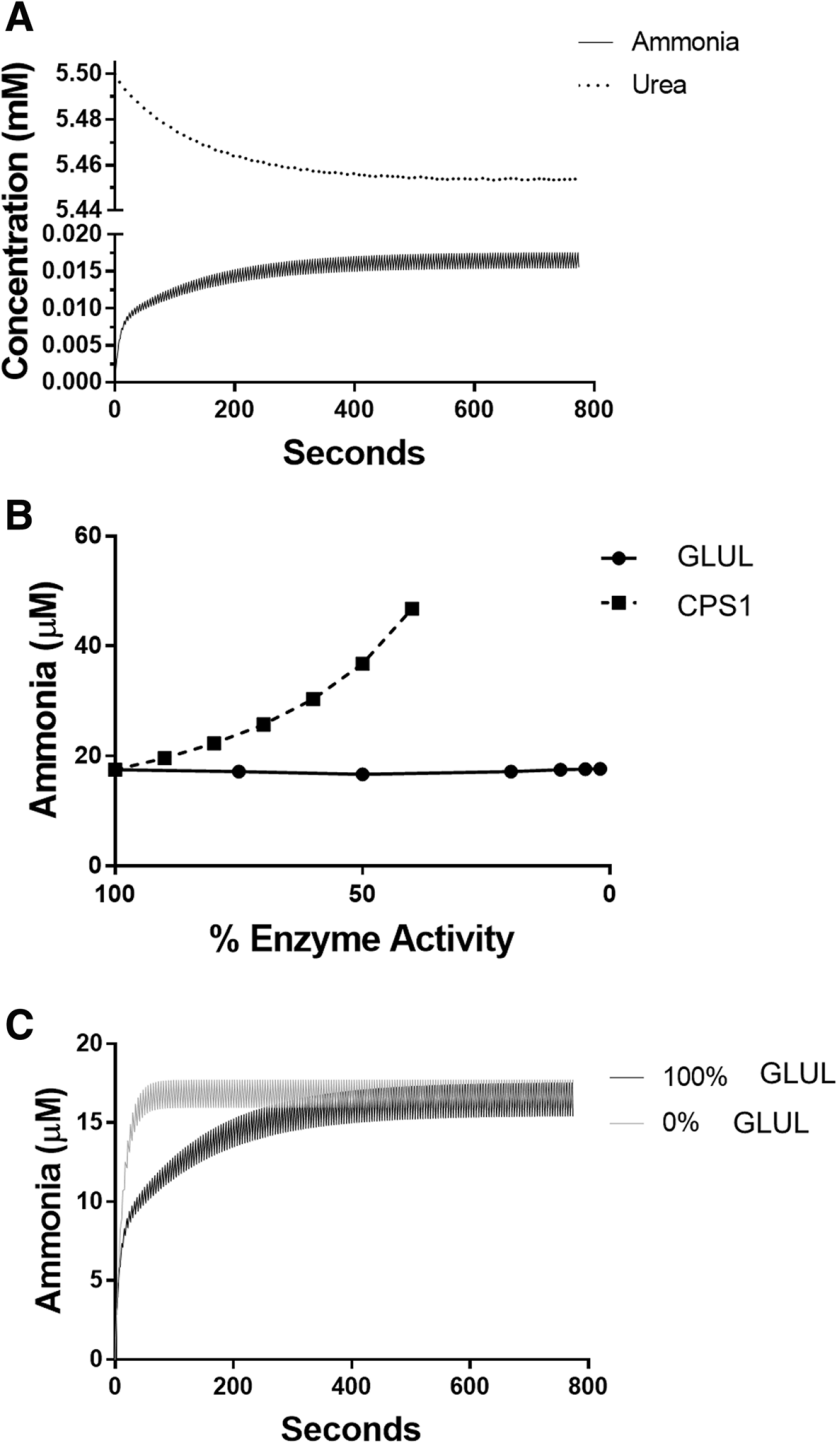
Model simulation results for an individual with normal enzyme activities on a recommended protein diet. **a** Simulation results for an individual with normal enzyme activities with the recommended daily protein intake. Note the break in the scale of the y-axis. **b** Ammonia steady state levels from simulations varying enzyme activities of CPS1 and GLUL. **c** Simulation results showing changes in ammonia kinetics with changes in GLUL activity

We modelled the spatial separation of enzymes by acinus zones by translating spatial separation into temporal separation. The time a red blood cell takes to travel through a sinusoid has been calculated to be 4.3 s [33]. Assuming a constant rate and equal division of zones, this is about 1.43 s per zone. CPS1 and GLS are found in two zones in the acinus (zones 1 and 2); this means a red blood cell would take about 2.87 s to cross these zones. By using event functions in the SimBiology software, the enzyme activities of CPS1 and glutaminase were turned on for 2.87 s and then off for 1.43 s while GLUL was turned on. This change in enzyme activities combined with the well-mixed assumption approximates the spatial enzyme separation found in the liver because blood is exposed to CPS1 and glutaminase for twice as long and just prior to exposure to GLUL activity before repeating the cycle. This cycling was repeated for 774 s during simulations (Fig. 2a), yielding a pattern of peaks and valleys in the simulation results that is a mathematical artifact of modeling spatial separation as temporal separation. The 774 s of simulation allowed sufficient time to reach the steady state definition (less than 0.002% change per second).

Steady state levels for ammonia were calculated following model simulations for different levels of protein in the diet (recommended, 71 g per day; average, 100 g per day; high, 122 g per day) and liver conditions (no disease, liver cirrhosis, and 50% CPS1 activity) for 9 conditions total. Simulation results were exported to Microsoft Excel, and steady state levels were determined from the data points corresponding to the top of the resulting ammonia concentration curves. The rate of change of the ammonia concentration during the final part of the simulation was calculated. Steady state was defined as the concentration when blood ammonia levels were changing less than 0.002% per second.

### Model assumptions and simplifications

Several assumptions and simplifications were used in the model. The urea cycle is simplified to the first committed step and an extra CPS1 reaction was added to maintain the stoichiometry of the entire urea cycle (“Urea for Balance” in Fig. 1). Other molecular species involved in the included reactions that are not included in the model are assumed to be in abundance, blood is assumed to be well-mixed, and we used temporal separation of enzymes to model spatial separation of enzymes in the liver acinus. Furthermore, GLS activity is the same in zones 1 and 2 in the model, but it may be slightly decreased in zone 2 in vivo. We recognize that blood is heterogeneous, and blood ammonia levels may be concentrated in some compartments compared to others. For example, arterial blood has been shown to have higher ammonia levels than venous blood in dogs with liver disease [42]. This difference of concentrations could limit systemic effects of blood ammonia. However, this was not included in the model. We also assume a continuous nutritional supply for simplicity. Furthermore, the model does not account for the activities of transporters but often assumes free passage of small molecules. There is also evidence for positive cooperativity for the binding of some of the species to enzymes in the model. For example, there is evidence of cooperativity for glutamine binding (Hill coefficient of 1.8) in the kinetics of the glutaminase enzyme [43] and possible glutaminase upregulation by a high protein diet [44] that was not included in the model. However, sensitivity analysis suggests that increases in glutaminase and GLUL activity will not have much effect on ammonia steady state levels. Even with these simplifying assumptions, the model results agree relatively well with the available clinical data (see Discussion). However, the available clinical data is sometimes incomplete, so further validation is not currently possible. The SBML file for this model can be found in Additional file 1.

### Sensitivity analysis

To determine the relative effects of altering enzyme kinetic parameters on the steady state blood ammonia levels, each of the parameters was individually increased or decreased by 50% under the normal simulation conditions for a liver without disease and a protein diet of 71 g per day. The simulations were run under these altered conditions and the steady state levels of ammonia were compared to those under normal conditions. Results are reported in Table 4 as percent change.

**Table 4:** Sensitivity analysis for kinetic parameters

Enzyme	Parameter	Parameter Percent Change	Blood Ammonia Percent Change
CPS1	V_max_	150%	−35.4
		50%	110.3
	K_m_	150%	52.6
		50%	−50.3
Glutaminase	V_max_	150%	0.6
		50%	−2.9
	K_m_	150%	−1.1
		50%	1.1
Glutamine Synthetase	V_max_	150%	4.6
		50%	−4.6
	K_m_	150%	−2.9
		50%	7.4
	K_i_	150%	0.6
		50%	−0.6

### SH-SY5Y culture, differentiation, and treatment

SH-SY5Y cells were cultured in a 1:1 mixture of DMEM (high glucose) and Ham’s F-12 medium. The medium contained 2.44 g/L sodium bicarbonate, 30 mg/L penicillin, 50 mg/L streptomycin, and 10% FBS. The cells were seeded at a concentration of 1,000 cells per well in 96-well plates and treated with retinoic acid (10 μM) for 4 days with the medium changed every 2 days. Next, ammonium chloride was added to the medium at the indicated concentrations and the cells were incubated for 24 additional hours.

### Protein assay

After 24 h of treatment with ammonium chloride, the cells were washed in PBS and then lysed with RIPA buffer (50 μl per well for three wells per condition). The lysate was pooled in a microcentrifuge tube and the Pierce BCA Protein Assay was performed in triplicate per manufacturer’s instructions.

### Software and statistical methods

The model was built and simulated using the MATLAB R2016a SimBiology software package. Data was analyzed using Microsoft Excel and GraphPad Prism v7.0. For cell culture studies, three independent experiments were performed and data was analyzed with a repeated measures one-way ANOVA with Fisher’s post hoc test.

## Results

### Model results approximated physiological steady state ammonia levels

A computational model estimating blood ammonia levels in individuals without disease and those with liver disease was constructed. The model was first simulated using the parameters for a liver without disease and the recommended protein content in the diet to determine if the resulting steady state levels were consistent with clinical data. The recommended protein intake was taken to be 71 g per day. The reference range for normal blood ammonia levels was taken to be 11–32 μM [45]. Normal blood urea levels are considered to be 3.6–7.1 mM [46]. The initial conditions in this study are 0 μM ammonia and 5.5 mM urea. Fig. 2a shows the results of 774 s of simulation of our model. The steady state ammonia level was 17.5 μM (Fig. 2a), and the steady state urea level remained around 5.5 mM (Fig. 2a), both values well within the normal range.

### Enzyme activity changes had different effects on ammonia and urea levels

To investigate the relative influences of CPS1 and GLUL on blood ammonia levels, enzyme activities were individually varied stepwise in the model for an individual without disease with a recommended protein intake, and steady state levels of ammonia were determined. The results show an inverse, non-linear relationship between CPS1 activity and ammonia levels (Fig. 2b). The steady state urea level did not show much change under any conditions tested likely because much larger changes are necessary to cause differences in the millimolar concentrations of urea compared to the micromolar concentrations of ammonia. Decreasing GLUL activity had almost no effect on steady state levels of ammonia (Fig. 2b). However, decreasing GLUL activity does affect the kinetics of ammonia formation. Eliminating GLUL activity caused the simulation to reach steady state ammonia levels much more quickly. In an individual without liver disease, the rate of ammonia formation for the first 12.9 s of the simulation was 0.55 μmoles per second, but this rate increased to 0.83 μmoles per second when GLUL activity was inactivated (Fig. 2c). A sensitivity analysis (Table 4) of the kinetic parameters revealed that, as expected, changes in CPS1 activity have by far the strongest effect on blood ammonia levels of any enzyme in the model.

### Changes in enzyme activity levels caused by liver cirrhosis affected blood ammonia levels

**Fig. 3:**
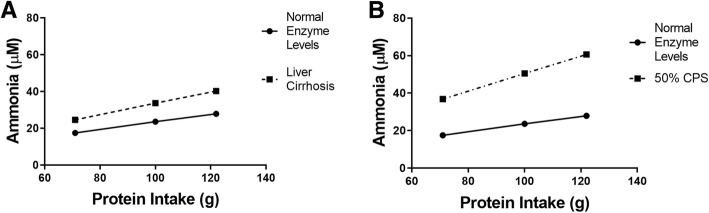
Ammonia levels changed with dietary protein levels, liver cirrhosis, and CPS1 activity. **a** The steady state ammonia concentration in the blood increases with increased ammonia absorption due to increases in dietary protein (See Table 1) and decreased liver function. **b** When CPS1 activity is reduced by half, steady state ammonia levels are higher than those in an individual with normal enzyme activities. This effect increases with increased ammonia absorption due to increased dietary protein levels (See Table 1)

Liver cirrhosis has been shown to decrease the activities of two of the enzymes in the model, so decreasing the V_max_ values accordingly can create a simple model of liver cirrhosis. As mentioned above, the recommended protein intake for a typical adult male is 71 g of protein per day. The average American diet is about 100 g of protein per day, and a high protein diet in our model was taken to be 122 g of protein per day. The amount of protein in the high protein diet was calculated by using the highest values of ammonia and urea excretion in the published reference ranges [40, 41] and assuming nitrogen balance (see Methods). To model liver cirrhosis, the V_max_ of CPS1 was reduced to 70% of the normal value, and the V_max_ of GLUL was reduced to 20% of the normal value [11]. Following simulation, the steady state ammonia and urea levels were determined as described above. The steady state ammonia levels under the various conditions are shown in Fig. 3a. Ammonia levels increased with increased dietary protein intake and with decreased liver function. For simulations of a liver without disease, increasing protein consumption from the recommended protein intake to the high protein diet increased ammonia levels by roughly 59%. Simulations of cirrhosis led to increases of blood ammonia levels of 41 to 130% depending upon the level of protein intake.

### Decreased CPS1 activity led to increased blood ammonia levels

Based upon the population frequency of the genetic disorder CPS1 deficiency, there are likely many heterozygous individuals with decreased CPS1 activity (see Discussion). To investigate the consequences of decreased CPS1 activity on blood ammonia and urea levels, the V_max_ for CPS1 was reduced by 50% and different protein levels in the diet were compared (Fig. 3b). Decreased CPS1 activity (50% below normal, as present in a heterozygous individual with a complete loss of function from one of the two alleles) led to more than a doubling in blood ammonia levels for the recommended protein intake and more than a tripling of blood ammonia levels for the high protein diet.

### Ammonium chloride treatment decreased viability of differentiated SH-SY5Y cells

**Fig. 4:**
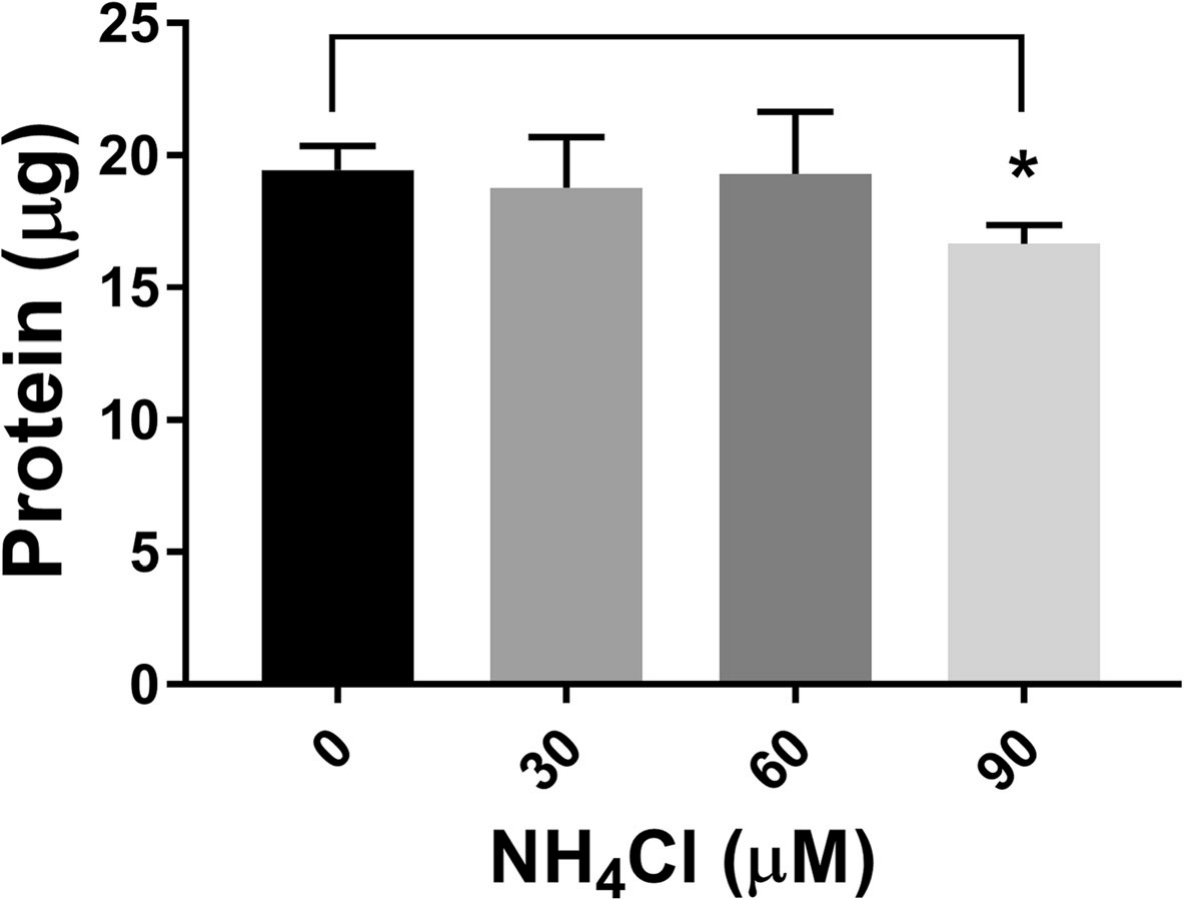
Ammonium chloride (90 μM) decreased the viability of retinoic acid-differentiated SH-SY5Y neuroblastoma cells in culture. * indicates that *p* = 0.01 compared to 0 μM NH_4_Cl added. Bars represent mean ± SEM, and *n* = 3 independent experiments

To test whether the increased ammonia levels observed in the simulations could be neurotoxic in vitro*,* we administered ammonia to retinoic acid-differentiated human neuroblastoma cells. Differentiated SH-SY5Y cells treated with 90 μM ammonium chloride showed 14% decreased viability as measured by protein content from cells attached to the plate after a PBS wash. Ammonium chloride concentrations of 30 μM or 60 μM showed no statistically significant effect on viability (Fig. 4).

## Discussion

A simple mathematical model of human organismal nitrogen metabolism is presented that uses published parameters for physiological inputs to give physiologically relevant outputs consistent with the available experimental data. While other models for ammonia metabolism in humans exist [47–49], this is the first to our knowledge to model the effects of altered levels of dietary protein intake on blood ammonia levels. Increased protein intake led to increased blood ammonia levels across all conditions. However, blood ammonia levels remained in the normal range when simulating a liver with normal enzyme activities. The simulation results showed that low GLUL activity can lead to more rapid changes in blood ammonia levels. The model highlights the effects of diet on ammonia levels in disease conditions. Our results indicate that increased protein intake likely causes blood ammonia to rise above normal levels in some patients with cirrhosis.

### Altering dietary protein consumption in cirrhosis patients

Because relative energy expenditure per kg body mass is increased in some cirrhotic patients [50], some sources recommend a high protein diet (1.8 g/kg per day) for these patients to maintain muscle mass if they do not already have HE [51]. Others suggest a normal, moderate intake of 0.8 g to 1.0 g of protein per kg per day [52]. Data has shown that high protein intake exacerbated encephalopathy in 35% of patients with cirrhosis [53]. Reduced protein intake was first shown to protect from encephalopathy in cirrhotic patients in 1952 [54]. In 2004, a study was performed with cirrhosis patients where protein was completely removed from the diet for 3 days and then slowly increased over 12 days back to the normal level [55]. At the end of the study, blood ammonia levels were non-significantly 17% lower in the patients with the restricted protein diet compared to patients on a normal protein diet of 1.2 g/kg per day, roughly equivalent to the average American protein diet in this model. However, the methods used in that study have been critiqued and questioned [56]. A larger patient group size and earlier measurements of blood ammonia levels would help to clarify if blood ammonia levels are indeed decreased by a low protein diet. If the findings of no significant effects do prove to be robust and highly reproducible, this leaves the possibility that a high protein diet may play a role in the development of HE, but a low protein diet is not helpful in its resolution. Even though several studies have suggested that restricting dietary protein intake below the recommended amount for an individual with normal enzyme activities may not be therapeutic for the roughly 60% of cirrhosis patients who suffer from malnutrition, other studies over the past 65 years on HE patients who can maintain a proper energy balance have consistently shown benefits of protein restriction [56]. We acknowledge the large heterogeneity in patient responses to changes in the level of dietary protein [57] and suggest that monitoring the cirrhosis patient’s energy balance will help determine the proper dietary protein level for that individual.

Previous research has shown that there was an 80% reduction in GLUL activity and a 30% reduction in CPS1 activity in a rat model of liver cirrhosis [11]. Results from adjusting the model to these parameters suggest that ammonia levels will increase with liver disease. Furthermore, a high protein diet will likely exacerbate these effects. We recognize that liver cirrhosis is a complex disease with many changes besides altered CPS1 and GLUL activity, so this model is a simplified representation of liver cirrhosis. Using an upper limit of 32 μM for the reference range of normal blood ammonia levels, the model indicates that a high protein diet with liver cirrhosis will result in a blood ammonia level that is at least 20% higher (40.3 μM) than the upper limit for the normal range (32 μM). This elevation may contribute to HE. The model suggests that controlling protein intake could be one method to slightly reduce the likelihood of developing HE in some patients with liver cirrhosis.

### Plasma ammonia levels likely rise after a meal

Our model predicts that blood ammonia levels will rise slightly by consumption of a high protein diet. Surprisingly, we could not find many studies in the literature examining dietary-induced changes in blood ammonia levels in healthy humans. One study found increased blood ammonia levels in women following consumption of a test drink containing whey protein. The ammonia level peaked at a value 20% higher than the initial level at 90 min after consumption [58]. Another study found increasing breath ammonia levels after a high protein challenge; ammonia levels plateaued roughly 5 h after the dietary challenge [59]. However, breath ammonia levels do not always correlate well with blood ammonia levels. A further study using Huntington’s disease patients who were put on a high (26.3%) protein diet did not find any association between the high protein diet and blood ammonia levels [19].

In studies with mice placed on a high protein diet, ammonia levels increased from 210 μM to 245 μM at night when the mice were active and feeding. Likewise, a high fat/low protein diet decreased ammonia levels from 170 μM to 130 μM when measured at night [60]. When rats were switched from a 20% protein diet to a 58% protein diet, colonic venous ammonia maximally increased from 100 μM to 340 μM 2 days after the switch in diet, which dropped to 170 μM after a week on the diet [61]. Another study using rats trained on a 6% protein diet showed that blood ammonia levels increased from 60 μM to 120 μM when they were given a 44% protein meal, and ammonia levels were maintained at that elevated level for at least 24 h [62]. There is also evidence of increases in blood ammonia levels in pigs after a protein meal [63, 64]. The limited data above suggest that systemic ammonia levels likely increase slightly following a meal, especially if the meal is high in protein, but more experiments should be performed to verify these initial findings and to determine the extent of brain ammonia level changes under the same conditions. In addition, more detailed studies using human subjects would help to better characterize the time dependency of elevated ammonia levels as well as to discern differential effects in patients with liver disease.

### Variability in the correlation between blood ammonia levels and the severity of HE

While there is a correlation between blood ammonia levels and HE severity [65] the exact blood concentration that leads to impairment may be different for individuals based on their specific nitrogen balance and the release of other neurotoxic factors from the liver. Some patients with hyperammonemia present with 100–200 μM blood ammonia levels and remain asymptomatic, while others such as infants with GLUL deficiency have severe encephalopathy with blood ammonia levels fluctuating between 100 and 150 μM [66]. However, decreased blood glutamine levels may also contribute to the encephalopathy in GLUL deficiency. Maintained blood ammonia levels over 300 μM are considered severe and invariably lead to encephalopathy. However, some newborns have been shown to have no lasting impairment by temporary blood ammonia concentrations up to 2 mM for a day or two [67]. In contrast with this data, blood ammonia levels (< 50 μM) that border the normal range have also been linked to HE [65]. These variabilities do not affect the validity of the model because the model represents the blood ammonia level changes for an average person.

To investigate the roles of CPS1 and GLUL in ammonia metabolism, enzyme activities were varied in the model with otherwise normal parameters (Fig. 2b, Table 4). CPS1 activity levels relevant to the model had an inverse, non-linear effect on ammonia concentrations. This is consistent with clinical data that demonstrate ammonia levels increase due to CPS1 deficiency [68]. GLUL activity, however, had very little effect on ammonia steady state levels. Simulating liver cirrhosis (70% CPS1, 20% GLUL) resulted in an average ammonia increase of 43% across all diets. GLUL activity appears to slow initial changes in blood ammonia levels. Since muscles may help metabolize some ammonia [69], slowing the rate of change may give the body time to adapt to the larger ammonia levels. These results are consistent with the known role of CPS1 together with the rest of the urea cycle to be a low affinity, high capacity system for removing ammonia, while GLUL is a high affinity, low capacity enzyme for removing ammonia [70].

### Ammonia levels in individuals deficient in GLUL

Human subjects with decreased GLUL activity have been shown to have blood ammonia levels of 100–150 μM [66], while mice with liver-specific GLUL knockout showed a blood ammonia level of roughly 150 μM [71]. Our model, under otherwise normal conditions, shows no change in ammonia steady state levels when GLUL is reduced (Fig. 2b). This is a major limitation of the study possibly due to the enzyme kinetics data available, but it could also be due to the way we have modeled the hepatic acinus, the functional unit of the liver. The ratio of CPS1 to GLUL activity or the amount of time that ammonia is associated with CPS1 activity compared to GLUL activity may be too high in our model, resulting in deviations from in vivo results. This suggests that some of the changes in ammonia levels observed in our model may be too conservative. However, CPS1 deficiency [72] has been demonstrated to lead to higher plasma ammonia levels than GLUL deficiency [73], indicating that GLUL does not exert as strong an effect on blood ammonia levels as CPS1.

### Ammonia levels in individuals deficient in CPS1

CPS1 deficiency is a rare autosomal recessive genetic disorder that results in very little CPS1 activity [74]. Individuals experience extreme hyperammonemia and the many detrimental effects that come with it [13]. The prevalence of CPS1 deficiency is about 1 in 800,000 [75]. Assuming strict Mendelian inheritance, if a mother and father each are heterozygous for a complete loss of function allele and have decreased CPS1 activity, there is a 1 in 4 chance that their children will have CPS1 deficiency. Working backwards from this assumption, the odds that both parents are heterozygous are 1 in 200,000. The odds that one parent is heterozygous is roughly 1 in 447. Therefore, heterozygosity for disease-causing CPS1 mutations is almost as prevalent as liver cirrhosis. However, regulatory effects may partially compensate for CPS1 heterogeneity, a scenario not explored in this study. The model predicts that individuals with CPS1 activity 50% of normal levels (currently thought to have no detrimental effect) will have high blood ammonia levels. Most CPS1 disease allele carriers are likely unaware that they possess a mutation in one CPS1 allele that may result in higher than normal levels of blood ammonia. Lifelong exposure to high levels of blood ammonia may have unknown, deleterious effects on neural function.

### Relatively low (90 μM) ammonia levels may affect neural cell viability or function

It is hypothesized that the increased levels of ammonia in liver disease interfere with the glutamine-glutamate balance involved in neurotransmission [76], which can lead to increased production of reactive oxygen and nitrogen species [77]. Increased brain ammonia levels also block potassium uptake in astrocytes, which causes increased potassium uptake in neurons that compromises inhibitory neurotransmission in the cortex, leading to seizure [78].

Cell culture experiments using retinoic acid-differentiated SH-SY5Y cells revealed that viability was decreased by relatively low concentrations (90 μM) of ammonium chloride. The ability of such a low concentration of brain ammonia to cause toxicity was surprising given that most cells [79] including rodent primary cortical and cerebellar granule cells and undifferentiated human SH-SY5Y cells require low (1–10) millimolar concentrations of ammonium chloride before toxicity is observed [77]. Ammonia has been reported to be slightly more toxic to neuroblastoma cells than to primary neurons [80], partially accounting for the lower toxicity threshold. Furthermore, the retinoic acid-mediated differentiation procedure we used likely sensitized the cells to ammonia toxicity as it is known to increase reactive oxygen species production [81].

The ability of uncharged NH_3_ to cross the blood brain barrier and the limited ability of the charged, protonated form NH_4_^+^ to cross the barrier combined with the difference in pH between the brain and the blood allows higher total ammonia levels to accumulate in the brain [4]. The model predicts serum (pH ~ 7.4) levels of ammonia/ammonium in individuals with normal enzyme activities will be 17.5 μM; since the brain has a pH of about 7.0 [82, 83], applying the Henderson-Hasselbalch equation implies a total ammonia/ammonium concentration in the brain of 44 μM for a individual on a recommended protein diet, more than twice the blood ammonia/ammonium concentration. The following diet combinations and liver conditions are predicted from the model results to have brain ammonia/ammonium concentrations of > 90 μM: high protein diet/cirrhosis (101 μM), recommended protein diet/decreased CPS1 activity (92 μM), average protein diet/decreased CPS1 activity (127 μM), and high protein diet/decreased CPS1 activity (152 μM).

The cell culture experiments that we performed highlight the relevance of our study. Brain ammonia levels predicted by the model for the disease conditions decreased the viability of differentiated neuroblastoma cells in culture. This suggests that these ammonia levels have the potential to damage or kill neurons, contributing to HE. Taken together the experimental and computational results suggest that several of the diet and liver condition combinations could negatively affect neuronal function. Mouse studies have shown that high protein diets, more so than high fat or high carbohydrate diets, are associated with decreased lifespan [84]. These results should stimulate further research into the mechanisms involved. Adhering to the recommended protein intake may help cirrhosis patients and CPS1 heterozygotes avoid high blood and brain ammonia levels and any associated cognitive dysfunction.

## Conclusions

The following testable hypotheses have been generated using this model: 1) Increasing dietary protein consumption increases blood ammonia levels in individuals with normal CPS1 and GLUL enzyme activities. 2) A low protein diet is beneficial for liver cirrhosis patients who have a normal energy balance. 3) Heterozygosity for CPS1 complete loss of function mutations leads to elevated blood ammonia levels. 4) Chronic but low-level hyperammonemia has negative effects on neurons and astrocytes such as sensitizing them to further toxic insults. 5) Increased blood ammonia levels contribute to the decreased lifespan of mice on a high protein diet [84].

This model describes physiological and pathophysiological human nitrogen metabolism in blood and liver using published parameters. It suggests that protein content in the diet and liver cirrhosis contribute to blood ammonia levels, and results from cell culture experiments suggest that these blood ammonia levels could affect neural functioning. Since high blood ammonia levels are associated with diseases such as HE, the model can be used to predict the conditions in which HE may develop. Furthermore, the model predicts that a 50% reduction in CPS1 activity, an activity level present in thousands of individuals worldwide, could lead to high blood ammonia levels. Limiting protein intake may be one effective way for some of these individuals to decrease blood ammonia levels and possible associated pathologies.

## Additional file

Additional file 1: SBML code for the model for an individual with normal enzyme activities with a recommended protein diet. (PDF 1135 kb)

## Data Availability

The SBML code for the model for an individual with normal enzyme activities on a recommended protein diet is included as supplementary material [see Additional file 1].
