# Supplementary material for: Effects of a high protein diet and liver disease in an in silico model of human ammonia metabolism
Source: Theor Biol Med Model. 2019 Jul 31;16:11. doi: 10.1186/s12976-019-0109-1 (PMC6670211; doi:10.1186/s12976-019-0109-1)
Supplement: Supplementary file 1 — SBML code for the model for an individual with normal enzyme activities with a recommended protein diet. (PDF 1135 kb) [file 12976_2019_109_MOESM1_ESM.pdf]

Electronic Supplementary Material 1 for “Effects of high protein diet and liver disease in an *in silico* model of human ammonia metabolism,” a paper submitted by Jeddidiah W. D. Griffin and Patrick C. Bradshaw to *Theoretical Biology and Medical Modelling*. SBML code for the model for an individual with normal enzyme activities on the recommended protein diet.

Correspondence: Department of Natural Sciences, Mars Hill University, Mars Hill, NC, USA, griffinjw@etsu.edu


```
<?xml version="1.0" encoding="UTF-8"?>
<sbml xmlns="http://www.sbml.org/sbml/level2/version4" level="2"
version="4">
  <annotation>
    <SimBiology xmlns="http://www.mathworks.com">
      <Version Major="5" Minor="4" Point="0"/>
    </SimBiology>
  </annotation>
  <model id="mwd6fafefa_b605_445f_b804_2e02e393dd3e" name="untitled">
    <listOfUnitDefinitions>
      <unitDefinition id="MWBUILTINUNIT_liter" name="liter">
        <listOfUnits>
          <unit kind="metre" exponent="3"/>
          <unit kind="dimensionless" multiplier="0.001"/>
        </listOfUnits>
      </unitDefinition>
      <unitDefinition id="MWBUILTINPREFIX_milli_MWBUILTINUNIT_mole"
name="millimole">
        <listOfUnits>
          <unit kind="mole" exponent="1" multiplier="0.001"/>
        </listOfUnits>
      </unitDefinition>
      <unitDefinition id="MWDERIVEDUNIT_millimole__minute"
name="millimole/(minute)">
        <listOfUnits>
          <unit kind="mole" exponent="1"/>
          <unit kind="second" exponent="-1"/>
          <unit kind="dimensionless" multiplier="1.66666666666667e-
005"/>
        </listOfUnits>
      </unitDefinition>
      <unitDefinition id="MWDERIVEDUNIT_millimole__liter"
name="millimole/liter">
        <listOfUnits>
          <unit kind="metre" exponent="-3"/>
          <unit kind="mole" exponent="1"/>
          <unit kind="dimensionless" multiplier="1"/>
        </listOfUnits>
      </unitDefinition>
      <unitDefinition id="MWDERIVEDUNIT_millimole__minute"
name="millimole/minute">
```

```
        <listOfUnits>
          <unit kind="mole" exponent="1"/>
          <unit kind="second" exponent="-1"/>
          <unit kind="dimensionless" multiplier="1.66666666666667e-
005"/>
        </listOfUnits>
      </unitDefinition>
    </listOfUnitDefinitions>
    <listOfCompartments>
      <compartment id="mwfd39d73a_90ba_47dc_8fea_568ef27807f9"
name="Blood" spatialDimensions="3" size="6.589"
units="MWBUILTINUNIT_liter" constant="true"/>
      <compartment id="mw74937632_864a_40d4_90b3_b865326ef92c"
name="Liver" spatialDimensions="3" size="0.4948"
units="MWBUILTINUNIT_liter" constant="true"/>
      <compartment id="mwf128b3a7_6c40_40ea_8ba0_9f5355e0337b"
name="Excreted" spatialDimensions="3" size="1.4"
units="MWBUILTINUNIT_liter" constant="true"/>
      <compartment id="mw100b8e10_19a9_464f_84ca_67b25ed1ded9"
name="Intestine" spatialDimensions="3" size="1"
units="MWBUILTINUNIT_liter" constant="true"/>
      <compartment id="mw0da013c8_a085_460b_9bd8_800dd0cc31a7"
name="Mitochondria" spatialDimensions="3" size="0.2357"
units="MWBUILTINUNIT_liter"
outside="mw74937632_864a_40d4_90b3_b865326ef92c" constant="true"/>
    </listOfCompartments>
    <listOfSpecies>
      <species id="mwd508e9bd_ed74_425d_9880_17e95bd7a346" name="NH3"
compartment="mwfd39d73a_90ba_47dc_8fea_568ef27807f9"
initialConcentration="0"
substanceUnits="MWBUILTINPREFIX_milli_MWBUILTINUNIT_mole"
hasOnlySubstanceUnits="false" boundaryCondition="false"
constant="false">
        <annotation>
          <SimBiology xmlns="http://www.mathworks.com">
            <Unit Numerator="millimole" Denominator="liter"/>
          </SimBiology>
        </annotation>
      </species>
      <species id="mwa6f6ed3a_00c3_49a0_b7f2_32da70289968"
name="Urea_B" compartment="mwfd39d73a_90ba_47dc_8fea_568ef27807f9"
initialConcentration="5.5"
substanceUnits="MWBUILTINPREFIX_milli_MWBUILTINUNIT_mole"
hasOnlySubstanceUnits="false" boundaryCondition="false"
constant="false">
        <annotation>
          <SimBiology xmlns="http://www.mathworks.com">
            <Unit Numerator="millimole" Denominator="liter"/>
          </SimBiology>
        </annotation>
      </species>
```

```
      <species id="mw5ffd8486_9941_4b71_8698_d5d06edc44df"
name="pUrea" compartment="mwfd39d73a_90ba_47dc_8fea_568ef27807f9"
initialConcentration="0"
substanceUnits="MWBUILTINPREFIX_milli_MWBUILTINUNIT_mole"
hasOnlySubstanceUnits="false" boundaryCondition="false"
constant="false">
        <annotation>
          <SimBiology xmlns="http://www.mathworks.com">
            <Unit Numerator="millimole" Denominator="liter"/>
          </SimBiology>
        </annotation>
      </species>
      <species id="mwc8acca22_48dd_47bf_9131_149ab9d7b6d3"
name="Glutamine" compartment="mwfd39d73a_90ba_47dc_8fea_568ef27807f9"
initialConcentration="0"
substanceUnits="MWBUILTINPREFIX_milli_MWBUILTINUNIT_mole"
hasOnlySubstanceUnits="false" boundaryCondition="false"
constant="false">
        <annotation>
          <SimBiology xmlns="http://www.mathworks.com">
            <Unit Numerator="millimole" Denominator="liter"/>
          </SimBiology>
        </annotation>
      </species>
      <species id="mw7f09c5aa_2a3b_47a0_9b81_79465a7f9739"
name="Urea_Ex" compartment="mwf128b3a7_6c40_40ea_8ba0_9f5355e0337b"
initialConcentration="0"
substanceUnits="MWBUILTINPREFIX_milli_MWBUILTINUNIT_mole"
hasOnlySubstanceUnits="false" boundaryCondition="false"
constant="false">
        <annotation>
          <SimBiology xmlns="http://www.mathworks.com">
            <Unit Numerator="millimole" Denominator="liter"/>
          </SimBiology>
        </annotation>
      </species>
      <species id="mw35a92c89_c99d_4c99_84de_bc6395c9b628" name="eNH3"
compartment="mwf128b3a7_6c40_40ea_8ba0_9f5355e0337b"
initialConcentration="0"
substanceUnits="MWBUILTINPREFIX_milli_MWBUILTINUNIT_mole"
hasOnlySubstanceUnits="false" boundaryCondition="false"
constant="false">
        <annotation>
          <SimBiology xmlns="http://www.mathworks.com">
            <Unit Numerator="millimole" Denominator="liter"/>
          </SimBiology>
        </annotation>
      </species>
      <species id="mwab682afe_b6ab_4431_a950_c9e9adc5ce9d"
name="NH3_I" compartment="mw100b8e10_19a9_464f_84ca_67b25ed1ded9"
initialAmount="708.377"
substanceUnits="MWBUILTINPREFIX_milli_MWBUILTINUNIT_mole"
```

```
hasOnlySubstanceUnits="true" boundaryCondition="false"
constant="false"/>
    </listOfSpecies>
    <listOfParameters>
      <parameter id="mw981d2085_3b27_41f8_8dc0_f785a5f8ae63"
name="U_Ex" value="0.244" units="MWDERIVEDUNIT_millimole__minute"
constant="true"/>
      <parameter id="mw1b4ea136_44e0_4f90_89a9_039b2e243b03"
name="NH3_Ex" value="0.004" units="MWDERIVEDUNIT_millimole__minute"
constant="true"/>
      <parameter id="mwff92591c_564f_4e8b_99d3_fa908ba56b83"
name="NH3_Abs" value="0.492" units="MWDERIVEDUNIT_millimole__minute"
constant="true"/>
      <parameter id="mw7d178aae_ff78_412a_830f_3be325d22773" name="T1"
value="1" units="dimensionless" constant="false"/>
      <parameter id="mwcedbe49e_2d28_4720_8fcd_207db64228cf" name="T2"
value="0" units="dimensionless" constant="false"/>
    </listOfParameters>
    <listOfReactions>
      <reaction id="mw13114aa6_1c63_4cf1_b524_7a06e3611e2d" name="Gln-
Syn" reversible="false" fast="false">
        <listOfReactants>
          <speciesReference
species="mwd508e9bd_ed74_425d_9880_17e95bd7a346" stoichiometry="1"/>
        </listOfReactants>
        <listOfProducts>
          <speciesReference
species="mwc8acca22_48dd_47bf_9131_149ab9d7b6d3" stoichiometry="1"/>
        </listOfProducts>
        <kineticLaw>
          <math xmlns="http://www.w3.org/1998/Math/MathML">
            <apply>
              <times/>
              <apply>
                <divide/>
                <apply>
                  <times/>
                  <ci> mw1b7d884f_e321_44c2_b2ea_a64bc054c885 </ci>
                  <ci> mwd508e9bd_ed74_425d_9880_17e95bd7a346 </ci>
                </apply>
                <apply>
                  <plus/>
                  <apply>
                    <times/>
                    <ci> mwc5d8a093_feff_4408_be62_5daceef23a29 </ci>
                    <apply>
                      <plus/>
                      <cn type="integer"> 1 </cn>
                      <apply>
                        <divide/>
                        <ci> mwc8acca22_48dd_47bf_9131_149ab9d7b6d3
</ci>
```

```
                        <ci> mwc73ef044_61af_4c1a_a2a8_c16204fcbf60
</ci>
                      </apply>
                    </apply>
                  </apply>
                  <ci> mwd508e9bd_ed74_425d_9880_17e95bd7a346 </ci>
                </apply>
              </apply>
              <ci> mwcedbe49e_2d28_4720_8fcd_207db64228cf </ci>
            </apply>
          </math>
          <listOfParameters>
            <parameter id="mw1b7d884f_e321_44c2_b2ea_a64bc054c885"
name="Vm" value="12.3" units="MWDERIVEDUNIT_millimole__minute"
constant="true"/>
            <parameter id="mwc5d8a093_feff_4408_be62_5daceef23a29"
name="KmNH3" value="0.15" units="MWDERIVEDUNIT_millimole__liter"
constant="true"/>
            <parameter id="mwc73ef044_61af_4c1a_a2a8_c16204fcbf60"
name="KiGln" value="0.6" units="MWDERIVEDUNIT_millimole__liter"
constant="true"/>
          </listOfParameters>
        </kineticLaw>
      </reaction>
      <reaction id="mw8908cbdb_bfa4_477c_8b58_ad304f305c43" name="CPS"
reversible="false" fast="false">
        <listOfReactants>
          <speciesReference
species="mwd508e9bd_ed74_425d_9880_17e95bd7a346" stoichiometry="1"/>
        </listOfReactants>
        <listOfProducts>
          <speciesReference
species="mwa6f6ed3a_00c3_49a0_b7f2_32da70289968" stoichiometry="1"/>
        </listOfProducts>
        <kineticLaw>
          <math xmlns="http://www.w3.org/1998/Math/MathML">
            <apply>
              <times/>
              <apply>
                <divide/>
                <apply>
                  <times/>
                  <ci> mwb4f7246b_7e4f_4a57_866c_c8fd5d2c5ce7 </ci>
                  <ci> mwd508e9bd_ed74_425d_9880_17e95bd7a346 </ci>
                </apply>
                <apply>
                  <plus/>
                  <ci> mwf5aa6d7d_25c3_4b69_aada_2049ff80625d </ci>
                  <ci> mwd508e9bd_ed74_425d_9880_17e95bd7a346 </ci>
                </apply>
              </apply>
              <ci> mw7d178aae_ff78_412a_830f_3be325d22773 </ci>
```

```
          </apply>
        </math>
        <listOfParameters>
          <parameter id="mwb4f7246b_7e4f_4a57_866c_c8fd5d2c5ce7"
name="Vm" value="8.05" units="MWDERIVEDUNIT_millimole__minute"
constant="true"/>
          <parameter id="mwf5aa6d7d_25c3_4b69_aada_2049ff80625d"
name="Km" value="0.35" units="MWDERIVEDUNIT_millimole__liter"
constant="true"/>
        </listOfParameters>
      </kineticLaw>
    </reaction>
    <reaction id="mw4a38d9ee_ee6d_4010_a9fa_4e37e3fbadef"
name="U_Ex" reversible="false" fast="false">
      <listOfReactants>
        <speciesReference
species="mwa6f6ed3a_00c3_49a0_b7f2_32da70289968" stoichiometry="1"/>
      </listOfReactants>
      <listOfProducts>
        <speciesReference
species="mw7f09c5aa_2a3b_47a0_9b81_79465a7f9739" stoichiometry="1"/>
      </listOfProducts>
      <kineticLaw>
        <math xmlns="http://www.w3.org/1998/Math/MathML">
          <ci> mw981d2085_3b27_41f8_8dc0_f785a5f8ae63 </ci>
        </math>
      </kineticLaw>
    </reaction>
    <reaction id="mw2097ca99_0100_4364_8b13_b55006dd6423"
name="NH3_Ex" reversible="false" fast="false">
      <listOfReactants>
        <speciesReference
species="mwd508e9bd_ed74_425d_9880_17e95bd7a346" stoichiometry="1"/>
      </listOfReactants>
      <listOfProducts>
        <speciesReference
species="mw35a92c89_c99d_4c99_84de_bc6395c9b628" stoichiometry="1"/>
      </listOfProducts>
      <kineticLaw>
        <math xmlns="http://www.w3.org/1998/Math/MathML">
          <ci> mw1b4ea136_44e0_4f90_89a9_039b2e243b03 </ci>
        </math>
      </kineticLaw>
    </reaction>
    <reaction id="mw5d2908f4_2177_4e58_badb_62dc7e83d254"
name="NH3_Abs" reversible="false" fast="false">
      <listOfReactants>
        <speciesReference
species="mwab682afe_b6ab_4431_a950_c9e9adc5ce9d" stoichiometry="1"/>
      </listOfReactants>
      <listOfProducts>
```

```
          <speciesReference
species="mwd508e9bd_ed74_425d_9880_17e95bd7a346" stoichiometry="1"/>
        </listOfProducts>
        <kineticLaw>
          <math xmlns="http://www.w3.org/1998/Math/MathML">
            <ci> mwff92591c_564f_4e8b_99d3_fa908ba56b83 </ci>
          </math>
        </kineticLaw>
      </reaction>
      <reaction id="mwdc104551_3d23_4e5b_9f84_765fcd396b95"
reversible="false" fast="false">
        <listOfReactants>
          <speciesReference
species="mwd508e9bd_ed74_425d_9880_17e95bd7a346" stoichiometry="1"/>
        </listOfReactants>
        <listOfProducts>
          <speciesReference
species="mw5ffd8486_9941_4b71_8698_d5d06edc44df" stoichiometry="1"/>
        </listOfProducts>
        <kineticLaw>
          <math xmlns="http://www.w3.org/1998/Math/MathML">
            <apply>
              <times/>
              <apply>
                <divide/>
                <apply>
                  <times/>
                  <ci> mw35fd8bfe_83a8_4863_8a4d_21a0a356d3d6 </ci>
                  <ci> mwd508e9bd_ed74_425d_9880_17e95bd7a346 </ci>
                </apply>
                <apply>
                  <plus/>
                  <ci> mw02557780_2003_4572_b384_5f9d85ff6372 </ci>
                  <ci> mwd508e9bd_ed74_425d_9880_17e95bd7a346 </ci>
                </apply>
              </apply>
              <ci> mw7d178aae_ff78_412a_830f_3be325d22773 </ci>
            </apply>
          </math>
          <listOfParameters>
            <parameter id="mw35fd8bfe_83a8_4863_8a4d_21a0a356d3d6"
name="Vm" value="8.05" units="MWDERIVEDUNIT_millimole__minute"
constant="true"/>
            <parameter id="mw02557780_2003_4572_b384_5f9d85ff6372"
name="KmNH3" value="0.35" units="MWDERIVEDUNIT_millimole__liter"
constant="true"/>
          </listOfParameters>
        </kineticLaw>
      </reaction>
      <reaction id="mw28a665bc_34e6_4502_897c_24ee0a0fbfce"
name="mGln-ase" reversible="false" fast="false">
        <listOfReactants>
```

```
          <speciesReference
species="mwc8acca22_48dd_47bf_9131_149ab9d7b6d3" stoichiometry="1"/>
        </listOfReactants>
        <listOfProducts>
          <speciesReference
species="mwd508e9bd_ed74_425d_9880_17e95bd7a346" stoichiometry="1"/>
        </listOfProducts>
        <kineticLaw>
          <math xmlns="http://www.w3.org/1998/Math/MathML">
            <apply>
              <times/>
              <apply>
                <divide/>
                <apply>
                  <times/>
                  <ci> mw236df257_360a_4d52_a6c4_da5a86625cfb </ci>
                  <ci> mwc8acca22_48dd_47bf_9131_149ab9d7b6d3 </ci>
                </apply>
                <apply>
                  <plus/>
                  <ci> mw090b8862_c0b5_4136_9f05_a809fb725b9c </ci>
                  <ci> mwc8acca22_48dd_47bf_9131_149ab9d7b6d3 </ci>
                </apply>
              </apply>
              <ci> mw7d178aae_ff78_412a_830f_3be325d22773 </ci>
            </apply>
          </math>
          <listOfParameters>
            <parameter id="mw236df257_360a_4d52_a6c4_da5a86625cfb"
name="Vm" value="28.54" units="MWDERIVEDUNIT_millimole__minute"
constant="true"/>
            <parameter id="mw090b8862_c0b5_4136_9f05_a809fb725b9c"
name="Kmgln" value="4" units="MWDERIVEDUNIT_millimole__liter"
constant="true"/>
          </listOfParameters>
        </kineticLaw>
      </reaction>
    </listOfReactions>
    <listOfEvents>
      <event id="mwf55002f5_38b2_4c8e_a7ed_dad0f3fb45ed"
name="event_1">
        <trigger>
          <math xmlns="http://www.w3.org/1998/Math/MathML">
            <apply>
              <geq/>
              <csymbol encoding="text"
definitionURL="http://www.sbml.org/sbml/symbols/time"> time </csymbol>
              <cn> 2.87 </cn>
            </apply>
          </math>
        </trigger>
        <listOfEventAssignments>
```

```
          <eventAssignment
variable="mw7d178aae_ff78_412a_830f_3be325d22773">
            &lt;math xmlns="http://www.w3.org/1998/Math/MathML"&gt;
              &lt;cn type="integer"&gt; 0 &lt;/cn&gt;
            &lt;/math&gt;
          &lt;/eventAssignment&gt;
        &lt;/listOfEventAssignments&gt;
      &lt;/event&gt;
      <event id="mwfe735dbc_904c_458c_8f8c_ce4b02458d49"
name="event_2">
        &lt;trigger&gt;
          &lt;math xmlns="http://www.w3.org/1998/Math/MathML"&gt;
            &lt;apply&gt;
              &lt;geq/&gt;
              <csymbol encoding="text"
definitionURL="http://www.sbml.org/sbml/symbols/time"> time &lt;/csymbol&gt;
              &lt;cn&gt; 2.87 &lt;/cn&gt;
            &lt;/apply&gt;
          &lt;/math&gt;
        &lt;/trigger&gt;
        &lt;listOfEventAssignments&gt;
          <eventAssignment
variable="mwcedbe49e_2d28_4720_8fcd_207db64228cf">
            &lt;math xmlns="http://www.w3.org/1998/Math/MathML"&gt;
              &lt;cn type="integer"&gt; 1 &lt;/cn&gt;
            &lt;/math&gt;
          &lt;/eventAssignment&gt;
        &lt;/listOfEventAssignments&gt;
      &lt;/event&gt;
      <event id="mw5bd35bbd_7a5c_4971_8cf1_d231c142f05d"
name="event_3">
        &lt;trigger&gt;
          &lt;math xmlns="http://www.w3.org/1998/Math/MathML"&gt;
            &lt;apply&gt;
              &lt;geq/&gt;
              <csymbol encoding="text"
definitionURL="http://www.sbml.org/sbml/symbols/time"> time &lt;/csymbol&gt;
              &lt;cn&gt; 4.3 &lt;/cn&gt;
            &lt;/apply&gt;
          &lt;/math&gt;
        &lt;/trigger&gt;
        &lt;listOfEventAssignments&gt;
          <eventAssignment
variable="mw7d178aae_ff78_412a_830f_3be325d22773">
            &lt;math xmlns="http://www.w3.org/1998/Math/MathML"&gt;
              &lt;cn type="integer"&gt; 1 &lt;/cn&gt;
            &lt;/math&gt;
          &lt;/eventAssignment&gt;
        &lt;/listOfEventAssignments&gt;
      &lt;/event&gt;
      <event id="mw265b43b9_b63e_4c8e_bff7_aeddcb7aae84"
name="event_4">
```

```
        <trigger>
          <math xmlns="http://www.w3.org/1998/Math/MathML">
            <apply>
              <geq/>
              <csymbol encoding="text"
definitionURL="http://www.sbml.org/sbml/symbols/time"> time </csymbol>
              <cn> 4.3 </cn>
            </apply>
          </math>
        </trigger>
        <listOfEventAssignments>
          <eventAssignment
variable="mwcedbe49e_2d28_4720_8fcd_207db64228cf">
            <math xmlns="http://www.w3.org/1998/Math/MathML">
              <cn type="integer"> 0 </cn>
            </math>
          </eventAssignment>
        </listOfEventAssignments>
      </event>
      <event id="mwb385e0f1_a89c_442f_832a_b5df1c14d835"
name="event_5">
        <trigger>
          <math xmlns="http://www.w3.org/1998/Math/MathML">
            <apply>
              <geq/>
              <csymbol encoding="text"
definitionURL="http://www.sbml.org/sbml/symbols/time"> time </csymbol>
              <cn> 7.17 </cn>
            </apply>
          </math>
        </trigger>
        <listOfEventAssignments>
          <eventAssignment
variable="mw7d178aae_ff78_412a_830f_3be325d22773">
            <math xmlns="http://www.w3.org/1998/Math/MathML">
              <cn type="integer"> 0 </cn>
            </math>
          </eventAssignment>
        </listOfEventAssignments>
      </event>
      <event id="mw104b1e99_44c5_46e1_bbcd_71f9a65724d3"
name="event_6">
        <trigger>
          <math xmlns="http://www.w3.org/1998/Math/MathML">
            <apply>
              <geq/>
              <csymbol encoding="text"
definitionURL="http://www.sbml.org/sbml/symbols/time"> time </csymbol>
              <cn> 7.17 </cn>
            </apply>
          </math>
        </trigger>
```

```
        <listOfEventAssignments>
          <eventAssignment 
variable="mwcedbe49e_2d28_4720_8fcd_207db64228cf">
            <math xmlns="http://www.w3.org/1998/Math/MathML">
              <cn type="integer"> 1 </cn>
            </math>
          </eventAssignment>
        </listOfEventAssignments>
      </event>
      <event id="mw3c4fbd8d_d182_48d9_9b12_8bb34a083290" 
name="event_7">
        <trigger>
          <math xmlns="http://www.w3.org/1998/Math/MathML">
            <apply>
              <geq/>
              <csymbol encoding="text" 
definitionURL="http://www.sbml.org/sbml/symbols/time"> time </csymbol>
              <cn> 8.6 </cn>
            </apply>
          </math>
        </trigger>
        <listOfEventAssignments>
          <eventAssignment 
variable="mw7d178aae_ff78_412a_830f_3be325d22773">
            <math xmlns="http://www.w3.org/1998/Math/MathML">
              <cn type="integer"> 1 </cn>
            </math>
          </eventAssignment>
        </listOfEventAssignments>
      </event>
      <event id="mwfc21e175_aa2e_4e77_a624_33415a0c5b7d" 
name="event_8">
        <trigger>
          <math xmlns="http://www.w3.org/1998/Math/MathML">
            <apply>
              <geq/>
              <csymbol encoding="text" 
definitionURL="http://www.sbml.org/sbml/symbols/time"> time </csymbol>
              <cn> 8.6 </cn>
            </apply>
          </math>
        </trigger>
        <listOfEventAssignments>
          <eventAssignment 
variable="mwcedbe49e_2d28_4720_8fcd_207db64228cf">
            <math xmlns="http://www.w3.org/1998/Math/MathML">
              <cn type="integer"> 0 </cn>
            </math>
          </eventAssignment>
        </listOfEventAssignments>
      </event>
```

```
      <event id="mw1eb2ba39_fa3e_4419_a1bc_d8911a7aed0c"
name="event_9">
        <trigger>
          <math xmlns="http://www.w3.org/1998/Math/MathML">
            <apply>
              <geq/>
              <csymbol encoding="text"
definitionURL="http://www.sbml.org/sbml/symbols/time"> time </csymbol>
              <cn> 11.47 </cn>
            </apply>
          </math>
        </trigger>
        <listOfEventAssignments>
          <eventAssignment
variable="mw7d178aae_ff78_412a_830f_3be325d22773">
            <math xmlns="http://www.w3.org/1998/Math/MathML">
              <cn type="integer"> 0 </cn>
            </math>
          </eventAssignment>
        </listOfEventAssignments>
      </event>
      <event id="mwc0054416_aeb3_4943_823d_c45d44633ad5"
name="event_10">
        <trigger>
          <math xmlns="http://www.w3.org/1998/Math/MathML">
            <apply>
              <geq/>
              <csymbol encoding="text"
definitionURL="http://www.sbml.org/sbml/symbols/time"> time </csymbol>
              <cn> 11.47 </cn>
            </apply>
          </math>
        </trigger>
        <listOfEventAssignments>
          <eventAssignment
variable="mwcedbe49e_2d28_4720_8fcd_207db64228cf">
            <math xmlns="http://www.w3.org/1998/Math/MathML">
              <cn type="integer"> 1 </cn>
            </math>
          </eventAssignment>
        </listOfEventAssignments>
      </event>
      <event id="mw8ab78393_8ef8_4a2a_b860_a31352ef120d"
name="event_11">
        <trigger>
          <math xmlns="http://www.w3.org/1998/Math/MathML">
            <apply>
              <geq/>
              <csymbol encoding="text"
definitionURL="http://www.sbml.org/sbml/symbols/time"> time </csymbol>
              <cn> 12.9 </cn>
            </apply>
```

```
          </math>
        </trigger>
        <listOfEventAssignments>
          <eventAssignment
variable="mw7d178aae_ff78_412a_830f_3be325d22773">
            <math xmlns="http://www.w3.org/1998/Math/MathML">
              <cn type="integer"> 1 </cn>
            </math>
          </eventAssignment>
        </listOfEventAssignments>
      </event>
      <event id="mwc634f082_8c31_409a_ad5f_40f483753730"
name="event_12">
        <trigger>
          <math xmlns="http://www.w3.org/1998/Math/MathML">
            <apply>
              <geq/>
              <csymbol encoding="text"
definitionURL="http://www.sbml.org/sbml/symbols/time"> time </csymbol>
              <cn> 12.9 </cn>
            </apply>
          </math>
        </trigger>
        <listOfEventAssignments>
          <eventAssignment
variable="mwcedbe49e_2d28_4720_8fcd_207db64228cf">
            <math xmlns="http://www.w3.org/1998/Math/MathML">
              <cn type="integer"> 0 </cn>
            </math>
          </eventAssignment>
        </listOfEventAssignments>
      </event>
      <event id="mw578b549c_2558_42d7_9e12_dbfc0f935c6e"
name="event_13">
        <trigger>
          <math xmlns="http://www.w3.org/1998/Math/MathML">
            <apply>
              <geq/>
              <csymbol encoding="text"
definitionURL="http://www.sbml.org/sbml/symbols/time"> time </csymbol>
              <cn> 15.77 </cn>
            </apply>
          </math>
        </trigger>
        <listOfEventAssignments>
          <eventAssignment
variable="mw7d178aae_ff78_412a_830f_3be325d22773">
            <math xmlns="http://www.w3.org/1998/Math/MathML">
              <cn type="integer"> 0 </cn>
            </math>
          </eventAssignment>
        </listOfEventAssignments>
```

```
      </event>
      <event id="mwfb458cbc_f946_40e1_a67f_bd26eab14ee0"
name="event_14">
        <trigger>
          <math xmlns="http://www.w3.org/1998/Math/MathML">
            <apply>
              <geq/>
              <csymbol encoding="text"
definitionURL="http://www.sbml.org/sbml/symbols/time"> time </csymbol>
              <cn> 15.77 </cn>
            </apply>
          </math>
        </trigger>
        <listOfEventAssignments>
          <eventAssignment
variable="mwcedbe49e_2d28_4720_8fcd_207db64228cf">
            <math xmlns="http://www.w3.org/1998/Math/MathML">
              <cn type="integer"> 1 </cn>
            </math>
          </eventAssignment>
        </listOfEventAssignments>
      </event>
      <event id="mwd1c74746_cc83_49b3_be60_642f0d331597"
name="event_15">
        <trigger>
          <math xmlns="http://www.w3.org/1998/Math/MathML">
            <apply>
              <geq/>
              <csymbol encoding="text"
definitionURL="http://www.sbml.org/sbml/symbols/time"> time </csymbol>
              <cn> 17.2 </cn>
            </apply>
          </math>
        </trigger>
        <listOfEventAssignments>
          <eventAssignment
variable="mwcedbe49e_2d28_4720_8fcd_207db64228cf">
            <math xmlns="http://www.w3.org/1998/Math/MathML">
              <cn type="integer"> 0 </cn>
            </math>
          </eventAssignment>
        </listOfEventAssignments>
      </event>
      <event id="mw9770404e_b9f3_41e0_9b1a_a934107f8abb"
name="event_16">
        <trigger>
          <math xmlns="http://www.w3.org/1998/Math/MathML">
            <apply>
              <geq/>
              <csymbol encoding="text"
definitionURL="http://www.sbml.org/sbml/symbols/time"> time </csymbol>
              <cn> 17.2 </cn>
```

```
            </apply>
          </math>
        </trigger>
        <listOfEventAssignments>
          <eventAssignment
variable="mw7d178aae_ff78_412a_830f_3be325d22773">
            <math xmlns="http://www.w3.org/1998/Math/MathML">
              <cn type="integer"> 1 </cn>
            </math>
          </eventAssignment>
        </listOfEventAssignments>
      </event>
      <event id="mwe5464dfe_5cf8_4b97_a70a_c1585323cccf"
name="event_17">
        <trigger>
          <math xmlns="http://www.w3.org/1998/Math/MathML">
            <apply>
              <geq/>
              <csymbol encoding="text"
definitionURL="http://www.sbml.org/sbml/symbols/time"> time </csymbol>
              <cn> 20.07 </cn>
            </apply>
          </math>
        </trigger>
        <listOfEventAssignments>
          <eventAssignment
variable="mw7d178aae_ff78_412a_830f_3be325d22773">
            <math xmlns="http://www.w3.org/1998/Math/MathML">
              <cn type="integer"> 0 </cn>
            </math>
          </eventAssignment>
        </listOfEventAssignments>
      </event>
      <event id="mw4d877c56_d206_433c_bfdc_f186f65847e1"
name="event_18">
        <trigger>
          <math xmlns="http://www.w3.org/1998/Math/MathML">
            <apply>
              <geq/>
              <csymbol encoding="text"
definitionURL="http://www.sbml.org/sbml/symbols/time"> time </csymbol>
              <cn> 21.5 </cn>
            </apply>
          </math>
        </trigger>
        <listOfEventAssignments>
          <eventAssignment
variable="mw7d178aae_ff78_412a_830f_3be325d22773">
            <math xmlns="http://www.w3.org/1998/Math/MathML">
              <cn type="integer"> 1 </cn>
            </math>
          </eventAssignment>
```

```
        </listOfEventAssignments>
      </event>
      <event id="mweeab42ee_6c81_4b09_9244_275b949ba07a"
name="event_19">
        <trigger>
          <math xmlns="http://www.w3.org/1998/Math/MathML">
            <apply>
              <geq/>
              <csymbol encoding="text"
definitionURL="http://www.sbml.org/sbml/symbols/time"> time </csymbol>
              <cn> 20.07 </cn>
            </apply>
          </math>
        </trigger>
        <listOfEventAssignments>
          <eventAssignment
variable="mwcedbe49e_2d28_4720_8fcd_207db64228cf">
            <math xmlns="http://www.w3.org/1998/Math/MathML">
              <cn type="integer"> 1 </cn>
            </math>
          </eventAssignment>
        </listOfEventAssignments>
      </event>
      <event id="mwa952952c_46f4_444b_8128_e414d2835b7b"
name="event_20">
        <trigger>
          <math xmlns="http://www.w3.org/1998/Math/MathML">
            <apply>
              <geq/>
              <csymbol encoding="text"
definitionURL="http://www.sbml.org/sbml/symbols/time"> time </csymbol>
              <cn> 21.5 </cn>
            </apply>
          </math>
        </trigger>
        <listOfEventAssignments>
          <eventAssignment
variable="mwcedbe49e_2d28_4720_8fcd_207db64228cf">
            <math xmlns="http://www.w3.org/1998/Math/MathML">
              <cn type="integer"> 0 </cn>
            </math>
          </eventAssignment>
        </listOfEventAssignments>
      </event>
      <event id="mw0161deee_80a1_40bf_add7_2ca1f03aae71"
name="event_21">
        <trigger>
          <math xmlns="http://www.w3.org/1998/Math/MathML">
            <apply>
              <geq/>
              <csymbol encoding="text"
definitionURL="http://www.sbml.org/sbml/symbols/time"> time </csymbol>
```

```
              <cn> 24.37 </cn>
            </apply>
          </math>
        </trigger>
        <listOfEventAssignments>
          <eventAssignment
variable="mw7d178aae_ff78_412a_830f_3be325d22773">
            <math xmlns="http://www.w3.org/1998/Math/MathML">
              <cn type="integer"> 0 </cn>
            </math>
          </eventAssignment>
        </listOfEventAssignments>
      </event>
      <event id="mwd536caa3_4b92_4bca_ae13_a49a0ac1ca1a"
name="event_22">
        <trigger>
          <math xmlns="http://www.w3.org/1998/Math/MathML">
            <apply>
              <geq/>
              <csymbol encoding="text"
definitionURL="http://www.sbml.org/sbml/symbols/time"> time </csymbol>
              <cn> 25.8 </cn>
            </apply>
          </math>
        </trigger>
        <listOfEventAssignments>
          <eventAssignment
variable="mw7d178aae_ff78_412a_830f_3be325d22773">
            <math xmlns="http://www.w3.org/1998/Math/MathML">
              <cn type="integer"> 1 </cn>
            </math>
          </eventAssignment>
        </listOfEventAssignments>
      </event>
      <event id="mw38e94287_8271_407a_b656_eeab537e943e"
name="event_23">
        <trigger>
          <math xmlns="http://www.w3.org/1998/Math/MathML">
            <apply>
              <geq/>
              <csymbol encoding="text"
definitionURL="http://www.sbml.org/sbml/symbols/time"> time </csymbol>
              <cn> 28.67 </cn>
            </apply>
          </math>
        </trigger>
        <listOfEventAssignments>
          <eventAssignment
variable="mw7d178aae_ff78_412a_830f_3be325d22773">
            <math xmlns="http://www.w3.org/1998/Math/MathML">
              <cn type="integer"> 0 </cn>
            </math>
```

```
          &lt;/eventAssignment&gt;
        &lt;/listOfEventAssignments&gt;
      &lt;/event&gt;
      <event id="mw72acefbf_69e7_49ce_8a3b_d1ff561f896a"
name="event_24">
        &lt;trigger&gt;
          &lt;math xmlns="http://www.w3.org/1998/Math/MathML"&gt;
            &lt;apply&gt;
              &lt;geq/&gt;
              <csymbol encoding="text"
definitionURL="http://www.sbml.org/sbml/symbols/time"> time &lt;/csymbol&gt;
              &lt;cn&gt; 24.37 &lt;/cn&gt;
            &lt;/apply&gt;
          &lt;/math&gt;
        &lt;/trigger&gt;
        &lt;listOfEventAssignments&gt;
          <eventAssignment
variable="mwcedbe49e_2d28_4720_8fcd_207db64228cf">
            &lt;math xmlns="http://www.w3.org/1998/Math/MathML"&gt;
              &lt;cn type="integer"&gt; 1 &lt;/cn&gt;
            &lt;/math&gt;
          &lt;/eventAssignment&gt;
        &lt;/listOfEventAssignments&gt;
      &lt;/event&gt;
      <event id="mwd6cd3d51_b96b_4050_9d01_5ceb0ea89a12"
name="event_25">
        &lt;trigger&gt;
          &lt;math xmlns="http://www.w3.org/1998/Math/MathML"&gt;
            &lt;apply&gt;
              &lt;geq/&gt;
              <csymbol encoding="text"
definitionURL="http://www.sbml.org/sbml/symbols/time"> time &lt;/csymbol&gt;
              &lt;cn&gt; 25.8 &lt;/cn&gt;
            &lt;/apply&gt;
          &lt;/math&gt;
        &lt;/trigger&gt;
        &lt;listOfEventAssignments&gt;
          <eventAssignment
variable="mwcedbe49e_2d28_4720_8fcd_207db64228cf">
            &lt;math xmlns="http://www.w3.org/1998/Math/MathML"&gt;
              &lt;cn type="integer"&gt; 0 &lt;/cn&gt;
            &lt;/math&gt;
          &lt;/eventAssignment&gt;
        &lt;/listOfEventAssignments&gt;
      &lt;/event&gt;
      <event id="mw963416cf_ddde_4462_88a8_e3844dd537e4"
name="event_26">
        &lt;trigger&gt;
          &lt;math xmlns="http://www.w3.org/1998/Math/MathML"&gt;
            &lt;apply&gt;
              &lt;geq/&gt;
```

```
              <csymbol encoding="text"
definitionURL="http://www.sbml.org/sbml/symbols/time"> time </csymbol>
              <cn> 28.67 </cn>
            </apply>
          </math>
        </trigger>
        <listOfEventAssignments>
          <eventAssignment
variable="mwcedbe49e_2d28_4720_8fcd_207db64228cf">
            <math xmlns="http://www.w3.org/1998/Math/MathML">
              <cn type="integer"> 1 </cn>
            </math>
          </eventAssignment>
        </listOfEventAssignments>
      </event>
      <event id="mwd025c23f_b54f_4d17_aa68_488432e445d6"
name="event_27">
        <trigger>
          <math xmlns="http://www.w3.org/1998/Math/MathML">
            <apply>
              <geq/>
              <csymbol encoding="text"
definitionURL="http://www.sbml.org/sbml/symbols/time"> time </csymbol>
              <cn> 30.1 </cn>
            </apply>
          </math>
        </trigger>
        <listOfEventAssignments>
          <eventAssignment
variable="mwcedbe49e_2d28_4720_8fcd_207db64228cf">
            <math xmlns="http://www.w3.org/1998/Math/MathML">
              <cn type="integer"> 0 </cn>
            </math>
          </eventAssignment>
        </listOfEventAssignments>
      </event>
      <event id="mwceb7e9de_5bd5_4de4_8359_de0661432719"
name="event_28">
        <trigger>
          <math xmlns="http://www.w3.org/1998/Math/MathML">
            <apply>
              <geq/>
              <csymbol encoding="text"
definitionURL="http://www.sbml.org/sbml/symbols/time"> time </csymbol>
              <cn> 32.97 </cn>
            </apply>
          </math>
        </trigger>
        <listOfEventAssignments>
          <eventAssignment
variable="mwcedbe49e_2d28_4720_8fcd_207db64228cf">
            <math xmlns="http://www.w3.org/1998/Math/MathML">
```

```
            <cn type="integer"> 1 </cn>
          </math>
        </eventAssignment>
      </listOfEventAssignments>
    </event>
    <event id="mw0bbed59b_0297_4f30_a3b7_86d367f8fcd7"
name="event_29">
      <trigger>
        <math xmlns="http://www.w3.org/1998/Math/MathML">
          <apply>
            <geq/>
            <csymbol encoding="text"
definitionURL="http://www.sbml.org/sbml/symbols/time"> time </csymbol>
            <cn> 34.4 </cn>
          </apply>
        </math>
      </trigger>
      <listOfEventAssignments>
        <eventAssignment
variable="mwcedbe49e_2d28_4720_8fcd_207db64228cf">
          <math xmlns="http://www.w3.org/1998/Math/MathML">
            <cn type="integer"> 0 </cn>
          </math>
        </eventAssignment>
      </listOfEventAssignments>
    </event>
    <event id="mw43eef1ac_609c_42dc_be65_2f51bbfe9ebc"
name="event_30">
      <trigger>
        <math xmlns="http://www.w3.org/1998/Math/MathML">
          <apply>
            <geq/>
            <csymbol encoding="text"
definitionURL="http://www.sbml.org/sbml/symbols/time"> time </csymbol>
            <cn> 30.1 </cn>
          </apply>
        </math>
      </trigger>
      <listOfEventAssignments>
        <eventAssignment
variable="mw7d178aae_ff78_412a_830f_3be325d22773">
          <math xmlns="http://www.w3.org/1998/Math/MathML">
            <cn type="integer"> 1 </cn>
          </math>
        </eventAssignment>
      </listOfEventAssignments>
    </event>
    <event id="mw40701cc8_a29d_42ff_9d63_ee1892da7d0e"
name="event_31">
      <trigger>
        <math xmlns="http://www.w3.org/1998/Math/MathML">
          <apply>
```

```
            &lt;geq/&gt;
            <csymbol encoding="text"
definitionURL="http://www.sbml.org/sbml/symbols/time"> time &lt;/csymbol&gt;
            &lt;cn&gt; 32.97 &lt;/cn&gt;
          &lt;/apply&gt;
        &lt;/math&gt;
      &lt;/trigger&gt;
      &lt;listOfEventAssignments&gt;
        <eventAssignment
variable="mw7d178aae_ff78_412a_830f_3be325d22773">
          &lt;math xmlns="http://www.w3.org/1998/Math/MathML"&gt;
            &lt;cn type="integer"&gt; 0 &lt;/cn&gt;
          &lt;/math&gt;
        &lt;/eventAssignment&gt;
      &lt;/listOfEventAssignments&gt;
    &lt;/event&gt;
    <event id="mw5795bb56_a394_485d_9337_5803ec31a3d4"
name="event_32">
      &lt;trigger&gt;
        &lt;math xmlns="http://www.w3.org/1998/Math/MathML"&gt;
          &lt;apply&gt;
            &lt;geq/&gt;
            <csymbol encoding="text"
definitionURL="http://www.sbml.org/sbml/symbols/time"> time &lt;/csymbol&gt;
            &lt;cn&gt; 34.4 &lt;/cn&gt;
          &lt;/apply&gt;
        &lt;/math&gt;
      &lt;/trigger&gt;
      &lt;listOfEventAssignments&gt;
        <eventAssignment
variable="mw7d178aae_ff78_412a_830f_3be325d22773">
          &lt;math xmlns="http://www.w3.org/1998/Math/MathML"&gt;
            &lt;cn type="integer"&gt; 1 &lt;/cn&gt;
          &lt;/math&gt;
        &lt;/eventAssignment&gt;
      &lt;/listOfEventAssignments&gt;
    &lt;/event&gt;
    <event id="mwc23ae716_6118_4516_ba30_f687540e09e4"
name="event_33">
      &lt;trigger&gt;
        &lt;math xmlns="http://www.w3.org/1998/Math/MathML"&gt;
          &lt;apply&gt;
            &lt;geq/&gt;
            <csymbol encoding="text"
definitionURL="http://www.sbml.org/sbml/symbols/time"> time &lt;/csymbol&gt;
            &lt;cn&gt; 37.27 &lt;/cn&gt;
          &lt;/apply&gt;
        &lt;/math&gt;
      &lt;/trigger&gt;
      &lt;listOfEventAssignments&gt;
        <eventAssignment
variable="mw7d178aae_ff78_412a_830f_3be325d22773">
```

```
            <math xmlns="http://www.w3.org/1998/Math/MathML">
              <cn type="integer"> 0 </cn>
            </math>
          </eventAssignment>
        </listOfEventAssignments>
      </event>
      <event id="mwa8d1e743_c4c8_425d_8d99_395f57911b66"
name="event_34">
        <trigger>
          <math xmlns="http://www.w3.org/1998/Math/MathML">
            <apply>
              <geq/>
              <csymbol encoding="text"
definitionURL="http://www.sbml.org/sbml/symbols/time"> time </csymbol>
              <cn> 38.7 </cn>
            </apply>
          </math>
        </trigger>
        <listOfEventAssignments>
          <eventAssignment
variable="mw7d178aae_ff78_412a_830f_3be325d22773">
            <math xmlns="http://www.w3.org/1998/Math/MathML">
              <cn type="integer"> 1 </cn>
            </math>
          </eventAssignment>
        </listOfEventAssignments>
      </event>
      <event id="mwb1346921_99eb_4109_a950_7b39c83ef31f"
name="event_35">
        <trigger>
          <math xmlns="http://www.w3.org/1998/Math/MathML">
            <apply>
              <geq/>
              <csymbol encoding="text"
definitionURL="http://www.sbml.org/sbml/symbols/time"> time </csymbol>
              <cn> 41.57 </cn>
            </apply>
          </math>
        </trigger>
        <listOfEventAssignments>
          <eventAssignment
variable="mw7d178aae_ff78_412a_830f_3be325d22773">
            <math xmlns="http://www.w3.org/1998/Math/MathML">
              <cn type="integer"> 0 </cn>
            </math>
          </eventAssignment>
        </listOfEventAssignments>
      </event>
      <event id="mw859db1a5_552a_45ec_b62d_57ed877b91ee"
name="event_36">
        <trigger>
          <math xmlns="http://www.w3.org/1998/Math/MathML">
```

```
            <apply>
              <geq/>
              <csymbol encoding="text"
definitionURL="http://www.sbml.org/sbml/symbols/time"> time </csymbol>
              <cn type="integer"> 43 </cn>
            </apply>
          </math>
        </trigger>
        <listOfEventAssignments>
          <eventAssignment
variable="mw7d178aae_ff78_412a_830f_3be325d22773">
            <math xmlns="http://www.w3.org/1998/Math/MathML">
              <cn type="integer"> 1 </cn>
            </math>
          </eventAssignment>
        </listOfEventAssignments>
      </event>
      <event id="mw8ef75b4f_3d15_40ca_aed8_862ee8ca24af"
name="event_37">
        <trigger>
          <math xmlns="http://www.w3.org/1998/Math/MathML">
            <apply>
              <geq/>
              <csymbol encoding="text"
definitionURL="http://www.sbml.org/sbml/symbols/time"> time </csymbol>
              <cn> 45.87 </cn>
            </apply>
          </math>
        </trigger>
        <listOfEventAssignments>
          <eventAssignment
variable="mw7d178aae_ff78_412a_830f_3be325d22773">
            <math xmlns="http://www.w3.org/1998/Math/MathML">
              <cn type="integer"> 0 </cn>
            </math>
          </eventAssignment>
        </listOfEventAssignments>
      </event>
      <event id="mw7fa43154_b9ea_4dc8_8248_d7f682171434"
name="event_38">
        <trigger>
          <math xmlns="http://www.w3.org/1998/Math/MathML">
            <apply>
              <geq/>
              <csymbol encoding="text"
definitionURL="http://www.sbml.org/sbml/symbols/time"> time </csymbol>
              <cn> 47.3 </cn>
            </apply>
          </math>
        </trigger>
        <listOfEventAssignments>
```

```
          <eventAssignment
variable="mw7d178aae_ff78_412a_830f_3be325d22773">
            <math xmlns="http://www.w3.org/1998/Math/MathML">
              <cn type="integer"> 1 </cn>
            </math>
          </eventAssignment>
        </listOfEventAssignments>
      </event>
      <event id="mw43cc49b8_908e_40aa_afd7_71972477dd93"
name="event_39">
        <trigger>
          <math xmlns="http://www.w3.org/1998/Math/MathML">
            <apply>
              <geq/>
              <csymbol encoding="text"
definitionURL="http://www.sbml.org/sbml/symbols/time"> time </csymbol>
              <cn> 50.17 </cn>
            </apply>
          </math>
        </trigger>
        <listOfEventAssignments>
          <eventAssignment
variable="mw7d178aae_ff78_412a_830f_3be325d22773">
            <math xmlns="http://www.w3.org/1998/Math/MathML">
              <cn type="integer"> 0 </cn>
            </math>
          </eventAssignment>
        </listOfEventAssignments>
      </event>
      <event id="mwefc87460_c196_4629_a0dc_4cc62941e5c3"
name="event_40">
        <trigger>
          <math xmlns="http://www.w3.org/1998/Math/MathML">
            <apply>
              <geq/>
              <csymbol encoding="text"
definitionURL="http://www.sbml.org/sbml/symbols/time"> time </csymbol>
              <cn> 51.6 </cn>
            </apply>
          </math>
        </trigger>
        <listOfEventAssignments>
          <eventAssignment
variable="mw7d178aae_ff78_412a_830f_3be325d22773">
            <math xmlns="http://www.w3.org/1998/Math/MathML">
              <cn type="integer"> 1 </cn>
            </math>
          </eventAssignment>
        </listOfEventAssignments>
      </event>
      <event id="mw5fc46bb4_ae5f_4f3c_8978_34c861a233fa"
name="event_41">
```

```
        <trigger>
          <math xmlns="http://www.w3.org/1998/Math/MathML">
            <apply>
              <geq/>
              <csymbol encoding="text"
definitionURL="http://www.sbml.org/sbml/symbols/time"> time </csymbol>
              <cn> 54.47 </cn>
            </apply>
          </math>
        </trigger>
        <listOfEventAssignments>
          <eventAssignment
variable="mw7d178aae_ff78_412a_830f_3be325d22773">
            <math xmlns="http://www.w3.org/1998/Math/MathML">
              <cn type="integer"> 0 </cn>
            </math>
          </eventAssignment>
        </listOfEventAssignments>
      </event>
      <event id="mw87d540c8_a2b8_44c6_a33a_25aeca002df4"
name="event_42">
        <trigger>
          <math xmlns="http://www.w3.org/1998/Math/MathML">
            <apply>
              <geq/>
              <csymbol encoding="text"
definitionURL="http://www.sbml.org/sbml/symbols/time"> time </csymbol>
              <cn> 55.9 </cn>
            </apply>
          </math>
        </trigger>
        <listOfEventAssignments>
          <eventAssignment
variable="mw7d178aae_ff78_412a_830f_3be325d22773">
            <math xmlns="http://www.w3.org/1998/Math/MathML">
              <cn type="integer"> 1 </cn>
            </math>
          </eventAssignment>
        </listOfEventAssignments>
      </event>
      <event id="mw98fcc0e9_a7bb_46bf_8063_836e5eb3a483"
name="event_43">
        <trigger>
          <math xmlns="http://www.w3.org/1998/Math/MathML">
            <apply>
              <geq/>
              <csymbol encoding="text"
definitionURL="http://www.sbml.org/sbml/symbols/time"> time </csymbol>
              <cn> 58.77 </cn>
            </apply>
          </math>
        </trigger>
```

```
        <listOfEventAssignments>
          <eventAssignment
variable="mw7d178aae_ff78_412a_830f_3be325d22773">
            <math xmlns="http://www.w3.org/1998/Math/MathML">
              <cn type="integer"> 0 </cn>
            </math>
          </eventAssignment>
        </listOfEventAssignments>
      </event>
      <event id="mw6212a120_eec3_45dd_8497_e70f2f59b9dd"
name="event_44">
        <trigger>
          <math xmlns="http://www.w3.org/1998/Math/MathML">
            <apply>
              <geq/>
              <csymbol encoding="text"
definitionURL="http://www.sbml.org/sbml/symbols/time"> time </csymbol>
              <cn> 60.2 </cn>
            </apply>
          </math>
        </trigger>
        <listOfEventAssignments>
          <eventAssignment
variable="mw7d178aae_ff78_412a_830f_3be325d22773">
            <math xmlns="http://www.w3.org/1998/Math/MathML">
              <cn type="integer"> 1 </cn>
            </math>
          </eventAssignment>
        </listOfEventAssignments>
      </event>
      <event id="mwd8d8dad2_852a_4236_8958_6da25106faaf"
name="event_45">
        <trigger>
          <math xmlns="http://www.w3.org/1998/Math/MathML">
            <apply>
              <geq/>
              <csymbol encoding="text"
definitionURL="http://www.sbml.org/sbml/symbols/time"> time </csymbol>
              <cn> 63.07 </cn>
            </apply>
          </math>
        </trigger>
        <listOfEventAssignments>
          <eventAssignment
variable="mw7d178aae_ff78_412a_830f_3be325d22773">
            <math xmlns="http://www.w3.org/1998/Math/MathML">
              <cn type="integer"> 0 </cn>
            </math>
          </eventAssignment>
        </listOfEventAssignments>
      </event>
```

```
      <event id="mw8a6e41e2_98ab_4037_b137_a19d540be9a9"
name="event_46">
        &lt;trigger&gt;
          &lt;math xmlns="http://www.w3.org/1998/Math/MathML"&gt;
            &lt;apply&gt;
              &lt;geq/&gt;
              <csymbol encoding="text"
definitionURL="http://www.sbml.org/sbml/symbols/time"> time &lt;/csymbol&gt;
              &lt;cn&gt; 64.5 &lt;/cn&gt;
            &lt;/apply&gt;
          &lt;/math&gt;
        &lt;/trigger&gt;
        &lt;listOfEventAssignments&gt;
          <eventAssignment
variable="mw7d178aae_ff78_412a_830f_3be325d22773">
            &lt;math xmlns="http://www.w3.org/1998/Math/MathML"&gt;
              &lt;cn type="integer"&gt; 1 &lt;/cn&gt;
            &lt;/math&gt;
          &lt;/eventAssignment&gt;
        &lt;/listOfEventAssignments&gt;
      &lt;/event&gt;
      <event id="mw931c269d_52be_4d4a_bdd7_236f73139791"
name="event_47">
        &lt;trigger&gt;
          &lt;math xmlns="http://www.w3.org/1998/Math/MathML"&gt;
            &lt;apply&gt;
              &lt;geq/&gt;
              <csymbol encoding="text"
definitionURL="http://www.sbml.org/sbml/symbols/time"> time &lt;/csymbol&gt;
              &lt;cn&gt; 67.37 &lt;/cn&gt;
            &lt;/apply&gt;
          &lt;/math&gt;
        &lt;/trigger&gt;
        &lt;listOfEventAssignments&gt;
          <eventAssignment
variable="mw7d178aae_ff78_412a_830f_3be325d22773">
            &lt;math xmlns="http://www.w3.org/1998/Math/MathML"&gt;
              &lt;cn type="integer"&gt; 0 &lt;/cn&gt;
            &lt;/math&gt;
          &lt;/eventAssignment&gt;
        &lt;/listOfEventAssignments&gt;
      &lt;/event&gt;
      <event id="mwd3e289a3_23a6_4d54_a75f_fdde53da6a81"
name="event_48">
        &lt;trigger&gt;
          &lt;math xmlns="http://www.w3.org/1998/Math/MathML"&gt;
            &lt;apply&gt;
              &lt;geq/&gt;
              <csymbol encoding="text"
definitionURL="http://www.sbml.org/sbml/symbols/time"> time &lt;/csymbol&gt;
              &lt;cn&gt; 68.8 &lt;/cn&gt;
            &lt;/apply&gt;
```

```
          </math>
        </trigger>
        <listOfEventAssignments>
          <eventAssignment
variable="mw7d178aae_ff78_412a_830f_3be325d22773">
            <math xmlns="http://www.w3.org/1998/Math/MathML">
              <cn type="integer"> 1 </cn>
            </math>
          </eventAssignment>
        </listOfEventAssignments>
      </event>
      <event id="mw1636656d_fc0b_43d9_8ebe_3e5dd1eaf57c"
name="event_49">
        <trigger>
          <math xmlns="http://www.w3.org/1998/Math/MathML">
            <apply>
              <geq/>
              <csymbol encoding="text"
definitionURL="http://www.sbml.org/sbml/symbols/time"> time </csymbol>
              <cn> 37.27 </cn>
            </apply>
          </math>
        </trigger>
        <listOfEventAssignments>
          <eventAssignment
variable="mwcedbe49e_2d28_4720_8fcd_207db64228cf">
            <math xmlns="http://www.w3.org/1998/Math/MathML">
              <cn type="integer"> 1 </cn>
            </math>
          </eventAssignment>
        </listOfEventAssignments>
      </event>
      <event id="mw27869041_8050_46fb_8048_fc5584f71b6d"
name="event_50">
        <trigger>
          <math xmlns="http://www.w3.org/1998/Math/MathML">
            <apply>
              <geq/>
              <csymbol encoding="text"
definitionURL="http://www.sbml.org/sbml/symbols/time"> time </csymbol>
              <cn> 38.7 </cn>
            </apply>
          </math>
        </trigger>
        <listOfEventAssignments>
          <eventAssignment
variable="mwcedbe49e_2d28_4720_8fcd_207db64228cf">
            <math xmlns="http://www.w3.org/1998/Math/MathML">
              <cn type="integer"> 0 </cn>
            </math>
          </eventAssignment>
        </listOfEventAssignments>
```

```
      </event>
      <event id="mw63e4dd89_6abb_445d_9d1d_58069d680adb"
name="event_51">
        <trigger>
          <math xmlns="http://www.w3.org/1998/Math/MathML">
            <apply>
              <geq/>
              <csymbol encoding="text"
definitionURL="http://www.sbml.org/sbml/symbols/time"> time </csymbol>
              <cn> 41.57 </cn>
            </apply>
          </math>
        </trigger>
        <listOfEventAssignments>
          <eventAssignment
variable="mwcedbe49e_2d28_4720_8fcd_207db64228cf">
            <math xmlns="http://www.w3.org/1998/Math/MathML">
              <cn type="integer"> 1 </cn>
            </math>
          </eventAssignment>
        </listOfEventAssignments>
      </event>
      <event id="mwfb364b72_bc7f_407d_ba92_67194812f353"
name="event_52">
        <trigger>
          <math xmlns="http://www.w3.org/1998/Math/MathML">
            <apply>
              <geq/>
              <csymbol encoding="text"
definitionURL="http://www.sbml.org/sbml/symbols/time"> time </csymbol>
              <cn type="integer"> 43 </cn>
            </apply>
          </math>
        </trigger>
        <listOfEventAssignments>
          <eventAssignment
variable="mwcedbe49e_2d28_4720_8fcd_207db64228cf">
            <math xmlns="http://www.w3.org/1998/Math/MathML">
              <cn type="integer"> 0 </cn>
            </math>
          </eventAssignment>
        </listOfEventAssignments>
      </event>
      <event id="mw3380b0a5_a3fb_4ba4_aa44_155e6e493222"
name="event_53">
        <trigger>
          <math xmlns="http://www.w3.org/1998/Math/MathML">
            <apply>
              <geq/>
              <csymbol encoding="text"
definitionURL="http://www.sbml.org/sbml/symbols/time"> time </csymbol>
              <cn> 45.87 </cn>
```

```
            &lt;/apply&gt;
          &lt;/math&gt;
        &lt;/trigger&gt;
        &lt;listOfEventAssignments&gt;
          <eventAssignment
variable="mwcedbe49e_2d28_4720_8fcd_207db64228cf">
            &lt;math xmlns="http://www.w3.org/1998/Math/MathML"&gt;
              &lt;cn type="integer"&gt; 1 &lt;/cn&gt;
            &lt;/math&gt;
          &lt;/eventAssignment&gt;
        &lt;/listOfEventAssignments&gt;
      &lt;/event&gt;
      <event id="mw160929ea_71c6_4bd0_ba84_e2c94aaa2118"
name="event_54">
        &lt;trigger&gt;
          &lt;math xmlns="http://www.w3.org/1998/Math/MathML"&gt;
            &lt;apply&gt;
              &lt;geq/&gt;
              <csymbol encoding="text"
definitionURL="http://www.sbml.org/sbml/symbols/time"> time &lt;/csymbol&gt;
              &lt;cn&gt; 47.3 &lt;/cn&gt;
            &lt;/apply&gt;
          &lt;/math&gt;
        &lt;/trigger&gt;
        &lt;listOfEventAssignments&gt;
          <eventAssignment
variable="mwcedbe49e_2d28_4720_8fcd_207db64228cf">
            &lt;math xmlns="http://www.w3.org/1998/Math/MathML"&gt;
              &lt;cn type="integer"&gt; 0 &lt;/cn&gt;
            &lt;/math&gt;
          &lt;/eventAssignment&gt;
        &lt;/listOfEventAssignments&gt;
      &lt;/event&gt;
      <event id="mwf81eb16e_59e5_4e2d_ada1_ae2e4ac60d6e"
name="event_55">
        &lt;trigger&gt;
          &lt;math xmlns="http://www.w3.org/1998/Math/MathML"&gt;
            &lt;apply&gt;
              &lt;geq/&gt;
              <csymbol encoding="text"
definitionURL="http://www.sbml.org/sbml/symbols/time"> time &lt;/csymbol&gt;
              &lt;cn&gt; 50.17 &lt;/cn&gt;
            &lt;/apply&gt;
          &lt;/math&gt;
        &lt;/trigger&gt;
        &lt;listOfEventAssignments&gt;
          <eventAssignment
variable="mwcedbe49e_2d28_4720_8fcd_207db64228cf">
            &lt;math xmlns="http://www.w3.org/1998/Math/MathML"&gt;
              &lt;cn type="integer"&gt; 1 &lt;/cn&gt;
            &lt;/math&gt;
          &lt;/eventAssignment&gt;
```

```
        </listOfEventAssignments>
      </event>
      <event id="mw0b54adc1_5716_478f_9feb_cbf436939be4"
name="event_56">
        <trigger>
          <math xmlns="http://www.w3.org/1998/Math/MathML">
            <apply>
              <geq/>
              <csymbol encoding="text"
definitionURL="http://www.sbml.org/sbml/symbols/time"> time </csymbol>
              <cn> 51.6 </cn>
            </apply>
          </math>
        </trigger>
        <listOfEventAssignments>
          <eventAssignment
variable="mwcedbe49e_2d28_4720_8fcd_207db64228cf">
            <math xmlns="http://www.w3.org/1998/Math/MathML">
              <cn type="integer"> 0 </cn>
            </math>
          </eventAssignment>
        </listOfEventAssignments>
      </event>
      <event id="mwc7b7c1b7_8af8_4138_8182_d52e0372cbc1"
name="event_57">
        <trigger>
          <math xmlns="http://www.w3.org/1998/Math/MathML">
            <apply>
              <geq/>
              <csymbol encoding="text"
definitionURL="http://www.sbml.org/sbml/symbols/time"> time </csymbol>
              <cn> 54.47 </cn>
            </apply>
          </math>
        </trigger>
        <listOfEventAssignments>
          <eventAssignment
variable="mwcedbe49e_2d28_4720_8fcd_207db64228cf">
            <math xmlns="http://www.w3.org/1998/Math/MathML">
              <cn type="integer"> 1 </cn>
            </math>
          </eventAssignment>
        </listOfEventAssignments>
      </event>
      <event id="mw3be62c7f_90e3_4241_ae04_587e37f1bd8d"
name="event_58">
        <trigger>
          <math xmlns="http://www.w3.org/1998/Math/MathML">
            <apply>
              <geq/>
              <csymbol encoding="text"
definitionURL="http://www.sbml.org/sbml/symbols/time"> time </csymbol>
```

```
              <cn> 55.9 </cn>
            </apply>
          </math>
        </trigger>
        <listOfEventAssignments>
          <eventAssignment
variable="mwcedbe49e_2d28_4720_8fcd_207db64228cf">
            <math xmlns="http://www.w3.org/1998/Math/MathML">
              <cn type="integer"> 0 </cn>
            </math>
          </eventAssignment>
        </listOfEventAssignments>
      </event>
      <event id="mw61cb932b_5c83_4c7b_91f2_75fda88765e9"
name="event_59">
        <trigger>
          <math xmlns="http://www.w3.org/1998/Math/MathML">
            <apply>
              <geq/>
              <csymbol encoding="text"
definitionURL="http://www.sbml.org/sbml/symbols/time"> time </csymbol>
              <cn> 58.77 </cn>
            </apply>
          </math>
        </trigger>
        <listOfEventAssignments>
          <eventAssignment
variable="mwcedbe49e_2d28_4720_8fcd_207db64228cf">
            <math xmlns="http://www.w3.org/1998/Math/MathML">
              <cn type="integer"> 1 </cn>
            </math>
          </eventAssignment>
        </listOfEventAssignments>
      </event>
      <event id="mw26370382_d39d_4bd4_8c0a_3b363029fffc"
name="event_60">
        <trigger>
          <math xmlns="http://www.w3.org/1998/Math/MathML">
            <apply>
              <geq/>
              <csymbol encoding="text"
definitionURL="http://www.sbml.org/sbml/symbols/time"> time </csymbol>
              <cn> 60.2 </cn>
            </apply>
          </math>
        </trigger>
        <listOfEventAssignments>
          <eventAssignment
variable="mwcedbe49e_2d28_4720_8fcd_207db64228cf">
            <math xmlns="http://www.w3.org/1998/Math/MathML">
              <cn type="integer"> 0 </cn>
            </math>
```

```
          </eventAssignment>
        </listOfEventAssignments>
      </event>
      <event id="mwe3a64046_1e09_4f8f_b0ac_2583dc8e17e4"
name="event_61">
        <trigger>
          <math xmlns="http://www.w3.org/1998/Math/MathML">
            <apply>
              <geq/>
              <csymbol encoding="text"
definitionURL="http://www.sbml.org/sbml/symbols/time"> time </csymbol>
              <cn> 63.07 </cn>
            </apply>
          </math>
        </trigger>
        <listOfEventAssignments>
          <eventAssignment
variable="mwcedbe49e_2d28_4720_8fcd_207db64228cf">
            <math xmlns="http://www.w3.org/1998/Math/MathML">
              <cn type="integer"> 1 </cn>
            </math>
          </eventAssignment>
        </listOfEventAssignments>
      </event>
      <event id="mw95f5a8b0_339a_4728_ae43_15d95ef855c9"
name="event_62">
        <trigger>
          <math xmlns="http://www.w3.org/1998/Math/MathML">
            <apply>
              <geq/>
              <csymbol encoding="text"
definitionURL="http://www.sbml.org/sbml/symbols/time"> time </csymbol>
              <cn> 64.5 </cn>
            </apply>
          </math>
        </trigger>
        <listOfEventAssignments>
          <eventAssignment
variable="mwcedbe49e_2d28_4720_8fcd_207db64228cf">
            <math xmlns="http://www.w3.org/1998/Math/MathML">
              <cn type="integer"> 0 </cn>
            </math>
          </eventAssignment>
        </listOfEventAssignments>
      </event>
      <event id="mw4f8a03e9_c6fa_40b3_859b_12193d8562c3"
name="event_63">
        <trigger>
          <math xmlns="http://www.w3.org/1998/Math/MathML">
            <apply>
              <geq/>
```

```
              <csymbol encoding="text"
definitionURL="http://www.sbml.org/sbml/symbols/time"> time </csymbol>
              <cn> 67.37 </cn>
            </apply>
          </math>
        </trigger>
        <listOfEventAssignments>
          <eventAssignment
variable="mwcedbe49e_2d28_4720_8fcd_207db64228cf">
            <math xmlns="http://www.w3.org/1998/Math/MathML">
              <cn type="integer"> 1 </cn>
            </math>
          </eventAssignment>
        </listOfEventAssignments>
      </event>
      <event id="mw424d7e03_142f_469b_ad4f_a50113f2113e"
name="event_64">
        <trigger>
          <math xmlns="http://www.w3.org/1998/Math/MathML">
            <apply>
              <geq/>
              <csymbol encoding="text"
definitionURL="http://www.sbml.org/sbml/symbols/time"> time </csymbol>
              <cn> 68.8 </cn>
            </apply>
          </math>
        </trigger>
        <listOfEventAssignments>
          <eventAssignment
variable="mwcedbe49e_2d28_4720_8fcd_207db64228cf">
            <math xmlns="http://www.w3.org/1998/Math/MathML">
              <cn type="integer"> 0 </cn>
            </math>
          </eventAssignment>
        </listOfEventAssignments>
      </event>
      <event id="mw24037ab3_4411_416e_87e6_d858b261c962"
name="event_65">
        <trigger>
          <math xmlns="http://www.w3.org/1998/Math/MathML">
            <apply>
              <geq/>
              <csymbol encoding="text"
definitionURL="http://www.sbml.org/sbml/symbols/time"> time </csymbol>
              <cn> 71.67 </cn>
            </apply>
          </math>
        </trigger>
        <listOfEventAssignments>
          <eventAssignment
variable="mwcedbe49e_2d28_4720_8fcd_207db64228cf">
            <math xmlns="http://www.w3.org/1998/Math/MathML">
```

```
              <cn type="integer"> 1 </cn>
            </math>
          </eventAssignment>
        </listOfEventAssignments>
      </event>
      <event id="mw7734f2cc_94b1_440a_a42d_e99970bc1d2a"
name="event_66">
        <trigger>
          <math xmlns="http://www.w3.org/1998/Math/MathML">
            <apply>
              <geq/>
              <csymbol encoding="text"
definitionURL="http://www.sbml.org/sbml/symbols/time"> time </csymbol>
              <cn> 73.1 </cn>
            </apply>
          </math>
        </trigger>
        <listOfEventAssignments>
          <eventAssignment
variable="mwcedbe49e_2d28_4720_8fcd_207db64228cf">
            <math xmlns="http://www.w3.org/1998/Math/MathML">
              <cn type="integer"> 0 </cn>
            </math>
          </eventAssignment>
        </listOfEventAssignments>
      </event>
      <event id="mw522f1d69_4c72_4862_99da_18eaf7ab4f67"
name="event_67">
        <trigger>
          <math xmlns="http://www.w3.org/1998/Math/MathML">
            <apply>
              <geq/>
              <csymbol encoding="text"
definitionURL="http://www.sbml.org/sbml/symbols/time"> time </csymbol>
              <cn> 75.97 </cn>
            </apply>
          </math>
        </trigger>
        <listOfEventAssignments>
          <eventAssignment
variable="mwcedbe49e_2d28_4720_8fcd_207db64228cf">
            <math xmlns="http://www.w3.org/1998/Math/MathML">
              <cn type="integer"> 1 </cn>
            </math>
          </eventAssignment>
        </listOfEventAssignments>
      </event>
      <event id="mw39345d8f_5583_4097_bc94_35ae9c9a53ad"
name="event_68">
        <trigger>
          <math xmlns="http://www.w3.org/1998/Math/MathML">
            <apply>
```

```
              &lt;geq/&gt;
              <csymbol encoding="text"
definitionURL="http://www.sbml.org/sbml/symbols/time"> time &lt;/csymbol&gt;
              &lt;cn&gt; 77.4 &lt;/cn&gt;
            &lt;/apply&gt;
          &lt;/math&gt;
        &lt;/trigger&gt;
        &lt;listOfEventAssignments&gt;
          <eventAssignment
variable="mwcedbe49e_2d28_4720_8fcd_207db64228cf">
            &lt;math xmlns="http://www.w3.org/1998/Math/MathML"&gt;
              &lt;cn type="integer"&gt; 0 &lt;/cn&gt;
            &lt;/math&gt;
          &lt;/eventAssignment&gt;
        &lt;/listOfEventAssignments&gt;
      &lt;/event&gt;
      <event id="mw64ec5954_ea08_49b5_af40_acf28be406fe"
name="event_69">
        &lt;trigger&gt;
          &lt;math xmlns="http://www.w3.org/1998/Math/MathML"&gt;
            &lt;apply&gt;
              &lt;geq/&gt;
              <csymbol encoding="text"
definitionURL="http://www.sbml.org/sbml/symbols/time"> time &lt;/csymbol&gt;
              &lt;cn&gt; 80.27 &lt;/cn&gt;
            &lt;/apply&gt;
          &lt;/math&gt;
        &lt;/trigger&gt;
        &lt;listOfEventAssignments&gt;
          <eventAssignment
variable="mwcedbe49e_2d28_4720_8fcd_207db64228cf">
            &lt;math xmlns="http://www.w3.org/1998/Math/MathML"&gt;
              &lt;cn type="integer"&gt; 1 &lt;/cn&gt;
            &lt;/math&gt;
          &lt;/eventAssignment&gt;
        &lt;/listOfEventAssignments&gt;
      &lt;/event&gt;
      <event id="mw9062fde8_a60f_4b6b_b584_9009374e14d9"
name="event_70">
        &lt;trigger&gt;
          &lt;math xmlns="http://www.w3.org/1998/Math/MathML"&gt;
            &lt;apply&gt;
              &lt;geq/&gt;
              <csymbol encoding="text"
definitionURL="http://www.sbml.org/sbml/symbols/time"> time &lt;/csymbol&gt;
              &lt;cn&gt; 81.7 &lt;/cn&gt;
            &lt;/apply&gt;
          &lt;/math&gt;
        &lt;/trigger&gt;
        &lt;listOfEventAssignments&gt;
          <eventAssignment
variable="mwcedbe49e_2d28_4720_8fcd_207db64228cf">
```

```
            <math xmlns="http://www.w3.org/1998/Math/MathML">
              <cn type="integer"> 0 </cn>
            </math>
          </eventAssignment>
        </listOfEventAssignments>
      </event>
      <event id="mw132c4d28_3702_416d_bc7e_aaabe3454d31"
name="event_71">
        <trigger>
          <math xmlns="http://www.w3.org/1998/Math/MathML">
            <apply>
              <geq/>
              <csymbol encoding="text"
definitionURL="http://www.sbml.org/sbml/symbols/time"> time </csymbol>
              <cn> 84.57 </cn>
            </apply>
          </math>
        </trigger>
        <listOfEventAssignments>
          <eventAssignment
variable="mwcedbe49e_2d28_4720_8fcd_207db64228cf">
            <math xmlns="http://www.w3.org/1998/Math/MathML">
              <cn type="integer"> 1 </cn>
            </math>
          </eventAssignment>
        </listOfEventAssignments>
      </event>
      <event id="mw4f2276f5_a49e_4e3d_acb5_ef75085a7f98"
name="event_72">
        <trigger>
          <math xmlns="http://www.w3.org/1998/Math/MathML">
            <apply>
              <geq/>
              <csymbol encoding="text"
definitionURL="http://www.sbml.org/sbml/symbols/time"> time </csymbol>
              <cn type="integer"> 86 </cn>
            </apply>
          </math>
        </trigger>
        <listOfEventAssignments>
          <eventAssignment
variable="mwcedbe49e_2d28_4720_8fcd_207db64228cf">
            <math xmlns="http://www.w3.org/1998/Math/MathML">
              <cn type="integer"> 0 </cn>
            </math>
          </eventAssignment>
        </listOfEventAssignments>
      </event>
      <event id="mw8b50ab2e_1f59_405f_946b_0ff09e79a899"
name="event_73">
        <trigger>
          <math xmlns="http://www.w3.org/1998/Math/MathML">
```

```
            &lt;apply&gt;
              &lt;geq/&gt;
              <csymbol encoding="text"
definitionURL="http://www.sbml.org/sbml/symbols/time"> time &lt;/csymbol&gt;
              &lt;cn&gt; 88.87 &lt;/cn&gt;
            &lt;/apply&gt;
          &lt;/math&gt;
        &lt;/trigger&gt;
        &lt;listOfEventAssignments&gt;
          <eventAssignment
variable="mwcedbe49e_2d28_4720_8fcd_207db64228cf">
            &lt;math xmlns="http://www.w3.org/1998/Math/MathML"&gt;
              &lt;cn type="integer"&gt; 1 &lt;/cn&gt;
            &lt;/math&gt;
          &lt;/eventAssignment&gt;
        &lt;/listOfEventAssignments&gt;
      &lt;/event&gt;
      <event id="mwddc4521a_4601_40fd_998d_5da50f458be1"
name="event_74">
        &lt;trigger&gt;
          &lt;math xmlns="http://www.w3.org/1998/Math/MathML"&gt;
            &lt;apply&gt;
              &lt;geq/&gt;
              <csymbol encoding="text"
definitionURL="http://www.sbml.org/sbml/symbols/time"> time &lt;/csymbol&gt;
              &lt;cn&gt; 90.3 &lt;/cn&gt;
            &lt;/apply&gt;
          &lt;/math&gt;
        &lt;/trigger&gt;
        &lt;listOfEventAssignments&gt;
          <eventAssignment
variable="mwcedbe49e_2d28_4720_8fcd_207db64228cf">
            &lt;math xmlns="http://www.w3.org/1998/Math/MathML"&gt;
              &lt;cn type="integer"&gt; 0 &lt;/cn&gt;
            &lt;/math&gt;
          &lt;/eventAssignment&gt;
        &lt;/listOfEventAssignments&gt;
      &lt;/event&gt;
      <event id="mw213270ba_a06c_43af_a9ba_0ab83684d1fc"
name="event_75">
        &lt;trigger&gt;
          &lt;math xmlns="http://www.w3.org/1998/Math/MathML"&gt;
            &lt;apply&gt;
              &lt;geq/&gt;
              <csymbol encoding="text"
definitionURL="http://www.sbml.org/sbml/symbols/time"> time &lt;/csymbol&gt;
              &lt;cn&gt; 71.67 &lt;/cn&gt;
            &lt;/apply&gt;
          &lt;/math&gt;
        &lt;/trigger&gt;
        &lt;listOfEventAssignments&gt;
```

```
          <eventAssignment
variable="mw7d178aae_ff78_412a_830f_3be325d22773">
            <math xmlns="http://www.w3.org/1998/Math/MathML">
              <cn type="integer"> 0 </cn>
            </math>
          </eventAssignment>
        </listOfEventAssignments>
      </event>
      <event id="mwfbf20cfc_bd1a_4315_8f69_3e4c14b35916"
name="event_76">
        <trigger>
          <math xmlns="http://www.w3.org/1998/Math/MathML">
            <apply>
              <geq/>
              <csymbol encoding="text"
definitionURL="http://www.sbml.org/sbml/symbols/time"> time </csymbol>
              <cn> 73.1 </cn>
            </apply>
          </math>
        </trigger>
        <listOfEventAssignments>
          <eventAssignment
variable="mw7d178aae_ff78_412a_830f_3be325d22773">
            <math xmlns="http://www.w3.org/1998/Math/MathML">
              <cn type="integer"> 1 </cn>
            </math>
          </eventAssignment>
        </listOfEventAssignments>
      </event>
      <event id="mw3ca95e16_f7b2_41a3_9477_1c6e0b6f8088"
name="event_77">
        <trigger>
          <math xmlns="http://www.w3.org/1998/Math/MathML">
            <apply>
              <geq/>
              <csymbol encoding="text"
definitionURL="http://www.sbml.org/sbml/symbols/time"> time </csymbol>
              <cn> 75.97 </cn>
            </apply>
          </math>
        </trigger>
        <listOfEventAssignments>
          <eventAssignment
variable="mw7d178aae_ff78_412a_830f_3be325d22773">
            <math xmlns="http://www.w3.org/1998/Math/MathML">
              <cn type="integer"> 0 </cn>
            </math>
          </eventAssignment>
        </listOfEventAssignments>
      </event>
      <event id="mw8fc96fee_4a19_406f_997f_021cba4790f5"
name="event_78">
```

```
        <trigger>
          <math xmlns="http://www.w3.org/1998/Math/MathML">
            <apply>
              <geq/>
              <csymbol encoding="text"
definitionURL="http://www.sbml.org/sbml/symbols/time"> time </csymbol>
              <cn> 77.4 </cn>
            </apply>
          </math>
        </trigger>
        <listOfEventAssignments>
          <eventAssignment
variable="mw7d178aae_ff78_412a_830f_3be325d22773">
            <math xmlns="http://www.w3.org/1998/Math/MathML">
              <cn type="integer"> 1 </cn>
            </math>
          </eventAssignment>
        </listOfEventAssignments>
      </event>
      <event id="mw17f30831_2274_41c0_87ef_0e0e76f49775"
name="event_79">
        <trigger>
          <math xmlns="http://www.w3.org/1998/Math/MathML">
            <apply>
              <geq/>
              <csymbol encoding="text"
definitionURL="http://www.sbml.org/sbml/symbols/time"> time </csymbol>
              <cn> 80.27 </cn>
            </apply>
          </math>
        </trigger>
        <listOfEventAssignments>
          <eventAssignment
variable="mw7d178aae_ff78_412a_830f_3be325d22773">
            <math xmlns="http://www.w3.org/1998/Math/MathML">
              <cn type="integer"> 0 </cn>
            </math>
          </eventAssignment>
        </listOfEventAssignments>
      </event>
      <event id="mw4d52cac2_772f_465e_8c5d_7669eac1a80f"
name="event_80">
        <trigger>
          <math xmlns="http://www.w3.org/1998/Math/MathML">
            <apply>
              <geq/>
              <csymbol encoding="text"
definitionURL="http://www.sbml.org/sbml/symbols/time"> time </csymbol>
              <cn> 81.7 </cn>
            </apply>
          </math>
        </trigger>
```

```
        <listOfEventAssignments>
          <eventAssignment
variable="mw7d178aae_ff78_412a_830f_3be325d22773">
            <math xmlns="http://www.w3.org/1998/Math/MathML">
              <cn type="integer"> 1 </cn>
            </math>
          </eventAssignment>
        </listOfEventAssignments>
      </event>
      <event id="mw2e7759fb_84ba_4da7_bddb_85041916042f"
name="event_81">
        <trigger>
          <math xmlns="http://www.w3.org/1998/Math/MathML">
            <apply>
              <geq/>
              <csymbol encoding="text"
definitionURL="http://www.sbml.org/sbml/symbols/time"> time </csymbol>
              <cn> 84.57 </cn>
            </apply>
          </math>
        </trigger>
        <listOfEventAssignments>
          <eventAssignment
variable="mw7d178aae_ff78_412a_830f_3be325d22773">
            <math xmlns="http://www.w3.org/1998/Math/MathML">
              <cn type="integer"> 0 </cn>
            </math>
          </eventAssignment>
        </listOfEventAssignments>
      </event>
      <event id="mw1479edc7_4504_43b1_9b27_9333af269527"
name="event_82">
        <trigger>
          <math xmlns="http://www.w3.org/1998/Math/MathML">
            <apply>
              <geq/>
              <csymbol encoding="text"
definitionURL="http://www.sbml.org/sbml/symbols/time"> time </csymbol>
              <cn type="integer"> 86 </cn>
            </apply>
          </math>
        </trigger>
        <listOfEventAssignments>
          <eventAssignment
variable="mw7d178aae_ff78_412a_830f_3be325d22773">
            <math xmlns="http://www.w3.org/1998/Math/MathML">
              <cn type="integer"> 1 </cn>
            </math>
          </eventAssignment>
        </listOfEventAssignments>
      </event>
```

```
      <event id="mwaabc15c1_3924_409a_bd92_9e82ff6a63f6"
name="event_83">
        <trigger>
          <math xmlns="http://www.w3.org/1998/Math/MathML">
            <apply>
              <geq/>
              <csymbol encoding="text"
definitionURL="http://www.sbml.org/sbml/symbols/time"> time </csymbol>
              <cn> 88.87 </cn>
            </apply>
          </math>
        </trigger>
        <listOfEventAssignments>
          <eventAssignment
variable="mw7d178aae_ff78_412a_830f_3be325d22773">
            <math xmlns="http://www.w3.org/1998/Math/MathML">
              <cn type="integer"> 0 </cn>
            </math>
          </eventAssignment>
        </listOfEventAssignments>
      </event>
      <event id="mw60e354ac_86b3_4f92_9d20_69dcce6253ef"
name="event_84">
        <trigger>
          <math xmlns="http://www.w3.org/1998/Math/MathML">
            <apply>
              <geq/>
              <csymbol encoding="text"
definitionURL="http://www.sbml.org/sbml/symbols/time"> time </csymbol>
              <cn> 90.3 </cn>
            </apply>
          </math>
        </trigger>
        <listOfEventAssignments>
          <eventAssignment
variable="mw7d178aae_ff78_412a_830f_3be325d22773">
            <math xmlns="http://www.w3.org/1998/Math/MathML">
              <cn type="integer"> 1 </cn>
            </math>
          </eventAssignment>
        </listOfEventAssignments>
      </event>
      <event id="mwb3e7898b_48f5_4fd1_98a0_3cadda5ef384"
name="event_85">
        <trigger>
          <math xmlns="http://www.w3.org/1998/Math/MathML">
            <apply>
              <geq/>
              <csymbol encoding="text"
definitionURL="http://www.sbml.org/sbml/symbols/time"> time </csymbol>
              <cn> 93.17 </cn>
            </apply>
```

```
          </math>
        </trigger>
        <listOfEventAssignments>
          <eventAssignment
variable="mw7d178aae_ff78_412a_830f_3be325d22773">
            <math xmlns="http://www.w3.org/1998/Math/MathML">
              <cn type="integer"> 0 </cn>
            </math>
          </eventAssignment>
        </listOfEventAssignments>
      </event>
      <event id="mw3f8fca35_ebc4_4cb7_adf9_44ed8f86e109"
name="event_86">
        <trigger>
          <math xmlns="http://www.w3.org/1998/Math/MathML">
            <apply>
              <geq/>
              <csymbol encoding="text"
definitionURL="http://www.sbml.org/sbml/symbols/time"> time </csymbol>
              <cn> 94.6 </cn>
            </apply>
          </math>
        </trigger>
        <listOfEventAssignments>
          <eventAssignment
variable="mw7d178aae_ff78_412a_830f_3be325d22773">
            <math xmlns="http://www.w3.org/1998/Math/MathML">
              <cn type="integer"> 1 </cn>
            </math>
          </eventAssignment>
        </listOfEventAssignments>
      </event>
      <event id="mw273be0cf_8833_4930_9e14_bb318e9c8c99"
name="event_87">
        <trigger>
          <math xmlns="http://www.w3.org/1998/Math/MathML">
            <apply>
              <geq/>
              <csymbol encoding="text"
definitionURL="http://www.sbml.org/sbml/symbols/time"> time </csymbol>
              <cn> 97.47 </cn>
            </apply>
          </math>
        </trigger>
        <listOfEventAssignments>
          <eventAssignment
variable="mw7d178aae_ff78_412a_830f_3be325d22773">
            <math xmlns="http://www.w3.org/1998/Math/MathML">
              <cn type="integer"> 0 </cn>
            </math>
          </eventAssignment>
        </listOfEventAssignments>
```

```
      </event>
      <event id="mw5d8dc5c8_213b_41b7_99c9_80a4d98ebba5"
name="event_88">
        <trigger>
          <math xmlns="http://www.w3.org/1998/Math/MathML">
            <apply>
              <geq/>
              <csymbol encoding="text"
definitionURL="http://www.sbml.org/sbml/symbols/time"> time </csymbol>
              <cn> 98.9 </cn>
            </apply>
          </math>
        </trigger>
        <listOfEventAssignments>
          <eventAssignment
variable="mw7d178aae_ff78_412a_830f_3be325d22773">
            <math xmlns="http://www.w3.org/1998/Math/MathML">
              <cn type="integer"> 1 </cn>
            </math>
          </eventAssignment>
        </listOfEventAssignments>
      </event>
      <event id="mw588f5c28_d139_4d6f_9c4b_7e10ab87aa00"
name="event_89">
        <trigger>
          <math xmlns="http://www.w3.org/1998/Math/MathML">
            <apply>
              <geq/>
              <csymbol encoding="text"
definitionURL="http://www.sbml.org/sbml/symbols/time"> time </csymbol>
              <cn> 101.77 </cn>
            </apply>
          </math>
        </trigger>
        <listOfEventAssignments>
          <eventAssignment
variable="mw7d178aae_ff78_412a_830f_3be325d22773">
            <math xmlns="http://www.w3.org/1998/Math/MathML">
              <cn type="integer"> 0 </cn>
            </math>
          </eventAssignment>
        </listOfEventAssignments>
      </event>
      <event id="mw5ffae9d7_7903_414c_93f9_e8c4eb25602f"
name="event_90">
        <trigger>
          <math xmlns="http://www.w3.org/1998/Math/MathML">
            <apply>
              <geq/>
              <csymbol encoding="text"
definitionURL="http://www.sbml.org/sbml/symbols/time"> time </csymbol>
              <cn> 103.2 </cn>
```

```
          &lt;/apply&gt;
        &lt;/math&gt;
      &lt;/trigger&gt;
      &lt;listOfEventAssignments&gt;
        <eventAssignment
variable="mw7d178aae_ff78_412a_830f_3be325d22773">
          &lt;math xmlns="http://www.w3.org/1998/Math/MathML"&gt;
            &lt;cn type="integer"&gt; 1 &lt;/cn&gt;
          &lt;/math&gt;
        &lt;/eventAssignment&gt;
      &lt;/listOfEventAssignments&gt;
    &lt;/event&gt;
    <event id="mw2e538155_678e_4105_ba72_ea62b4b68ed7"
name="event_91">
      &lt;trigger&gt;
        &lt;math xmlns="http://www.w3.org/1998/Math/MathML"&gt;
          &lt;apply&gt;
            &lt;geq/&gt;
            <csymbol encoding="text"
definitionURL="http://www.sbml.org/sbml/symbols/time"> time &lt;/csymbol&gt;
            &lt;cn&gt; 106.07 &lt;/cn&gt;
          &lt;/apply&gt;
        &lt;/math&gt;
      &lt;/trigger&gt;
      &lt;listOfEventAssignments&gt;
        <eventAssignment
variable="mw7d178aae_ff78_412a_830f_3be325d22773">
          &lt;math xmlns="http://www.w3.org/1998/Math/MathML"&gt;
            &lt;cn type="integer"&gt; 0 &lt;/cn&gt;
          &lt;/math&gt;
        &lt;/eventAssignment&gt;
      &lt;/listOfEventAssignments&gt;
    &lt;/event&gt;
    <event id="mw4f348ef3_224e_4633_9acc_56cac1a1e4bf"
name="event_92">
      &lt;trigger&gt;
        &lt;math xmlns="http://www.w3.org/1998/Math/MathML"&gt;
          &lt;apply&gt;
            &lt;geq/&gt;
            <csymbol encoding="text"
definitionURL="http://www.sbml.org/sbml/symbols/time"> time &lt;/csymbol&gt;
            &lt;cn&gt; 107.5 &lt;/cn&gt;
          &lt;/apply&gt;
        &lt;/math&gt;
      &lt;/trigger&gt;
      &lt;listOfEventAssignments&gt;
        <eventAssignment
variable="mw7d178aae_ff78_412a_830f_3be325d22773">
          &lt;math xmlns="http://www.w3.org/1998/Math/MathML"&gt;
            &lt;cn type="integer"&gt; 1 &lt;/cn&gt;
          &lt;/math&gt;
        &lt;/eventAssignment&gt;
```

```
        </listOfEventAssignments>
      </event>
      <event id="mw6dfd0312_055a_419e_ba7b_73ed0f5f3db3"
name="event_93">
        <trigger>
          <math xmlns="http://www.w3.org/1998/Math/MathML">
            <apply>
              <geq/>
              <csymbol encoding="text"
definitionURL="http://www.sbml.org/sbml/symbols/time"> time </csymbol>
              <cn> 110.37 </cn>
            </apply>
          </math>
        </trigger>
        <listOfEventAssignments>
          <eventAssignment
variable="mw7d178aae_ff78_412a_830f_3be325d22773">
            <math xmlns="http://www.w3.org/1998/Math/MathML">
              <cn type="integer"> 0 </cn>
            </math>
          </eventAssignment>
        </listOfEventAssignments>
      </event>
      <event id="mw124a5740_e7f3_40ef_bc05_55bf8f534573"
name="event_94">
        <trigger>
          <math xmlns="http://www.w3.org/1998/Math/MathML">
            <apply>
              <geq/>
              <csymbol encoding="text"
definitionURL="http://www.sbml.org/sbml/symbols/time"> time </csymbol>
              <cn> 111.8 </cn>
            </apply>
          </math>
        </trigger>
        <listOfEventAssignments>
          <eventAssignment
variable="mw7d178aae_ff78_412a_830f_3be325d22773">
            <math xmlns="http://www.w3.org/1998/Math/MathML">
              <cn type="integer"> 1 </cn>
            </math>
          </eventAssignment>
        </listOfEventAssignments>
      </event>
      <event id="mw9d3082c6_9361_4e28_b586_a68ccf164450"
name="event_95">
        <trigger>
          <math xmlns="http://www.w3.org/1998/Math/MathML">
            <apply>
              <geq/>
              <csymbol encoding="text"
definitionURL="http://www.sbml.org/sbml/symbols/time"> time </csymbol>
```

```
              &lt;cn&gt; 93.17 &lt;/cn&gt;
            &lt;/apply&gt;
          &lt;/math&gt;
        &lt;/trigger&gt;
        &lt;listOfEventAssignments&gt;
          <eventAssignment
variable="mwcedbe49e_2d28_4720_8fcd_207db64228cf">
            &lt;math xmlns="http://www.w3.org/1998/Math/MathML"&gt;
              &lt;cn type="integer"&gt; 1 &lt;/cn&gt;
            &lt;/math&gt;
          &lt;/eventAssignment&gt;
        &lt;/listOfEventAssignments&gt;
      &lt;/event&gt;
      <event id="mw1684612f_45e9_4db7_88f8_65ad300eb5b4"
name="event_96">
        &lt;trigger&gt;
          &lt;math xmlns="http://www.w3.org/1998/Math/MathML"&gt;
            &lt;apply&gt;
              &lt;geq/&gt;
              <csymbol encoding="text"
definitionURL="http://www.sbml.org/sbml/symbols/time"> time &lt;/csymbol&gt;
              &lt;cn&gt; 94.6 &lt;/cn&gt;
            &lt;/apply&gt;
          &lt;/math&gt;
        &lt;/trigger&gt;
        &lt;listOfEventAssignments&gt;
          <eventAssignment
variable="mwcedbe49e_2d28_4720_8fcd_207db64228cf">
            &lt;math xmlns="http://www.w3.org/1998/Math/MathML"&gt;
              &lt;cn type="integer"&gt; 0 &lt;/cn&gt;
            &lt;/math&gt;
          &lt;/eventAssignment&gt;
        &lt;/listOfEventAssignments&gt;
      &lt;/event&gt;
      <event id="mw221bea83_2b20_4819_978a_dda54c82970e"
name="event_97">
        &lt;trigger&gt;
          &lt;math xmlns="http://www.w3.org/1998/Math/MathML"&gt;
            &lt;apply&gt;
              &lt;geq/&gt;
              <csymbol encoding="text"
definitionURL="http://www.sbml.org/sbml/symbols/time"> time &lt;/csymbol&gt;
              &lt;cn&gt; 97.47 &lt;/cn&gt;
            &lt;/apply&gt;
          &lt;/math&gt;
        &lt;/trigger&gt;
        &lt;listOfEventAssignments&gt;
          <eventAssignment
variable="mwcedbe49e_2d28_4720_8fcd_207db64228cf">
            &lt;math xmlns="http://www.w3.org/1998/Math/MathML"&gt;
              &lt;cn type="integer"&gt; 1 &lt;/cn&gt;
            &lt;/math&gt;
```

```
          </eventAssignment>
        </listOfEventAssignments>
      </event>
      <event id="mw332c1c50_adae_42b9_b17c_ddec56281ef9"
name="event_98">
        <trigger>
          <math xmlns="http://www.w3.org/1998/Math/MathML">
            <apply>
              <geq/>
              <csymbol encoding="text"
definitionURL="http://www.sbml.org/sbml/symbols/time"> time </csymbol>
              <cn> 98.9 </cn>
            </apply>
          </math>
        </trigger>
        <listOfEventAssignments>
          <eventAssignment
variable="mwcedbe49e_2d28_4720_8fcd_207db64228cf">
            <math xmlns="http://www.w3.org/1998/Math/MathML">
              <cn type="integer"> 0 </cn>
            </math>
          </eventAssignment>
        </listOfEventAssignments>
      </event>
      <event id="mw37746b80_671a_4989_a6fe_aaa6d8d00cf9"
name="event_99">
        <trigger>
          <math xmlns="http://www.w3.org/1998/Math/MathML">
            <apply>
              <geq/>
              <csymbol encoding="text"
definitionURL="http://www.sbml.org/sbml/symbols/time"> time </csymbol>
              <cn> 101.77 </cn>
            </apply>
          </math>
        </trigger>
        <listOfEventAssignments>
          <eventAssignment
variable="mwcedbe49e_2d28_4720_8fcd_207db64228cf">
            <math xmlns="http://www.w3.org/1998/Math/MathML">
              <cn type="integer"> 1 </cn>
            </math>
          </eventAssignment>
        </listOfEventAssignments>
      </event>
      <event id="mw72312340_98a9_4b90_bdbb_36b664a46f8b"
name="event_100">
        <trigger>
          <math xmlns="http://www.w3.org/1998/Math/MathML">
            <apply>
              <geq/>
```

```
              <csymbol encoding="text"
definitionURL="http://www.sbml.org/sbml/symbols/time"> time </csymbol>
              <cn> 103.2 </cn>
            </apply>
          </math>
        </trigger>
        <listOfEventAssignments>
          <eventAssignment
variable="mwcedbe49e_2d28_4720_8fcd_207db64228cf">
            <math xmlns="http://www.w3.org/1998/Math/MathML">
              <cn type="integer"> 0 </cn>
            </math>
          </eventAssignment>
        </listOfEventAssignments>
      </event>
      <event id="mw7e422b0b_0e71_46db_bba0_68a2e1d4973f"
name="event_101">
        <trigger>
          <math xmlns="http://www.w3.org/1998/Math/MathML">
            <apply>
              <geq/>
              <csymbol encoding="text"
definitionURL="http://www.sbml.org/sbml/symbols/time"> time </csymbol>
              <cn> 106.07 </cn>
            </apply>
          </math>
        </trigger>
        <listOfEventAssignments>
          <eventAssignment
variable="mwcedbe49e_2d28_4720_8fcd_207db64228cf">
            <math xmlns="http://www.w3.org/1998/Math/MathML">
              <cn type="integer"> 1 </cn>
            </math>
          </eventAssignment>
        </listOfEventAssignments>
      </event>
      <event id="mw6b6b348b_5711_4296_81a6_70a4194d81d4"
name="event_102">
        <trigger>
          <math xmlns="http://www.w3.org/1998/Math/MathML">
            <apply>
              <geq/>
              <csymbol encoding="text"
definitionURL="http://www.sbml.org/sbml/symbols/time"> time </csymbol>
              <cn> 107.5 </cn>
            </apply>
          </math>
        </trigger>
        <listOfEventAssignments>
          <eventAssignment
variable="mwcedbe49e_2d28_4720_8fcd_207db64228cf">
            <math xmlns="http://www.w3.org/1998/Math/MathML">
```

```
            <cn type="integer"> 0 </cn>
          </math>
        </eventAssignment>
      </listOfEventAssignments>
    </event>
    <event id="mwb98867fa_ba8b_4322_9989_79c555870df5"
name="event_103">
      <trigger>
        <math xmlns="http://www.w3.org/1998/Math/MathML">
          <apply>
            <geq/>
            <csymbol encoding="text"
definitionURL="http://www.sbml.org/sbml/symbols/time"> time </csymbol>
            <cn> 110.37 </cn>
          </apply>
        </math>
      </trigger>
      <listOfEventAssignments>
        <eventAssignment
variable="mwcedbe49e_2d28_4720_8fcd_207db64228cf">
          <math xmlns="http://www.w3.org/1998/Math/MathML">
            <cn type="integer"> 1 </cn>
          </math>
        </eventAssignment>
      </listOfEventAssignments>
    </event>
    <event id="mw9850c0c7_c7c8_4eb1_9aeb_4de205ccb882"
name="event_104">
      <trigger>
        <math xmlns="http://www.w3.org/1998/Math/MathML">
          <apply>
            <geq/>
            <csymbol encoding="text"
definitionURL="http://www.sbml.org/sbml/symbols/time"> time </csymbol>
            <cn> 111.8 </cn>
          </apply>
        </math>
      </trigger>
      <listOfEventAssignments>
        <eventAssignment
variable="mwcedbe49e_2d28_4720_8fcd_207db64228cf">
          <math xmlns="http://www.w3.org/1998/Math/MathML">
            <cn type="integer"> 0 </cn>
          </math>
        </eventAssignment>
      </listOfEventAssignments>
    </event>
    <event id="mw93432ff9_ae9a_41c5_a74c_41ba48ecd721"
name="event_105">
      <trigger>
        <math xmlns="http://www.w3.org/1998/Math/MathML">
          <apply>
```

```
            &lt;geq/&gt;
            <csymbol encoding="text"
definitionURL="http://www.sbml.org/sbml/symbols/time"> time &lt;/csymbol&gt;
            &lt;cn&gt; 114.67 &lt;/cn&gt;
          &lt;/apply&gt;
        &lt;/math&gt;
      &lt;/trigger&gt;
      &lt;listOfEventAssignments&gt;
        <eventAssignment
variable="mwcedbe49e_2d28_4720_8fcd_207db64228cf">
          &lt;math xmlns="http://www.w3.org/1998/Math/MathML"&gt;
            &lt;cn type="integer"&gt; 1 &lt;/cn&gt;
          &lt;/math&gt;
        &lt;/eventAssignment&gt;
      &lt;/listOfEventAssignments&gt;
    &lt;/event&gt;
    <event id="mwaa5977a5_fe8b_44f3_ad5b_b037fc4c4a1e"
name="event_106">
      &lt;trigger&gt;
        &lt;math xmlns="http://www.w3.org/1998/Math/MathML"&gt;
          &lt;apply&gt;
            &lt;geq/&gt;
            <csymbol encoding="text"
definitionURL="http://www.sbml.org/sbml/symbols/time"> time &lt;/csymbol&gt;
            &lt;cn&gt; 116.1 &lt;/cn&gt;
          &lt;/apply&gt;
        &lt;/math&gt;
      &lt;/trigger&gt;
      &lt;listOfEventAssignments&gt;
        <eventAssignment
variable="mwcedbe49e_2d28_4720_8fcd_207db64228cf">
          &lt;math xmlns="http://www.w3.org/1998/Math/MathML"&gt;
            &lt;cn type="integer"&gt; 0 &lt;/cn&gt;
          &lt;/math&gt;
        &lt;/eventAssignment&gt;
      &lt;/listOfEventAssignments&gt;
    &lt;/event&gt;
    <event id="mwae4848c6_06e6_4752_b029_3f3681c42d27"
name="event_107">
      &lt;trigger&gt;
        &lt;math xmlns="http://www.w3.org/1998/Math/MathML"&gt;
          &lt;apply&gt;
            &lt;geq/&gt;
            <csymbol encoding="text"
definitionURL="http://www.sbml.org/sbml/symbols/time"> time &lt;/csymbol&gt;
            &lt;cn&gt; 118.97 &lt;/cn&gt;
          &lt;/apply&gt;
        &lt;/math&gt;
      &lt;/trigger&gt;
      &lt;listOfEventAssignments&gt;
        <eventAssignment
variable="mwcedbe49e_2d28_4720_8fcd_207db64228cf">
```

```
            &lt;math xmlns="http://www.w3.org/1998/Math/MathML"&gt;
              &lt;cn type="integer"&gt; 1 &lt;/cn&gt;
            &lt;/math&gt;
          &lt;/eventAssignment&gt;
        &lt;/listOfEventAssignments&gt;
      &lt;/event&gt;
      <event id="mw84a24786_4ab0_4380_acf4_205819bc6f90"
name="event_108">
        &lt;trigger&gt;
          &lt;math xmlns="http://www.w3.org/1998/Math/MathML"&gt;
            &lt;apply&gt;
              &lt;geq/&gt;
              <csymbol encoding="text"
definitionURL="http://www.sbml.org/sbml/symbols/time"> time &lt;/csymbol&gt;
              &lt;cn&gt; 120.4 &lt;/cn&gt;
            &lt;/apply&gt;
          &lt;/math&gt;
        &lt;/trigger&gt;
        &lt;listOfEventAssignments&gt;
          <eventAssignment
variable="mwcedbe49e_2d28_4720_8fcd_207db64228cf">
            &lt;math xmlns="http://www.w3.org/1998/Math/MathML"&gt;
              &lt;cn type="integer"&gt; 0 &lt;/cn&gt;
            &lt;/math&gt;
          &lt;/eventAssignment&gt;
        &lt;/listOfEventAssignments&gt;
      &lt;/event&gt;
      <event id="mw775d1bcf_9cc5_4354_a251_880b41cdf927"
name="event_109">
        &lt;trigger&gt;
          &lt;math xmlns="http://www.w3.org/1998/Math/MathML"&gt;
            &lt;apply&gt;
              &lt;geq/&gt;
              <csymbol encoding="text"
definitionURL="http://www.sbml.org/sbml/symbols/time"> time &lt;/csymbol&gt;
              &lt;cn&gt; 123.27 &lt;/cn&gt;
            &lt;/apply&gt;
          &lt;/math&gt;
        &lt;/trigger&gt;
        &lt;listOfEventAssignments&gt;
          <eventAssignment
variable="mwcedbe49e_2d28_4720_8fcd_207db64228cf">
            &lt;math xmlns="http://www.w3.org/1998/Math/MathML"&gt;
              &lt;cn type="integer"&gt; 1 &lt;/cn&gt;
            &lt;/math&gt;
          &lt;/eventAssignment&gt;
        &lt;/listOfEventAssignments&gt;
      &lt;/event&gt;
      <event id="mw0fcd1b38_0d15_402c_b6bd_558fb7b568e6"
name="event_110">
        &lt;trigger&gt;
          &lt;math xmlns="http://www.w3.org/1998/Math/MathML"&gt;
```

```
          <apply>
            <geq/>
            <csymbol encoding="text"
definitionURL="http://www.sbml.org/sbml/symbols/time"> time </csymbol>
            <cn> 124.7 </cn>
          </apply>
        </math>
      </trigger>
      <listOfEventAssignments>
        <eventAssignment
variable="mwcedbe49e_2d28_4720_8fcd_207db64228cf">
          <math xmlns="http://www.w3.org/1998/Math/MathML">
            <cn type="integer"> 0 </cn>
          </math>
        </eventAssignment>
      </listOfEventAssignments>
    </event>
    <event id="mwd4a5884f_2e76_4bc7_b381_2b31b200c129"
name="event_111">
      <trigger>
        <math xmlns="http://www.w3.org/1998/Math/MathML">
          <apply>
            <geq/>
            <csymbol encoding="text"
definitionURL="http://www.sbml.org/sbml/symbols/time"> time </csymbol>
            <cn> 127.57 </cn>
          </apply>
        </math>
      </trigger>
      <listOfEventAssignments>
        <eventAssignment
variable="mwcedbe49e_2d28_4720_8fcd_207db64228cf">
          <math xmlns="http://www.w3.org/1998/Math/MathML">
            <cn type="integer"> 1 </cn>
          </math>
        </eventAssignment>
      </listOfEventAssignments>
    </event>
    <event id="mw4677bbd4_141b_41d4_8c0b_f509bcdcb3d6"
name="event_112">
      <trigger>
        <math xmlns="http://www.w3.org/1998/Math/MathML">
          <apply>
            <geq/>
            <csymbol encoding="text"
definitionURL="http://www.sbml.org/sbml/symbols/time"> time </csymbol>
            <cn type="integer"> 129 </cn>
          </apply>
        </math>
      </trigger>
      <listOfEventAssignments>
```

```
          <eventAssignment
variable="mwcedbe49e_2d28_4720_8fcd_207db64228cf">
            &lt;math xmlns="http://www.w3.org/1998/Math/MathML"&gt;
              &lt;cn type="integer"&gt; 0 &lt;/cn&gt;
            &lt;/math&gt;
          &lt;/eventAssignment&gt;
        &lt;/listOfEventAssignments&gt;
      &lt;/event&gt;
      <event id="mw4028f4df_1402_4688_92d1_bee5480550ae"
name="event_113">
        &lt;trigger&gt;
          &lt;math xmlns="http://www.w3.org/1998/Math/MathML"&gt;
            &lt;apply&gt;
              &lt;geq/&gt;
              <csymbol encoding="text"
definitionURL="http://www.sbml.org/sbml/symbols/time"> time &lt;/csymbol&gt;
              &lt;cn&gt; 131.87 &lt;/cn&gt;
            &lt;/apply&gt;
          &lt;/math&gt;
        &lt;/trigger&gt;
        &lt;listOfEventAssignments&gt;
          <eventAssignment
variable="mwcedbe49e_2d28_4720_8fcd_207db64228cf">
            &lt;math xmlns="http://www.w3.org/1998/Math/MathML"&gt;
              &lt;cn type="integer"&gt; 1 &lt;/cn&gt;
            &lt;/math&gt;
          &lt;/eventAssignment&gt;
        &lt;/listOfEventAssignments&gt;
      &lt;/event&gt;
      <event id="mw756affbb_1d89_41e0_bd58_291d1bed6ab7"
name="event_114">
        &lt;trigger&gt;
          &lt;math xmlns="http://www.w3.org/1998/Math/MathML"&gt;
            &lt;apply&gt;
              &lt;geq/&gt;
              <csymbol encoding="text"
definitionURL="http://www.sbml.org/sbml/symbols/time"> time &lt;/csymbol&gt;
              &lt;cn&gt; 133.3 &lt;/cn&gt;
            &lt;/apply&gt;
          &lt;/math&gt;
        &lt;/trigger&gt;
        &lt;listOfEventAssignments&gt;
          <eventAssignment
variable="mwcedbe49e_2d28_4720_8fcd_207db64228cf">
            &lt;math xmlns="http://www.w3.org/1998/Math/MathML"&gt;
              &lt;cn type="integer"&gt; 0 &lt;/cn&gt;
            &lt;/math&gt;
          &lt;/eventAssignment&gt;
        &lt;/listOfEventAssignments&gt;
      &lt;/event&gt;
      <event id="mw8085c338_2ec6_4edd_b700_66380670d990"
name="event_115">
```

```
        <trigger>
          <math xmlns="http://www.w3.org/1998/Math/MathML">
            <apply>
              <geq/>
              <csymbol encoding="text"
definitionURL="http://www.sbml.org/sbml/symbols/time"> time </csymbol>
              <cn> 136.17 </cn>
            </apply>
          </math>
        </trigger>
        <listOfEventAssignments>
          <eventAssignment
variable="mwcedbe49e_2d28_4720_8fcd_207db64228cf">
            <math xmlns="http://www.w3.org/1998/Math/MathML">
              <cn type="integer"> 1 </cn>
            </math>
          </eventAssignment>
        </listOfEventAssignments>
      </event>
      <event id="mw815f5595_7bac_4346_9af8_698433b23948"
name="event_116">
        <trigger>
          <math xmlns="http://www.w3.org/1998/Math/MathML">
            <apply>
              <geq/>
              <csymbol encoding="text"
definitionURL="http://www.sbml.org/sbml/symbols/time"> time </csymbol>
              <cn> 137.6 </cn>
            </apply>
          </math>
        </trigger>
        <listOfEventAssignments>
          <eventAssignment
variable="mwcedbe49e_2d28_4720_8fcd_207db64228cf">
            <math xmlns="http://www.w3.org/1998/Math/MathML">
              <cn type="integer"> 0 </cn>
            </math>
          </eventAssignment>
        </listOfEventAssignments>
      </event>
      <event id="mw3b936fa0_9860_455e_ae4b_f88bbade0a43"
name="event_117">
        <trigger>
          <math xmlns="http://www.w3.org/1998/Math/MathML">
            <apply>
              <geq/>
              <csymbol encoding="text"
definitionURL="http://www.sbml.org/sbml/symbols/time"> time </csymbol>
              <cn> 140.47 </cn>
            </apply>
          </math>
        </trigger>
```

```
        <listOfEventAssignments>
          <eventAssignment
variable="mwcedbe49e_2d28_4720_8fcd_207db64228cf">
            <math xmlns="http://www.w3.org/1998/Math/MathML">
              <cn type="integer"> 1 </cn>
            </math>
          </eventAssignment>
        </listOfEventAssignments>
      </event>
      <event id="mwb0af20a4_e288_4d84_8ade_d1d292577034"
name="event_118">
        <trigger>
          <math xmlns="http://www.w3.org/1998/Math/MathML">
            <apply>
              <geq/>
              <csymbol encoding="text"
definitionURL="http://www.sbml.org/sbml/symbols/time"> time </csymbol>
              <cn> 141.9 </cn>
            </apply>
          </math>
        </trigger>
        <listOfEventAssignments>
          <eventAssignment
variable="mwcedbe49e_2d28_4720_8fcd_207db64228cf">
            <math xmlns="http://www.w3.org/1998/Math/MathML">
              <cn type="integer"> 0 </cn>
            </math>
          </eventAssignment>
        </listOfEventAssignments>
      </event>
      <event id="mw20ca8125_751b_4ef3_9fe1_2cde03e6a0db"
name="event_119">
        <trigger>
          <math xmlns="http://www.w3.org/1998/Math/MathML">
            <apply>
              <geq/>
              <csymbol encoding="text"
definitionURL="http://www.sbml.org/sbml/symbols/time"> time </csymbol>
              <cn> 144.77 </cn>
            </apply>
          </math>
        </trigger>
        <listOfEventAssignments>
          <eventAssignment
variable="mwcedbe49e_2d28_4720_8fcd_207db64228cf">
            <math xmlns="http://www.w3.org/1998/Math/MathML">
              <cn type="integer"> 1 </cn>
            </math>
          </eventAssignment>
        </listOfEventAssignments>
      </event>
```

```
      <event id="mw4a94f86c_533a_4deb_875c_39af65672ce6"
name="event_120">
        <trigger>
          <math xmlns="http://www.w3.org/1998/Math/MathML">
            <apply>
              <geq/>
              <csymbol encoding="text"
definitionURL="http://www.sbml.org/sbml/symbols/time"> time </csymbol>
              <cn> 146.2 </cn>
            </apply>
          </math>
        </trigger>
        <listOfEventAssignments>
          <eventAssignment
variable="mwcedbe49e_2d28_4720_8fcd_207db64228cf">
            <math xmlns="http://www.w3.org/1998/Math/MathML">
              <cn type="integer"> 0 </cn>
            </math>
          </eventAssignment>
        </listOfEventAssignments>
      </event>
      <event id="mw6ef869a8_eb43_4955_abe0_ec9768990cca"
name="event_121">
        <trigger>
          <math xmlns="http://www.w3.org/1998/Math/MathML">
            <apply>
              <geq/>
              <csymbol encoding="text"
definitionURL="http://www.sbml.org/sbml/symbols/time"> time </csymbol>
              <cn> 149.07 </cn>
            </apply>
          </math>
        </trigger>
        <listOfEventAssignments>
          <eventAssignment
variable="mwcedbe49e_2d28_4720_8fcd_207db64228cf">
            <math xmlns="http://www.w3.org/1998/Math/MathML">
              <cn type="integer"> 1 </cn>
            </math>
          </eventAssignment>
        </listOfEventAssignments>
      </event>
      <event id="mwcc76e719_5723_489c_99cb_59b1f54bdb25"
name="event_122">
        <trigger>
          <math xmlns="http://www.w3.org/1998/Math/MathML">
            <apply>
              <geq/>
              <csymbol encoding="text"
definitionURL="http://www.sbml.org/sbml/symbols/time"> time </csymbol>
              <cn> 150.5 </cn>
            </apply>
```

```
          </math>
        </trigger>
        <listOfEventAssignments>
          <eventAssignment
variable="mwcedbe49e_2d28_4720_8fcd_207db64228cf">
            <math xmlns="http://www.w3.org/1998/Math/MathML">
              <cn type="integer"> 0 </cn>
            </math>
          </eventAssignment>
        </listOfEventAssignments>
      </event>
      <event id="mw1cb43de1_d929_433c_9475_b6b84eda46d9"
name="event_123">
        <trigger>
          <math xmlns="http://www.w3.org/1998/Math/MathML">
            <apply>
              <geq/>
              <csymbol encoding="text"
definitionURL="http://www.sbml.org/sbml/symbols/time"> time </csymbol>
              <cn> 153.37 </cn>
            </apply>
          </math>
        </trigger>
        <listOfEventAssignments>
          <eventAssignment
variable="mwcedbe49e_2d28_4720_8fcd_207db64228cf">
            <math xmlns="http://www.w3.org/1998/Math/MathML">
              <cn type="integer"> 1 </cn>
            </math>
          </eventAssignment>
        </listOfEventAssignments>
      </event>
      <event id="mwca308cf5_5f47_4808_aba5_72b682c26e01"
name="event_124">
        <trigger>
          <math xmlns="http://www.w3.org/1998/Math/MathML">
            <apply>
              <geq/>
              <csymbol encoding="text"
definitionURL="http://www.sbml.org/sbml/symbols/time"> time </csymbol>
              <cn> 154.8 </cn>
            </apply>
          </math>
        </trigger>
        <listOfEventAssignments>
          <eventAssignment
variable="mwcedbe49e_2d28_4720_8fcd_207db64228cf">
            <math xmlns="http://www.w3.org/1998/Math/MathML">
              <cn type="integer"> 0 </cn>
            </math>
          </eventAssignment>
        </listOfEventAssignments>
```

```
      </event>
      <event id="mw4df988b0_1435_404b_88f2_498bee5155ca"
name="event_125">
        <trigger>
          <math xmlns="http://www.w3.org/1998/Math/MathML">
            <apply>
              <geq/>
              <csymbol encoding="text"
definitionURL="http://www.sbml.org/sbml/symbols/time"> time </csymbol>
              <cn> 157.67 </cn>
            </apply>
          </math>
        </trigger>
        <listOfEventAssignments>
          <eventAssignment
variable="mwcedbe49e_2d28_4720_8fcd_207db64228cf">
            <math xmlns="http://www.w3.org/1998/Math/MathML">
              <cn type="integer"> 1 </cn>
            </math>
          </eventAssignment>
        </listOfEventAssignments>
      </event>
      <event id="mw8525968e_860a_4abb_b379_d581790d3158"
name="event_126">
        <trigger>
          <math xmlns="http://www.w3.org/1998/Math/MathML">
            <apply>
              <geq/>
              <csymbol encoding="text"
definitionURL="http://www.sbml.org/sbml/symbols/time"> time </csymbol>
              <cn> 159.1 </cn>
            </apply>
          </math>
        </trigger>
        <listOfEventAssignments>
          <eventAssignment
variable="mwcedbe49e_2d28_4720_8fcd_207db64228cf">
            <math xmlns="http://www.w3.org/1998/Math/MathML">
              <cn type="integer"> 0 </cn>
            </math>
          </eventAssignment>
        </listOfEventAssignments>
      </event>
      <event id="mwe97046b6_33fe_4ef1_9156_3e37c2f1a1ad"
name="event_127">
        <trigger>
          <math xmlns="http://www.w3.org/1998/Math/MathML">
            <apply>
              <geq/>
              <csymbol encoding="text"
definitionURL="http://www.sbml.org/sbml/symbols/time"> time </csymbol>
              <cn> 161.97 </cn>
```

```
            &lt;/apply&gt;
          &lt;/math&gt;
        &lt;/trigger&gt;
        &lt;listOfEventAssignments&gt;
          <eventAssignment
variable="mwcedbe49e_2d28_4720_8fcd_207db64228cf">
            &lt;math xmlns="http://www.w3.org/1998/Math/MathML"&gt;
              &lt;cn type="integer"&gt; 1 &lt;/cn&gt;
            &lt;/math&gt;
          &lt;/eventAssignment&gt;
        &lt;/listOfEventAssignments&gt;
      &lt;/event&gt;
      <event id="mw08ce16cd_8f2f_40f2_9b8d_a5ede98dd159"
name="event_128">
        &lt;trigger&gt;
          &lt;math xmlns="http://www.w3.org/1998/Math/MathML"&gt;
            &lt;apply&gt;
              &lt;geq/&gt;
              <csymbol encoding="text"
definitionURL="http://www.sbml.org/sbml/symbols/time"> time &lt;/csymbol&gt;
              &lt;cn&gt; 163.4 &lt;/cn&gt;
            &lt;/apply&gt;
          &lt;/math&gt;
        &lt;/trigger&gt;
        &lt;listOfEventAssignments&gt;
          <eventAssignment
variable="mwcedbe49e_2d28_4720_8fcd_207db64228cf">
            &lt;math xmlns="http://www.w3.org/1998/Math/MathML"&gt;
              &lt;cn type="integer"&gt; 0 &lt;/cn&gt;
            &lt;/math&gt;
          &lt;/eventAssignment&gt;
        &lt;/listOfEventAssignments&gt;
      &lt;/event&gt;
      <event id="mw80866077_e0c6_428f_803c_f05c3aebb404"
name="event_129">
        &lt;trigger&gt;
          &lt;math xmlns="http://www.w3.org/1998/Math/MathML"&gt;
            &lt;apply&gt;
              &lt;geq/&gt;
              <csymbol encoding="text"
definitionURL="http://www.sbml.org/sbml/symbols/time"> time &lt;/csymbol&gt;
              &lt;cn&gt; 166.27 &lt;/cn&gt;
            &lt;/apply&gt;
          &lt;/math&gt;
        &lt;/trigger&gt;
        &lt;listOfEventAssignments&gt;
          <eventAssignment
variable="mwcedbe49e_2d28_4720_8fcd_207db64228cf">
            &lt;math xmlns="http://www.w3.org/1998/Math/MathML"&gt;
              &lt;cn type="integer"&gt; 1 &lt;/cn&gt;
            &lt;/math&gt;
          &lt;/eventAssignment&gt;
```

```
        </listOfEventAssignments>
      </event>
      <event id="mw633fa921_f2e0_471b_bf6d_1d2df65292e4"
name="event_130">
        <trigger>
          <math xmlns="http://www.w3.org/1998/Math/MathML">
            <apply>
              <geq/>
              <csymbol encoding="text"
definitionURL="http://www.sbml.org/sbml/symbols/time"> time </csymbol>
              <cn> 167.7 </cn>
            </apply>
          </math>
        </trigger>
        <listOfEventAssignments>
          <eventAssignment
variable="mwcedbe49e_2d28_4720_8fcd_207db64228cf">
            <math xmlns="http://www.w3.org/1998/Math/MathML">
              <cn type="integer"> 0 </cn>
            </math>
          </eventAssignment>
        </listOfEventAssignments>
      </event>
      <event id="mwd12aeb63_fb36_4ea5_8b41_3f90bbae82d0"
name="event_131">
        <trigger>
          <math xmlns="http://www.w3.org/1998/Math/MathML">
            <apply>
              <geq/>
              <csymbol encoding="text"
definitionURL="http://www.sbml.org/sbml/symbols/time"> time </csymbol>
              <cn> 170.57 </cn>
            </apply>
          </math>
        </trigger>
        <listOfEventAssignments>
          <eventAssignment
variable="mwcedbe49e_2d28_4720_8fcd_207db64228cf">
            <math xmlns="http://www.w3.org/1998/Math/MathML">
              <cn type="integer"> 1 </cn>
            </math>
          </eventAssignment>
        </listOfEventAssignments>
      </event>
      <event id="mw2b886551_4978_4768_afff_53fa86ce2697"
name="event_132">
        <trigger>
          <math xmlns="http://www.w3.org/1998/Math/MathML">
            <apply>
              <geq/>
              <csymbol encoding="text"
definitionURL="http://www.sbml.org/sbml/symbols/time"> time </csymbol>
```

```
              <cn type="integer"> 172 </cn>
            </apply>
          </math>
        </trigger>
        <listOfEventAssignments>
          <eventAssignment
variable="mwcedbe49e_2d28_4720_8fcd_207db64228cf">
            <math xmlns="http://www.w3.org/1998/Math/MathML">
              <cn type="integer"> 0 </cn>
            </math>
          </eventAssignment>
        </listOfEventAssignments>
      </event>
      <event id="mw0c063e72_c04a_42c2_adac_fc0b3877a526"
name="event_133">
        <trigger>
          <math xmlns="http://www.w3.org/1998/Math/MathML">
            <apply>
              <geq/>
              <csymbol encoding="text"
definitionURL="http://www.sbml.org/sbml/symbols/time"> time </csymbol>
              <cn> 174.87 </cn>
            </apply>
          </math>
        </trigger>
        <listOfEventAssignments>
          <eventAssignment
variable="mwcedbe49e_2d28_4720_8fcd_207db64228cf">
            <math xmlns="http://www.w3.org/1998/Math/MathML">
              <cn type="integer"> 1 </cn>
            </math>
          </eventAssignment>
        </listOfEventAssignments>
      </event>
      <event id="mwce8882de_644c_407e_a7f5_5c074f3a2d9a"
name="event_134">
        <trigger>
          <math xmlns="http://www.w3.org/1998/Math/MathML">
            <apply>
              <geq/>
              <csymbol encoding="text"
definitionURL="http://www.sbml.org/sbml/symbols/time"> time </csymbol>
              <cn> 176.3 </cn>
            </apply>
          </math>
        </trigger>
        <listOfEventAssignments>
          <eventAssignment
variable="mwcedbe49e_2d28_4720_8fcd_207db64228cf">
            <math xmlns="http://www.w3.org/1998/Math/MathML">
              <cn type="integer"> 0 </cn>
            </math>
```

```
          &lt;/eventAssignment&gt;
        &lt;/listOfEventAssignments&gt;
      &lt;/event&gt;
      <event id="mwfc20b106_8d94_41fb_a3c8_36824d767cd3"
name="event_135">
        &lt;trigger&gt;
          &lt;math xmlns="http://www.w3.org/1998/Math/MathML"&gt;
            &lt;apply&gt;
              &lt;geq/&gt;
              <csymbol encoding="text"
definitionURL="http://www.sbml.org/sbml/symbols/time"> time &lt;/csymbol&gt;
              &lt;cn&gt; 114.67 &lt;/cn&gt;
            &lt;/apply&gt;
          &lt;/math&gt;
        &lt;/trigger&gt;
        &lt;listOfEventAssignments&gt;
          <eventAssignment
variable="mw7d178aae_ff78_412a_830f_3be325d22773">
            &lt;math xmlns="http://www.w3.org/1998/Math/MathML"&gt;
              &lt;cn type="integer"&gt; 0 &lt;/cn&gt;
            &lt;/math&gt;
          &lt;/eventAssignment&gt;
        &lt;/listOfEventAssignments&gt;
      &lt;/event&gt;
      <event id="mwd1e3b0bf_7a38_456c_9061_598e2b3013bc"
name="event_136">
        &lt;trigger&gt;
          &lt;math xmlns="http://www.w3.org/1998/Math/MathML"&gt;
            &lt;apply&gt;
              &lt;geq/&gt;
              <csymbol encoding="text"
definitionURL="http://www.sbml.org/sbml/symbols/time"> time &lt;/csymbol&gt;
              &lt;cn&gt; 116.1 &lt;/cn&gt;
            &lt;/apply&gt;
          &lt;/math&gt;
        &lt;/trigger&gt;
        &lt;listOfEventAssignments&gt;
          <eventAssignment
variable="mw7d178aae_ff78_412a_830f_3be325d22773">
            &lt;math xmlns="http://www.w3.org/1998/Math/MathML"&gt;
              &lt;cn type="integer"&gt; 1 &lt;/cn&gt;
            &lt;/math&gt;
          &lt;/eventAssignment&gt;
        &lt;/listOfEventAssignments&gt;
      &lt;/event&gt;
      <event id="mwa75a457b_26b4_4034_befa_4d9dca6d8330"
name="event_137">
        &lt;trigger&gt;
          &lt;math xmlns="http://www.w3.org/1998/Math/MathML"&gt;
            &lt;apply&gt;
              &lt;geq/&gt;
```

```
              <csymbol encoding="text"
definitionURL="http://www.sbml.org/sbml/symbols/time"> time </csymbol>
              <cn> 118.97 </cn>
            </apply>
          </math>
        </trigger>
        <listOfEventAssignments>
          <eventAssignment
variable="mw7d178aae_ff78_412a_830f_3be325d22773">
            <math xmlns="http://www.w3.org/1998/Math/MathML">
              <cn type="integer"> 0 </cn>
            </math>
          </eventAssignment>
        </listOfEventAssignments>
      </event>
      <event id="mwb27ee007_b1c6_4a8d_98a5_25b9ac168d92"
name="event_138">
        <trigger>
          <math xmlns="http://www.w3.org/1998/Math/MathML">
            <apply>
              <geq/>
              <csymbol encoding="text"
definitionURL="http://www.sbml.org/sbml/symbols/time"> time </csymbol>
              <cn> 120.4 </cn>
            </apply>
          </math>
        </trigger>
        <listOfEventAssignments>
          <eventAssignment
variable="mw7d178aae_ff78_412a_830f_3be325d22773">
            <math xmlns="http://www.w3.org/1998/Math/MathML">
              <cn type="integer"> 1 </cn>
            </math>
          </eventAssignment>
        </listOfEventAssignments>
      </event>
      <event id="mw92c9f3c3_60ae_4a75_95b8_655588a1c262"
name="event_139">
        <trigger>
          <math xmlns="http://www.w3.org/1998/Math/MathML">
            <apply>
              <geq/>
              <csymbol encoding="text"
definitionURL="http://www.sbml.org/sbml/symbols/time"> time </csymbol>
              <cn> 123.27 </cn>
            </apply>
          </math>
        </trigger>
        <listOfEventAssignments>
          <eventAssignment
variable="mw7d178aae_ff78_412a_830f_3be325d22773">
            <math xmlns="http://www.w3.org/1998/Math/MathML">
```

```
              <cn type="integer"> 0 </cn>
            </math>
          </eventAssignment>
        </listOfEventAssignments>
      </event>
      <event id="mw8de2767c_a143_4c0d_a8c3_5d7fce520973"
name="event_140">
        <trigger>
          <math xmlns="http://www.w3.org/1998/Math/MathML">
            <apply>
              <geq/>
              <csymbol encoding="text"
definitionURL="http://www.sbml.org/sbml/symbols/time"> time </csymbol>
              <cn> 124.7 </cn>
            </apply>
          </math>
        </trigger>
        <listOfEventAssignments>
          <eventAssignment
variable="mw7d178aae_ff78_412a_830f_3be325d22773">
            <math xmlns="http://www.w3.org/1998/Math/MathML">
              <cn type="integer"> 1 </cn>
            </math>
          </eventAssignment>
        </listOfEventAssignments>
      </event>
      <event id="mw683214ec_9a5c_4ade_be28_e9fb652406dd"
name="event_141">
        <trigger>
          <math xmlns="http://www.w3.org/1998/Math/MathML">
            <apply>
              <geq/>
              <csymbol encoding="text"
definitionURL="http://www.sbml.org/sbml/symbols/time"> time </csymbol>
              <cn> 127.57 </cn>
            </apply>
          </math>
        </trigger>
        <listOfEventAssignments>
          <eventAssignment
variable="mw7d178aae_ff78_412a_830f_3be325d22773">
            <math xmlns="http://www.w3.org/1998/Math/MathML">
              <cn type="integer"> 0 </cn>
            </math>
          </eventAssignment>
        </listOfEventAssignments>
      </event>
      <event id="mwa2265ada_89b6_43ba_bc8a_a43052e941de"
name="event_142">
        <trigger>
          <math xmlns="http://www.w3.org/1998/Math/MathML">
            <apply>
```

```
              &lt;geq/&gt;
              <csymbol encoding="text"
definitionURL="http://www.sbml.org/sbml/symbols/time"> time &lt;/csymbol&gt;
              &lt;cn type="integer"&gt; 129 &lt;/cn&gt;
            &lt;/apply&gt;
          &lt;/math&gt;
        &lt;/trigger&gt;
        &lt;listOfEventAssignments&gt;
          <eventAssignment
variable="mw7d178aae_ff78_412a_830f_3be325d22773">
            &lt;math xmlns="http://www.w3.org/1998/Math/MathML"&gt;
              &lt;cn type="integer"&gt; 1 &lt;/cn&gt;
            &lt;/math&gt;
          &lt;/eventAssignment&gt;
        &lt;/listOfEventAssignments&gt;
      &lt;/event&gt;
      <event id="mw5e398ce6_9ca0_4e0d_9aa3_cb54a8f5e006"
name="event_143">
        &lt;trigger&gt;
          &lt;math xmlns="http://www.w3.org/1998/Math/MathML"&gt;
            &lt;apply&gt;
              &lt;geq/&gt;
              <csymbol encoding="text"
definitionURL="http://www.sbml.org/sbml/symbols/time"> time &lt;/csymbol&gt;
              &lt;cn&gt; 131.87 &lt;/cn&gt;
            &lt;/apply&gt;
          &lt;/math&gt;
        &lt;/trigger&gt;
        &lt;listOfEventAssignments&gt;
          <eventAssignment
variable="mw7d178aae_ff78_412a_830f_3be325d22773">
            &lt;math xmlns="http://www.w3.org/1998/Math/MathML"&gt;
              &lt;cn type="integer"&gt; 0 &lt;/cn&gt;
            &lt;/math&gt;
          &lt;/eventAssignment&gt;
        &lt;/listOfEventAssignments&gt;
      &lt;/event&gt;
      <event id="mwfc463fb3_1306_4cc1_867e_20228024e122"
name="event_144">
        &lt;trigger&gt;
          &lt;math xmlns="http://www.w3.org/1998/Math/MathML"&gt;
            &lt;apply&gt;
              &lt;geq/&gt;
              <csymbol encoding="text"
definitionURL="http://www.sbml.org/sbml/symbols/time"> time &lt;/csymbol&gt;
              &lt;cn&gt; 133.3 &lt;/cn&gt;
            &lt;/apply&gt;
          &lt;/math&gt;
        &lt;/trigger&gt;
        &lt;listOfEventAssignments&gt;
          <eventAssignment
variable="mw7d178aae_ff78_412a_830f_3be325d22773">
```

```
            <math xmlns="http://www.w3.org/1998/Math/MathML">
              <cn type="integer"> 1 </cn>
            </math>
          </eventAssignment>
        </listOfEventAssignments>
      </event>
      <event id="mw5cc541a1_6975_42a6_b216_57f7ffe7a4dc"
name="event_145">
        <trigger>
          <math xmlns="http://www.w3.org/1998/Math/MathML">
            <apply>
              <geq/>
              <csymbol encoding="text"
definitionURL="http://www.sbml.org/sbml/symbols/time"> time </csymbol>
              <cn> 136.17 </cn>
            </apply>
          </math>
        </trigger>
        <listOfEventAssignments>
          <eventAssignment
variable="mw7d178aae_ff78_412a_830f_3be325d22773">
            <math xmlns="http://www.w3.org/1998/Math/MathML">
              <cn type="integer"> 0 </cn>
            </math>
          </eventAssignment>
        </listOfEventAssignments>
      </event>
      <event id="mwbb417ab3_b2f8_4d5b_b543_68c225dd0366"
name="event_146">
        <trigger>
          <math xmlns="http://www.w3.org/1998/Math/MathML">
            <apply>
              <geq/>
              <csymbol encoding="text"
definitionURL="http://www.sbml.org/sbml/symbols/time"> time </csymbol>
              <cn> 137.6 </cn>
            </apply>
          </math>
        </trigger>
        <listOfEventAssignments>
          <eventAssignment
variable="mw7d178aae_ff78_412a_830f_3be325d22773">
            <math xmlns="http://www.w3.org/1998/Math/MathML">
              <cn type="integer"> 1 </cn>
            </math>
          </eventAssignment>
        </listOfEventAssignments>
      </event>
      <event id="mwe0889250_1287_4abe_8ed6_32be294c1236"
name="event_147">
        <trigger>
          <math xmlns="http://www.w3.org/1998/Math/MathML">
```

```
            <apply>
              <geq/>
              <csymbol encoding="text"
definitionURL="http://www.sbml.org/sbml/symbols/time"> time </csymbol>
              <cn> 140.47 </cn>
            </apply>
          </math>
        </trigger>
        <listOfEventAssignments>
          <eventAssignment
variable="mw7d178aae_ff78_412a_830f_3be325d22773">
            <math xmlns="http://www.w3.org/1998/Math/MathML">
              <cn type="integer"> 0 </cn>
            </math>
          </eventAssignment>
        </listOfEventAssignments>
      </event>
      <event id="mw4e273a21_6194_46d8_98a5_0fd676901fd9"
name="event_148">
        <trigger>
          <math xmlns="http://www.w3.org/1998/Math/MathML">
            <apply>
              <geq/>
              <csymbol encoding="text"
definitionURL="http://www.sbml.org/sbml/symbols/time"> time </csymbol>
              <cn> 141.9 </cn>
            </apply>
          </math>
        </trigger>
        <listOfEventAssignments>
          <eventAssignment
variable="mw7d178aae_ff78_412a_830f_3be325d22773">
            <math xmlns="http://www.w3.org/1998/Math/MathML">
              <cn type="integer"> 1 </cn>
            </math>
          </eventAssignment>
        </listOfEventAssignments>
      </event>
      <event id="mw09b4dd31_5d8c_4761_92dd_a233221a34e6"
name="event_149">
        <trigger>
          <math xmlns="http://www.w3.org/1998/Math/MathML">
            <apply>
              <geq/>
              <csymbol encoding="text"
definitionURL="http://www.sbml.org/sbml/symbols/time"> time </csymbol>
              <cn> 144.77 </cn>
            </apply>
          </math>
        </trigger>
        <listOfEventAssignments>
```

```
          <eventAssignment
variable="mw7d178aae_ff78_412a_830f_3be325d22773">
            <math xmlns="http://www.w3.org/1998/Math/MathML">
              <cn type="integer"> 0 </cn>
            </math>
          </eventAssignment>
        </listOfEventAssignments>
      </event>
      <event id="mw992bb146_04d4_4559_8bee_cacc3b9f5baf"
name="event_150">
        <trigger>
          <math xmlns="http://www.w3.org/1998/Math/MathML">
            <apply>
              <geq/>
              <csymbol encoding="text"
definitionURL="http://www.sbml.org/sbml/symbols/time"> time </csymbol>
              <cn> 146.2 </cn>
            </apply>
          </math>
        </trigger>
        <listOfEventAssignments>
          <eventAssignment
variable="mw7d178aae_ff78_412a_830f_3be325d22773">
            <math xmlns="http://www.w3.org/1998/Math/MathML">
              <cn type="integer"> 1 </cn>
            </math>
          </eventAssignment>
        </listOfEventAssignments>
      </event>
      <event id="mw4f9c4fc4_2799_46a5_9575_3eaecdc58deb"
name="event_151">
        <trigger>
          <math xmlns="http://www.w3.org/1998/Math/MathML">
            <apply>
              <geq/>
              <csymbol encoding="text"
definitionURL="http://www.sbml.org/sbml/symbols/time"> time </csymbol>
              <cn> 149.07 </cn>
            </apply>
          </math>
        </trigger>
        <listOfEventAssignments>
          <eventAssignment
variable="mw7d178aae_ff78_412a_830f_3be325d22773">
            <math xmlns="http://www.w3.org/1998/Math/MathML">
              <cn type="integer"> 0 </cn>
            </math>
          </eventAssignment>
        </listOfEventAssignments>
      </event>
      <event id="mw021e3975_b755_42fa_8f19_f9ed33313818"
name="event_152">
```

```
        &lt;trigger&gt;
          &lt;math xmlns="http://www.w3.org/1998/Math/MathML"&gt;
            &lt;apply&gt;
              &lt;geq/&gt;
              <csymbol encoding="text"
definitionURL="http://www.sbml.org/sbml/symbols/time"> time &lt;/csymbol&gt;
              &lt;cn&gt; 150.5 &lt;/cn&gt;
            &lt;/apply&gt;
          &lt;/math&gt;
        &lt;/trigger&gt;
        &lt;listOfEventAssignments&gt;
          <eventAssignment
variable="mw7d178aae_ff78_412a_830f_3be325d22773">
            &lt;math xmlns="http://www.w3.org/1998/Math/MathML"&gt;
              &lt;cn type="integer"&gt; 1 &lt;/cn&gt;
            &lt;/math&gt;
          &lt;/eventAssignment&gt;
        &lt;/listOfEventAssignments&gt;
      &lt;/event&gt;
      <event id="mw454452c2_2b23_4385_870f_03d3738bbcf6"
name="event_153">
        &lt;trigger&gt;
          &lt;math xmlns="http://www.w3.org/1998/Math/MathML"&gt;
            &lt;apply&gt;
              &lt;geq/&gt;
              <csymbol encoding="text"
definitionURL="http://www.sbml.org/sbml/symbols/time"> time &lt;/csymbol&gt;
              &lt;cn&gt; 153.37 &lt;/cn&gt;
            &lt;/apply&gt;
          &lt;/math&gt;
        &lt;/trigger&gt;
        &lt;listOfEventAssignments&gt;
          <eventAssignment
variable="mw7d178aae_ff78_412a_830f_3be325d22773">
            &lt;math xmlns="http://www.w3.org/1998/Math/MathML"&gt;
              &lt;cn type="integer"&gt; 0 &lt;/cn&gt;
            &lt;/math&gt;
          &lt;/eventAssignment&gt;
        &lt;/listOfEventAssignments&gt;
      &lt;/event&gt;
      <event id="mw0e7c3cb5_bfa0_494f_b0f6_962c66c082f3"
name="event_154">
        &lt;trigger&gt;
          &lt;math xmlns="http://www.w3.org/1998/Math/MathML"&gt;
            &lt;apply&gt;
              &lt;geq/&gt;
              <csymbol encoding="text"
definitionURL="http://www.sbml.org/sbml/symbols/time"> time &lt;/csymbol&gt;
              &lt;cn&gt; 154.8 &lt;/cn&gt;
            &lt;/apply&gt;
          &lt;/math&gt;
        &lt;/trigger&gt;
```

```
        <listOfEventAssignments>
          <eventAssignment
variable="mw7d178aae_ff78_412a_830f_3be325d22773">
            <math xmlns="http://www.w3.org/1998/Math/MathML">
              <cn type="integer"> 1 </cn>
            </math>
          </eventAssignment>
        </listOfEventAssignments>
      </event>
      <event id="mw893fe7de_e30d_477e_a1ab_06271610b170"
name="event_155">
        <trigger>
          <math xmlns="http://www.w3.org/1998/Math/MathML">
            <apply>
              <geq/>
              <csymbol encoding="text"
definitionURL="http://www.sbml.org/sbml/symbols/time"> time </csymbol>
              <cn> 157.67 </cn>
            </apply>
          </math>
        </trigger>
        <listOfEventAssignments>
          <eventAssignment
variable="mw7d178aae_ff78_412a_830f_3be325d22773">
            <math xmlns="http://www.w3.org/1998/Math/MathML">
              <cn type="integer"> 0 </cn>
            </math>
          </eventAssignment>
        </listOfEventAssignments>
      </event>
      <event id="mwb9df38ca_a72c_4968_b70a_2585eb7e9e2f"
name="event_156">
        <trigger>
          <math xmlns="http://www.w3.org/1998/Math/MathML">
            <apply>
              <geq/>
              <csymbol encoding="text"
definitionURL="http://www.sbml.org/sbml/symbols/time"> time </csymbol>
              <cn> 159.1 </cn>
            </apply>
          </math>
        </trigger>
        <listOfEventAssignments>
          <eventAssignment
variable="mw7d178aae_ff78_412a_830f_3be325d22773">
            <math xmlns="http://www.w3.org/1998/Math/MathML">
              <cn type="integer"> 1 </cn>
            </math>
          </eventAssignment>
        </listOfEventAssignments>
      </event>
```

```
      <event id="mw989bc174_85a6_441f_b918_bdeced4c8083"
name="event_157">
        &lt;trigger&gt;
          &lt;math xmlns="http://www.w3.org/1998/Math/MathML"&gt;
            &lt;apply&gt;
              &lt;geq/&gt;
              <csymbol encoding="text"
definitionURL="http://www.sbml.org/sbml/symbols/time"> time &lt;/csymbol&gt;
              &lt;cn&gt; 161.97 &lt;/cn&gt;
            &lt;/apply&gt;
          &lt;/math&gt;
        &lt;/trigger&gt;
        &lt;listOfEventAssignments&gt;
          <eventAssignment
variable="mw7d178aae_ff78_412a_830f_3be325d22773">
            &lt;math xmlns="http://www.w3.org/1998/Math/MathML"&gt;
              &lt;cn type="integer"&gt; 0 &lt;/cn&gt;
            &lt;/math&gt;
          &lt;/eventAssignment&gt;
        &lt;/listOfEventAssignments&gt;
      &lt;/event&gt;
      <event id="mw1888f2b1_7f07_4be2_846b_88c7e8017efc"
name="event_158">
        &lt;trigger&gt;
          &lt;math xmlns="http://www.w3.org/1998/Math/MathML"&gt;
            &lt;apply&gt;
              &lt;geq/&gt;
              <csymbol encoding="text"
definitionURL="http://www.sbml.org/sbml/symbols/time"> time &lt;/csymbol&gt;
              &lt;cn&gt; 163.4 &lt;/cn&gt;
            &lt;/apply&gt;
          &lt;/math&gt;
        &lt;/trigger&gt;
        &lt;listOfEventAssignments&gt;
          <eventAssignment
variable="mw7d178aae_ff78_412a_830f_3be325d22773">
            &lt;math xmlns="http://www.w3.org/1998/Math/MathML"&gt;
              &lt;cn type="integer"&gt; 1 &lt;/cn&gt;
            &lt;/math&gt;
          &lt;/eventAssignment&gt;
        &lt;/listOfEventAssignments&gt;
      &lt;/event&gt;
      <event id="mwb21a0426_d729_47cd_9dbc_3355c4b0cbe8"
name="event_159">
        &lt;trigger&gt;
          &lt;math xmlns="http://www.w3.org/1998/Math/MathML"&gt;
            &lt;apply&gt;
              &lt;geq/&gt;
              <csymbol encoding="text"
definitionURL="http://www.sbml.org/sbml/symbols/time"> time &lt;/csymbol&gt;
              &lt;cn&gt; 166.27 &lt;/cn&gt;
            &lt;/apply&gt;
```

```
          </math>
        </trigger>
        <listOfEventAssignments>
          <eventAssignment
variable="mw7d178aae_ff78_412a_830f_3be325d22773">
            <math xmlns="http://www.w3.org/1998/Math/MathML">
              <cn type="integer"> 0 </cn>
            </math>
          </eventAssignment>
        </listOfEventAssignments>
      </event>
      <event id="mwbe3b80c8_c29a_4520_a7fb_66553c215c28"
name="event_160">
        <trigger>
          <math xmlns="http://www.w3.org/1998/Math/MathML">
            <apply>
              <geq/>
              <csymbol encoding="text"
definitionURL="http://www.sbml.org/sbml/symbols/time"> time </csymbol>
              <cn> 167.7 </cn>
            </apply>
          </math>
        </trigger>
        <listOfEventAssignments>
          <eventAssignment
variable="mw7d178aae_ff78_412a_830f_3be325d22773">
            <math xmlns="http://www.w3.org/1998/Math/MathML">
              <cn type="integer"> 1 </cn>
            </math>
          </eventAssignment>
        </listOfEventAssignments>
      </event>
      <event id="mwb86937d4_d5f8_40a7_b3e2_ea77afd468bc"
name="event_161">
        <trigger>
          <math xmlns="http://www.w3.org/1998/Math/MathML">
            <apply>
              <geq/>
              <csymbol encoding="text"
definitionURL="http://www.sbml.org/sbml/symbols/time"> time </csymbol>
              <cn> 170.57 </cn>
            </apply>
          </math>
        </trigger>
        <listOfEventAssignments>
          <eventAssignment
variable="mw7d178aae_ff78_412a_830f_3be325d22773">
            <math xmlns="http://www.w3.org/1998/Math/MathML">
              <cn type="integer"> 0 </cn>
            </math>
          </eventAssignment>
        </listOfEventAssignments>
```

```
      &lt;/event&gt;
      <event id="mw301c213d_95f8_4f0c_babf_176260ba4cc1"
name="event_162">
        &lt;trigger&gt;
          &lt;math xmlns="http://www.w3.org/1998/Math/MathML"&gt;
            &lt;apply&gt;
              &lt;geq/&gt;
              <csymbol encoding="text"
definitionURL="http://www.sbml.org/sbml/symbols/time"> time &lt;/csymbol&gt;
              &lt;cn type="integer"&gt; 172 &lt;/cn&gt;
            &lt;/apply&gt;
          &lt;/math&gt;
        &lt;/trigger&gt;
        &lt;listOfEventAssignments&gt;
          <eventAssignment
variable="mw7d178aae_ff78_412a_830f_3be325d22773">
            &lt;math xmlns="http://www.w3.org/1998/Math/MathML"&gt;
              &lt;cn type="integer"&gt; 1 &lt;/cn&gt;
            &lt;/math&gt;
          &lt;/eventAssignment&gt;
        &lt;/listOfEventAssignments&gt;
      &lt;/event&gt;
      <event id="mw0461458b_684a_4c45_bbfd_2d3989c97017"
name="event_163">
        &lt;trigger&gt;
          &lt;math xmlns="http://www.w3.org/1998/Math/MathML"&gt;
            &lt;apply&gt;
              &lt;geq/&gt;
              <csymbol encoding="text"
definitionURL="http://www.sbml.org/sbml/symbols/time"> time &lt;/csymbol&gt;
              &lt;cn&gt; 174.87 &lt;/cn&gt;
            &lt;/apply&gt;
          &lt;/math&gt;
        &lt;/trigger&gt;
        &lt;listOfEventAssignments&gt;
          <eventAssignment
variable="mw7d178aae_ff78_412a_830f_3be325d22773">
            &lt;math xmlns="http://www.w3.org/1998/Math/MathML"&gt;
              &lt;cn type="integer"&gt; 0 &lt;/cn&gt;
            &lt;/math&gt;
          &lt;/eventAssignment&gt;
        &lt;/listOfEventAssignments&gt;
      &lt;/event&gt;
      <event id="mw6158a3f2_67a0_4f9b_8131_e4d0c612e84a"
name="event_164">
        &lt;trigger&gt;
          &lt;math xmlns="http://www.w3.org/1998/Math/MathML"&gt;
            &lt;apply&gt;
              &lt;geq/&gt;
              <csymbol encoding="text"
definitionURL="http://www.sbml.org/sbml/symbols/time"> time &lt;/csymbol&gt;
              &lt;cn&gt; 176.3 &lt;/cn&gt;
```

```
            &lt;/apply&gt;
          &lt;/math&gt;
        &lt;/trigger&gt;
        &lt;listOfEventAssignments&gt;
          <eventAssignment
variable="mw7d178aae_ff78_412a_830f_3be325d22773">
            &lt;math xmlns="http://www.w3.org/1998/Math/MathML"&gt;
              &lt;cn type="integer"&gt; 1 &lt;/cn&gt;
            &lt;/math&gt;
          &lt;/eventAssignment&gt;
        &lt;/listOfEventAssignments&gt;
      &lt;/event&gt;
      <event id="mwa2b2242e_efcf_4862_874d_73dfb0d1354c"
name="event_165">
        &lt;trigger&gt;
          &lt;math xmlns="http://www.w3.org/1998/Math/MathML"&gt;
            &lt;apply&gt;
              &lt;geq/&gt;
              <csymbol encoding="text"
definitionURL="http://www.sbml.org/sbml/symbols/time"> time &lt;/csymbol&gt;
              &lt;cn&gt; 179.17 &lt;/cn&gt;
            &lt;/apply&gt;
          &lt;/math&gt;
        &lt;/trigger&gt;
        &lt;listOfEventAssignments&gt;
          <eventAssignment
variable="mw7d178aae_ff78_412a_830f_3be325d22773">
            &lt;math xmlns="http://www.w3.org/1998/Math/MathML"&gt;
              &lt;cn type="integer"&gt; 0 &lt;/cn&gt;
            &lt;/math&gt;
          &lt;/eventAssignment&gt;
        &lt;/listOfEventAssignments&gt;
      &lt;/event&gt;
      <event id="mw5949312b_ccc0_4661_8e99_b3d3f3fc295d"
name="event_166">
        &lt;trigger&gt;
          &lt;math xmlns="http://www.w3.org/1998/Math/MathML"&gt;
            &lt;apply&gt;
              &lt;geq/&gt;
              <csymbol encoding="text"
definitionURL="http://www.sbml.org/sbml/symbols/time"> time &lt;/csymbol&gt;
              &lt;cn&gt; 179.17 &lt;/cn&gt;
            &lt;/apply&gt;
          &lt;/math&gt;
        &lt;/trigger&gt;
        &lt;listOfEventAssignments&gt;
          <eventAssignment
variable="mwcedbe49e_2d28_4720_8fcd_207db64228cf">
            &lt;math xmlns="http://www.w3.org/1998/Math/MathML"&gt;
              &lt;cn type="integer"&gt; 1 &lt;/cn&gt;
            &lt;/math&gt;
          &lt;/eventAssignment&gt;
```

```
        </listOfEventAssignments>
      </event>
      <event id="mw06aa407f_1c43_4d1e_a6f3_d733fa298eab"
name="event_167">
        <trigger>
          <math xmlns="http://www.w3.org/1998/Math/MathML">
            <apply>
              <geq/>
              <csymbol encoding="text"
definitionURL="http://www.sbml.org/sbml/symbols/time"> time </csymbol>
              <cn> 180.6 </cn>
            </apply>
          </math>
        </trigger>
        <listOfEventAssignments>
          <eventAssignment
variable="mwcedbe49e_2d28_4720_8fcd_207db64228cf">
            <math xmlns="http://www.w3.org/1998/Math/MathML">
              <cn type="integer"> 0 </cn>
            </math>
          </eventAssignment>
        </listOfEventAssignments>
      </event>
      <event id="mw1195eaae_5910_4715_a214_af5f86d3eb25"
name="event_168">
        <trigger>
          <math xmlns="http://www.w3.org/1998/Math/MathML">
            <apply>
              <geq/>
              <csymbol encoding="text"
definitionURL="http://www.sbml.org/sbml/symbols/time"> time </csymbol>
              <cn> 180.6 </cn>
            </apply>
          </math>
        </trigger>
        <listOfEventAssignments>
          <eventAssignment
variable="mw7d178aae_ff78_412a_830f_3be325d22773">
            <math xmlns="http://www.w3.org/1998/Math/MathML">
              <cn type="integer"> 1 </cn>
            </math>
          </eventAssignment>
        </listOfEventAssignments>
      </event>
      <event id="mw40a1c895_bcff_4d16_9b83_d201100e63a0"
name="event_169">
        <trigger>
          <math xmlns="http://www.w3.org/1998/Math/MathML">
            <apply>
              <geq/>
              <csymbol encoding="text"
definitionURL="http://www.sbml.org/sbml/symbols/time"> time </csymbol>
```

```
              &lt;cn&gt; 183.47 &lt;/cn&gt;
            &lt;/apply&gt;
          &lt;/math&gt;
        &lt;/trigger&gt;
        &lt;listOfEventAssignments&gt;
          <eventAssignment
variable="mw7d178aae_ff78_412a_830f_3be325d22773">
            &lt;math xmlns="http://www.w3.org/1998/Math/MathML"&gt;
              &lt;cn type="integer"&gt; 0 &lt;/cn&gt;
            &lt;/math&gt;
          &lt;/eventAssignment&gt;
        &lt;/listOfEventAssignments&gt;
      &lt;/event&gt;
      <event id="mwb80b7326_06ee_4af6_9dca_2978a628fef2"
name="event_170">
        &lt;trigger&gt;
          &lt;math xmlns="http://www.w3.org/1998/Math/MathML"&gt;
            &lt;apply&gt;
              &lt;geq/&gt;
              <csymbol encoding="text"
definitionURL="http://www.sbml.org/sbml/symbols/time"> time &lt;/csymbol&gt;
              &lt;cn&gt; 184.9 &lt;/cn&gt;
            &lt;/apply&gt;
          &lt;/math&gt;
        &lt;/trigger&gt;
        &lt;listOfEventAssignments&gt;
          <eventAssignment
variable="mw7d178aae_ff78_412a_830f_3be325d22773">
            &lt;math xmlns="http://www.w3.org/1998/Math/MathML"&gt;
              &lt;cn type="integer"&gt; 1 &lt;/cn&gt;
            &lt;/math&gt;
          &lt;/eventAssignment&gt;
        &lt;/listOfEventAssignments&gt;
      &lt;/event&gt;
      <event id="mw0f5bf79e_87b5_40a8_bd3f_5b6a5c327ba3"
name="event_171">
        &lt;trigger&gt;
          &lt;math xmlns="http://www.w3.org/1998/Math/MathML"&gt;
            &lt;apply&gt;
              &lt;geq/&gt;
              <csymbol encoding="text"
definitionURL="http://www.sbml.org/sbml/symbols/time"> time &lt;/csymbol&gt;
              &lt;cn&gt; 187.77 &lt;/cn&gt;
            &lt;/apply&gt;
          &lt;/math&gt;
        &lt;/trigger&gt;
        &lt;listOfEventAssignments&gt;
          <eventAssignment
variable="mw7d178aae_ff78_412a_830f_3be325d22773">
            &lt;math xmlns="http://www.w3.org/1998/Math/MathML"&gt;
              &lt;cn type="integer"&gt; 0 &lt;/cn&gt;
            &lt;/math&gt;
```

```
          </eventAssignment>
        </listOfEventAssignments>
      </event>
      <event id="mw26e5231b_8495_43f2_8e67_a153c8d4cbb0"
name="event_172">
        <trigger>
          <math xmlns="http://www.w3.org/1998/Math/MathML">
            <apply>
              <geq/>
              <csymbol encoding="text"
definitionURL="http://www.sbml.org/sbml/symbols/time"> time </csymbol>
              <cn> 189.2 </cn>
            </apply>
          </math>
        </trigger>
        <listOfEventAssignments>
          <eventAssignment
variable="mw7d178aae_ff78_412a_830f_3be325d22773">
            <math xmlns="http://www.w3.org/1998/Math/MathML">
              <cn type="integer"> 1 </cn>
            </math>
          </eventAssignment>
        </listOfEventAssignments>
      </event>
      <event id="mw295bf576_c16f_4f4a_b6fe_117229453fb5"
name="event_173">
        <trigger>
          <math xmlns="http://www.w3.org/1998/Math/MathML">
            <apply>
              <geq/>
              <csymbol encoding="text"
definitionURL="http://www.sbml.org/sbml/symbols/time"> time </csymbol>
              <cn> 192.07 </cn>
            </apply>
          </math>
        </trigger>
        <listOfEventAssignments>
          <eventAssignment
variable="mw7d178aae_ff78_412a_830f_3be325d22773">
            <math xmlns="http://www.w3.org/1998/Math/MathML">
              <cn type="integer"> 0 </cn>
            </math>
          </eventAssignment>
        </listOfEventAssignments>
      </event>
      <event id="mw8a3fcd8d_1a4a_4df8_887b_efc4cba7cb0b"
name="event_174">
        <trigger>
          <math xmlns="http://www.w3.org/1998/Math/MathML">
            <apply>
              <geq/>
```

```
              <csymbol encoding="text" 
definitionURL="http://www.sbml.org/sbml/symbols/time"> time &lt;/csymbol&gt;
              &lt;cn&gt; 193.5 &lt;/cn&gt;
            &lt;/apply&gt;
          &lt;/math&gt;
        &lt;/trigger&gt;
        &lt;listOfEventAssignments&gt;
          <eventAssignment 
variable="mw7d178aae_ff78_412a_830f_3be325d22773">
            &lt;math xmlns="http://www.w3.org/1998/Math/MathML"&gt;
              &lt;cn type="integer"&gt; 1 &lt;/cn&gt;
            &lt;/math&gt;
          &lt;/eventAssignment&gt;
        &lt;/listOfEventAssignments&gt;
      &lt;/event&gt;
      <event id="mw7df543fc_c9d2_47ef_a090_e339e6c33deb" 
name="event_175">
        &lt;trigger&gt;
          &lt;math xmlns="http://www.w3.org/1998/Math/MathML"&gt;
            &lt;apply&gt;
              &lt;geq/&gt;
              <csymbol encoding="text" 
definitionURL="http://www.sbml.org/sbml/symbols/time"> time &lt;/csymbol&gt;
              &lt;cn&gt; 196.37 &lt;/cn&gt;
            &lt;/apply&gt;
          &lt;/math&gt;
        &lt;/trigger&gt;
        &lt;listOfEventAssignments&gt;
          <eventAssignment 
variable="mw7d178aae_ff78_412a_830f_3be325d22773">
            &lt;math xmlns="http://www.w3.org/1998/Math/MathML"&gt;
              &lt;cn type="integer"&gt; 0 &lt;/cn&gt;
            &lt;/math&gt;
          &lt;/eventAssignment&gt;
        &lt;/listOfEventAssignments&gt;
      &lt;/event&gt;
      <event id="mw22fa5b5a_298a_484d_b872_88d21180b327" 
name="event_176">
        &lt;trigger&gt;
          &lt;math xmlns="http://www.w3.org/1998/Math/MathML"&gt;
            &lt;apply&gt;
              &lt;geq/&gt;
              <csymbol encoding="text" 
definitionURL="http://www.sbml.org/sbml/symbols/time"> time &lt;/csymbol&gt;
              &lt;cn&gt; 197.8 &lt;/cn&gt;
            &lt;/apply&gt;
          &lt;/math&gt;
        &lt;/trigger&gt;
        &lt;listOfEventAssignments&gt;
          <eventAssignment 
variable="mw7d178aae_ff78_412a_830f_3be325d22773">
            &lt;math xmlns="http://www.w3.org/1998/Math/MathML"&gt;
```

```
            &lt;cn type="integer"&gt; 1 &lt;/cn&gt;
          &lt;/math&gt;
        &lt;/eventAssignment&gt;
      &lt;/listOfEventAssignments&gt;
    &lt;/event&gt;
    <event id="mw547b8e2f_7e1d_419e_a26a_cad0b741230f"
name="event_177">
      &lt;trigger&gt;
        &lt;math xmlns="http://www.w3.org/1998/Math/MathML"&gt;
          &lt;apply&gt;
            &lt;geq/&gt;
            <csymbol encoding="text"
definitionURL="http://www.sbml.org/sbml/symbols/time"> time &lt;/csymbol&gt;
            &lt;cn&gt; 200.67 &lt;/cn&gt;
          &lt;/apply&gt;
        &lt;/math&gt;
      &lt;/trigger&gt;
      &lt;listOfEventAssignments&gt;
        <eventAssignment
variable="mw7d178aae_ff78_412a_830f_3be325d22773">
          &lt;math xmlns="http://www.w3.org/1998/Math/MathML"&gt;
            &lt;cn type="integer"&gt; 0 &lt;/cn&gt;
          &lt;/math&gt;
        &lt;/eventAssignment&gt;
      &lt;/listOfEventAssignments&gt;
    &lt;/event&gt;
    <event id="mw9b806d4b_e477_4903_9f6e_5507a058e4d2"
name="event_178">
      &lt;trigger&gt;
        &lt;math xmlns="http://www.w3.org/1998/Math/MathML"&gt;
          &lt;apply&gt;
            &lt;geq/&gt;
            <csymbol encoding="text"
definitionURL="http://www.sbml.org/sbml/symbols/time"> time &lt;/csymbol&gt;
            &lt;cn&gt; 183.47 &lt;/cn&gt;
          &lt;/apply&gt;
        &lt;/math&gt;
      &lt;/trigger&gt;
      &lt;listOfEventAssignments&gt;
        <eventAssignment
variable="mwcedbe49e_2d28_4720_8fcd_207db64228cf">
          &lt;math xmlns="http://www.w3.org/1998/Math/MathML"&gt;
            &lt;cn type="integer"&gt; 1 &lt;/cn&gt;
          &lt;/math&gt;
        &lt;/eventAssignment&gt;
      &lt;/listOfEventAssignments&gt;
    &lt;/event&gt;
    <event id="mw273c5fb2_70fb_4108_bf86_c4911455538c"
name="event_179">
      &lt;trigger&gt;
        &lt;math xmlns="http://www.w3.org/1998/Math/MathML"&gt;
          &lt;apply&gt;
```

```
              &lt;geq/&gt;
              <csymbol encoding="text"
definitionURL="http://www.sbml.org/sbml/symbols/time"> time &lt;/csymbol&gt;
              &lt;cn&gt; 184.9 &lt;/cn&gt;
            &lt;/apply&gt;
          &lt;/math&gt;
        &lt;/trigger&gt;
        &lt;listOfEventAssignments&gt;
          <eventAssignment
variable="mwcedbe49e_2d28_4720_8fcd_207db64228cf">
            &lt;math xmlns="http://www.w3.org/1998/Math/MathML"&gt;
              &lt;cn type="integer"&gt; 0 &lt;/cn&gt;
            &lt;/math&gt;
          &lt;/eventAssignment&gt;
        &lt;/listOfEventAssignments&gt;
      &lt;/event&gt;
      <event id="mw6a0fa441_b00a_468e_a299_06153afa9523"
name="event_180">
        &lt;trigger&gt;
          &lt;math xmlns="http://www.w3.org/1998/Math/MathML"&gt;
            &lt;apply&gt;
              &lt;geq/&gt;
              <csymbol encoding="text"
definitionURL="http://www.sbml.org/sbml/symbols/time"> time &lt;/csymbol&gt;
              &lt;cn&gt; 187.77 &lt;/cn&gt;
            &lt;/apply&gt;
          &lt;/math&gt;
        &lt;/trigger&gt;
        &lt;listOfEventAssignments&gt;
          <eventAssignment
variable="mwcedbe49e_2d28_4720_8fcd_207db64228cf">
            &lt;math xmlns="http://www.w3.org/1998/Math/MathML"&gt;
              &lt;cn type="integer"&gt; 1 &lt;/cn&gt;
            &lt;/math&gt;
          &lt;/eventAssignment&gt;
        &lt;/listOfEventAssignments&gt;
      &lt;/event&gt;
      <event id="mwe0d13dc4_03ba_464f_b466_ddae133d5416"
name="event_181">
        &lt;trigger&gt;
          &lt;math xmlns="http://www.w3.org/1998/Math/MathML"&gt;
            &lt;apply&gt;
              &lt;geq/&gt;
              <csymbol encoding="text"
definitionURL="http://www.sbml.org/sbml/symbols/time"> time &lt;/csymbol&gt;
              &lt;cn&gt; 189.2 &lt;/cn&gt;
            &lt;/apply&gt;
          &lt;/math&gt;
        &lt;/trigger&gt;
        &lt;listOfEventAssignments&gt;
          <eventAssignment
variable="mwcedbe49e_2d28_4720_8fcd_207db64228cf">
```

```
            <math xmlns="http://www.w3.org/1998/Math/MathML">
              <cn type="integer"> 0 </cn>
            </math>
          </eventAssignment>
        </listOfEventAssignments>
      </event>
      <event id="mw60f8a984_f44b_420c_939a_ac72f7b223d5"
name="event_182">
        <trigger>
          <math xmlns="http://www.w3.org/1998/Math/MathML">
            <apply>
              <geq/>
              <csymbol encoding="text"
definitionURL="http://www.sbml.org/sbml/symbols/time"> time </csymbol>
              <cn> 192.07 </cn>
            </apply>
          </math>
        </trigger>
        <listOfEventAssignments>
          <eventAssignment
variable="mwcedbe49e_2d28_4720_8fcd_207db64228cf">
            <math xmlns="http://www.w3.org/1998/Math/MathML">
              <cn type="integer"> 1 </cn>
            </math>
          </eventAssignment>
        </listOfEventAssignments>
      </event>
      <event id="mw254f925f_c0e2_4966_87a2_6572e6f24289"
name="event_183">
        <trigger>
          <math xmlns="http://www.w3.org/1998/Math/MathML">
            <apply>
              <geq/>
              <csymbol encoding="text"
definitionURL="http://www.sbml.org/sbml/symbols/time"> time </csymbol>
              <cn> 193.5 </cn>
            </apply>
          </math>
        </trigger>
        <listOfEventAssignments>
          <eventAssignment
variable="mwcedbe49e_2d28_4720_8fcd_207db64228cf">
            <math xmlns="http://www.w3.org/1998/Math/MathML">
              <cn type="integer"> 0 </cn>
            </math>
          </eventAssignment>
        </listOfEventAssignments>
      </event>
      <event id="mw8993bc6f_5c7e_4a73_8302_ff13a05c5846"
name="event_184">
        <trigger>
          <math xmlns="http://www.w3.org/1998/Math/MathML">
```

```
            <apply>
              <geq/>
              <csymbol encoding="text"
definitionURL="http://www.sbml.org/sbml/symbols/time"> time </csymbol>
              <cn> 196.37 </cn>
            </apply>
          </math>
        </trigger>
        <listOfEventAssignments>
          <eventAssignment
variable="mwcedbe49e_2d28_4720_8fcd_207db64228cf">
            <math xmlns="http://www.w3.org/1998/Math/MathML">
              <cn type="integer"> 1 </cn>
            </math>
          </eventAssignment>
        </listOfEventAssignments>
      </event>
      <event id="mw317c548e_6df3_4662_8561_49b1262486e1"
name="event_185">
        <trigger>
          <math xmlns="http://www.w3.org/1998/Math/MathML">
            <apply>
              <geq/>
              <csymbol encoding="text"
definitionURL="http://www.sbml.org/sbml/symbols/time"> time </csymbol>
              <cn> 197.8 </cn>
            </apply>
          </math>
        </trigger>
        <listOfEventAssignments>
          <eventAssignment
variable="mwcedbe49e_2d28_4720_8fcd_207db64228cf">
            <math xmlns="http://www.w3.org/1998/Math/MathML">
              <cn type="integer"> 0 </cn>
            </math>
          </eventAssignment>
        </listOfEventAssignments>
      </event>
      <event id="mw3080906f_68af_42b8_b31b_e62615fff72f"
name="event_186">
        <trigger>
          <math xmlns="http://www.w3.org/1998/Math/MathML">
            <apply>
              <geq/>
              <csymbol encoding="text"
definitionURL="http://www.sbml.org/sbml/symbols/time"> time </csymbol>
              <cn> 200.67 </cn>
            </apply>
          </math>
        </trigger>
        <listOfEventAssignments>
```

```
          <eventAssignment
variable="mwcedbe49e_2d28_4720_8fcd_207db64228cf">
            &lt;math xmlns="http://www.w3.org/1998/Math/MathML"&gt;
              &lt;cn type="integer"&gt; 1 &lt;/cn&gt;
            &lt;/math&gt;
          &lt;/eventAssignment&gt;
        &lt;/listOfEventAssignments&gt;
      &lt;/event&gt;
      <event id="mw9544518f_9bfe_4666_937e_6593f36c50ae"
name="event_187">
        &lt;trigger&gt;
          &lt;math xmlns="http://www.w3.org/1998/Math/MathML"&gt;
            &lt;apply&gt;
              &lt;geq/&gt;
              <csymbol encoding="text"
definitionURL="http://www.sbml.org/sbml/symbols/time"> time &lt;/csymbol&gt;
              &lt;cn&gt; 202.1 &lt;/cn&gt;
            &lt;/apply&gt;
          &lt;/math&gt;
        &lt;/trigger&gt;
        &lt;listOfEventAssignments&gt;
          <eventAssignment
variable="mwcedbe49e_2d28_4720_8fcd_207db64228cf">
            &lt;math xmlns="http://www.w3.org/1998/Math/MathML"&gt;
              &lt;cn type="integer"&gt; 0 &lt;/cn&gt;
            &lt;/math&gt;
          &lt;/eventAssignment&gt;
        &lt;/listOfEventAssignments&gt;
      &lt;/event&gt;
      <event id="mw8dd71d02_9291_4c52_9ec2_8d389767aebd"
name="event_188">
        &lt;trigger&gt;
          &lt;math xmlns="http://www.w3.org/1998/Math/MathML"&gt;
            &lt;apply&gt;
              &lt;geq/&gt;
              <csymbol encoding="text"
definitionURL="http://www.sbml.org/sbml/symbols/time"> time &lt;/csymbol&gt;
              &lt;cn&gt; 202.1 &lt;/cn&gt;
            &lt;/apply&gt;
          &lt;/math&gt;
        &lt;/trigger&gt;
        &lt;listOfEventAssignments&gt;
          <eventAssignment
variable="mw7d178aae_ff78_412a_830f_3be325d22773">
            &lt;math xmlns="http://www.w3.org/1998/Math/MathML"&gt;
              &lt;cn type="integer"&gt; 1 &lt;/cn&gt;
            &lt;/math&gt;
          &lt;/eventAssignment&gt;
        &lt;/listOfEventAssignments&gt;
      &lt;/event&gt;
      <event id="mweaa858fc_f3a0_4d3a_a1fc_99fbf850cfe7"
name="event_189">
```

```
        <trigger>
          <math xmlns="http://www.w3.org/1998/Math/MathML">
            <apply>
              <geq/>
              <csymbol encoding="text"
definitionURL="http://www.sbml.org/sbml/symbols/time"> time </csymbol>
              <cn> 204.97 </cn>
            </apply>
          </math>
        </trigger>
        <listOfEventAssignments>
          <eventAssignment
variable="mw7d178aae_ff78_412a_830f_3be325d22773">
            <math xmlns="http://www.w3.org/1998/Math/MathML">
              <cn type="integer"> 0 </cn>
            </math>
          </eventAssignment>
        </listOfEventAssignments>
      </event>
      <event id="mw6ef43e3b_a50b_4c51_bd62_502ec508d18b"
name="event_190">
        <trigger>
          <math xmlns="http://www.w3.org/1998/Math/MathML">
            <apply>
              <geq/>
              <csymbol encoding="text"
definitionURL="http://www.sbml.org/sbml/symbols/time"> time </csymbol>
              <cn> 206.4 </cn>
            </apply>
          </math>
        </trigger>
        <listOfEventAssignments>
          <eventAssignment
variable="mw7d178aae_ff78_412a_830f_3be325d22773">
            <math xmlns="http://www.w3.org/1998/Math/MathML">
              <cn type="integer"> 1 </cn>
            </math>
          </eventAssignment>
        </listOfEventAssignments>
      </event>
      <event id="mwf18efbf8_0983_4893_8730_20c49817aac8"
name="event_191">
        <trigger>
          <math xmlns="http://www.w3.org/1998/Math/MathML">
            <apply>
              <geq/>
              <csymbol encoding="text"
definitionURL="http://www.sbml.org/sbml/symbols/time"> time </csymbol>
              <cn> 209.27 </cn>
            </apply>
          </math>
        </trigger>
```

```
        <listOfEventAssignments>
          <eventAssignment
variable="mw7d178aae_ff78_412a_830f_3be325d22773">
            <math xmlns="http://www.w3.org/1998/Math/MathML">
              <cn type="integer"> 0 </cn>
            </math>
          </eventAssignment>
        </listOfEventAssignments>
      </event>
      <event id="mw99071009_7980_49fb_a950_d5414f832f6d"
name="event_192">
        <trigger>
          <math xmlns="http://www.w3.org/1998/Math/MathML">
            <apply>
              <geq/>
              <csymbol encoding="text"
definitionURL="http://www.sbml.org/sbml/symbols/time"> time </csymbol>
              <cn> 210.7 </cn>
            </apply>
          </math>
        </trigger>
        <listOfEventAssignments>
          <eventAssignment
variable="mw7d178aae_ff78_412a_830f_3be325d22773">
            <math xmlns="http://www.w3.org/1998/Math/MathML">
              <cn type="integer"> 1 </cn>
            </math>
          </eventAssignment>
        </listOfEventAssignments>
      </event>
      <event id="mw154ff8d2_b0a6_46a3_bcec_5d492f350133"
name="event_193">
        <trigger>
          <math xmlns="http://www.w3.org/1998/Math/MathML">
            <apply>
              <geq/>
              <csymbol encoding="text"
definitionURL="http://www.sbml.org/sbml/symbols/time"> time </csymbol>
              <cn> 213.57 </cn>
            </apply>
          </math>
        </trigger>
        <listOfEventAssignments>
          <eventAssignment
variable="mw7d178aae_ff78_412a_830f_3be325d22773">
            <math xmlns="http://www.w3.org/1998/Math/MathML">
              <cn type="integer"> 0 </cn>
            </math>
          </eventAssignment>
        </listOfEventAssignments>
      </event>
```

```
      <event id="mw3767d747_38df_4b5f_a47e_034faf838a2b"
name="event_194">
        <trigger>
          <math xmlns="http://www.w3.org/1998/Math/MathML">
            <apply>
              <geq/>
              <csymbol encoding="text"
definitionURL="http://www.sbml.org/sbml/symbols/time"> time </csymbol>
              <cn type="integer"> 215 </cn>
            </apply>
          </math>
        </trigger>
        <listOfEventAssignments>
          <eventAssignment
variable="mw7d178aae_ff78_412a_830f_3be325d22773">
            <math xmlns="http://www.w3.org/1998/Math/MathML">
              <cn type="integer"> 1 </cn>
            </math>
          </eventAssignment>
        </listOfEventAssignments>
      </event>
      <event id="mwce026865_e892_4e58_94c9_92d515600752"
name="event_195">
        <trigger>
          <math xmlns="http://www.w3.org/1998/Math/MathML">
            <apply>
              <geq/>
              <csymbol encoding="text"
definitionURL="http://www.sbml.org/sbml/symbols/time"> time </csymbol>
              <cn> 217.87 </cn>
            </apply>
          </math>
        </trigger>
        <listOfEventAssignments>
          <eventAssignment
variable="mw7d178aae_ff78_412a_830f_3be325d22773">
            <math xmlns="http://www.w3.org/1998/Math/MathML">
              <cn type="integer"> 0 </cn>
            </math>
          </eventAssignment>
        </listOfEventAssignments>
      </event>
      <event id="mwf853dbd8_cc92_45b5_b713_bbaaa3338ce3"
name="event_196">
        <trigger>
          <math xmlns="http://www.w3.org/1998/Math/MathML">
            <apply>
              <geq/>
              <csymbol encoding="text"
definitionURL="http://www.sbml.org/sbml/symbols/time"> time </csymbol>
              <cn> 219.3 </cn>
            </apply>
```

```
          </math>
        </trigger>
        <listOfEventAssignments>
          <eventAssignment
variable="mw7d178aae_ff78_412a_830f_3be325d22773">
            <math xmlns="http://www.w3.org/1998/Math/MathML">
              <cn type="integer"> 1 </cn>
            </math>
          </eventAssignment>
        </listOfEventAssignments>
      </event>
      <event id="mwc7378ea0_3e75_4224_8e45_463784514ef9"
name="event_197">
        <trigger>
          <math xmlns="http://www.w3.org/1998/Math/MathML">
            <apply>
              <geq/>
              <csymbol encoding="text"
definitionURL="http://www.sbml.org/sbml/symbols/time"> time </csymbol>
              <cn> 222.17 </cn>
            </apply>
          </math>
        </trigger>
        <listOfEventAssignments>
          <eventAssignment
variable="mw7d178aae_ff78_412a_830f_3be325d22773">
            <math xmlns="http://www.w3.org/1998/Math/MathML">
              <cn type="integer"> 0 </cn>
            </math>
          </eventAssignment>
        </listOfEventAssignments>
      </event>
      <event id="mwa0f507c1_0a5a_4e62_b434_c1b10367ac5d"
name="event_198">
        <trigger>
          <math xmlns="http://www.w3.org/1998/Math/MathML">
            <apply>
              <geq/>
              <csymbol encoding="text"
definitionURL="http://www.sbml.org/sbml/symbols/time"> time </csymbol>
              <cn> 223.6 </cn>
            </apply>
          </math>
        </trigger>
        <listOfEventAssignments>
          <eventAssignment
variable="mw7d178aae_ff78_412a_830f_3be325d22773">
            <math xmlns="http://www.w3.org/1998/Math/MathML">
              <cn type="integer"> 1 </cn>
            </math>
          </eventAssignment>
        </listOfEventAssignments>
```

```
      &lt;/event&gt;
      <event id="mw766e0d5f_b7f0_4e09_aba3_e0574e4bee63"
name="event_199">
        &lt;trigger&gt;
          &lt;math xmlns="http://www.w3.org/1998/Math/MathML"&gt;
            &lt;apply&gt;
              &lt;geq/&gt;
              <csymbol encoding="text"
definitionURL="http://www.sbml.org/sbml/symbols/time"> time &lt;/csymbol&gt;
              &lt;cn&gt; 226.47 &lt;/cn&gt;
            &lt;/apply&gt;
          &lt;/math&gt;
        &lt;/trigger&gt;
        &lt;listOfEventAssignments&gt;
          <eventAssignment
variable="mw7d178aae_ff78_412a_830f_3be325d22773">
            &lt;math xmlns="http://www.w3.org/1998/Math/MathML"&gt;
              &lt;cn type="integer"&gt; 0 &lt;/cn&gt;
            &lt;/math&gt;
          &lt;/eventAssignment&gt;
        &lt;/listOfEventAssignments&gt;
      &lt;/event&gt;
      <event id="mwc7555e51_c078_4429_b8ac_219ceca78a7a"
name="event_200">
        &lt;trigger&gt;
          &lt;math xmlns="http://www.w3.org/1998/Math/MathML"&gt;
            &lt;apply&gt;
              &lt;geq/&gt;
              <csymbol encoding="text"
definitionURL="http://www.sbml.org/sbml/symbols/time"> time &lt;/csymbol&gt;
              &lt;cn&gt; 227.9 &lt;/cn&gt;
            &lt;/apply&gt;
          &lt;/math&gt;
        &lt;/trigger&gt;
        &lt;listOfEventAssignments&gt;
          <eventAssignment
variable="mw7d178aae_ff78_412a_830f_3be325d22773">
            &lt;math xmlns="http://www.w3.org/1998/Math/MathML"&gt;
              &lt;cn type="integer"&gt; 1 &lt;/cn&gt;
            &lt;/math&gt;
          &lt;/eventAssignment&gt;
        &lt;/listOfEventAssignments&gt;
      &lt;/event&gt;
      <event id="mwb28e70f4_3d3a_40bc_ada1_303b5d5d2142"
name="event_201">
        &lt;trigger&gt;
          &lt;math xmlns="http://www.w3.org/1998/Math/MathML"&gt;
            &lt;apply&gt;
              &lt;geq/&gt;
              <csymbol encoding="text"
definitionURL="http://www.sbml.org/sbml/symbols/time"> time &lt;/csymbol&gt;
              &lt;cn&gt; 230.77 &lt;/cn&gt;
```

```
          </apply>
        </math>
      </trigger>
      <listOfEventAssignments>
        <eventAssignment
variable="mw7d178aae_ff78_412a_830f_3be325d22773">
          <math xmlns="http://www.w3.org/1998/Math/MathML">
            <cn type="integer"> 0 </cn>
          </math>
        </eventAssignment>
      </listOfEventAssignments>
    </event>
    <event id="mw0dfd2fe1_f412_4756_9671_73db83f40546"
name="event_202">
      <trigger>
        <math xmlns="http://www.w3.org/1998/Math/MathML">
          <apply>
            <geq/>
            <csymbol encoding="text"
definitionURL="http://www.sbml.org/sbml/symbols/time"> time </csymbol>
            <cn> 232.2 </cn>
          </apply>
        </math>
      </trigger>
      <listOfEventAssignments>
        <eventAssignment
variable="mw7d178aae_ff78_412a_830f_3be325d22773">
          <math xmlns="http://www.w3.org/1998/Math/MathML">
            <cn type="integer"> 1 </cn>
          </math>
        </eventAssignment>
      </listOfEventAssignments>
    </event>
    <event id="mw4b0ae136_db71_4aed_9b40_fe8f44e6338d"
name="event_203">
      <trigger>
        <math xmlns="http://www.w3.org/1998/Math/MathML">
          <apply>
            <geq/>
            <csymbol encoding="text"
definitionURL="http://www.sbml.org/sbml/symbols/time"> time </csymbol>
            <cn> 235.07 </cn>
          </apply>
        </math>
      </trigger>
      <listOfEventAssignments>
        <eventAssignment
variable="mw7d178aae_ff78_412a_830f_3be325d22773">
          <math xmlns="http://www.w3.org/1998/Math/MathML">
            <cn type="integer"> 0 </cn>
          </math>
        </eventAssignment>
```

```
        &lt;/listOfEventAssignments&gt;
      &lt;/event&gt;
      <event id="mw801c1de1_296b_41b5_b891_f24783ecf23e"
name="event_204">
        &lt;trigger&gt;
          &lt;math xmlns="http://www.w3.org/1998/Math/MathML"&gt;
            &lt;apply&gt;
              &lt;geq/&gt;
              <csymbol encoding="text"
definitionURL="http://www.sbml.org/sbml/symbols/time"> time &lt;/csymbol&gt;
              &lt;cn&gt; 236.5 &lt;/cn&gt;
            &lt;/apply&gt;
          &lt;/math&gt;
        &lt;/trigger&gt;
        &lt;listOfEventAssignments&gt;
          <eventAssignment
variable="mw7d178aae_ff78_412a_830f_3be325d22773">
            &lt;math xmlns="http://www.w3.org/1998/Math/MathML"&gt;
              &lt;cn type="integer"&gt; 1 &lt;/cn&gt;
            &lt;/math&gt;
          &lt;/eventAssignment&gt;
        &lt;/listOfEventAssignments&gt;
      &lt;/event&gt;
      <event id="mw2fd929ac_c63c_4293_b12b_65e40b3469d6"
name="event_205">
        &lt;trigger&gt;
          &lt;math xmlns="http://www.w3.org/1998/Math/MathML"&gt;
            &lt;apply&gt;
              &lt;geq/&gt;
              <csymbol encoding="text"
definitionURL="http://www.sbml.org/sbml/symbols/time"> time &lt;/csymbol&gt;
              &lt;cn&gt; 239.37 &lt;/cn&gt;
            &lt;/apply&gt;
          &lt;/math&gt;
        &lt;/trigger&gt;
        &lt;listOfEventAssignments&gt;
          <eventAssignment
variable="mw7d178aae_ff78_412a_830f_3be325d22773">
            &lt;math xmlns="http://www.w3.org/1998/Math/MathML"&gt;
              &lt;cn type="integer"&gt; 0 &lt;/cn&gt;
            &lt;/math&gt;
          &lt;/eventAssignment&gt;
        &lt;/listOfEventAssignments&gt;
      &lt;/event&gt;
      <event id="mw885b4bbd_5324_4a75_a9c1_73ee9f6ca738"
name="event_206">
        &lt;trigger&gt;
          &lt;math xmlns="http://www.w3.org/1998/Math/MathML"&gt;
            &lt;apply&gt;
              &lt;geq/&gt;
              <csymbol encoding="text"
definitionURL="http://www.sbml.org/sbml/symbols/time"> time &lt;/csymbol&gt;
```

```
            <cn> 240.8 </cn>
          </apply>
        </math>
      </trigger>
      <listOfEventAssignments>
        <eventAssignment
variable="mw7d178aae_ff78_412a_830f_3be325d22773">
          <math xmlns="http://www.w3.org/1998/Math/MathML">
            <cn type="integer"> 1 </cn>
          </math>
        </eventAssignment>
      </listOfEventAssignments>
    </event>
    <event id="mwd869da93_bb11_463c_a46f_90730a21104e"
name="event_207">
      <trigger>
        <math xmlns="http://www.w3.org/1998/Math/MathML">
          <apply>
            <geq/>
            <csymbol encoding="text"
definitionURL="http://www.sbml.org/sbml/symbols/time"> time </csymbol>
            <cn> 243.67 </cn>
          </apply>
        </math>
      </trigger>
      <listOfEventAssignments>
        <eventAssignment
variable="mw7d178aae_ff78_412a_830f_3be325d22773">
          <math xmlns="http://www.w3.org/1998/Math/MathML">
            <cn type="integer"> 0 </cn>
          </math>
        </eventAssignment>
      </listOfEventAssignments>
    </event>
    <event id="mw2f7d1ab5_7c7c_4628_bf3e_ffd79c084da8"
name="event_208">
      <trigger>
        <math xmlns="http://www.w3.org/1998/Math/MathML">
          <apply>
            <geq/>
            <csymbol encoding="text"
definitionURL="http://www.sbml.org/sbml/symbols/time"> time </csymbol>
            <cn> 245.1 </cn>
          </apply>
        </math>
      </trigger>
      <listOfEventAssignments>
        <eventAssignment
variable="mw7d178aae_ff78_412a_830f_3be325d22773">
          <math xmlns="http://www.w3.org/1998/Math/MathML">
            <cn type="integer"> 1 </cn>
          </math>
```

```
          </eventAssignment>
        </listOfEventAssignments>
      </event>
      <event id="mw37969f0a_de40_4783_bcb7_c79b96401aea"
name="event_209">
        <trigger>
          <math xmlns="http://www.w3.org/1998/Math/MathML">
            <apply>
              <geq/>
              <csymbol encoding="text"
definitionURL="http://www.sbml.org/sbml/symbols/time"> time </csymbol>
              <cn> 204.97 </cn>
            </apply>
          </math>
        </trigger>
        <listOfEventAssignments>
          <eventAssignment
variable="mwcedbe49e_2d28_4720_8fcd_207db64228cf">
            <math xmlns="http://www.w3.org/1998/Math/MathML">
              <cn type="integer"> 1 </cn>
            </math>
          </eventAssignment>
        </listOfEventAssignments>
      </event>
      <event id="mw64dba7d3_004a_48b7_a498_5aa94feff241"
name="event_210">
        <trigger>
          <math xmlns="http://www.w3.org/1998/Math/MathML">
            <apply>
              <geq/>
              <csymbol encoding="text"
definitionURL="http://www.sbml.org/sbml/symbols/time"> time </csymbol>
              <cn> 206.4 </cn>
            </apply>
          </math>
        </trigger>
        <listOfEventAssignments>
          <eventAssignment
variable="mwcedbe49e_2d28_4720_8fcd_207db64228cf">
            <math xmlns="http://www.w3.org/1998/Math/MathML">
              <cn type="integer"> 0 </cn>
            </math>
          </eventAssignment>
        </listOfEventAssignments>
      </event>
      <event id="mw5b0b8386_22d7_4149_a51d_1ac7a9ef9de3"
name="event_211">
        <trigger>
          <math xmlns="http://www.w3.org/1998/Math/MathML">
            <apply>
              <geq/>
```

```
              <csymbol encoding="text"
definitionURL="http://www.sbml.org/sbml/symbols/time"> time </csymbol>
              <cn> 209.27 </cn>
            </apply>
          </math>
        </trigger>
        <listOfEventAssignments>
          <eventAssignment
variable="mwcedbe49e_2d28_4720_8fcd_207db64228cf">
            <math xmlns="http://www.w3.org/1998/Math/MathML">
              <cn type="integer"> 1 </cn>
            </math>
          </eventAssignment>
        </listOfEventAssignments>
      </event>
      <event id="mw15f8521b_2813_4666_ae59_f402c4651cd0"
name="event_212">
        <trigger>
          <math xmlns="http://www.w3.org/1998/Math/MathML">
            <apply>
              <geq/>
              <csymbol encoding="text"
definitionURL="http://www.sbml.org/sbml/symbols/time"> time </csymbol>
              <cn> 210.7 </cn>
            </apply>
          </math>
        </trigger>
        <listOfEventAssignments>
          <eventAssignment
variable="mwcedbe49e_2d28_4720_8fcd_207db64228cf">
            <math xmlns="http://www.w3.org/1998/Math/MathML">
              <cn type="integer"> 0 </cn>
            </math>
          </eventAssignment>
        </listOfEventAssignments>
      </event>
      <event id="mw4dd0b528_26b6_4673_a261_430e13f191fb"
name="event_213">
        <trigger>
          <math xmlns="http://www.w3.org/1998/Math/MathML">
            <apply>
              <geq/>
              <csymbol encoding="text"
definitionURL="http://www.sbml.org/sbml/symbols/time"> time </csymbol>
              <cn> 213.57 </cn>
            </apply>
          </math>
        </trigger>
        <listOfEventAssignments>
          <eventAssignment
variable="mwcedbe49e_2d28_4720_8fcd_207db64228cf">
            <math xmlns="http://www.w3.org/1998/Math/MathML">
```

```
              <cn type="integer"> 1 </cn>
            </math>
          </eventAssignment>
        </listOfEventAssignments>
      </event>
      <event id="mw510e06a4_8618_4ca6_b274_8511a3e14b42"
name="event_214">
        <trigger>
          <math xmlns="http://www.w3.org/1998/Math/MathML">
            <apply>
              <geq/>
              <csymbol encoding="text"
definitionURL="http://www.sbml.org/sbml/symbols/time"> time </csymbol>
              <cn type="integer"> 215 </cn>
            </apply>
          </math>
        </trigger>
        <listOfEventAssignments>
          <eventAssignment
variable="mwcedbe49e_2d28_4720_8fcd_207db64228cf">
            <math xmlns="http://www.w3.org/1998/Math/MathML">
              <cn type="integer"> 0 </cn>
            </math>
          </eventAssignment>
        </listOfEventAssignments>
      </event>
      <event id="mw296c9c80_9847_47a2_9b86_1b512f0988b4"
name="event_215">
        <trigger>
          <math xmlns="http://www.w3.org/1998/Math/MathML">
            <apply>
              <geq/>
              <csymbol encoding="text"
definitionURL="http://www.sbml.org/sbml/symbols/time"> time </csymbol>
              <cn> 217.87 </cn>
            </apply>
          </math>
        </trigger>
        <listOfEventAssignments>
          <eventAssignment
variable="mwcedbe49e_2d28_4720_8fcd_207db64228cf">
            <math xmlns="http://www.w3.org/1998/Math/MathML">
              <cn type="integer"> 1 </cn>
            </math>
          </eventAssignment>
        </listOfEventAssignments>
      </event>
      <event id="mwfda8f644_65ff_4a0d_b155_ae6af641e617"
name="event_216">
        <trigger>
          <math xmlns="http://www.w3.org/1998/Math/MathML">
            <apply>
```

```
              &lt;geq/&gt;
              <csymbol encoding="text"
definitionURL="http://www.sbml.org/sbml/symbols/time"> time &lt;/csymbol&gt;
              &lt;cn&gt; 219.3 &lt;/cn&gt;
            &lt;/apply&gt;
          &lt;/math&gt;
        &lt;/trigger&gt;
        &lt;listOfEventAssignments&gt;
          <eventAssignment
variable="mwcedbe49e_2d28_4720_8fcd_207db64228cf">
            &lt;math xmlns="http://www.w3.org/1998/Math/MathML"&gt;
              &lt;cn type="integer"&gt; 0 &lt;/cn&gt;
            &lt;/math&gt;
          &lt;/eventAssignment&gt;
        &lt;/listOfEventAssignments&gt;
      &lt;/event&gt;
      <event id="mwaa8e34cd_abea_4f91_b34b_a3fffebff55d"
name="event_217">
        &lt;trigger&gt;
          &lt;math xmlns="http://www.w3.org/1998/Math/MathML"&gt;
            &lt;apply&gt;
              &lt;geq/&gt;
              <csymbol encoding="text"
definitionURL="http://www.sbml.org/sbml/symbols/time"> time &lt;/csymbol&gt;
              &lt;cn&gt; 222.17 &lt;/cn&gt;
            &lt;/apply&gt;
          &lt;/math&gt;
        &lt;/trigger&gt;
        &lt;listOfEventAssignments&gt;
          <eventAssignment
variable="mwcedbe49e_2d28_4720_8fcd_207db64228cf">
            &lt;math xmlns="http://www.w3.org/1998/Math/MathML"&gt;
              &lt;cn type="integer"&gt; 1 &lt;/cn&gt;
            &lt;/math&gt;
          &lt;/eventAssignment&gt;
        &lt;/listOfEventAssignments&gt;
      &lt;/event&gt;
      <event id="mw59cb5cda_99c3_464c_a51a_f69571617328"
name="event_218">
        &lt;trigger&gt;
          &lt;math xmlns="http://www.w3.org/1998/Math/MathML"&gt;
            &lt;apply&gt;
              &lt;geq/&gt;
              <csymbol encoding="text"
definitionURL="http://www.sbml.org/sbml/symbols/time"> time &lt;/csymbol&gt;
              &lt;cn&gt; 223.6 &lt;/cn&gt;
            &lt;/apply&gt;
          &lt;/math&gt;
        &lt;/trigger&gt;
        &lt;listOfEventAssignments&gt;
          <eventAssignment
variable="mwcedbe49e_2d28_4720_8fcd_207db64228cf">
```

```
            <math xmlns="http://www.w3.org/1998/Math/MathML">
              <cn type="integer"> 0 </cn>
            </math>
          </eventAssignment>
        </listOfEventAssignments>
      </event>
      <event id="mw2c78e34d_14c8_4131_9295_885787e2ca59"
name="event_219">
        <trigger>
          <math xmlns="http://www.w3.org/1998/Math/MathML">
            <apply>
              <geq/>
              <csymbol encoding="text"
definitionURL="http://www.sbml.org/sbml/symbols/time"> time </csymbol>
              <cn> 226.47 </cn>
            </apply>
          </math>
        </trigger>
        <listOfEventAssignments>
          <eventAssignment
variable="mwcedbe49e_2d28_4720_8fcd_207db64228cf">
            <math xmlns="http://www.w3.org/1998/Math/MathML">
              <cn type="integer"> 1 </cn>
            </math>
          </eventAssignment>
        </listOfEventAssignments>
      </event>
      <event id="mwccd0d3df_ecf6_4d49_900c_fe04598e2d3f"
name="event_220">
        <trigger>
          <math xmlns="http://www.w3.org/1998/Math/MathML">
            <apply>
              <geq/>
              <csymbol encoding="text"
definitionURL="http://www.sbml.org/sbml/symbols/time"> time </csymbol>
              <cn> 227.9 </cn>
            </apply>
          </math>
        </trigger>
        <listOfEventAssignments>
          <eventAssignment
variable="mwcedbe49e_2d28_4720_8fcd_207db64228cf">
            <math xmlns="http://www.w3.org/1998/Math/MathML">
              <cn type="integer"> 0 </cn>
            </math>
          </eventAssignment>
        </listOfEventAssignments>
      </event>
      <event id="mw4403c997_db62_4d10_b9fb_489260538dd0"
name="event_221">
        <trigger>
          <math xmlns="http://www.w3.org/1998/Math/MathML">
```

```
            <apply>
              <geq/>
              <csymbol encoding="text"
definitionURL="http://www.sbml.org/sbml/symbols/time"> time </csymbol>
              <cn> 230.77 </cn>
            </apply>
          </math>
        </trigger>
        <listOfEventAssignments>
          <eventAssignment
variable="mwcedbe49e_2d28_4720_8fcd_207db64228cf">
            <math xmlns="http://www.w3.org/1998/Math/MathML">
              <cn type="integer"> 1 </cn>
            </math>
          </eventAssignment>
        </listOfEventAssignments>
      </event>
      <event id="mwd6ea5930_a8c5_453f_9bf2_78f21b4f70ca"
name="event_222">
        <trigger>
          <math xmlns="http://www.w3.org/1998/Math/MathML">
            <apply>
              <geq/>
              <csymbol encoding="text"
definitionURL="http://www.sbml.org/sbml/symbols/time"> time </csymbol>
              <cn> 232.2 </cn>
            </apply>
          </math>
        </trigger>
        <listOfEventAssignments>
          <eventAssignment
variable="mwcedbe49e_2d28_4720_8fcd_207db64228cf">
            <math xmlns="http://www.w3.org/1998/Math/MathML">
              <cn type="integer"> 0 </cn>
            </math>
          </eventAssignment>
        </listOfEventAssignments>
      </event>
      <event id="mwd4c8721d_b7ee_4e51_97f5_2a0c13c64b1d"
name="event_223">
        <trigger>
          <math xmlns="http://www.w3.org/1998/Math/MathML">
            <apply>
              <geq/>
              <csymbol encoding="text"
definitionURL="http://www.sbml.org/sbml/symbols/time"> time </csymbol>
              <cn> 235.07 </cn>
            </apply>
          </math>
        </trigger>
        <listOfEventAssignments>
```

```
          <eventAssignment
variable="mwcedbe49e_2d28_4720_8fcd_207db64228cf">
            <math xmlns="http://www.w3.org/1998/Math/MathML">
              <cn type="integer"> 1 </cn>
            </math>
          </eventAssignment>
        </listOfEventAssignments>
      </event>
      <event id="mw36c4058e_7ca6_4362_a640_b71c04c18bb3"
name="event_224">
        <trigger>
          <math xmlns="http://www.w3.org/1998/Math/MathML">
            <apply>
              <geq/>
              <csymbol encoding="text"
definitionURL="http://www.sbml.org/sbml/symbols/time"> time </csymbol>
              <cn> 236.5 </cn>
            </apply>
          </math>
        </trigger>
        <listOfEventAssignments>
          <eventAssignment
variable="mwcedbe49e_2d28_4720_8fcd_207db64228cf">
            <math xmlns="http://www.w3.org/1998/Math/MathML">
              <cn type="integer"> 0 </cn>
            </math>
          </eventAssignment>
        </listOfEventAssignments>
      </event>
      <event id="mw9c6de8b4_007e_4cc3_bee3_ae25e42c6bee"
name="event_225">
        <trigger>
          <math xmlns="http://www.w3.org/1998/Math/MathML">
            <apply>
              <geq/>
              <csymbol encoding="text"
definitionURL="http://www.sbml.org/sbml/symbols/time"> time </csymbol>
              <cn> 239.37 </cn>
            </apply>
          </math>
        </trigger>
        <listOfEventAssignments>
          <eventAssignment
variable="mwcedbe49e_2d28_4720_8fcd_207db64228cf">
            <math xmlns="http://www.w3.org/1998/Math/MathML">
              <cn type="integer"> 1 </cn>
            </math>
          </eventAssignment>
        </listOfEventAssignments>
      </event>
      <event id="mwa1bd6469_2025_454d_b402_97893ef8a825"
name="event_226">
```

```
        <trigger>
          <math xmlns="http://www.w3.org/1998/Math/MathML">
            <apply>
              <geq/>
              <csymbol encoding="text"
definitionURL="http://www.sbml.org/sbml/symbols/time"> time </csymbol>
              <cn> 240.8 </cn>
            </apply>
          </math>
        </trigger>
        <listOfEventAssignments>
          <eventAssignment
variable="mwcedbe49e_2d28_4720_8fcd_207db64228cf">
            <math xmlns="http://www.w3.org/1998/Math/MathML">
              <cn type="integer"> 0 </cn>
            </math>
          </eventAssignment>
        </listOfEventAssignments>
      </event>
      <event id="mwfe526f4d_c32d_4cd0_81ff_75dc8605648d"
name="event_227">
        <trigger>
          <math xmlns="http://www.w3.org/1998/Math/MathML">
            <apply>
              <geq/>
              <csymbol encoding="text"
definitionURL="http://www.sbml.org/sbml/symbols/time"> time </csymbol>
              <cn> 243.67 </cn>
            </apply>
          </math>
        </trigger>
        <listOfEventAssignments>
          <eventAssignment
variable="mwcedbe49e_2d28_4720_8fcd_207db64228cf">
            <math xmlns="http://www.w3.org/1998/Math/MathML">
              <cn type="integer"> 1 </cn>
            </math>
          </eventAssignment>
        </listOfEventAssignments>
      </event>
      <event id="mw2e514e7c_8d5c_4c92_ac3b_987b2cd5fd4e"
name="event_228">
        <trigger>
          <math xmlns="http://www.w3.org/1998/Math/MathML">
            <apply>
              <geq/>
              <csymbol encoding="text"
definitionURL="http://www.sbml.org/sbml/symbols/time"> time </csymbol>
              <cn> 245.1 </cn>
            </apply>
          </math>
        </trigger>
```

```
        <listOfEventAssignments>
          <eventAssignment
variable="mwcedbe49e_2d28_4720_8fcd_207db64228cf">
            <math xmlns="http://www.w3.org/1998/Math/MathML">
              <cn type="integer"> 0 </cn>
            </math>
          </eventAssignment>
        </listOfEventAssignments>
      </event>
      <event id="mwc240277c_3dc6_42ff_bbf0_54a37886f938"
name="event_229">
        <trigger>
          <math xmlns="http://www.w3.org/1998/Math/MathML">
            <apply>
              <geq/>
              <csymbol encoding="text"
definitionURL="http://www.sbml.org/sbml/symbols/time"> time </csymbol>
              <cn> 247.97 </cn>
            </apply>
          </math>
        </trigger>
        <listOfEventAssignments>
          <eventAssignment
variable="mw7d178aae_ff78_412a_830f_3be325d22773">
            <math xmlns="http://www.w3.org/1998/Math/MathML">
              <cn type="integer"> 0 </cn>
            </math>
          </eventAssignment>
        </listOfEventAssignments>
      </event>
      <event id="mwfe9c9930_c067_4f25_a85f_ebeb480cd7cf"
name="event_230">
        <trigger>
          <math xmlns="http://www.w3.org/1998/Math/MathML">
            <apply>
              <geq/>
              <csymbol encoding="text"
definitionURL="http://www.sbml.org/sbml/symbols/time"> time </csymbol>
              <cn> 249.4 </cn>
            </apply>
          </math>
        </trigger>
        <listOfEventAssignments>
          <eventAssignment
variable="mw7d178aae_ff78_412a_830f_3be325d22773">
            <math xmlns="http://www.w3.org/1998/Math/MathML">
              <cn type="integer"> 1 </cn>
            </math>
          </eventAssignment>
        </listOfEventAssignments>
      </event>
```

```
      <event id="mw2336f7c9_02e7_483f_8af3_34752048c516"
name="event_231">
        <trigger>
          <math xmlns="http://www.w3.org/1998/Math/MathML">
            <apply>
              <geq/>
              <csymbol encoding="text"
definitionURL="http://www.sbml.org/sbml/symbols/time"> time </csymbol>
              <cn> 252.27 </cn>
            </apply>
          </math>
        </trigger>
        <listOfEventAssignments>
          <eventAssignment
variable="mw7d178aae_ff78_412a_830f_3be325d22773">
            <math xmlns="http://www.w3.org/1998/Math/MathML">
              <cn type="integer"> 0 </cn>
            </math>
          </eventAssignment>
        </listOfEventAssignments>
      </event>
      <event id="mw501e7e2b_7909_4f80_8823_e8350fa38673"
name="event_232">
        <trigger>
          <math xmlns="http://www.w3.org/1998/Math/MathML">
            <apply>
              <geq/>
              <csymbol encoding="text"
definitionURL="http://www.sbml.org/sbml/symbols/time"> time </csymbol>
              <cn> 253.7 </cn>
            </apply>
          </math>
        </trigger>
        <listOfEventAssignments>
          <eventAssignment
variable="mw7d178aae_ff78_412a_830f_3be325d22773">
            <math xmlns="http://www.w3.org/1998/Math/MathML">
              <cn type="integer"> 1 </cn>
            </math>
          </eventAssignment>
        </listOfEventAssignments>
      </event>
      <event id="mw47081e49_54c5_4e68_90fa_12b62ae2908a"
name="event_233">
        <trigger>
          <math xmlns="http://www.w3.org/1998/Math/MathML">
            <apply>
              <geq/>
              <csymbol encoding="text"
definitionURL="http://www.sbml.org/sbml/symbols/time"> time </csymbol>
              <cn> 256.57 </cn>
            </apply>
```

```
          </math>
        </trigger>
        <listOfEventAssignments>
          <eventAssignment
variable="mw7d178aae_ff78_412a_830f_3be325d22773">
            <math xmlns="http://www.w3.org/1998/Math/MathML">
              <cn type="integer"> 0 </cn>
            </math>
          </eventAssignment>
        </listOfEventAssignments>
      </event>
      <event id="mw6af34870_68b7_44b8_8533_673013fc9f59"
name="event_234">
        <trigger>
          <math xmlns="http://www.w3.org/1998/Math/MathML">
            <apply>
              <geq/>
              <csymbol encoding="text"
definitionURL="http://www.sbml.org/sbml/symbols/time"> time </csymbol>
              <cn type="integer"> 258 </cn>
            </apply>
          </math>
        </trigger>
        <listOfEventAssignments>
          <eventAssignment
variable="mw7d178aae_ff78_412a_830f_3be325d22773">
            <math xmlns="http://www.w3.org/1998/Math/MathML">
              <cn type="integer"> 1 </cn>
            </math>
          </eventAssignment>
        </listOfEventAssignments>
      </event>
      <event id="mw59de027e_f376_4c1e_8242_8c0cdf24196f"
name="event_235">
        <trigger>
          <math xmlns="http://www.w3.org/1998/Math/MathML">
            <apply>
              <geq/>
              <csymbol encoding="text"
definitionURL="http://www.sbml.org/sbml/symbols/time"> time </csymbol>
              <cn> 247.97 </cn>
            </apply>
          </math>
        </trigger>
        <listOfEventAssignments>
          <eventAssignment
variable="mwcedbe49e_2d28_4720_8fcd_207db64228cf">
            <math xmlns="http://www.w3.org/1998/Math/MathML">
              <cn type="integer"> 1 </cn>
            </math>
          </eventAssignment>
        </listOfEventAssignments>
```

```
      </event>
      <event id="mweff02d5e_932e_4493_8ac9_e67e4530ea1d"
name="event_236">
        <trigger>
          <math xmlns="http://www.w3.org/1998/Math/MathML">
            <apply>
              <geq/>
              <csymbol encoding="text"
definitionURL="http://www.sbml.org/sbml/symbols/time"> time </csymbol>
              <cn> 249.4 </cn>
            </apply>
          </math>
        </trigger>
        <listOfEventAssignments>
          <eventAssignment
variable="mwcedbe49e_2d28_4720_8fcd_207db64228cf">
            <math xmlns="http://www.w3.org/1998/Math/MathML">
              <cn type="integer"> 0 </cn>
            </math>
          </eventAssignment>
        </listOfEventAssignments>
      </event>
      <event id="mwbd21b63a_7600_4b42_8544_9974dd09bece"
name="event_237">
        <trigger>
          <math xmlns="http://www.w3.org/1998/Math/MathML">
            <apply>
              <geq/>
              <csymbol encoding="text"
definitionURL="http://www.sbml.org/sbml/symbols/time"> time </csymbol>
              <cn> 252.27 </cn>
            </apply>
          </math>
        </trigger>
        <listOfEventAssignments>
          <eventAssignment
variable="mwcedbe49e_2d28_4720_8fcd_207db64228cf">
            <math xmlns="http://www.w3.org/1998/Math/MathML">
              <cn type="integer"> 1 </cn>
            </math>
          </eventAssignment>
        </listOfEventAssignments>
      </event>
      <event id="mw9857b266_8489_42cc_bbb0_c967d7874f99"
name="event_238">
        <trigger>
          <math xmlns="http://www.w3.org/1998/Math/MathML">
            <apply>
              <geq/>
              <csymbol encoding="text"
definitionURL="http://www.sbml.org/sbml/symbols/time"> time </csymbol>
              <cn> 253.7 </cn>
```

```
            </apply>
          </math>
        </trigger>
        <listOfEventAssignments>
          <eventAssignment
variable="mwcedbe49e_2d28_4720_8fcd_207db64228cf">
            <math xmlns="http://www.w3.org/1998/Math/MathML">
              <cn type="integer"> 0 </cn>
            </math>
          </eventAssignment>
        </listOfEventAssignments>
      </event>
      <event id="mwdc1f340e_5a55_4219_a81d_2a7579897493"
name="event_239">
        <trigger>
          <math xmlns="http://www.w3.org/1998/Math/MathML">
            <apply>
              <geq/>
              <csymbol encoding="text"
definitionURL="http://www.sbml.org/sbml/symbols/time"> time </csymbol>
              <cn> 256.57 </cn>
            </apply>
          </math>
        </trigger>
        <listOfEventAssignments>
          <eventAssignment
variable="mwcedbe49e_2d28_4720_8fcd_207db64228cf">
            <math xmlns="http://www.w3.org/1998/Math/MathML">
              <cn type="integer"> 1 </cn>
            </math>
          </eventAssignment>
        </listOfEventAssignments>
      </event>
      <event id="mw6960d083_c9c8_46e8_a736_5a0b6dd23ae5"
name="event_240">
        <trigger>
          <math xmlns="http://www.w3.org/1998/Math/MathML">
            <apply>
              <geq/>
              <csymbol encoding="text"
definitionURL="http://www.sbml.org/sbml/symbols/time"> time </csymbol>
              <cn type="integer"> 258 </cn>
            </apply>
          </math>
        </trigger>
        <listOfEventAssignments>
          <eventAssignment
variable="mwcedbe49e_2d28_4720_8fcd_207db64228cf">
            <math xmlns="http://www.w3.org/1998/Math/MathML">
              <cn type="integer"> 0 </cn>
            </math>
          </eventAssignment>
```

```
        &lt;/listOfEventAssignments&gt;
      &lt;/event&gt;
      <event id="mwe4c4f58d_f0dd_4a4b_91c6_c7cffb61c4f3"
name="event_241">
        &lt;trigger&gt;
          &lt;math xmlns="http://www.w3.org/1998/Math/MathML"&gt;
            &lt;apply&gt;
              &lt;geq/&gt;
              <csymbol encoding="text"
definitionURL="http://www.sbml.org/sbml/symbols/time"> time &lt;/csymbol&gt;
              &lt;cn&gt; 260.87 &lt;/cn&gt;
            &lt;/apply&gt;
          &lt;/math&gt;
        &lt;/trigger&gt;
        &lt;listOfEventAssignments&gt;
          <eventAssignment
variable="mw7d178aae_ff78_412a_830f_3be325d22773">
            &lt;math xmlns="http://www.w3.org/1998/Math/MathML"&gt;
              &lt;cn type="integer"&gt; 0 &lt;/cn&gt;
            &lt;/math&gt;
          &lt;/eventAssignment&gt;
        &lt;/listOfEventAssignments&gt;
      &lt;/event&gt;
      <event id="mwdc88ea4c_ccf0_4c2b_9ea0_f8dda641ace6"
name="event_242">
        &lt;trigger&gt;
          &lt;math xmlns="http://www.w3.org/1998/Math/MathML"&gt;
            &lt;apply&gt;
              &lt;geq/&gt;
              <csymbol encoding="text"
definitionURL="http://www.sbml.org/sbml/symbols/time"> time &lt;/csymbol&gt;
              &lt;cn&gt; 262.3 &lt;/cn&gt;
            &lt;/apply&gt;
          &lt;/math&gt;
        &lt;/trigger&gt;
        &lt;listOfEventAssignments&gt;
          <eventAssignment
variable="mw7d178aae_ff78_412a_830f_3be325d22773">
            &lt;math xmlns="http://www.w3.org/1998/Math/MathML"&gt;
              &lt;cn type="integer"&gt; 1 &lt;/cn&gt;
            &lt;/math&gt;
          &lt;/eventAssignment&gt;
        &lt;/listOfEventAssignments&gt;
      &lt;/event&gt;
      <event id="mw6964490a_24f2_40b9_bdfd_5769c33507cf"
name="event_243">
        &lt;trigger&gt;
          &lt;math xmlns="http://www.w3.org/1998/Math/MathML"&gt;
            &lt;apply&gt;
              &lt;geq/&gt;
              <csymbol encoding="text"
definitionURL="http://www.sbml.org/sbml/symbols/time"> time &lt;/csymbol&gt;
```

```
              &lt;cn&gt; 265.17 &lt;/cn&gt;
            &lt;/apply&gt;
          &lt;/math&gt;
        &lt;/trigger&gt;
        &lt;listOfEventAssignments&gt;
          <eventAssignment
variable="mw7d178aae_ff78_412a_830f_3be325d22773">
            &lt;math xmlns="http://www.w3.org/1998/Math/MathML"&gt;
              &lt;cn type="integer"&gt; 0 &lt;/cn&gt;
            &lt;/math&gt;
          &lt;/eventAssignment&gt;
        &lt;/listOfEventAssignments&gt;
      &lt;/event&gt;
      <event id="mw80b76227_b509_431a_9d14_5c0c78a52c0b"
name="event_244">
        &lt;trigger&gt;
          &lt;math xmlns="http://www.w3.org/1998/Math/MathML"&gt;
            &lt;apply&gt;
              &lt;geq/&gt;
              <csymbol encoding="text"
definitionURL="http://www.sbml.org/sbml/symbols/time"> time &lt;/csymbol&gt;
              &lt;cn&gt; 266.6 &lt;/cn&gt;
            &lt;/apply&gt;
          &lt;/math&gt;
        &lt;/trigger&gt;
        &lt;listOfEventAssignments&gt;
          <eventAssignment
variable="mw7d178aae_ff78_412a_830f_3be325d22773">
            &lt;math xmlns="http://www.w3.org/1998/Math/MathML"&gt;
              &lt;cn type="integer"&gt; 1 &lt;/cn&gt;
            &lt;/math&gt;
          &lt;/eventAssignment&gt;
        &lt;/listOfEventAssignments&gt;
      &lt;/event&gt;
      <event id="mw30b61e8a_7d9d_4bfd_9024_ea919e9b6585"
name="event_245">
        &lt;trigger&gt;
          &lt;math xmlns="http://www.w3.org/1998/Math/MathML"&gt;
            &lt;apply&gt;
              &lt;geq/&gt;
              <csymbol encoding="text"
definitionURL="http://www.sbml.org/sbml/symbols/time"> time &lt;/csymbol&gt;
              &lt;cn&gt; 269.47 &lt;/cn&gt;
            &lt;/apply&gt;
          &lt;/math&gt;
        &lt;/trigger&gt;
        &lt;listOfEventAssignments&gt;
          <eventAssignment
variable="mw7d178aae_ff78_412a_830f_3be325d22773">
            &lt;math xmlns="http://www.w3.org/1998/Math/MathML"&gt;
              &lt;cn type="integer"&gt; 0 &lt;/cn&gt;
            &lt;/math&gt;
```

```
          &lt;/eventAssignment&gt;
        &lt;/listOfEventAssignments&gt;
      &lt;/event&gt;
      <event id="mwbab3a784_5d5d_450b_96fd_7945e8a96ddc"
name="event_246">
        &lt;trigger&gt;
          &lt;math xmlns="http://www.w3.org/1998/Math/MathML"&gt;
            &lt;apply&gt;
              &lt;geq/&gt;
              <csymbol encoding="text"
definitionURL="http://www.sbml.org/sbml/symbols/time"> time &lt;/csymbol&gt;
              &lt;cn&gt; 270.9 &lt;/cn&gt;
            &lt;/apply&gt;
          &lt;/math&gt;
        &lt;/trigger&gt;
        &lt;listOfEventAssignments&gt;
          <eventAssignment
variable="mw7d178aae_ff78_412a_830f_3be325d22773">
            &lt;math xmlns="http://www.w3.org/1998/Math/MathML"&gt;
              &lt;cn type="integer"&gt; 1 &lt;/cn&gt;
            &lt;/math&gt;
          &lt;/eventAssignment&gt;
        &lt;/listOfEventAssignments&gt;
      &lt;/event&gt;
      <event id="mwde5264bc_1e9a_4332_8afd_0a02f1adcf60"
name="event_247">
        &lt;trigger&gt;
          &lt;math xmlns="http://www.w3.org/1998/Math/MathML"&gt;
            &lt;apply&gt;
              &lt;geq/&gt;
              <csymbol encoding="text"
definitionURL="http://www.sbml.org/sbml/symbols/time"> time &lt;/csymbol&gt;
              &lt;cn&gt; 273.77 &lt;/cn&gt;
            &lt;/apply&gt;
          &lt;/math&gt;
        &lt;/trigger&gt;
        &lt;listOfEventAssignments&gt;
          <eventAssignment
variable="mw7d178aae_ff78_412a_830f_3be325d22773">
            &lt;math xmlns="http://www.w3.org/1998/Math/MathML"&gt;
              &lt;cn type="integer"&gt; 0 &lt;/cn&gt;
            &lt;/math&gt;
          &lt;/eventAssignment&gt;
        &lt;/listOfEventAssignments&gt;
      &lt;/event&gt;
      <event id="mw08ad67fc_bdd8_4e42_a7c5_438b9b853808"
name="event_248">
        &lt;trigger&gt;
          &lt;math xmlns="http://www.w3.org/1998/Math/MathML"&gt;
            &lt;apply&gt;
              &lt;geq/&gt;
```

```
              <csymbol encoding="text"
definitionURL="http://www.sbml.org/sbml/symbols/time"> time </csymbol>
              <cn> 275.2 </cn>
            </apply>
          </math>
        </trigger>
        <listOfEventAssignments>
          <eventAssignment
variable="mw7d178aae_ff78_412a_830f_3be325d22773">
            <math xmlns="http://www.w3.org/1998/Math/MathML">
              <cn type="integer"> 1 </cn>
            </math>
          </eventAssignment>
        </listOfEventAssignments>
      </event>
      <event id="mwac10e708_a3e3_476b_8dda_0501b52a8e5e"
name="event_249">
        <trigger>
          <math xmlns="http://www.w3.org/1998/Math/MathML">
            <apply>
              <geq/>
              <csymbol encoding="text"
definitionURL="http://www.sbml.org/sbml/symbols/time"> time </csymbol>
              <cn> 278.07 </cn>
            </apply>
          </math>
        </trigger>
        <listOfEventAssignments>
          <eventAssignment
variable="mw7d178aae_ff78_412a_830f_3be325d22773">
            <math xmlns="http://www.w3.org/1998/Math/MathML">
              <cn type="integer"> 0 </cn>
            </math>
          </eventAssignment>
        </listOfEventAssignments>
      </event>
      <event id="mw90f83909_0bdf_4216_88df_0639cc4d49c7"
name="event_250">
        <trigger>
          <math xmlns="http://www.w3.org/1998/Math/MathML">
            <apply>
              <geq/>
              <csymbol encoding="text"
definitionURL="http://www.sbml.org/sbml/symbols/time"> time </csymbol>
              <cn> 279.5 </cn>
            </apply>
          </math>
        </trigger>
        <listOfEventAssignments>
          <eventAssignment
variable="mw7d178aae_ff78_412a_830f_3be325d22773">
            <math xmlns="http://www.w3.org/1998/Math/MathML">
```

```
              <cn type="integer"> 1 </cn>
            </math>
          </eventAssignment>
        </listOfEventAssignments>
      </event>
      <event id="mwc89de84c_cb1c_4992_b32d_98ebfc4e78e2"
name="event_251">
        <trigger>
          <math xmlns="http://www.w3.org/1998/Math/MathML">
            <apply>
              <geq/>
              <csymbol encoding="text"
definitionURL="http://www.sbml.org/sbml/symbols/time"> time </csymbol>
              <cn> 282.37 </cn>
            </apply>
          </math>
        </trigger>
        <listOfEventAssignments>
          <eventAssignment
variable="mw7d178aae_ff78_412a_830f_3be325d22773">
            <math xmlns="http://www.w3.org/1998/Math/MathML">
              <cn type="integer"> 0 </cn>
            </math>
          </eventAssignment>
        </listOfEventAssignments>
      </event>
      <event id="mw8eca7e8d_4848_4306_a013_c8b76fb423df"
name="event_252">
        <trigger>
          <math xmlns="http://www.w3.org/1998/Math/MathML">
            <apply>
              <geq/>
              <csymbol encoding="text"
definitionURL="http://www.sbml.org/sbml/symbols/time"> time </csymbol>
              <cn> 283.8 </cn>
            </apply>
          </math>
        </trigger>
        <listOfEventAssignments>
          <eventAssignment
variable="mw7d178aae_ff78_412a_830f_3be325d22773">
            <math xmlns="http://www.w3.org/1998/Math/MathML">
              <cn type="integer"> 1 </cn>
            </math>
          </eventAssignment>
        </listOfEventAssignments>
      </event>
      <event id="mw562094f1_ed96_498c_906a_a1a9865aae7f"
name="event_253">
        <trigger>
          <math xmlns="http://www.w3.org/1998/Math/MathML">
            <apply>
```

```
              <geq/>
              <csymbol encoding="text"
definitionURL="http://www.sbml.org/sbml/symbols/time"> time </csymbol>
              <cn> 286.67 </cn>
            </apply>
          </math>
        </trigger>
        <listOfEventAssignments>
          <eventAssignment
variable="mw7d178aae_ff78_412a_830f_3be325d22773">
            <math xmlns="http://www.w3.org/1998/Math/MathML">
              <cn type="integer"> 0 </cn>
            </math>
          </eventAssignment>
        </listOfEventAssignments>
      </event>
      <event id="mw056f99cd_7297_4495_b78c_7ec0d95f3306"
name="event_254">
        <trigger>
          <math xmlns="http://www.w3.org/1998/Math/MathML">
            <apply>
              <geq/>
              <csymbol encoding="text"
definitionURL="http://www.sbml.org/sbml/symbols/time"> time </csymbol>
              <cn> 288.1 </cn>
            </apply>
          </math>
        </trigger>
        <listOfEventAssignments>
          <eventAssignment
variable="mw7d178aae_ff78_412a_830f_3be325d22773">
            <math xmlns="http://www.w3.org/1998/Math/MathML">
              <cn type="integer"> 1 </cn>
            </math>
          </eventAssignment>
        </listOfEventAssignments>
      </event>
      <event id="mwb074cabe_b8b0_4ab8_a107_af098230ee13"
name="event_255">
        <trigger>
          <math xmlns="http://www.w3.org/1998/Math/MathML">
            <apply>
              <geq/>
              <csymbol encoding="text"
definitionURL="http://www.sbml.org/sbml/symbols/time"> time </csymbol>
              <cn> 290.97 </cn>
            </apply>
          </math>
        </trigger>
        <listOfEventAssignments>
          <eventAssignment
variable="mw7d178aae_ff78_412a_830f_3be325d22773">
```

```
            <math xmlns="http://www.w3.org/1998/Math/MathML">
              <cn type="integer"> 0 </cn>
            </math>
          </eventAssignment>
        </listOfEventAssignments>
      </event>
      <event id="mwf4609902_2822_4b9d_85a1_53bda9b8b925"
name="event_256">
        <trigger>
          <math xmlns="http://www.w3.org/1998/Math/MathML">
            <apply>
              <geq/>
              <csymbol encoding="text"
definitionURL="http://www.sbml.org/sbml/symbols/time"> time </csymbol>
              <cn> 292.4 </cn>
            </apply>
          </math>
        </trigger>
        <listOfEventAssignments>
          <eventAssignment
variable="mw7d178aae_ff78_412a_830f_3be325d22773">
            <math xmlns="http://www.w3.org/1998/Math/MathML">
              <cn type="integer"> 1 </cn>
            </math>
          </eventAssignment>
        </listOfEventAssignments>
      </event>
      <event id="mwc90f7089_e580_47bf_ba5b_e87717ee45ce"
name="event_257">
        <trigger>
          <math xmlns="http://www.w3.org/1998/Math/MathML">
            <apply>
              <geq/>
              <csymbol encoding="text"
definitionURL="http://www.sbml.org/sbml/symbols/time"> time </csymbol>
              <cn> 295.27 </cn>
            </apply>
          </math>
        </trigger>
        <listOfEventAssignments>
          <eventAssignment
variable="mw7d178aae_ff78_412a_830f_3be325d22773">
            <math xmlns="http://www.w3.org/1998/Math/MathML">
              <cn type="integer"> 0 </cn>
            </math>
          </eventAssignment>
        </listOfEventAssignments>
      </event>
      <event id="mwc964cf39_5b40_4238_933f_c44e3557993f"
name="event_258">
        <trigger>
          <math xmlns="http://www.w3.org/1998/Math/MathML">
```

```
          <apply>
            <geq/>
            <csymbol encoding="text"
definitionURL="http://www.sbml.org/sbml/symbols/time"> time </csymbol>
            <cn> 296.7 </cn>
          </apply>
        </math>
      </trigger>
      <listOfEventAssignments>
        <eventAssignment
variable="mw7d178aae_ff78_412a_830f_3be325d22773">
          <math xmlns="http://www.w3.org/1998/Math/MathML">
            <cn type="integer"> 1 </cn>
          </math>
        </eventAssignment>
      </listOfEventAssignments>
    </event>
    <event id="mw044c4d85_0d5e_4e8b_a29a_ef038d6594d4"
name="event_259">
      <trigger>
        <math xmlns="http://www.w3.org/1998/Math/MathML">
          <apply>
            <geq/>
            <csymbol encoding="text"
definitionURL="http://www.sbml.org/sbml/symbols/time"> time </csymbol>
            <cn> 299.57 </cn>
          </apply>
        </math>
      </trigger>
      <listOfEventAssignments>
        <eventAssignment
variable="mw7d178aae_ff78_412a_830f_3be325d22773">
          <math xmlns="http://www.w3.org/1998/Math/MathML">
            <cn type="integer"> 0 </cn>
          </math>
        </eventAssignment>
      </listOfEventAssignments>
    </event>
    <event id="mwcbeec7e8_4328_4e29_b833_6484ea12ecaa"
name="event_260">
      <trigger>
        <math xmlns="http://www.w3.org/1998/Math/MathML">
          <apply>
            <geq/>
            <csymbol encoding="text"
definitionURL="http://www.sbml.org/sbml/symbols/time"> time </csymbol>
            <cn type="integer"> 301 </cn>
          </apply>
        </math>
      </trigger>
      <listOfEventAssignments>
```

```
          <eventAssignment
variable="mw7d178aae_ff78_412a_830f_3be325d22773">
            <math xmlns="http://www.w3.org/1998/Math/MathML">
              <cn type="integer"> 1 </cn>
            </math>
          </eventAssignment>
        </listOfEventAssignments>
      </event>
      <event id="mwb33df3fb_4dfb_49ae_8b0a_1b8d7309a80b"
name="event_261">
        <trigger>
          <math xmlns="http://www.w3.org/1998/Math/MathML">
            <apply>
              <geq/>
              <csymbol encoding="text"
definitionURL="http://www.sbml.org/sbml/symbols/time"> time </csymbol>
              <cn> 260.87 </cn>
            </apply>
          </math>
        </trigger>
        <listOfEventAssignments>
          <eventAssignment
variable="mwcedbe49e_2d28_4720_8fcd_207db64228cf">
            <math xmlns="http://www.w3.org/1998/Math/MathML">
              <cn type="integer"> 1 </cn>
            </math>
          </eventAssignment>
        </listOfEventAssignments>
      </event>
      <event id="mw6e16463b_4b24_422b_b483_295fef0237a5"
name="event_262">
        <trigger>
          <math xmlns="http://www.w3.org/1998/Math/MathML">
            <apply>
              <geq/>
              <csymbol encoding="text"
definitionURL="http://www.sbml.org/sbml/symbols/time"> time </csymbol>
              <cn> 262.3 </cn>
            </apply>
          </math>
        </trigger>
        <listOfEventAssignments>
          <eventAssignment
variable="mwcedbe49e_2d28_4720_8fcd_207db64228cf">
            <math xmlns="http://www.w3.org/1998/Math/MathML">
              <cn type="integer"> 0 </cn>
            </math>
          </eventAssignment>
        </listOfEventAssignments>
      </event>
      <event id="mw35afb587_9f93_4ef6_b9b0_2fcfb1cadacb"
name="event_263">
```

```
        <trigger>
          <math xmlns="http://www.w3.org/1998/Math/MathML">
            <apply>
              <geq/>
              <csymbol encoding="text"
definitionURL="http://www.sbml.org/sbml/symbols/time"> time </csymbol>
              <cn> 265.17 </cn>
            </apply>
          </math>
        </trigger>
        <listOfEventAssignments>
          <eventAssignment
variable="mwcedbe49e_2d28_4720_8fcd_207db64228cf">
            <math xmlns="http://www.w3.org/1998/Math/MathML">
              <cn type="integer"> 1 </cn>
            </math>
          </eventAssignment>
        </listOfEventAssignments>
      </event>
      <event id="mw1465d169_8079_4bb7_90af_39d2b7193dc7"
name="event_264">
        <trigger>
          <math xmlns="http://www.w3.org/1998/Math/MathML">
            <apply>
              <geq/>
              <csymbol encoding="text"
definitionURL="http://www.sbml.org/sbml/symbols/time"> time </csymbol>
              <cn> 266.6 </cn>
            </apply>
          </math>
        </trigger>
        <listOfEventAssignments>
          <eventAssignment
variable="mwcedbe49e_2d28_4720_8fcd_207db64228cf">
            <math xmlns="http://www.w3.org/1998/Math/MathML">
              <cn type="integer"> 0 </cn>
            </math>
          </eventAssignment>
        </listOfEventAssignments>
      </event>
      <event id="mw0a71e0e0_16ef_4227_97ac_ad86ad239507"
name="event_265">
        <trigger>
          <math xmlns="http://www.w3.org/1998/Math/MathML">
            <apply>
              <geq/>
              <csymbol encoding="text"
definitionURL="http://www.sbml.org/sbml/symbols/time"> time </csymbol>
              <cn> 269.47 </cn>
            </apply>
          </math>
        </trigger>
```

```
        <listOfEventAssignments>
          <eventAssignment
variable="mwcedbe49e_2d28_4720_8fcd_207db64228cf">
            <math xmlns="http://www.w3.org/1998/Math/MathML">
              <cn type="integer"> 1 </cn>
            </math>
          </eventAssignment>
        </listOfEventAssignments>
      </event>
      <event id="mw65bac45e_544f_45ea_a180_e0a71908c525"
name="event_266">
        <trigger>
          <math xmlns="http://www.w3.org/1998/Math/MathML">
            <apply>
              <geq/>
              <csymbol encoding="text"
definitionURL="http://www.sbml.org/sbml/symbols/time"> time </csymbol>
              <cn> 270.9 </cn>
            </apply>
          </math>
        </trigger>
        <listOfEventAssignments>
          <eventAssignment
variable="mwcedbe49e_2d28_4720_8fcd_207db64228cf">
            <math xmlns="http://www.w3.org/1998/Math/MathML">
              <cn type="integer"> 0 </cn>
            </math>
          </eventAssignment>
        </listOfEventAssignments>
      </event>
      <event id="mwfbdc703f_11e0_4bd8_a1ad_d50c0ce203e1"
name="event_267">
        <trigger>
          <math xmlns="http://www.w3.org/1998/Math/MathML">
            <apply>
              <geq/>
              <csymbol encoding="text"
definitionURL="http://www.sbml.org/sbml/symbols/time"> time </csymbol>
              <cn> 273.77 </cn>
            </apply>
          </math>
        </trigger>
        <listOfEventAssignments>
          <eventAssignment
variable="mwcedbe49e_2d28_4720_8fcd_207db64228cf">
            <math xmlns="http://www.w3.org/1998/Math/MathML">
              <cn type="integer"> 1 </cn>
            </math>
          </eventAssignment>
        </listOfEventAssignments>
      </event>
```

```
      <event id="mw9e39e492_6fd7_42be_89a5_9d6f1962f84f"
name="event_268">
        &lt;trigger&gt;
          &lt;math xmlns="http://www.w3.org/1998/Math/MathML"&gt;
            &lt;apply&gt;
              &lt;geq/&gt;
              <csymbol encoding="text"
definitionURL="http://www.sbml.org/sbml/symbols/time"> time &lt;/csymbol&gt;
              &lt;cn&gt; 275.2 &lt;/cn&gt;
            &lt;/apply&gt;
          &lt;/math&gt;
        &lt;/trigger&gt;
        &lt;listOfEventAssignments&gt;
          <eventAssignment
variable="mwcedbe49e_2d28_4720_8fcd_207db64228cf">
            &lt;math xmlns="http://www.w3.org/1998/Math/MathML"&gt;
              &lt;cn type="integer"&gt; 0 &lt;/cn&gt;
            &lt;/math&gt;
          &lt;/eventAssignment&gt;
        &lt;/listOfEventAssignments&gt;
      &lt;/event&gt;
      <event id="mw0c2b4f43_eb80_4a45_a996_e377d2b441c1"
name="event_269">
        &lt;trigger&gt;
          &lt;math xmlns="http://www.w3.org/1998/Math/MathML"&gt;
            &lt;apply&gt;
              &lt;geq/&gt;
              <csymbol encoding="text"
definitionURL="http://www.sbml.org/sbml/symbols/time"> time &lt;/csymbol&gt;
              &lt;cn&gt; 278.07 &lt;/cn&gt;
            &lt;/apply&gt;
          &lt;/math&gt;
        &lt;/trigger&gt;
        &lt;listOfEventAssignments&gt;
          <eventAssignment
variable="mwcedbe49e_2d28_4720_8fcd_207db64228cf">
            &lt;math xmlns="http://www.w3.org/1998/Math/MathML"&gt;
              &lt;cn type="integer"&gt; 1 &lt;/cn&gt;
            &lt;/math&gt;
          &lt;/eventAssignment&gt;
        &lt;/listOfEventAssignments&gt;
      &lt;/event&gt;
      <event id="mwcbb9283f_a760_4366_9b11_cc7dd0139aaa"
name="event_270">
        &lt;trigger&gt;
          &lt;math xmlns="http://www.w3.org/1998/Math/MathML"&gt;
            &lt;apply&gt;
              &lt;geq/&gt;
              <csymbol encoding="text"
definitionURL="http://www.sbml.org/sbml/symbols/time"> time &lt;/csymbol&gt;
              &lt;cn&gt; 279.5 &lt;/cn&gt;
            &lt;/apply&gt;
```

```
          &lt;/math&gt;
        &lt;/trigger&gt;
        &lt;listOfEventAssignments&gt;
          <eventAssignment
variable="mwcedbe49e_2d28_4720_8fcd_207db64228cf">
            &lt;math xmlns="http://www.w3.org/1998/Math/MathML"&gt;
              &lt;cn type="integer"&gt; 0 &lt;/cn&gt;
            &lt;/math&gt;
          &lt;/eventAssignment&gt;
        &lt;/listOfEventAssignments&gt;
      &lt;/event&gt;
      <event id="mw6ba9ed43_a6b5_41c7_ad8a_745b61187daa"
name="event_271">
        &lt;trigger&gt;
          &lt;math xmlns="http://www.w3.org/1998/Math/MathML"&gt;
            &lt;apply&gt;
              &lt;geq/&gt;
              <csymbol encoding="text"
definitionURL="http://www.sbml.org/sbml/symbols/time"> time &lt;/csymbol&gt;
              &lt;cn&gt; 282.37 &lt;/cn&gt;
            &lt;/apply&gt;
          &lt;/math&gt;
        &lt;/trigger&gt;
        &lt;listOfEventAssignments&gt;
          <eventAssignment
variable="mwcedbe49e_2d28_4720_8fcd_207db64228cf">
            &lt;math xmlns="http://www.w3.org/1998/Math/MathML"&gt;
              &lt;cn type="integer"&gt; 1 &lt;/cn&gt;
            &lt;/math&gt;
          &lt;/eventAssignment&gt;
        &lt;/listOfEventAssignments&gt;
      &lt;/event&gt;
      <event id="mw5130cb8b_4d00_4d9c_bba3_5ce4388b97e4"
name="event_272">
        &lt;trigger&gt;
          &lt;math xmlns="http://www.w3.org/1998/Math/MathML"&gt;
            &lt;apply&gt;
              &lt;geq/&gt;
              <csymbol encoding="text"
definitionURL="http://www.sbml.org/sbml/symbols/time"> time &lt;/csymbol&gt;
              &lt;cn&gt; 283.8 &lt;/cn&gt;
            &lt;/apply&gt;
          &lt;/math&gt;
        &lt;/trigger&gt;
        &lt;listOfEventAssignments&gt;
          <eventAssignment
variable="mwcedbe49e_2d28_4720_8fcd_207db64228cf">
            &lt;math xmlns="http://www.w3.org/1998/Math/MathML"&gt;
              &lt;cn type="integer"&gt; 0 &lt;/cn&gt;
            &lt;/math&gt;
          &lt;/eventAssignment&gt;
        &lt;/listOfEventAssignments&gt;
```

```
      </event>
      <event id="mwd862055c_8673_49b6_865b_b6e39bae8863"
name="event_273">
        <trigger>
          <math xmlns="http://www.w3.org/1998/Math/MathML">
            <apply>
              <geq/>
              <csymbol encoding="text"
definitionURL="http://www.sbml.org/sbml/symbols/time"> time </csymbol>
              <cn> 286.67 </cn>
            </apply>
          </math>
        </trigger>
        <listOfEventAssignments>
          <eventAssignment
variable="mwcedbe49e_2d28_4720_8fcd_207db64228cf">
            <math xmlns="http://www.w3.org/1998/Math/MathML">
              <cn type="integer"> 1 </cn>
            </math>
          </eventAssignment>
        </listOfEventAssignments>
      </event>
      <event id="mw15d39753_caf8_48d7_8e10_6f1de422e49a"
name="event_274">
        <trigger>
          <math xmlns="http://www.w3.org/1998/Math/MathML">
            <apply>
              <geq/>
              <csymbol encoding="text"
definitionURL="http://www.sbml.org/sbml/symbols/time"> time </csymbol>
              <cn> 288.1 </cn>
            </apply>
          </math>
        </trigger>
        <listOfEventAssignments>
          <eventAssignment
variable="mwcedbe49e_2d28_4720_8fcd_207db64228cf">
            <math xmlns="http://www.w3.org/1998/Math/MathML">
              <cn type="integer"> 0 </cn>
            </math>
          </eventAssignment>
        </listOfEventAssignments>
      </event>
      <event id="mw20373c32_1a82_4c82_ba4e_7148597184bf"
name="event_275">
        <trigger>
          <math xmlns="http://www.w3.org/1998/Math/MathML">
            <apply>
              <geq/>
              <csymbol encoding="text"
definitionURL="http://www.sbml.org/sbml/symbols/time"> time </csymbol>
              <cn> 290.97 </cn>
```

```
          </apply>
        </math>
      </trigger>
      <listOfEventAssignments>
        <eventAssignment
variable="mwcedbe49e_2d28_4720_8fcd_207db64228cf">
          <math xmlns="http://www.w3.org/1998/Math/MathML">
            <cn type="integer"> 1 </cn>
          </math>
        </eventAssignment>
      </listOfEventAssignments>
    </event>
    <event id="mw0f2ea1bd_fb38_4fae_a067_d3a6fbb37c79"
name="event_276">
      <trigger>
        <math xmlns="http://www.w3.org/1998/Math/MathML">
          <apply>
            <geq/>
            <csymbol encoding="text"
definitionURL="http://www.sbml.org/sbml/symbols/time"> time </csymbol>
            <cn> 292.4 </cn>
          </apply>
        </math>
      </trigger>
      <listOfEventAssignments>
        <eventAssignment
variable="mwcedbe49e_2d28_4720_8fcd_207db64228cf">
          <math xmlns="http://www.w3.org/1998/Math/MathML">
            <cn type="integer"> 0 </cn>
          </math>
        </eventAssignment>
      </listOfEventAssignments>
    </event>
    <event id="mwa7427d1b_c73f_436d_9f48_4571713bc4e8"
name="event_277">
      <trigger>
        <math xmlns="http://www.w3.org/1998/Math/MathML">
          <apply>
            <geq/>
            <csymbol encoding="text"
definitionURL="http://www.sbml.org/sbml/symbols/time"> time </csymbol>
            <cn> 295.27 </cn>
          </apply>
        </math>
      </trigger>
      <listOfEventAssignments>
        <eventAssignment
variable="mwcedbe49e_2d28_4720_8fcd_207db64228cf">
          <math xmlns="http://www.w3.org/1998/Math/MathML">
            <cn type="integer"> 1 </cn>
          </math>
        </eventAssignment>
```

```
        </listOfEventAssignments>
      </event>
      <event id="mw7376e8d7_5cac_4bea_8d6c_809b30e00d14"
name="event_278">
        <trigger>
          <math xmlns="http://www.w3.org/1998/Math/MathML">
            <apply>
              <geq/>
              <csymbol encoding="text"
definitionURL="http://www.sbml.org/sbml/symbols/time"> time </csymbol>
              <cn> 296.7 </cn>
            </apply>
          </math>
        </trigger>
        <listOfEventAssignments>
          <eventAssignment
variable="mwcedbe49e_2d28_4720_8fcd_207db64228cf">
            <math xmlns="http://www.w3.org/1998/Math/MathML">
              <cn type="integer"> 0 </cn>
            </math>
          </eventAssignment>
        </listOfEventAssignments>
      </event>
      <event id="mw975f196d_8dbf_40f9_a377_e89f0344d5cd"
name="event_279">
        <trigger>
          <math xmlns="http://www.w3.org/1998/Math/MathML">
            <apply>
              <geq/>
              <csymbol encoding="text"
definitionURL="http://www.sbml.org/sbml/symbols/time"> time </csymbol>
              <cn> 299.57 </cn>
            </apply>
          </math>
        </trigger>
        <listOfEventAssignments>
          <eventAssignment
variable="mwcedbe49e_2d28_4720_8fcd_207db64228cf">
            <math xmlns="http://www.w3.org/1998/Math/MathML">
              <cn type="integer"> 1 </cn>
            </math>
          </eventAssignment>
        </listOfEventAssignments>
      </event>
      <event id="mw1fc948b6_f678_40f7_a9f6_42567cbf8be5"
name="event_280">
        <trigger>
          <math xmlns="http://www.w3.org/1998/Math/MathML">
            <apply>
              <geq/>
              <csymbol encoding="text"
definitionURL="http://www.sbml.org/sbml/symbols/time"> time </csymbol>
```

```
              <cn type="integer"> 301 </cn>
            </apply>
          </math>
        </trigger>
        <listOfEventAssignments>
          <eventAssignment
variable="mwcedbe49e_2d28_4720_8fcd_207db64228cf">
            <math xmlns="http://www.w3.org/1998/Math/MathML">
              <cn type="integer"> 0 </cn>
            </math>
          </eventAssignment>
        </listOfEventAssignments>
      </event>
      <event id="mwef25e86a_d3ab_4657_8d0d_d69bce66c0c4"
name="event_281">
        <trigger>
          <math xmlns="http://www.w3.org/1998/Math/MathML">
            <apply>
              <geq/>
              <csymbol encoding="text"
definitionURL="http://www.sbml.org/sbml/symbols/time"> time </csymbol>
              <cn> 303.87 </cn>
            </apply>
          </math>
        </trigger>
        <listOfEventAssignments>
          <eventAssignment
variable="mw7d178aae_ff78_412a_830f_3be325d22773">
            <math xmlns="http://www.w3.org/1998/Math/MathML">
              <cn type="integer"> 0 </cn>
            </math>
          </eventAssignment>
        </listOfEventAssignments>
      </event>
      <event id="mw8612fb3b_5856_4973_8fa0_4c3a32d65c37"
name="event_282">
        <trigger>
          <math xmlns="http://www.w3.org/1998/Math/MathML">
            <apply>
              <geq/>
              <csymbol encoding="text"
definitionURL="http://www.sbml.org/sbml/symbols/time"> time </csymbol>
              <cn> 305.3 </cn>
            </apply>
          </math>
        </trigger>
        <listOfEventAssignments>
          <eventAssignment
variable="mw7d178aae_ff78_412a_830f_3be325d22773">
            <math xmlns="http://www.w3.org/1998/Math/MathML">
              <cn type="integer"> 1 </cn>
            </math>
```

```
          &lt;/eventAssignment&gt;
        &lt;/listOfEventAssignments&gt;
      &lt;/event&gt;
      <event id="mwaa8e0af9_b365_41b9_9480_997bc4a1ac5e"
name="event_283">
        &lt;trigger&gt;
          &lt;math xmlns="http://www.w3.org/1998/Math/MathML"&gt;
            &lt;apply&gt;
              &lt;geq/&gt;
              <csymbol encoding="text"
definitionURL="http://www.sbml.org/sbml/symbols/time"> time &lt;/csymbol&gt;
              &lt;cn&gt; 308.17 &lt;/cn&gt;
            &lt;/apply&gt;
          &lt;/math&gt;
        &lt;/trigger&gt;
        &lt;listOfEventAssignments&gt;
          <eventAssignment
variable="mw7d178aae_ff78_412a_830f_3be325d22773">
            &lt;math xmlns="http://www.w3.org/1998/Math/MathML"&gt;
              &lt;cn type="integer"&gt; 0 &lt;/cn&gt;
            &lt;/math&gt;
          &lt;/eventAssignment&gt;
        &lt;/listOfEventAssignments&gt;
      &lt;/event&gt;
      <event id="mwde3b9ebb_a460_45e1_8cc0_8c8c41e434dc"
name="event_284">
        &lt;trigger&gt;
          &lt;math xmlns="http://www.w3.org/1998/Math/MathML"&gt;
            &lt;apply&gt;
              &lt;geq/&gt;
              <csymbol encoding="text"
definitionURL="http://www.sbml.org/sbml/symbols/time"> time &lt;/csymbol&gt;
              &lt;cn&gt; 309.6 &lt;/cn&gt;
            &lt;/apply&gt;
          &lt;/math&gt;
        &lt;/trigger&gt;
        &lt;listOfEventAssignments&gt;
          <eventAssignment
variable="mw7d178aae_ff78_412a_830f_3be325d22773">
            &lt;math xmlns="http://www.w3.org/1998/Math/MathML"&gt;
              &lt;cn type="integer"&gt; 1 &lt;/cn&gt;
            &lt;/math&gt;
          &lt;/eventAssignment&gt;
        &lt;/listOfEventAssignments&gt;
      &lt;/event&gt;
      <event id="mw911ffb4c_cb0c_438d_984d_d293240dfcfb"
name="event_285">
        &lt;trigger&gt;
          &lt;math xmlns="http://www.w3.org/1998/Math/MathML"&gt;
            &lt;apply&gt;
              &lt;geq/&gt;
```

```
              <csymbol encoding="text"
definitionURL="http://www.sbml.org/sbml/symbols/time"> time </csymbol>
              <cn> 312.47 </cn>
            </apply>
          </math>
        </trigger>
        <listOfEventAssignments>
          <eventAssignment
variable="mw7d178aae_ff78_412a_830f_3be325d22773">
            <math xmlns="http://www.w3.org/1998/Math/MathML">
              <cn type="integer"> 0 </cn>
            </math>
          </eventAssignment>
        </listOfEventAssignments>
      </event>
      <event id="mwea8ea176_48f1_48c4_b9ca_b6c03f5f834a"
name="event_286">
        <trigger>
          <math xmlns="http://www.w3.org/1998/Math/MathML">
            <apply>
              <geq/>
              <csymbol encoding="text"
definitionURL="http://www.sbml.org/sbml/symbols/time"> time </csymbol>
              <cn> 313.9 </cn>
            </apply>
          </math>
        </trigger>
        <listOfEventAssignments>
          <eventAssignment
variable="mw7d178aae_ff78_412a_830f_3be325d22773">
            <math xmlns="http://www.w3.org/1998/Math/MathML">
              <cn type="integer"> 1 </cn>
            </math>
          </eventAssignment>
        </listOfEventAssignments>
      </event>
      <event id="mw46de5a5f_d14e_496e_bc4c_af0f6fab6651"
name="event_287">
        <trigger>
          <math xmlns="http://www.w3.org/1998/Math/MathML">
            <apply>
              <geq/>
              <csymbol encoding="text"
definitionURL="http://www.sbml.org/sbml/symbols/time"> time </csymbol>
              <cn> 316.77 </cn>
            </apply>
          </math>
        </trigger>
        <listOfEventAssignments>
          <eventAssignment
variable="mw7d178aae_ff78_412a_830f_3be325d22773">
            <math xmlns="http://www.w3.org/1998/Math/MathML">
```

```
            &lt;cn type="integer"&gt; 0 &lt;/cn&gt;
          &lt;/math&gt;
        &lt;/eventAssignment&gt;
      &lt;/listOfEventAssignments&gt;
    &lt;/event&gt;
    <event id="mw42ae0f7b_096d_4c6e_adc5_1b35f758a226"
name="event_288">
      &lt;trigger&gt;
        &lt;math xmlns="http://www.w3.org/1998/Math/MathML"&gt;
          &lt;apply&gt;
            &lt;geq/&gt;
            <csymbol encoding="text"
definitionURL="http://www.sbml.org/sbml/symbols/time"> time &lt;/csymbol&gt;
            &lt;cn&gt; 318.2 &lt;/cn&gt;
          &lt;/apply&gt;
        &lt;/math&gt;
      &lt;/trigger&gt;
      &lt;listOfEventAssignments&gt;
        <eventAssignment
variable="mw7d178aae_ff78_412a_830f_3be325d22773">
          &lt;math xmlns="http://www.w3.org/1998/Math/MathML"&gt;
            &lt;cn type="integer"&gt; 1 &lt;/cn&gt;
          &lt;/math&gt;
        &lt;/eventAssignment&gt;
      &lt;/listOfEventAssignments&gt;
    &lt;/event&gt;
    <event id="mwa1eafe55_2a90_41e7_8cd8_24e1ce1a7bf5"
name="event_289">
      &lt;trigger&gt;
        &lt;math xmlns="http://www.w3.org/1998/Math/MathML"&gt;
          &lt;apply&gt;
            &lt;geq/&gt;
            <csymbol encoding="text"
definitionURL="http://www.sbml.org/sbml/symbols/time"> time &lt;/csymbol&gt;
            &lt;cn&gt; 321.07 &lt;/cn&gt;
          &lt;/apply&gt;
        &lt;/math&gt;
      &lt;/trigger&gt;
      &lt;listOfEventAssignments&gt;
        <eventAssignment
variable="mw7d178aae_ff78_412a_830f_3be325d22773">
          &lt;math xmlns="http://www.w3.org/1998/Math/MathML"&gt;
            &lt;cn type="integer"&gt; 0 &lt;/cn&gt;
          &lt;/math&gt;
        &lt;/eventAssignment&gt;
      &lt;/listOfEventAssignments&gt;
    &lt;/event&gt;
    <event id="mw301522ee_2cec_417c_8ec0_bf0afd0944fe"
name="event_290">
      &lt;trigger&gt;
        &lt;math xmlns="http://www.w3.org/1998/Math/MathML"&gt;
          &lt;apply&gt;
```

```
              &lt;geq/&gt;
              <csymbol encoding="text"
definitionURL="http://www.sbml.org/sbml/symbols/time"> time &lt;/csymbol&gt;
              &lt;cn&gt; 322.5 &lt;/cn&gt;
            &lt;/apply&gt;
          &lt;/math&gt;
        &lt;/trigger&gt;
        &lt;listOfEventAssignments&gt;
          <eventAssignment
variable="mw7d178aae_ff78_412a_830f_3be325d22773">
            &lt;math xmlns="http://www.w3.org/1998/Math/MathML"&gt;
              &lt;cn type="integer"&gt; 1 &lt;/cn&gt;
            &lt;/math&gt;
          &lt;/eventAssignment&gt;
        &lt;/listOfEventAssignments&gt;
      &lt;/event&gt;
      <event id="mw0b799f6d_6396_47c9_9429_ca6f93e5605c"
name="event_291">
        &lt;trigger&gt;
          &lt;math xmlns="http://www.w3.org/1998/Math/MathML"&gt;
            &lt;apply&gt;
              &lt;geq/&gt;
              <csymbol encoding="text"
definitionURL="http://www.sbml.org/sbml/symbols/time"> time &lt;/csymbol&gt;
              &lt;cn&gt; 325.37 &lt;/cn&gt;
            &lt;/apply&gt;
          &lt;/math&gt;
        &lt;/trigger&gt;
        &lt;listOfEventAssignments&gt;
          <eventAssignment
variable="mw7d178aae_ff78_412a_830f_3be325d22773">
            &lt;math xmlns="http://www.w3.org/1998/Math/MathML"&gt;
              &lt;cn type="integer"&gt; 0 &lt;/cn&gt;
            &lt;/math&gt;
          &lt;/eventAssignment&gt;
        &lt;/listOfEventAssignments&gt;
      &lt;/event&gt;
      <event id="mwaa0e6ced_dbaa_43af_b1d4_d36105e928dd"
name="event_292">
        &lt;trigger&gt;
          &lt;math xmlns="http://www.w3.org/1998/Math/MathML"&gt;
            &lt;apply&gt;
              &lt;geq/&gt;
              <csymbol encoding="text"
definitionURL="http://www.sbml.org/sbml/symbols/time"> time &lt;/csymbol&gt;
              &lt;cn&gt; 326.8 &lt;/cn&gt;
            &lt;/apply&gt;
          &lt;/math&gt;
        &lt;/trigger&gt;
        &lt;listOfEventAssignments&gt;
          <eventAssignment
variable="mw7d178aae_ff78_412a_830f_3be325d22773">
```

```
            &lt;math xmlns="http://www.w3.org/1998/Math/MathML"&gt;
              &lt;cn type="integer"&gt; 1 &lt;/cn&gt;
            &lt;/math&gt;
          &lt;/eventAssignment&gt;
        &lt;/listOfEventAssignments&gt;
      &lt;/event&gt;
      <event id="mweeb34661_5425_408c_83fd_af347c5e59b9"
name="event_293">
        &lt;trigger&gt;
          &lt;math xmlns="http://www.w3.org/1998/Math/MathML"&gt;
            &lt;apply&gt;
              &lt;geq/&gt;
              <csymbol encoding="text"
definitionURL="http://www.sbml.org/sbml/symbols/time"> time &lt;/csymbol&gt;
              &lt;cn&gt; 329.67 &lt;/cn&gt;
            &lt;/apply&gt;
          &lt;/math&gt;
        &lt;/trigger&gt;
        &lt;listOfEventAssignments&gt;
          <eventAssignment
variable="mw7d178aae_ff78_412a_830f_3be325d22773">
            &lt;math xmlns="http://www.w3.org/1998/Math/MathML"&gt;
              &lt;cn type="integer"&gt; 0 &lt;/cn&gt;
            &lt;/math&gt;
          &lt;/eventAssignment&gt;
        &lt;/listOfEventAssignments&gt;
      &lt;/event&gt;
      <event id="mw407eea26_e0cb_4f19_9948_80971fcde774"
name="event_294">
        &lt;trigger&gt;
          &lt;math xmlns="http://www.w3.org/1998/Math/MathML"&gt;
            &lt;apply&gt;
              &lt;geq/&gt;
              <csymbol encoding="text"
definitionURL="http://www.sbml.org/sbml/symbols/time"> time &lt;/csymbol&gt;
              &lt;cn&gt; 331.1 &lt;/cn&gt;
            &lt;/apply&gt;
          &lt;/math&gt;
        &lt;/trigger&gt;
        &lt;listOfEventAssignments&gt;
          <eventAssignment
variable="mw7d178aae_ff78_412a_830f_3be325d22773">
            &lt;math xmlns="http://www.w3.org/1998/Math/MathML"&gt;
              &lt;cn type="integer"&gt; 1 &lt;/cn&gt;
            &lt;/math&gt;
          &lt;/eventAssignment&gt;
        &lt;/listOfEventAssignments&gt;
      &lt;/event&gt;
      <event id="mw270c8263_a944_4072_9c3b_f929a1ed765b"
name="event_295">
        &lt;trigger&gt;
          &lt;math xmlns="http://www.w3.org/1998/Math/MathML"&gt;
```

```
          &lt;apply&gt;
            &lt;geq/&gt;
            <csymbol encoding="text"
definitionURL="http://www.sbml.org/sbml/symbols/time"> time &lt;/csymbol&gt;
            &lt;cn&gt; 333.97 &lt;/cn&gt;
          &lt;/apply&gt;
        &lt;/math&gt;
      &lt;/trigger&gt;
      &lt;listOfEventAssignments&gt;
        <eventAssignment
variable="mw7d178aae_ff78_412a_830f_3be325d22773">
          &lt;math xmlns="http://www.w3.org/1998/Math/MathML"&gt;
            &lt;cn type="integer"&gt; 0 &lt;/cn&gt;
          &lt;/math&gt;
        &lt;/eventAssignment&gt;
      &lt;/listOfEventAssignments&gt;
    &lt;/event&gt;
    <event id="mwda6f784f_da22_49ad_9e65_1dd4dd4d2d19"
name="event_296">
      &lt;trigger&gt;
        &lt;math xmlns="http://www.w3.org/1998/Math/MathML"&gt;
          &lt;apply&gt;
            &lt;geq/&gt;
            <csymbol encoding="text"
definitionURL="http://www.sbml.org/sbml/symbols/time"> time &lt;/csymbol&gt;
            &lt;cn&gt; 335.4 &lt;/cn&gt;
          &lt;/apply&gt;
        &lt;/math&gt;
      &lt;/trigger&gt;
      &lt;listOfEventAssignments&gt;
        <eventAssignment
variable="mw7d178aae_ff78_412a_830f_3be325d22773">
          &lt;math xmlns="http://www.w3.org/1998/Math/MathML"&gt;
            &lt;cn type="integer"&gt; 1 &lt;/cn&gt;
          &lt;/math&gt;
        &lt;/eventAssignment&gt;
      &lt;/listOfEventAssignments&gt;
    &lt;/event&gt;
    <event id="mwe0a1e9f9_a54c_4766_a466_cfe49dff8851"
name="event_297">
      &lt;trigger&gt;
        &lt;math xmlns="http://www.w3.org/1998/Math/MathML"&gt;
          &lt;apply&gt;
            &lt;geq/&gt;
            <csymbol encoding="text"
definitionURL="http://www.sbml.org/sbml/symbols/time"> time &lt;/csymbol&gt;
            &lt;cn&gt; 338.27 &lt;/cn&gt;
          &lt;/apply&gt;
        &lt;/math&gt;
      &lt;/trigger&gt;
      &lt;listOfEventAssignments&gt;
```

```
          <eventAssignment
variable="mw7d178aae_ff78_412a_830f_3be325d22773">
            &lt;math xmlns="http://www.w3.org/1998/Math/MathML"&gt;
              &lt;cn type="integer"&gt; 0 &lt;/cn&gt;
            &lt;/math&gt;
          &lt;/eventAssignment&gt;
        &lt;/listOfEventAssignments&gt;
      &lt;/event&gt;
      <event id="mw9c27a640_9be9_4a76_a4a9_8b606210ea3b"
name="event_298">
        &lt;trigger&gt;
          &lt;math xmlns="http://www.w3.org/1998/Math/MathML"&gt;
            &lt;apply&gt;
              &lt;geq/&gt;
              <csymbol encoding="text"
definitionURL="http://www.sbml.org/sbml/symbols/time"> time &lt;/csymbol&gt;
              &lt;cn&gt; 339.7 &lt;/cn&gt;
            &lt;/apply&gt;
          &lt;/math&gt;
        &lt;/trigger&gt;
        &lt;listOfEventAssignments&gt;
          <eventAssignment
variable="mw7d178aae_ff78_412a_830f_3be325d22773">
            &lt;math xmlns="http://www.w3.org/1998/Math/MathML"&gt;
              &lt;cn type="integer"&gt; 1 &lt;/cn&gt;
            &lt;/math&gt;
          &lt;/eventAssignment&gt;
        &lt;/listOfEventAssignments&gt;
      &lt;/event&gt;
      <event id="mwd02a617b_d23b_4813_8f70_0d972e7f3db7"
name="event_299">
        &lt;trigger&gt;
          &lt;math xmlns="http://www.w3.org/1998/Math/MathML"&gt;
            &lt;apply&gt;
              &lt;geq/&gt;
              <csymbol encoding="text"
definitionURL="http://www.sbml.org/sbml/symbols/time"> time &lt;/csymbol&gt;
              &lt;cn&gt; 342.57 &lt;/cn&gt;
            &lt;/apply&gt;
          &lt;/math&gt;
        &lt;/trigger&gt;
        &lt;listOfEventAssignments&gt;
          <eventAssignment
variable="mw7d178aae_ff78_412a_830f_3be325d22773">
            &lt;math xmlns="http://www.w3.org/1998/Math/MathML"&gt;
              &lt;cn type="integer"&gt; 0 &lt;/cn&gt;
            &lt;/math&gt;
          &lt;/eventAssignment&gt;
        &lt;/listOfEventAssignments&gt;
      &lt;/event&gt;
      <event id="mw9713d4c7_954d_4ae9_996c_39c26006a310"
name="event_300">
```

```
        &lt;trigger&gt;
          &lt;math xmlns="http://www.w3.org/1998/Math/MathML"&gt;
            &lt;apply&gt;
              &lt;geq/&gt;
              <csymbol encoding="text"
definitionURL="http://www.sbml.org/sbml/symbols/time"> time &lt;/csymbol&gt;
              &lt;cn type="integer"&gt; 344 &lt;/cn&gt;
            &lt;/apply&gt;
          &lt;/math&gt;
        &lt;/trigger&gt;
        &lt;listOfEventAssignments&gt;
          <eventAssignment
variable="mw7d178aae_ff78_412a_830f_3be325d22773">
            &lt;math xmlns="http://www.w3.org/1998/Math/MathML"&gt;
              &lt;cn type="integer"&gt; 1 &lt;/cn&gt;
            &lt;/math&gt;
          &lt;/eventAssignment&gt;
        &lt;/listOfEventAssignments&gt;
      &lt;/event&gt;
      <event id="mw090b43ad_23eb_4461_a455_e599eaaef99d"
name="event_301">
        &lt;trigger&gt;
          &lt;math xmlns="http://www.w3.org/1998/Math/MathML"&gt;
            &lt;apply&gt;
              &lt;geq/&gt;
              <csymbol encoding="text"
definitionURL="http://www.sbml.org/sbml/symbols/time"> time &lt;/csymbol&gt;
              &lt;cn&gt; 346.87 &lt;/cn&gt;
            &lt;/apply&gt;
          &lt;/math&gt;
        &lt;/trigger&gt;
        &lt;listOfEventAssignments&gt;
          <eventAssignment
variable="mw7d178aae_ff78_412a_830f_3be325d22773">
            &lt;math xmlns="http://www.w3.org/1998/Math/MathML"&gt;
              &lt;cn type="integer"&gt; 0 &lt;/cn&gt;
            &lt;/math&gt;
          &lt;/eventAssignment&gt;
        &lt;/listOfEventAssignments&gt;
      &lt;/event&gt;
      <event id="mwb2d7b09a_f8f2_42bd_be40_bbf9a7f616d5"
name="event_302">
        &lt;trigger&gt;
          &lt;math xmlns="http://www.w3.org/1998/Math/MathML"&gt;
            &lt;apply&gt;
              &lt;geq/&gt;
              <csymbol encoding="text"
definitionURL="http://www.sbml.org/sbml/symbols/time"> time &lt;/csymbol&gt;
              &lt;cn&gt; 348.3 &lt;/cn&gt;
            &lt;/apply&gt;
          &lt;/math&gt;
        &lt;/trigger&gt;
```

```
        <listOfEventAssignments>
          <eventAssignment
variable="mw7d178aae_ff78_412a_830f_3be325d22773">
            <math xmlns="http://www.w3.org/1998/Math/MathML">
              <cn type="integer"> 1 </cn>
            </math>
          </eventAssignment>
        </listOfEventAssignments>
      </event>
      <event id="mwb2b7b35f_7028_4784_9c77_9db1baf6dfb4"
name="event_303">
        <trigger>
          <math xmlns="http://www.w3.org/1998/Math/MathML">
            <apply>
              <geq/>
              <csymbol encoding="text"
definitionURL="http://www.sbml.org/sbml/symbols/time"> time </csymbol>
              <cn> 351.17 </cn>
            </apply>
          </math>
        </trigger>
        <listOfEventAssignments>
          <eventAssignment
variable="mw7d178aae_ff78_412a_830f_3be325d22773">
            <math xmlns="http://www.w3.org/1998/Math/MathML">
              <cn type="integer"> 0 </cn>
            </math>
          </eventAssignment>
        </listOfEventAssignments>
      </event>
      <event id="mw304e6304_6975_4108_b31e_c68c6225c070"
name="event_304">
        <trigger>
          <math xmlns="http://www.w3.org/1998/Math/MathML">
            <apply>
              <geq/>
              <csymbol encoding="text"
definitionURL="http://www.sbml.org/sbml/symbols/time"> time </csymbol>
              <cn> 352.6 </cn>
            </apply>
          </math>
        </trigger>
        <listOfEventAssignments>
          <eventAssignment
variable="mw7d178aae_ff78_412a_830f_3be325d22773">
            <math xmlns="http://www.w3.org/1998/Math/MathML">
              <cn type="integer"> 1 </cn>
            </math>
          </eventAssignment>
        </listOfEventAssignments>
      </event>
```

```
      <event id="mwf3dbede1_d50e_47dd_9bee_7a59a99cb823"
name="event_305">
        <trigger>
          <math xmlns="http://www.w3.org/1998/Math/MathML">
            <apply>
              <geq/>
              <csymbol encoding="text"
definitionURL="http://www.sbml.org/sbml/symbols/time"> time </csymbol>
              <cn> 355.47 </cn>
            </apply>
          </math>
        </trigger>
        <listOfEventAssignments>
          <eventAssignment
variable="mw7d178aae_ff78_412a_830f_3be325d22773">
            <math xmlns="http://www.w3.org/1998/Math/MathML">
              <cn type="integer"> 0 </cn>
            </math>
          </eventAssignment>
        </listOfEventAssignments>
      </event>
      <event id="mwfc7c034a_dd45_408d_b278_c368ac031749"
name="event_306">
        <trigger>
          <math xmlns="http://www.w3.org/1998/Math/MathML">
            <apply>
              <geq/>
              <csymbol encoding="text"
definitionURL="http://www.sbml.org/sbml/symbols/time"> time </csymbol>
              <cn> 356.9 </cn>
            </apply>
          </math>
        </trigger>
        <listOfEventAssignments>
          <eventAssignment
variable="mw7d178aae_ff78_412a_830f_3be325d22773">
            <math xmlns="http://www.w3.org/1998/Math/MathML">
              <cn type="integer"> 1 </cn>
            </math>
          </eventAssignment>
        </listOfEventAssignments>
      </event>
      <event id="mwc7fada0c_f15e_4383_bab2_3369d7f491e9"
name="event_307">
        <trigger>
          <math xmlns="http://www.w3.org/1998/Math/MathML">
            <apply>
              <geq/>
              <csymbol encoding="text"
definitionURL="http://www.sbml.org/sbml/symbols/time"> time </csymbol>
              <cn> 359.77 </cn>
            </apply>
```

```
          </math>
        </trigger>
        <listOfEventAssignments>
          <eventAssignment
variable="mw7d178aae_ff78_412a_830f_3be325d22773">
            <math xmlns="http://www.w3.org/1998/Math/MathML">
              <cn type="integer"> 0 </cn>
            </math>
          </eventAssignment>
        </listOfEventAssignments>
      </event>
      <event id="mw3227f668_3707_459f_a87c_046ccb487095"
name="event_308">
        <trigger>
          <math xmlns="http://www.w3.org/1998/Math/MathML">
            <apply>
              <geq/>
              <csymbol encoding="text"
definitionURL="http://www.sbml.org/sbml/symbols/time"> time </csymbol>
              <cn> 361.2 </cn>
            </apply>
          </math>
        </trigger>
        <listOfEventAssignments>
          <eventAssignment
variable="mw7d178aae_ff78_412a_830f_3be325d22773">
            <math xmlns="http://www.w3.org/1998/Math/MathML">
              <cn type="integer"> 1 </cn>
            </math>
          </eventAssignment>
        </listOfEventAssignments>
      </event>
      <event id="mw49336e7f_625b_41cc_b1dd_5d3820b793f4"
name="event_309">
        <trigger>
          <math xmlns="http://www.w3.org/1998/Math/MathML">
            <apply>
              <geq/>
              <csymbol encoding="text"
definitionURL="http://www.sbml.org/sbml/symbols/time"> time </csymbol>
              <cn> 364.07 </cn>
            </apply>
          </math>
        </trigger>
        <listOfEventAssignments>
          <eventAssignment
variable="mw7d178aae_ff78_412a_830f_3be325d22773">
            <math xmlns="http://www.w3.org/1998/Math/MathML">
              <cn type="integer"> 0 </cn>
            </math>
          </eventAssignment>
        </listOfEventAssignments>
```

```
      &lt;/event&gt;
      <event id="mw9279c5b2_a539_4560_b3a1_c7e7f11b90fe"
name="event_310">
        &lt;trigger&gt;
          &lt;math xmlns="http://www.w3.org/1998/Math/MathML"&gt;
            &lt;apply&gt;
              &lt;geq/&gt;
              <csymbol encoding="text"
definitionURL="http://www.sbml.org/sbml/symbols/time"> time &lt;/csymbol&gt;
              &lt;cn&gt; 365.5 &lt;/cn&gt;
            &lt;/apply&gt;
          &lt;/math&gt;
        &lt;/trigger&gt;
        &lt;listOfEventAssignments&gt;
          <eventAssignment
variable="mw7d178aae_ff78_412a_830f_3be325d22773">
            &lt;math xmlns="http://www.w3.org/1998/Math/MathML"&gt;
              &lt;cn type="integer"&gt; 1 &lt;/cn&gt;
            &lt;/math&gt;
          &lt;/eventAssignment&gt;
        &lt;/listOfEventAssignments&gt;
      &lt;/event&gt;
      <event id="mw8df4f70f_b260_4158_ae4e_70b1842cb235"
name="event_311">
        &lt;trigger&gt;
          &lt;math xmlns="http://www.w3.org/1998/Math/MathML"&gt;
            &lt;apply&gt;
              &lt;geq/&gt;
              <csymbol encoding="text"
definitionURL="http://www.sbml.org/sbml/symbols/time"> time &lt;/csymbol&gt;
              &lt;cn&gt; 368.37 &lt;/cn&gt;
            &lt;/apply&gt;
          &lt;/math&gt;
        &lt;/trigger&gt;
        &lt;listOfEventAssignments&gt;
          <eventAssignment
variable="mw7d178aae_ff78_412a_830f_3be325d22773">
            &lt;math xmlns="http://www.w3.org/1998/Math/MathML"&gt;
              &lt;cn type="integer"&gt; 0 &lt;/cn&gt;
            &lt;/math&gt;
          &lt;/eventAssignment&gt;
        &lt;/listOfEventAssignments&gt;
      &lt;/event&gt;
      <event id="mwd85b986c_fd78_4938_9470_2135d267d058"
name="event_312">
        &lt;trigger&gt;
          &lt;math xmlns="http://www.w3.org/1998/Math/MathML"&gt;
            &lt;apply&gt;
              &lt;geq/&gt;
              <csymbol encoding="text"
definitionURL="http://www.sbml.org/sbml/symbols/time"> time &lt;/csymbol&gt;
              &lt;cn&gt; 369.8 &lt;/cn&gt;
```

```
          </apply>
        </math>
      </trigger>
      <listOfEventAssignments>
        <eventAssignment
variable="mw7d178aae_ff78_412a_830f_3be325d22773">
          <math xmlns="http://www.w3.org/1998/Math/MathML">
            <cn type="integer"> 1 </cn>
          </math>
        </eventAssignment>
      </listOfEventAssignments>
    </event>
    <event id="mw9cb86f1d_1fec_45da_a8aa_974512faa860"
name="event_313">
      <trigger>
        <math xmlns="http://www.w3.org/1998/Math/MathML">
          <apply>
            <geq/>
            <csymbol encoding="text"
definitionURL="http://www.sbml.org/sbml/symbols/time"> time </csymbol>
            <cn> 372.67 </cn>
          </apply>
        </math>
      </trigger>
      <listOfEventAssignments>
        <eventAssignment
variable="mw7d178aae_ff78_412a_830f_3be325d22773">
          <math xmlns="http://www.w3.org/1998/Math/MathML">
            <cn type="integer"> 0 </cn>
          </math>
        </eventAssignment>
      </listOfEventAssignments>
    </event>
    <event id="mw2a62f8b9_229d_415b_937f_9bfe593796c4"
name="event_314">
      <trigger>
        <math xmlns="http://www.w3.org/1998/Math/MathML">
          <apply>
            <geq/>
            <csymbol encoding="text"
definitionURL="http://www.sbml.org/sbml/symbols/time"> time </csymbol>
            <cn> 374.1 </cn>
          </apply>
        </math>
      </trigger>
      <listOfEventAssignments>
        <eventAssignment
variable="mw7d178aae_ff78_412a_830f_3be325d22773">
          <math xmlns="http://www.w3.org/1998/Math/MathML">
            <cn type="integer"> 1 </cn>
          </math>
        </eventAssignment>
```

```
        </listOfEventAssignments>
      </event>
      <event id="mw825ea0e8_a859_4df7_9b3a_7b64f982621e"
name="event_315">
        <trigger>
          <math xmlns="http://www.w3.org/1998/Math/MathML">
            <apply>
              <geq/>
              <csymbol encoding="text"
definitionURL="http://www.sbml.org/sbml/symbols/time"> time </csymbol>
              <cn> 376.97 </cn>
            </apply>
          </math>
        </trigger>
        <listOfEventAssignments>
          <eventAssignment
variable="mw7d178aae_ff78_412a_830f_3be325d22773">
            <math xmlns="http://www.w3.org/1998/Math/MathML">
              <cn type="integer"> 0 </cn>
            </math>
          </eventAssignment>
        </listOfEventAssignments>
      </event>
      <event id="mw0bb9a56d_0623_405b_9dff_f2f94cad599a"
name="event_316">
        <trigger>
          <math xmlns="http://www.w3.org/1998/Math/MathML">
            <apply>
              <geq/>
              <csymbol encoding="text"
definitionURL="http://www.sbml.org/sbml/symbols/time"> time </csymbol>
              <cn> 378.4 </cn>
            </apply>
          </math>
        </trigger>
        <listOfEventAssignments>
          <eventAssignment
variable="mw7d178aae_ff78_412a_830f_3be325d22773">
            <math xmlns="http://www.w3.org/1998/Math/MathML">
              <cn type="integer"> 1 </cn>
            </math>
          </eventAssignment>
        </listOfEventAssignments>
      </event>
      <event id="mw8b5e1873_3c85_45c7_abd1_8e7321f7a1a5"
name="event_317">
        <trigger>
          <math xmlns="http://www.w3.org/1998/Math/MathML">
            <apply>
              <geq/>
              <csymbol encoding="text"
definitionURL="http://www.sbml.org/sbml/symbols/time"> time </csymbol>
```

```
              <cn> 381.27 </cn>
            </apply>
          </math>
        </trigger>
        <listOfEventAssignments>
          <eventAssignment
variable="mw7d178aae_ff78_412a_830f_3be325d22773">
            <math xmlns="http://www.w3.org/1998/Math/MathML">
              <cn type="integer"> 0 </cn>
            </math>
          </eventAssignment>
        </listOfEventAssignments>
      </event>
      <event id="mw9ad0ac0a_2e62_447b_8934_8ff9b5a0cdd8"
name="event_318">
        <trigger>
          <math xmlns="http://www.w3.org/1998/Math/MathML">
            <apply>
              <geq/>
              <csymbol encoding="text"
definitionURL="http://www.sbml.org/sbml/symbols/time"> time </csymbol>
              <cn> 382.7 </cn>
            </apply>
          </math>
        </trigger>
        <listOfEventAssignments>
          <eventAssignment
variable="mw7d178aae_ff78_412a_830f_3be325d22773">
            <math xmlns="http://www.w3.org/1998/Math/MathML">
              <cn type="integer"> 1 </cn>
            </math>
          </eventAssignment>
        </listOfEventAssignments>
      </event>
      <event id="mw5b57362d_cce8_4eec_b7d2_6247f7f00ca1"
name="event_319">
        <trigger>
          <math xmlns="http://www.w3.org/1998/Math/MathML">
            <apply>
              <geq/>
              <csymbol encoding="text"
definitionURL="http://www.sbml.org/sbml/symbols/time"> time </csymbol>
              <cn> 385.57 </cn>
            </apply>
          </math>
        </trigger>
        <listOfEventAssignments>
          <eventAssignment
variable="mw7d178aae_ff78_412a_830f_3be325d22773">
            <math xmlns="http://www.w3.org/1998/Math/MathML">
              <cn type="integer"> 0 </cn>
            </math>
```

```
          </eventAssignment>
        </listOfEventAssignments>
      </event>
      <event id="mw04f758a0_836d_47ec_9828_07fd7e4f24d2"
name="event_320">
        <trigger>
          <math xmlns="http://www.w3.org/1998/Math/MathML">
            <apply>
              <geq/>
              <csymbol encoding="text"
definitionURL="http://www.sbml.org/sbml/symbols/time"> time </csymbol>
              <cn type="integer"> 387 </cn>
            </apply>
          </math>
        </trigger>
        <listOfEventAssignments>
          <eventAssignment
variable="mw7d178aae_ff78_412a_830f_3be325d22773">
            <math xmlns="http://www.w3.org/1998/Math/MathML">
              <cn type="integer"> 1 </cn>
            </math>
          </eventAssignment>
        </listOfEventAssignments>
      </event>
      <event id="mw11311b0e_af4d_48b0_b756_e4c0ce1c0cae"
name="event_321">
        <trigger>
          <math xmlns="http://www.w3.org/1998/Math/MathML">
            <apply>
              <geq/>
              <csymbol encoding="text"
definitionURL="http://www.sbml.org/sbml/symbols/time"> time </csymbol>
              <cn> 303.87 </cn>
            </apply>
          </math>
        </trigger>
        <listOfEventAssignments>
          <eventAssignment
variable="mwcedbe49e_2d28_4720_8fcd_207db64228cf">
            <math xmlns="http://www.w3.org/1998/Math/MathML">
              <cn type="integer"> 1 </cn>
            </math>
          </eventAssignment>
        </listOfEventAssignments>
      </event>
      <event id="mw8ba3cf97_de07_48f7_827d_0c5558de6d55"
name="event_322">
        <trigger>
          <math xmlns="http://www.w3.org/1998/Math/MathML">
            <apply>
              <geq/>
```

```
              <csymbol encoding="text"
definitionURL="http://www.sbml.org/sbml/symbols/time"> time </csymbol>
              <cn> 305.3 </cn>
            </apply>
          </math>
        </trigger>
        <listOfEventAssignments>
          <eventAssignment
variable="mwcedbe49e_2d28_4720_8fcd_207db64228cf">
            <math xmlns="http://www.w3.org/1998/Math/MathML">
              <cn type="integer"> 0 </cn>
            </math>
          </eventAssignment>
        </listOfEventAssignments>
      </event>
      <event id="mwe39d20c8_c32c_4642_8e4e_cc9bb2e15292"
name="event_323">
        <trigger>
          <math xmlns="http://www.w3.org/1998/Math/MathML">
            <apply>
              <geq/>
              <csymbol encoding="text"
definitionURL="http://www.sbml.org/sbml/symbols/time"> time </csymbol>
              <cn> 308.17 </cn>
            </apply>
          </math>
        </trigger>
        <listOfEventAssignments>
          <eventAssignment
variable="mwcedbe49e_2d28_4720_8fcd_207db64228cf">
            <math xmlns="http://www.w3.org/1998/Math/MathML">
              <cn type="integer"> 1 </cn>
            </math>
          </eventAssignment>
        </listOfEventAssignments>
      </event>
      <event id="mw1c2558ae_79c5_4da7_80b5_b3bf38001af8"
name="event_324">
        <trigger>
          <math xmlns="http://www.w3.org/1998/Math/MathML">
            <apply>
              <geq/>
              <csymbol encoding="text"
definitionURL="http://www.sbml.org/sbml/symbols/time"> time </csymbol>
              <cn> 309.6 </cn>
            </apply>
          </math>
        </trigger>
        <listOfEventAssignments>
          <eventAssignment
variable="mwcedbe49e_2d28_4720_8fcd_207db64228cf">
            <math xmlns="http://www.w3.org/1998/Math/MathML">
```

```
            &lt;cn type="integer"&gt; 0 &lt;/cn&gt;
          &lt;/math&gt;
        &lt;/eventAssignment&gt;
      &lt;/listOfEventAssignments&gt;
    &lt;/event&gt;
    <event id="mw0e3d9c45_5e92_4062_aa51_635093e3ecfc"
name="event_325">
      &lt;trigger&gt;
        &lt;math xmlns="http://www.w3.org/1998/Math/MathML"&gt;
          &lt;apply&gt;
            &lt;geq/&gt;
            <csymbol encoding="text"
definitionURL="http://www.sbml.org/sbml/symbols/time"> time &lt;/csymbol&gt;
            &lt;cn&gt; 312.47 &lt;/cn&gt;
          &lt;/apply&gt;
        &lt;/math&gt;
      &lt;/trigger&gt;
      &lt;listOfEventAssignments&gt;
        <eventAssignment
variable="mwcedbe49e_2d28_4720_8fcd_207db64228cf">
          &lt;math xmlns="http://www.w3.org/1998/Math/MathML"&gt;
            &lt;cn type="integer"&gt; 1 &lt;/cn&gt;
          &lt;/math&gt;
        &lt;/eventAssignment&gt;
      &lt;/listOfEventAssignments&gt;
    &lt;/event&gt;
    <event id="mw8e2dd940_e1cb_4d4c_b3db_f8d7f2d88a03"
name="event_326">
      &lt;trigger&gt;
        &lt;math xmlns="http://www.w3.org/1998/Math/MathML"&gt;
          &lt;apply&gt;
            &lt;geq/&gt;
            <csymbol encoding="text"
definitionURL="http://www.sbml.org/sbml/symbols/time"> time &lt;/csymbol&gt;
            &lt;cn&gt; 313.9 &lt;/cn&gt;
          &lt;/apply&gt;
        &lt;/math&gt;
      &lt;/trigger&gt;
      &lt;listOfEventAssignments&gt;
        <eventAssignment
variable="mwcedbe49e_2d28_4720_8fcd_207db64228cf">
          &lt;math xmlns="http://www.w3.org/1998/Math/MathML"&gt;
            &lt;cn type="integer"&gt; 0 &lt;/cn&gt;
          &lt;/math&gt;
        &lt;/eventAssignment&gt;
      &lt;/listOfEventAssignments&gt;
    &lt;/event&gt;
    <event id="mwbe46c140_71b5_470e_86d5_eed43187c613"
name="event_327">
      &lt;trigger&gt;
        &lt;math xmlns="http://www.w3.org/1998/Math/MathML"&gt;
          &lt;apply&gt;
```

```
              &lt;geq/&gt;
              <csymbol encoding="text"
definitionURL="http://www.sbml.org/sbml/symbols/time"> time &lt;/csymbol&gt;
              &lt;cn&gt; 316.77 &lt;/cn&gt;
            &lt;/apply&gt;
          &lt;/math&gt;
        &lt;/trigger&gt;
        &lt;listOfEventAssignments&gt;
          <eventAssignment
variable="mwcedbe49e_2d28_4720_8fcd_207db64228cf">
            &lt;math xmlns="http://www.w3.org/1998/Math/MathML"&gt;
              &lt;cn type="integer"&gt; 1 &lt;/cn&gt;
            &lt;/math&gt;
          &lt;/eventAssignment&gt;
        &lt;/listOfEventAssignments&gt;
      &lt;/event&gt;
      <event id="mw36f6576c_e82e_43bc_ac86_bd8d5e385cb1"
name="event_328">
        &lt;trigger&gt;
          &lt;math xmlns="http://www.w3.org/1998/Math/MathML"&gt;
            &lt;apply&gt;
              &lt;geq/&gt;
              <csymbol encoding="text"
definitionURL="http://www.sbml.org/sbml/symbols/time"> time &lt;/csymbol&gt;
              &lt;cn&gt; 318.2 &lt;/cn&gt;
            &lt;/apply&gt;
          &lt;/math&gt;
        &lt;/trigger&gt;
        &lt;listOfEventAssignments&gt;
          <eventAssignment
variable="mwcedbe49e_2d28_4720_8fcd_207db64228cf">
            &lt;math xmlns="http://www.w3.org/1998/Math/MathML"&gt;
              &lt;cn type="integer"&gt; 0 &lt;/cn&gt;
            &lt;/math&gt;
          &lt;/eventAssignment&gt;
        &lt;/listOfEventAssignments&gt;
      &lt;/event&gt;
      <event id="mw62fd2231_baff_457f_b15a_92d2a1df7810"
name="event_329">
        &lt;trigger&gt;
          &lt;math xmlns="http://www.w3.org/1998/Math/MathML"&gt;
            &lt;apply&gt;
              &lt;geq/&gt;
              <csymbol encoding="text"
definitionURL="http://www.sbml.org/sbml/symbols/time"> time &lt;/csymbol&gt;
              &lt;cn&gt; 321.07 &lt;/cn&gt;
            &lt;/apply&gt;
          &lt;/math&gt;
        &lt;/trigger&gt;
        &lt;listOfEventAssignments&gt;
          <eventAssignment
variable="mwcedbe49e_2d28_4720_8fcd_207db64228cf">
```

```
            <math xmlns="http://www.w3.org/1998/Math/MathML">
              <cn type="integer"> 1 </cn>
            </math>
          </eventAssignment>
        </listOfEventAssignments>
      </event>
      <event id="mwdd8a4070_c262_4646_adf1_1e37fc88374e"
name="event_330">
        <trigger>
          <math xmlns="http://www.w3.org/1998/Math/MathML">
            <apply>
              <geq/>
              <csymbol encoding="text"
definitionURL="http://www.sbml.org/sbml/symbols/time"> time </csymbol>
              <cn> 322.5 </cn>
            </apply>
          </math>
        </trigger>
        <listOfEventAssignments>
          <eventAssignment
variable="mwcedbe49e_2d28_4720_8fcd_207db64228cf">
            <math xmlns="http://www.w3.org/1998/Math/MathML">
              <cn type="integer"> 0 </cn>
            </math>
          </eventAssignment>
        </listOfEventAssignments>
      </event>
      <event id="mw5dec2638_6015_4595_abc8_b90d4cb5a96a"
name="event_331">
        <trigger>
          <math xmlns="http://www.w3.org/1998/Math/MathML">
            <apply>
              <geq/>
              <csymbol encoding="text"
definitionURL="http://www.sbml.org/sbml/symbols/time"> time </csymbol>
              <cn> 325.37 </cn>
            </apply>
          </math>
        </trigger>
        <listOfEventAssignments>
          <eventAssignment
variable="mwcedbe49e_2d28_4720_8fcd_207db64228cf">
            <math xmlns="http://www.w3.org/1998/Math/MathML">
              <cn type="integer"> 1 </cn>
            </math>
          </eventAssignment>
        </listOfEventAssignments>
      </event>
      <event id="mw01ae1f4c_4895_4a9a_bd06_a45869b6bc36"
name="event_332">
        <trigger>
          <math xmlns="http://www.w3.org/1998/Math/MathML">
```

```
            <apply>
              <geq/>
              <csymbol encoding="text"
definitionURL="http://www.sbml.org/sbml/symbols/time"> time </csymbol>
              <cn> 326.8 </cn>
            </apply>
          </math>
        </trigger>
        <listOfEventAssignments>
          <eventAssignment
variable="mwcedbe49e_2d28_4720_8fcd_207db64228cf">
            <math xmlns="http://www.w3.org/1998/Math/MathML">
              <cn type="integer"> 0 </cn>
            </math>
          </eventAssignment>
        </listOfEventAssignments>
      </event>
      <event id="mw6cc33d28_f37d_4754_a3ce_2f1addfa42be"
name="event_333">
        <trigger>
          <math xmlns="http://www.w3.org/1998/Math/MathML">
            <apply>
              <geq/>
              <csymbol encoding="text"
definitionURL="http://www.sbml.org/sbml/symbols/time"> time </csymbol>
              <cn> 329.67 </cn>
            </apply>
          </math>
        </trigger>
        <listOfEventAssignments>
          <eventAssignment
variable="mwcedbe49e_2d28_4720_8fcd_207db64228cf">
            <math xmlns="http://www.w3.org/1998/Math/MathML">
              <cn type="integer"> 1 </cn>
            </math>
          </eventAssignment>
        </listOfEventAssignments>
      </event>
      <event id="mw0f393e3c_4a7e_40fe_993e_302907f993dc"
name="event_334">
        <trigger>
          <math xmlns="http://www.w3.org/1998/Math/MathML">
            <apply>
              <geq/>
              <csymbol encoding="text"
definitionURL="http://www.sbml.org/sbml/symbols/time"> time </csymbol>
              <cn> 331.1 </cn>
            </apply>
          </math>
        </trigger>
        <listOfEventAssignments>
```

```
          <eventAssignment
variable="mwcedbe49e_2d28_4720_8fcd_207db64228cf">
            <math xmlns="http://www.w3.org/1998/Math/MathML">
              <cn type="integer"> 0 </cn>
            </math>
          </eventAssignment>
        </listOfEventAssignments>
      </event>
      <event id="mwd58e1fd3_7b97_473f_9e2f_f1ca6fda2456"
name="event_335">
        <trigger>
          <math xmlns="http://www.w3.org/1998/Math/MathML">
            <apply>
              <geq/>
              <csymbol encoding="text"
definitionURL="http://www.sbml.org/sbml/symbols/time"> time </csymbol>
              <cn> 333.97 </cn>
            </apply>
          </math>
        </trigger>
        <listOfEventAssignments>
          <eventAssignment
variable="mwcedbe49e_2d28_4720_8fcd_207db64228cf">
            <math xmlns="http://www.w3.org/1998/Math/MathML">
              <cn type="integer"> 1 </cn>
            </math>
          </eventAssignment>
        </listOfEventAssignments>
      </event>
      <event id="mw32289231_bf24_4e9e_84cf_63c82a35900c"
name="event_336">
        <trigger>
          <math xmlns="http://www.w3.org/1998/Math/MathML">
            <apply>
              <geq/>
              <csymbol encoding="text"
definitionURL="http://www.sbml.org/sbml/symbols/time"> time </csymbol>
              <cn> 335.4 </cn>
            </apply>
          </math>
        </trigger>
        <listOfEventAssignments>
          <eventAssignment
variable="mwcedbe49e_2d28_4720_8fcd_207db64228cf">
            <math xmlns="http://www.w3.org/1998/Math/MathML">
              <cn type="integer"> 0 </cn>
            </math>
          </eventAssignment>
        </listOfEventAssignments>
      </event>
      <event id="mwb7d05c82_a51e_4391_bda5_9ec0dfc21a68"
name="event_337">
```

```
        <trigger>
          <math xmlns="http://www.w3.org/1998/Math/MathML">
            <apply>
              <geq/>
              <csymbol encoding="text"
definitionURL="http://www.sbml.org/sbml/symbols/time"> time </csymbol>
              <cn> 338.27 </cn>
            </apply>
          </math>
        </trigger>
        <listOfEventAssignments>
          <eventAssignment
variable="mwcedbe49e_2d28_4720_8fcd_207db64228cf">
            <math xmlns="http://www.w3.org/1998/Math/MathML">
              <cn type="integer"> 1 </cn>
            </math>
          </eventAssignment>
        </listOfEventAssignments>
      </event>
      <event id="mw0034fac4_df88_45b2_b2e0_659491439e4c"
name="event_338">
        <trigger>
          <math xmlns="http://www.w3.org/1998/Math/MathML">
            <apply>
              <geq/>
              <csymbol encoding="text"
definitionURL="http://www.sbml.org/sbml/symbols/time"> time </csymbol>
              <cn> 339.7 </cn>
            </apply>
          </math>
        </trigger>
        <listOfEventAssignments>
          <eventAssignment
variable="mwcedbe49e_2d28_4720_8fcd_207db64228cf">
            <math xmlns="http://www.w3.org/1998/Math/MathML">
              <cn type="integer"> 0 </cn>
            </math>
          </eventAssignment>
        </listOfEventAssignments>
      </event>
      <event id="mw6cfb4af7_58b9_4022_a275_a85cf3872884"
name="event_339">
        <trigger>
          <math xmlns="http://www.w3.org/1998/Math/MathML">
            <apply>
              <geq/>
              <csymbol encoding="text"
definitionURL="http://www.sbml.org/sbml/symbols/time"> time </csymbol>
              <cn> 342.57 </cn>
            </apply>
          </math>
        </trigger>
```

```
        <listOfEventAssignments>
          <eventAssignment
variable="mwcedbe49e_2d28_4720_8fcd_207db64228cf">
            <math xmlns="http://www.w3.org/1998/Math/MathML">
              <cn type="integer"> 1 </cn>
            </math>
          </eventAssignment>
        </listOfEventAssignments>
      </event>
      <event id="mw8f47610f_3518_47b5_96e3_7215cf79b483"
name="event_340">
        <trigger>
          <math xmlns="http://www.w3.org/1998/Math/MathML">
            <apply>
              <geq/>
              <csymbol encoding="text"
definitionURL="http://www.sbml.org/sbml/symbols/time"> time </csymbol>
              <cn type="integer"> 344 </cn>
            </apply>
          </math>
        </trigger>
        <listOfEventAssignments>
          <eventAssignment
variable="mwcedbe49e_2d28_4720_8fcd_207db64228cf">
            <math xmlns="http://www.w3.org/1998/Math/MathML">
              <cn type="integer"> 0 </cn>
            </math>
          </eventAssignment>
        </listOfEventAssignments>
      </event>
      <event id="mw458106a5_9d53_4cac_9439_9f048c3d5743"
name="event_341">
        <trigger>
          <math xmlns="http://www.w3.org/1998/Math/MathML">
            <apply>
              <geq/>
              <csymbol encoding="text"
definitionURL="http://www.sbml.org/sbml/symbols/time"> time </csymbol>
              <cn> 346.87 </cn>
            </apply>
          </math>
        </trigger>
        <listOfEventAssignments>
          <eventAssignment
variable="mwcedbe49e_2d28_4720_8fcd_207db64228cf">
            <math xmlns="http://www.w3.org/1998/Math/MathML">
              <cn type="integer"> 1 </cn>
            </math>
          </eventAssignment>
        </listOfEventAssignments>
      </event>
```

```
      <event id="mw6c41422e_ba5c_4694_ac6f_fb751ccfa846"
name="event_342">
        <trigger>
          <math xmlns="http://www.w3.org/1998/Math/MathML">
            <apply>
              <geq/>
              <csymbol encoding="text"
definitionURL="http://www.sbml.org/sbml/symbols/time"> time </csymbol>
              <cn> 348.3 </cn>
            </apply>
          </math>
        </trigger>
        <listOfEventAssignments>
          <eventAssignment
variable="mwcedbe49e_2d28_4720_8fcd_207db64228cf">
            <math xmlns="http://www.w3.org/1998/Math/MathML">
              <cn type="integer"> 0 </cn>
            </math>
          </eventAssignment>
        </listOfEventAssignments>
      </event>
      <event id="mwb7223958_fbe3_44eb_996d_1cdc647b0bf0"
name="event_343">
        <trigger>
          <math xmlns="http://www.w3.org/1998/Math/MathML">
            <apply>
              <geq/>
              <csymbol encoding="text"
definitionURL="http://www.sbml.org/sbml/symbols/time"> time </csymbol>
              <cn> 351.17 </cn>
            </apply>
          </math>
        </trigger>
        <listOfEventAssignments>
          <eventAssignment
variable="mwcedbe49e_2d28_4720_8fcd_207db64228cf">
            <math xmlns="http://www.w3.org/1998/Math/MathML">
              <cn type="integer"> 1 </cn>
            </math>
          </eventAssignment>
        </listOfEventAssignments>
      </event>
      <event id="mw101fdbd8_0a63_412b_ab05_fed02337fe99"
name="event_344">
        <trigger>
          <math xmlns="http://www.w3.org/1998/Math/MathML">
            <apply>
              <geq/>
              <csymbol encoding="text"
definitionURL="http://www.sbml.org/sbml/symbols/time"> time </csymbol>
              <cn> 352.6 </cn>
            </apply>
```

```
          </math>
        </trigger>
        <listOfEventAssignments>
          <eventAssignment
variable="mwcedbe49e_2d28_4720_8fcd_207db64228cf">
            <math xmlns="http://www.w3.org/1998/Math/MathML">
              <cn type="integer"> 0 </cn>
            </math>
          </eventAssignment>
        </listOfEventAssignments>
      </event>
      <event id="mw76ef2e93_ecdc_45ff_a297_f15203cea9b5"
name="event_345">
        <trigger>
          <math xmlns="http://www.w3.org/1998/Math/MathML">
            <apply>
              <geq/>
              <csymbol encoding="text"
definitionURL="http://www.sbml.org/sbml/symbols/time"> time </csymbol>
              <cn> 355.47 </cn>
            </apply>
          </math>
        </trigger>
        <listOfEventAssignments>
          <eventAssignment
variable="mwcedbe49e_2d28_4720_8fcd_207db64228cf">
            <math xmlns="http://www.w3.org/1998/Math/MathML">
              <cn type="integer"> 1 </cn>
            </math>
          </eventAssignment>
        </listOfEventAssignments>
      </event>
      <event id="mw68207aff_5815_4761_aa63_dc1b672e3786"
name="event_346">
        <trigger>
          <math xmlns="http://www.w3.org/1998/Math/MathML">
            <apply>
              <geq/>
              <csymbol encoding="text"
definitionURL="http://www.sbml.org/sbml/symbols/time"> time </csymbol>
              <cn> 356.9 </cn>
            </apply>
          </math>
        </trigger>
        <listOfEventAssignments>
          <eventAssignment
variable="mwcedbe49e_2d28_4720_8fcd_207db64228cf">
            <math xmlns="http://www.w3.org/1998/Math/MathML">
              <cn type="integer"> 0 </cn>
            </math>
          </eventAssignment>
        </listOfEventAssignments>
```

```
      </event>
      <event id="mw15a06de0_69ca_4495_bb60_eff303fe01e8"
name="event_347">
        <trigger>
          <math xmlns="http://www.w3.org/1998/Math/MathML">
            <apply>
              <geq/>
              <csymbol encoding="text"
definitionURL="http://www.sbml.org/sbml/symbols/time"> time </csymbol>
              <cn> 359.77 </cn>
            </apply>
          </math>
        </trigger>
        <listOfEventAssignments>
          <eventAssignment
variable="mwcedbe49e_2d28_4720_8fcd_207db64228cf">
            <math xmlns="http://www.w3.org/1998/Math/MathML">
              <cn type="integer"> 1 </cn>
            </math>
          </eventAssignment>
        </listOfEventAssignments>
      </event>
      <event id="mwf5faddba_21c6_4898_92a4_45a21d230575"
name="event_348">
        <trigger>
          <math xmlns="http://www.w3.org/1998/Math/MathML">
            <apply>
              <geq/>
              <csymbol encoding="text"
definitionURL="http://www.sbml.org/sbml/symbols/time"> time </csymbol>
              <cn> 361.2 </cn>
            </apply>
          </math>
        </trigger>
        <listOfEventAssignments>
          <eventAssignment
variable="mwcedbe49e_2d28_4720_8fcd_207db64228cf">
            <math xmlns="http://www.w3.org/1998/Math/MathML">
              <cn type="integer"> 0 </cn>
            </math>
          </eventAssignment>
        </listOfEventAssignments>
      </event>
      <event id="mw89004e19_d193_400a_9085_e8aa9e2ddc50"
name="event_349">
        <trigger>
          <math xmlns="http://www.w3.org/1998/Math/MathML">
            <apply>
              <geq/>
              <csymbol encoding="text"
definitionURL="http://www.sbml.org/sbml/symbols/time"> time </csymbol>
              <cn> 364.07 </cn>
```

```
          &lt;/apply&gt;
        &lt;/math&gt;
      &lt;/trigger&gt;
      &lt;listOfEventAssignments&gt;
        <eventAssignment
variable="mwcedbe49e_2d28_4720_8fcd_207db64228cf">
          &lt;math xmlns="http://www.w3.org/1998/Math/MathML"&gt;
            &lt;cn type="integer"&gt; 1 &lt;/cn&gt;
          &lt;/math&gt;
        &lt;/eventAssignment&gt;
      &lt;/listOfEventAssignments&gt;
    &lt;/event&gt;
    <event id="mwb8f02112_d0a8_460e_8052_59d6d3f47758"
name="event_350">
      &lt;trigger&gt;
        &lt;math xmlns="http://www.w3.org/1998/Math/MathML"&gt;
          &lt;apply&gt;
            &lt;geq/&gt;
            <csymbol encoding="text"
definitionURL="http://www.sbml.org/sbml/symbols/time"> time &lt;/csymbol&gt;
            &lt;cn&gt; 365.5 &lt;/cn&gt;
          &lt;/apply&gt;
        &lt;/math&gt;
      &lt;/trigger&gt;
      &lt;listOfEventAssignments&gt;
        <eventAssignment
variable="mwcedbe49e_2d28_4720_8fcd_207db64228cf">
          &lt;math xmlns="http://www.w3.org/1998/Math/MathML"&gt;
            &lt;cn type="integer"&gt; 0 &lt;/cn&gt;
          &lt;/math&gt;
        &lt;/eventAssignment&gt;
      &lt;/listOfEventAssignments&gt;
    &lt;/event&gt;
    <event id="mweb897173_4307_4a5a_9831_c97741b13845"
name="event_351">
      &lt;trigger&gt;
        &lt;math xmlns="http://www.w3.org/1998/Math/MathML"&gt;
          &lt;apply&gt;
            &lt;geq/&gt;
            <csymbol encoding="text"
definitionURL="http://www.sbml.org/sbml/symbols/time"> time &lt;/csymbol&gt;
            &lt;cn&gt; 368.37 &lt;/cn&gt;
          &lt;/apply&gt;
        &lt;/math&gt;
      &lt;/trigger&gt;
      &lt;listOfEventAssignments&gt;
        <eventAssignment
variable="mwcedbe49e_2d28_4720_8fcd_207db64228cf">
          &lt;math xmlns="http://www.w3.org/1998/Math/MathML"&gt;
            &lt;cn type="integer"&gt; 1 &lt;/cn&gt;
          &lt;/math&gt;
        &lt;/eventAssignment&gt;
```

```
        &lt;/listOfEventAssignments&gt;
      &lt;/event&gt;
      <event id="mw65c09402_620e_4a86_9b41_b452c7c01658"
name="event_352">
        &lt;trigger&gt;
          &lt;math xmlns="http://www.w3.org/1998/Math/MathML"&gt;
            &lt;apply&gt;
              &lt;geq/&gt;
              <csymbol encoding="text"
definitionURL="http://www.sbml.org/sbml/symbols/time"> time &lt;/csymbol&gt;
              &lt;cn&gt; 369.8 &lt;/cn&gt;
            &lt;/apply&gt;
          &lt;/math&gt;
        &lt;/trigger&gt;
        &lt;listOfEventAssignments&gt;
          <eventAssignment
variable="mwcedbe49e_2d28_4720_8fcd_207db64228cf">
            &lt;math xmlns="http://www.w3.org/1998/Math/MathML"&gt;
              &lt;cn type="integer"&gt; 0 &lt;/cn&gt;
            &lt;/math&gt;
          &lt;/eventAssignment&gt;
        &lt;/listOfEventAssignments&gt;
      &lt;/event&gt;
      <event id="mw9fed03b4_143a_45cc_b3a8_3366133b9a67"
name="event_353">
        &lt;trigger&gt;
          &lt;math xmlns="http://www.w3.org/1998/Math/MathML"&gt;
            &lt;apply&gt;
              &lt;geq/&gt;
              <csymbol encoding="text"
definitionURL="http://www.sbml.org/sbml/symbols/time"> time &lt;/csymbol&gt;
              &lt;cn&gt; 372.67 &lt;/cn&gt;
            &lt;/apply&gt;
          &lt;/math&gt;
        &lt;/trigger&gt;
        &lt;listOfEventAssignments&gt;
          <eventAssignment
variable="mwcedbe49e_2d28_4720_8fcd_207db64228cf">
            &lt;math xmlns="http://www.w3.org/1998/Math/MathML"&gt;
              &lt;cn type="integer"&gt; 1 &lt;/cn&gt;
            &lt;/math&gt;
          &lt;/eventAssignment&gt;
        &lt;/listOfEventAssignments&gt;
      &lt;/event&gt;
      <event id="mwf861ba25_0f28_451d_88e2_e0dfbea76ee4"
name="event_354">
        &lt;trigger&gt;
          &lt;math xmlns="http://www.w3.org/1998/Math/MathML"&gt;
            &lt;apply&gt;
              &lt;geq/&gt;
              <csymbol encoding="text"
definitionURL="http://www.sbml.org/sbml/symbols/time"> time &lt;/csymbol&gt;
```

```
              <cn> 374.1 </cn>
            </apply>
          </math>
        </trigger>
        <listOfEventAssignments>
          <eventAssignment
variable="mwcedbe49e_2d28_4720_8fcd_207db64228cf">
            <math xmlns="http://www.w3.org/1998/Math/MathML">
              <cn type="integer"> 0 </cn>
            </math>
          </eventAssignment>
        </listOfEventAssignments>
      </event>
      <event id="mw7abccc98_fdb5_433d_a758_61627ed331d8"
name="event_355">
        <trigger>
          <math xmlns="http://www.w3.org/1998/Math/MathML">
            <apply>
              <geq/>
              <csymbol encoding="text"
definitionURL="http://www.sbml.org/sbml/symbols/time"> time </csymbol>
              <cn> 376.97 </cn>
            </apply>
          </math>
        </trigger>
        <listOfEventAssignments>
          <eventAssignment
variable="mwcedbe49e_2d28_4720_8fcd_207db64228cf">
            <math xmlns="http://www.w3.org/1998/Math/MathML">
              <cn type="integer"> 1 </cn>
            </math>
          </eventAssignment>
        </listOfEventAssignments>
      </event>
      <event id="mwa91f48e4_6b41_4c7e_83dc_3463fa76154f"
name="event_356">
        <trigger>
          <math xmlns="http://www.w3.org/1998/Math/MathML">
            <apply>
              <geq/>
              <csymbol encoding="text"
definitionURL="http://www.sbml.org/sbml/symbols/time"> time </csymbol>
              <cn> 378.4 </cn>
            </apply>
          </math>
        </trigger>
        <listOfEventAssignments>
          <eventAssignment
variable="mwcedbe49e_2d28_4720_8fcd_207db64228cf">
            <math xmlns="http://www.w3.org/1998/Math/MathML">
              <cn type="integer"> 0 </cn>
            </math>
```

```
          </eventAssignment>
        </listOfEventAssignments>
      </event>
      <event id="mw575fe735_a4ee_4097_967d_04dc9db28f7b"
name="event_357">
        <trigger>
          <math xmlns="http://www.w3.org/1998/Math/MathML">
            <apply>
              <geq/>
              <csymbol encoding="text"
definitionURL="http://www.sbml.org/sbml/symbols/time"> time </csymbol>
              <cn> 381.27 </cn>
            </apply>
          </math>
        </trigger>
        <listOfEventAssignments>
          <eventAssignment
variable="mwcedbe49e_2d28_4720_8fcd_207db64228cf">
            <math xmlns="http://www.w3.org/1998/Math/MathML">
              <cn type="integer"> 1 </cn>
            </math>
          </eventAssignment>
        </listOfEventAssignments>
      </event>
      <event id="mwe4d72a98_781a_44aa_823c_105261d00c23"
name="event_358">
        <trigger>
          <math xmlns="http://www.w3.org/1998/Math/MathML">
            <apply>
              <geq/>
              <csymbol encoding="text"
definitionURL="http://www.sbml.org/sbml/symbols/time"> time </csymbol>
              <cn> 382.7 </cn>
            </apply>
          </math>
        </trigger>
        <listOfEventAssignments>
          <eventAssignment
variable="mwcedbe49e_2d28_4720_8fcd_207db64228cf">
            <math xmlns="http://www.w3.org/1998/Math/MathML">
              <cn type="integer"> 0 </cn>
            </math>
          </eventAssignment>
        </listOfEventAssignments>
      </event>
      <event id="mw523be0a9_df0c_41e8_8748_e4f6b4276051"
name="event_359">
        <trigger>
          <math xmlns="http://www.w3.org/1998/Math/MathML">
            <apply>
              <geq/>
```

```
              <csymbol encoding="text" 
definitionURL="http://www.sbml.org/sbml/symbols/time"> time &lt;/csymbol&gt;
              &lt;cn&gt; 385.57 &lt;/cn&gt;
            &lt;/apply&gt;
          &lt;/math&gt;
        &lt;/trigger&gt;
        &lt;listOfEventAssignments&gt;
          <eventAssignment 
variable="mwcedbe49e_2d28_4720_8fcd_207db64228cf">
            &lt;math xmlns="http://www.w3.org/1998/Math/MathML"&gt;
              &lt;cn type="integer"&gt; 1 &lt;/cn&gt;
            &lt;/math&gt;
          &lt;/eventAssignment&gt;
        &lt;/listOfEventAssignments&gt;
      &lt;/event&gt;
      <event id="mwb6f63b51_d845_419e_952c_f0d4029a4cad" 
name="event_360">
        &lt;trigger&gt;
          &lt;math xmlns="http://www.w3.org/1998/Math/MathML"&gt;
            &lt;apply&gt;
              &lt;geq/&gt;
              <csymbol encoding="text" 
definitionURL="http://www.sbml.org/sbml/symbols/time"> time &lt;/csymbol&gt;
              &lt;cn type="integer"&gt; 387 &lt;/cn&gt;
            &lt;/apply&gt;
          &lt;/math&gt;
        &lt;/trigger&gt;
        &lt;listOfEventAssignments&gt;
          <eventAssignment 
variable="mwcedbe49e_2d28_4720_8fcd_207db64228cf">
            &lt;math xmlns="http://www.w3.org/1998/Math/MathML"&gt;
              &lt;cn type="integer"&gt; 0 &lt;/cn&gt;
            &lt;/math&gt;
          &lt;/eventAssignment&gt;
        &lt;/listOfEventAssignments&gt;
      &lt;/event&gt;
      <event id="mw8c9b1244_91db_4b42_b391_2c37d247c757" 
name="event_361">
        &lt;trigger&gt;
          &lt;math xmlns="http://www.w3.org/1998/Math/MathML"&gt;
            &lt;apply&gt;
              &lt;geq/&gt;
              <csymbol encoding="text" 
definitionURL="http://www.sbml.org/sbml/symbols/time"> time &lt;/csymbol&gt;
              &lt;cn&gt; 389.87 &lt;/cn&gt;
            &lt;/apply&gt;
          &lt;/math&gt;
        &lt;/trigger&gt;
        &lt;listOfEventAssignments&gt;
          <eventAssignment 
variable="mw7d178aae_ff78_412a_830f_3be325d22773">
            &lt;math xmlns="http://www.w3.org/1998/Math/MathML"&gt;
```

```
            &lt;cn type="integer"&gt; 0 &lt;/cn&gt;
          &lt;/math&gt;
        &lt;/eventAssignment&gt;
      &lt;/listOfEventAssignments&gt;
    &lt;/event&gt;
    <event id="mwa41a3e2b_f048_4cb1_b379_880239c1f8df"
name="event_362">
      &lt;trigger&gt;
        &lt;math xmlns="http://www.w3.org/1998/Math/MathML"&gt;
          &lt;apply&gt;
            &lt;geq/&gt;
            <csymbol encoding="text"
definitionURL="http://www.sbml.org/sbml/symbols/time"> time &lt;/csymbol&gt;
            &lt;cn&gt; 391.3 &lt;/cn&gt;
          &lt;/apply&gt;
        &lt;/math&gt;
      &lt;/trigger&gt;
      &lt;listOfEventAssignments&gt;
        <eventAssignment
variable="mw7d178aae_ff78_412a_830f_3be325d22773">
          &lt;math xmlns="http://www.w3.org/1998/Math/MathML"&gt;
            &lt;cn type="integer"&gt; 1 &lt;/cn&gt;
          &lt;/math&gt;
        &lt;/eventAssignment&gt;
      &lt;/listOfEventAssignments&gt;
    &lt;/event&gt;
    <event id="mw8f3c4a31_d663_4808_b6cb_1bc559df31c0"
name="event_363">
      &lt;trigger&gt;
        &lt;math xmlns="http://www.w3.org/1998/Math/MathML"&gt;
          &lt;apply&gt;
            &lt;geq/&gt;
            <csymbol encoding="text"
definitionURL="http://www.sbml.org/sbml/symbols/time"> time &lt;/csymbol&gt;
            &lt;cn&gt; 394.17 &lt;/cn&gt;
          &lt;/apply&gt;
        &lt;/math&gt;
      &lt;/trigger&gt;
      &lt;listOfEventAssignments&gt;
        <eventAssignment
variable="mw7d178aae_ff78_412a_830f_3be325d22773">
          &lt;math xmlns="http://www.w3.org/1998/Math/MathML"&gt;
            &lt;cn type="integer"&gt; 0 &lt;/cn&gt;
          &lt;/math&gt;
        &lt;/eventAssignment&gt;
      &lt;/listOfEventAssignments&gt;
    &lt;/event&gt;
    <event id="mw1d52a9fc_90d4_45de_97df_d1e66151226c"
name="event_364">
      &lt;trigger&gt;
        &lt;math xmlns="http://www.w3.org/1998/Math/MathML"&gt;
          &lt;apply&gt;
```

```
            <geq/>
            <csymbol encoding="text"
definitionURL="http://www.sbml.org/sbml/symbols/time"> time </csymbol>
            <cn> 395.6 </cn>
          </apply>
        </math>
      </trigger>
      <listOfEventAssignments>
        <eventAssignment
variable="mw7d178aae_ff78_412a_830f_3be325d22773">
          <math xmlns="http://www.w3.org/1998/Math/MathML">
            <cn type="integer"> 1 </cn>
          </math>
        </eventAssignment>
      </listOfEventAssignments>
    </event>
    <event id="mw35e1460d_daff_4f42_ae9c_bc7907e610bb"
name="event_365">
      <trigger>
        <math xmlns="http://www.w3.org/1998/Math/MathML">
          <apply>
            <geq/>
            <csymbol encoding="text"
definitionURL="http://www.sbml.org/sbml/symbols/time"> time </csymbol>
            <cn> 398.47 </cn>
          </apply>
        </math>
      </trigger>
      <listOfEventAssignments>
        <eventAssignment
variable="mw7d178aae_ff78_412a_830f_3be325d22773">
          <math xmlns="http://www.w3.org/1998/Math/MathML">
            <cn type="integer"> 0 </cn>
          </math>
        </eventAssignment>
      </listOfEventAssignments>
    </event>
    <event id="mw7e56c9d0_33d6_4d83_8385_fbd40c76b60f"
name="event_366">
      <trigger>
        <math xmlns="http://www.w3.org/1998/Math/MathML">
          <apply>
            <geq/>
            <csymbol encoding="text"
definitionURL="http://www.sbml.org/sbml/symbols/time"> time </csymbol>
            <cn> 399.9 </cn>
          </apply>
        </math>
      </trigger>
      <listOfEventAssignments>
        <eventAssignment
variable="mw7d178aae_ff78_412a_830f_3be325d22773">
```

```
            <math xmlns="http://www.w3.org/1998/Math/MathML">
              <cn type="integer"> 1 </cn>
            </math>
          </eventAssignment>
        </listOfEventAssignments>
      </event>
      <event id="mwb1b8abcf_ed51_44b7_bde3_a1b7bf0c4943"
name="event_367">
        <trigger>
          <math xmlns="http://www.w3.org/1998/Math/MathML">
            <apply>
              <geq/>
              <csymbol encoding="text"
definitionURL="http://www.sbml.org/sbml/symbols/time"> time </csymbol>
              <cn> 402.77 </cn>
            </apply>
          </math>
        </trigger>
        <listOfEventAssignments>
          <eventAssignment
variable="mw7d178aae_ff78_412a_830f_3be325d22773">
            <math xmlns="http://www.w3.org/1998/Math/MathML">
              <cn type="integer"> 0 </cn>
            </math>
          </eventAssignment>
        </listOfEventAssignments>
      </event>
      <event id="mwb755c879_0875_4673_9fbe_7bf4d4ff2bf3"
name="event_368">
        <trigger>
          <math xmlns="http://www.w3.org/1998/Math/MathML">
            <apply>
              <geq/>
              <csymbol encoding="text"
definitionURL="http://www.sbml.org/sbml/symbols/time"> time </csymbol>
              <cn> 404.2 </cn>
            </apply>
          </math>
        </trigger>
        <listOfEventAssignments>
          <eventAssignment
variable="mw7d178aae_ff78_412a_830f_3be325d22773">
            <math xmlns="http://www.w3.org/1998/Math/MathML">
              <cn type="integer"> 1 </cn>
            </math>
          </eventAssignment>
        </listOfEventAssignments>
      </event>
      <event id="mw3f211869_aa94_4492_a299_e21e66dfefff"
name="event_369">
        <trigger>
          <math xmlns="http://www.w3.org/1998/Math/MathML">
```

```
            <apply>
              <geq/>
              <csymbol encoding="text"
definitionURL="http://www.sbml.org/sbml/symbols/time"> time </csymbol>
              <cn> 407.07 </cn>
            </apply>
          </math>
        </trigger>
        <listOfEventAssignments>
          <eventAssignment
variable="mw7d178aae_ff78_412a_830f_3be325d22773">
            <math xmlns="http://www.w3.org/1998/Math/MathML">
              <cn type="integer"> 0 </cn>
            </math>
          </eventAssignment>
        </listOfEventAssignments>
      </event>
      <event id="mw298ef628_a43d_4302_80f3_a0a88ab9ee92"
name="event_370">
        <trigger>
          <math xmlns="http://www.w3.org/1998/Math/MathML">
            <apply>
              <geq/>
              <csymbol encoding="text"
definitionURL="http://www.sbml.org/sbml/symbols/time"> time </csymbol>
              <cn> 408.5 </cn>
            </apply>
          </math>
        </trigger>
        <listOfEventAssignments>
          <eventAssignment
variable="mw7d178aae_ff78_412a_830f_3be325d22773">
            <math xmlns="http://www.w3.org/1998/Math/MathML">
              <cn type="integer"> 1 </cn>
            </math>
          </eventAssignment>
        </listOfEventAssignments>
      </event>
      <event id="mw64a87998_4264_468e_bcb8_a7e3f29a7cd9"
name="event_371">
        <trigger>
          <math xmlns="http://www.w3.org/1998/Math/MathML">
            <apply>
              <geq/>
              <csymbol encoding="text"
definitionURL="http://www.sbml.org/sbml/symbols/time"> time </csymbol>
              <cn> 411.37 </cn>
            </apply>
          </math>
        </trigger>
        <listOfEventAssignments>
```

```
          <eventAssignment
variable="mw7d178aae_ff78_412a_830f_3be325d22773">
            <math xmlns="http://www.w3.org/1998/Math/MathML">
              <cn type="integer"> 0 </cn>
            </math>
          </eventAssignment>
        </listOfEventAssignments>
      </event>
      <event id="mw1b230616_2cd3_49b2_bda3_37c09185c019"
name="event_372">
        <trigger>
          <math xmlns="http://www.w3.org/1998/Math/MathML">
            <apply>
              <geq/>
              <csymbol encoding="text"
definitionURL="http://www.sbml.org/sbml/symbols/time"> time </csymbol>
              <cn> 412.8 </cn>
            </apply>
          </math>
        </trigger>
        <listOfEventAssignments>
          <eventAssignment
variable="mw7d178aae_ff78_412a_830f_3be325d22773">
            <math xmlns="http://www.w3.org/1998/Math/MathML">
              <cn type="integer"> 1 </cn>
            </math>
          </eventAssignment>
        </listOfEventAssignments>
      </event>
      <event id="mwd559e5c6_b441_4419_862c_834dd216d185"
name="event_373">
        <trigger>
          <math xmlns="http://www.w3.org/1998/Math/MathML">
            <apply>
              <geq/>
              <csymbol encoding="text"
definitionURL="http://www.sbml.org/sbml/symbols/time"> time </csymbol>
              <cn> 415.67 </cn>
            </apply>
          </math>
        </trigger>
        <listOfEventAssignments>
          <eventAssignment
variable="mw7d178aae_ff78_412a_830f_3be325d22773">
            <math xmlns="http://www.w3.org/1998/Math/MathML">
              <cn type="integer"> 0 </cn>
            </math>
          </eventAssignment>
        </listOfEventAssignments>
      </event>
      <event id="mwaab2a40c_f581_4093_a2d1_b1c0bace80e5"
name="event_374">
```

```
        <trigger>
          <math xmlns="http://www.w3.org/1998/Math/MathML">
            <apply>
              <geq/>
              <csymbol encoding="text"
definitionURL="http://www.sbml.org/sbml/symbols/time"> time </csymbol>
              <cn> 417.1 </cn>
            </apply>
          </math>
        </trigger>
        <listOfEventAssignments>
          <eventAssignment
variable="mw7d178aae_ff78_412a_830f_3be325d22773">
            <math xmlns="http://www.w3.org/1998/Math/MathML">
              <cn type="integer"> 1 </cn>
            </math>
          </eventAssignment>
        </listOfEventAssignments>
      </event>
      <event id="mw7f413f1f_2182_4a97_b38c_8eefa3b70bbf"
name="event_375">
        <trigger>
          <math xmlns="http://www.w3.org/1998/Math/MathML">
            <apply>
              <geq/>
              <csymbol encoding="text"
definitionURL="http://www.sbml.org/sbml/symbols/time"> time </csymbol>
              <cn> 419.97 </cn>
            </apply>
          </math>
        </trigger>
        <listOfEventAssignments>
          <eventAssignment
variable="mw7d178aae_ff78_412a_830f_3be325d22773">
            <math xmlns="http://www.w3.org/1998/Math/MathML">
              <cn type="integer"> 0 </cn>
            </math>
          </eventAssignment>
        </listOfEventAssignments>
      </event>
      <event id="mw4404c6e6_e96b_484d_a2c8_aff265b0669b"
name="event_376">
        <trigger>
          <math xmlns="http://www.w3.org/1998/Math/MathML">
            <apply>
              <geq/>
              <csymbol encoding="text"
definitionURL="http://www.sbml.org/sbml/symbols/time"> time </csymbol>
              <cn> 421.4 </cn>
            </apply>
          </math>
        </trigger>
```

```
        &lt;listOfEventAssignments&gt;
          <eventAssignment
variable="mw7d178aae_ff78_412a_830f_3be325d22773">
            &lt;math xmlns="http://www.w3.org/1998/Math/MathML"&gt;
              &lt;cn type="integer"&gt; 1 &lt;/cn&gt;
            &lt;/math&gt;
          &lt;/eventAssignment&gt;
        &lt;/listOfEventAssignments&gt;
      &lt;/event&gt;
      <event id="mw1deb2e15_5922_4034_ab52_8d30ad244720"
name="event_377">
        &lt;trigger&gt;
          &lt;math xmlns="http://www.w3.org/1998/Math/MathML"&gt;
            &lt;apply&gt;
              &lt;geq/&gt;
              <csymbol encoding="text"
definitionURL="http://www.sbml.org/sbml/symbols/time"> time &lt;/csymbol&gt;
              &lt;cn&gt; 424.27 &lt;/cn&gt;
            &lt;/apply&gt;
          &lt;/math&gt;
        &lt;/trigger&gt;
        &lt;listOfEventAssignments&gt;
          <eventAssignment
variable="mw7d178aae_ff78_412a_830f_3be325d22773">
            &lt;math xmlns="http://www.w3.org/1998/Math/MathML"&gt;
              &lt;cn type="integer"&gt; 0 &lt;/cn&gt;
            &lt;/math&gt;
          &lt;/eventAssignment&gt;
        &lt;/listOfEventAssignments&gt;
      &lt;/event&gt;
      <event id="mwd7a8394e_9b66_4b33_be40_8fca4540eb69"
name="event_378">
        &lt;trigger&gt;
          &lt;math xmlns="http://www.w3.org/1998/Math/MathML"&gt;
            &lt;apply&gt;
              &lt;geq/&gt;
              <csymbol encoding="text"
definitionURL="http://www.sbml.org/sbml/symbols/time"> time &lt;/csymbol&gt;
              &lt;cn&gt; 425.7 &lt;/cn&gt;
            &lt;/apply&gt;
          &lt;/math&gt;
        &lt;/trigger&gt;
        &lt;listOfEventAssignments&gt;
          <eventAssignment
variable="mw7d178aae_ff78_412a_830f_3be325d22773">
            &lt;math xmlns="http://www.w3.org/1998/Math/MathML"&gt;
              &lt;cn type="integer"&gt; 1 &lt;/cn&gt;
            &lt;/math&gt;
          &lt;/eventAssignment&gt;
        &lt;/listOfEventAssignments&gt;
      &lt;/event&gt;
```

```
      <event id="mw71f779b3_b905_4176_886e_a2574bfd5d35"
name="event_379">
        &lt;trigger&gt;
          &lt;math xmlns="http://www.w3.org/1998/Math/MathML"&gt;
            &lt;apply&gt;
              &lt;geq/&gt;
              <csymbol encoding="text"
definitionURL="http://www.sbml.org/sbml/symbols/time"> time &lt;/csymbol&gt;
              &lt;cn&gt; 428.57 &lt;/cn&gt;
            &lt;/apply&gt;
          &lt;/math&gt;
        &lt;/trigger&gt;
        &lt;listOfEventAssignments&gt;
          <eventAssignment
variable="mw7d178aae_ff78_412a_830f_3be325d22773">
            &lt;math xmlns="http://www.w3.org/1998/Math/MathML"&gt;
              &lt;cn type="integer"&gt; 0 &lt;/cn&gt;
            &lt;/math&gt;
          &lt;/eventAssignment&gt;
        &lt;/listOfEventAssignments&gt;
      &lt;/event&gt;
      <event id="mw0c859876_2f0f_4def_9ccf_f2d5eab73c03"
name="event_380">
        &lt;trigger&gt;
          &lt;math xmlns="http://www.w3.org/1998/Math/MathML"&gt;
            &lt;apply&gt;
              &lt;geq/&gt;
              <csymbol encoding="text"
definitionURL="http://www.sbml.org/sbml/symbols/time"> time &lt;/csymbol&gt;
              &lt;cn type="integer"&gt; 430 &lt;/cn&gt;
            &lt;/apply&gt;
          &lt;/math&gt;
        &lt;/trigger&gt;
        &lt;listOfEventAssignments&gt;
          <eventAssignment
variable="mw7d178aae_ff78_412a_830f_3be325d22773">
            &lt;math xmlns="http://www.w3.org/1998/Math/MathML"&gt;
              &lt;cn type="integer"&gt; 1 &lt;/cn&gt;
            &lt;/math&gt;
          &lt;/eventAssignment&gt;
        &lt;/listOfEventAssignments&gt;
      &lt;/event&gt;
      <event id="mw638ed258_310a_4407_8e61_1f8679074634"
name="event_381">
        &lt;trigger&gt;
          &lt;math xmlns="http://www.w3.org/1998/Math/MathML"&gt;
            &lt;apply&gt;
              &lt;geq/&gt;
              <csymbol encoding="text"
definitionURL="http://www.sbml.org/sbml/symbols/time"> time &lt;/csymbol&gt;
              &lt;cn&gt; 432.87 &lt;/cn&gt;
            &lt;/apply&gt;
```

```
          </math>
        </trigger>
        <listOfEventAssignments>
          <eventAssignment
variable="mw7d178aae_ff78_412a_830f_3be325d22773">
            <math xmlns="http://www.w3.org/1998/Math/MathML">
              <cn type="integer"> 0 </cn>
            </math>
          </eventAssignment>
        </listOfEventAssignments>
      </event>
      <event id="mw0c392545_ac4c_4cec_9e15_57cc9f8f8f8d"
name="event_382">
        <trigger>
          <math xmlns="http://www.w3.org/1998/Math/MathML">
            <apply>
              <geq/>
              <csymbol encoding="text"
definitionURL="http://www.sbml.org/sbml/symbols/time"> time </csymbol>
              <cn> 434.3 </cn>
            </apply>
          </math>
        </trigger>
        <listOfEventAssignments>
          <eventAssignment
variable="mw7d178aae_ff78_412a_830f_3be325d22773">
            <math xmlns="http://www.w3.org/1998/Math/MathML">
              <cn type="integer"> 1 </cn>
            </math>
          </eventAssignment>
        </listOfEventAssignments>
      </event>
      <event id="mwbbe3859b_697b_4864_a178_2e79c235bac8"
name="event_383">
        <trigger>
          <math xmlns="http://www.w3.org/1998/Math/MathML">
            <apply>
              <geq/>
              <csymbol encoding="text"
definitionURL="http://www.sbml.org/sbml/symbols/time"> time </csymbol>
              <cn> 437.17 </cn>
            </apply>
          </math>
        </trigger>
        <listOfEventAssignments>
          <eventAssignment
variable="mw7d178aae_ff78_412a_830f_3be325d22773">
            <math xmlns="http://www.w3.org/1998/Math/MathML">
              <cn type="integer"> 0 </cn>
            </math>
          </eventAssignment>
        </listOfEventAssignments>
```

```
      </event>
      <event id="mw3fccef24_69ae_4904_a0ef_bd87c954fd3a"
name="event_384">
        <trigger>
          <math xmlns="http://www.w3.org/1998/Math/MathML">
            <apply>
              <geq/>
              <csymbol encoding="text"
definitionURL="http://www.sbml.org/sbml/symbols/time"> time </csymbol>
              <cn> 438.6 </cn>
            </apply>
          </math>
        </trigger>
        <listOfEventAssignments>
          <eventAssignment
variable="mw7d178aae_ff78_412a_830f_3be325d22773">
            <math xmlns="http://www.w3.org/1998/Math/MathML">
              <cn type="integer"> 1 </cn>
            </math>
          </eventAssignment>
        </listOfEventAssignments>
      </event>
      <event id="mwffc2756c_834c_473a_81e8_15b892c74164"
name="event_385">
        <trigger>
          <math xmlns="http://www.w3.org/1998/Math/MathML">
            <apply>
              <geq/>
              <csymbol encoding="text"
definitionURL="http://www.sbml.org/sbml/symbols/time"> time </csymbol>
              <cn> 441.47 </cn>
            </apply>
          </math>
        </trigger>
        <listOfEventAssignments>
          <eventAssignment
variable="mw7d178aae_ff78_412a_830f_3be325d22773">
            <math xmlns="http://www.w3.org/1998/Math/MathML">
              <cn type="integer"> 0 </cn>
            </math>
          </eventAssignment>
        </listOfEventAssignments>
      </event>
      <event id="mw3147801e_7a80_49f7_af76_0c9e6bfd6195"
name="event_386">
        <trigger>
          <math xmlns="http://www.w3.org/1998/Math/MathML">
            <apply>
              <geq/>
              <csymbol encoding="text"
definitionURL="http://www.sbml.org/sbml/symbols/time"> time </csymbol>
              <cn> 442.9 </cn>
```

```
          </apply>
        </math>
      </trigger>
      <listOfEventAssignments>
        <eventAssignment
variable="mw7d178aae_ff78_412a_830f_3be325d22773">
          <math xmlns="http://www.w3.org/1998/Math/MathML">
            <cn type="integer"> 1 </cn>
          </math>
        </eventAssignment>
      </listOfEventAssignments>
    </event>
    <event id="mw1a66d83a_88f2_46c6_ba2d_78949b72e2fc"
name="event_387">
      <trigger>
        <math xmlns="http://www.w3.org/1998/Math/MathML">
          <apply>
            <geq/>
            <csymbol encoding="text"
definitionURL="http://www.sbml.org/sbml/symbols/time"> time </csymbol>
            <cn> 445.77 </cn>
          </apply>
        </math>
      </trigger>
      <listOfEventAssignments>
        <eventAssignment
variable="mw7d178aae_ff78_412a_830f_3be325d22773">
          <math xmlns="http://www.w3.org/1998/Math/MathML">
            <cn type="integer"> 0 </cn>
          </math>
        </eventAssignment>
      </listOfEventAssignments>
    </event>
    <event id="mw9a611a8f_6b1c_4604_997c_d021fb7683d1"
name="event_388">
      <trigger>
        <math xmlns="http://www.w3.org/1998/Math/MathML">
          <apply>
            <geq/>
            <csymbol encoding="text"
definitionURL="http://www.sbml.org/sbml/symbols/time"> time </csymbol>
            <cn> 447.2 </cn>
          </apply>
        </math>
      </trigger>
      <listOfEventAssignments>
        <eventAssignment
variable="mw7d178aae_ff78_412a_830f_3be325d22773">
          <math xmlns="http://www.w3.org/1998/Math/MathML">
            <cn type="integer"> 1 </cn>
          </math>
        </eventAssignment>
```

```
        &lt;/listOfEventAssignments&gt;
      &lt;/event&gt;
      <event id="mwf91eee1b_a91d_409e_9795_e99f98adb6d1"
name="event_389">
        &lt;trigger&gt;
          &lt;math xmlns="http://www.w3.org/1998/Math/MathML"&gt;
            &lt;apply&gt;
              &lt;geq/&gt;
              <csymbol encoding="text"
definitionURL="http://www.sbml.org/sbml/symbols/time"> time &lt;/csymbol&gt;
              &lt;cn&gt; 450.07 &lt;/cn&gt;
            &lt;/apply&gt;
          &lt;/math&gt;
        &lt;/trigger&gt;
        &lt;listOfEventAssignments&gt;
          <eventAssignment
variable="mw7d178aae_ff78_412a_830f_3be325d22773">
            &lt;math xmlns="http://www.w3.org/1998/Math/MathML"&gt;
              &lt;cn type="integer"&gt; 0 &lt;/cn&gt;
            &lt;/math&gt;
          &lt;/eventAssignment&gt;
        &lt;/listOfEventAssignments&gt;
      &lt;/event&gt;
      <event id="mw0c6d23aa_2d22_4d8f_b1d6_5e38616d34e1"
name="event_390">
        &lt;trigger&gt;
          &lt;math xmlns="http://www.w3.org/1998/Math/MathML"&gt;
            &lt;apply&gt;
              &lt;geq/&gt;
              <csymbol encoding="text"
definitionURL="http://www.sbml.org/sbml/symbols/time"> time &lt;/csymbol&gt;
              &lt;cn&gt; 451.5 &lt;/cn&gt;
            &lt;/apply&gt;
          &lt;/math&gt;
        &lt;/trigger&gt;
        &lt;listOfEventAssignments&gt;
          <eventAssignment
variable="mw7d178aae_ff78_412a_830f_3be325d22773">
            &lt;math xmlns="http://www.w3.org/1998/Math/MathML"&gt;
              &lt;cn type="integer"&gt; 1 &lt;/cn&gt;
            &lt;/math&gt;
          &lt;/eventAssignment&gt;
        &lt;/listOfEventAssignments&gt;
      &lt;/event&gt;
      <event id="mwf6185ac7_111e_462e_9075_cffc839e193a"
name="event_391">
        &lt;trigger&gt;
          &lt;math xmlns="http://www.w3.org/1998/Math/MathML"&gt;
            &lt;apply&gt;
              &lt;geq/&gt;
              <csymbol encoding="text"
definitionURL="http://www.sbml.org/sbml/symbols/time"> time &lt;/csymbol&gt;
```

```
            &lt;cn&gt; 454.37 &lt;/cn&gt;
          &lt;/apply&gt;
        &lt;/math&gt;
      &lt;/trigger&gt;
      &lt;listOfEventAssignments&gt;
        <eventAssignment
variable="mw7d178aae_ff78_412a_830f_3be325d22773">
          &lt;math xmlns="http://www.w3.org/1998/Math/MathML"&gt;
            &lt;cn type="integer"&gt; 0 &lt;/cn&gt;
          &lt;/math&gt;
        &lt;/eventAssignment&gt;
      &lt;/listOfEventAssignments&gt;
    &lt;/event&gt;
    <event id="mwdc3db4ff_fb2a_4062_83e9_697f61243f2f"
name="event_392">
      &lt;trigger&gt;
        &lt;math xmlns="http://www.w3.org/1998/Math/MathML"&gt;
          &lt;apply&gt;
            &lt;geq/&gt;
            <csymbol encoding="text"
definitionURL="http://www.sbml.org/sbml/symbols/time"> time &lt;/csymbol&gt;
            &lt;cn&gt; 455.8 &lt;/cn&gt;
          &lt;/apply&gt;
        &lt;/math&gt;
      &lt;/trigger&gt;
      &lt;listOfEventAssignments&gt;
        <eventAssignment
variable="mw7d178aae_ff78_412a_830f_3be325d22773">
          &lt;math xmlns="http://www.w3.org/1998/Math/MathML"&gt;
            &lt;cn type="integer"&gt; 1 &lt;/cn&gt;
          &lt;/math&gt;
        &lt;/eventAssignment&gt;
      &lt;/listOfEventAssignments&gt;
    &lt;/event&gt;
    <event id="mw1e41d49a_3328_40d7_8c0f_dfee2af27272"
name="event_393">
      &lt;trigger&gt;
        &lt;math xmlns="http://www.w3.org/1998/Math/MathML"&gt;
          &lt;apply&gt;
            &lt;geq/&gt;
            <csymbol encoding="text"
definitionURL="http://www.sbml.org/sbml/symbols/time"> time &lt;/csymbol&gt;
            &lt;cn&gt; 458.67 &lt;/cn&gt;
          &lt;/apply&gt;
        &lt;/math&gt;
      &lt;/trigger&gt;
      &lt;listOfEventAssignments&gt;
        <eventAssignment
variable="mw7d178aae_ff78_412a_830f_3be325d22773">
          &lt;math xmlns="http://www.w3.org/1998/Math/MathML"&gt;
            &lt;cn type="integer"&gt; 0 &lt;/cn&gt;
          &lt;/math&gt;
```

```
          </eventAssignment>
        </listOfEventAssignments>
      </event>
      <event id="mwa8791649_5e1b_4333_a318_3eec1b5a46d8"
name="event_394">
        <trigger>
          <math xmlns="http://www.w3.org/1998/Math/MathML">
            <apply>
              <geq/>
              <csymbol encoding="text"
definitionURL="http://www.sbml.org/sbml/symbols/time"> time </csymbol>
              <cn> 460.1 </cn>
            </apply>
          </math>
        </trigger>
        <listOfEventAssignments>
          <eventAssignment
variable="mw7d178aae_ff78_412a_830f_3be325d22773">
            <math xmlns="http://www.w3.org/1998/Math/MathML">
              <cn type="integer"> 1 </cn>
            </math>
          </eventAssignment>
        </listOfEventAssignments>
      </event>
      <event id="mw236c778a_7eca_4ec8_a2ad_6bf18caea312"
name="event_395">
        <trigger>
          <math xmlns="http://www.w3.org/1998/Math/MathML">
            <apply>
              <geq/>
              <csymbol encoding="text"
definitionURL="http://www.sbml.org/sbml/symbols/time"> time </csymbol>
              <cn> 462.97 </cn>
            </apply>
          </math>
        </trigger>
        <listOfEventAssignments>
          <eventAssignment
variable="mw7d178aae_ff78_412a_830f_3be325d22773">
            <math xmlns="http://www.w3.org/1998/Math/MathML">
              <cn type="integer"> 0 </cn>
            </math>
          </eventAssignment>
        </listOfEventAssignments>
      </event>
      <event id="mwb82d3d7e_e3d2_4507_a510_81b97f1592e3"
name="event_396">
        <trigger>
          <math xmlns="http://www.w3.org/1998/Math/MathML">
            <apply>
              <geq/>
```

```
              <csymbol encoding="text"
definitionURL="http://www.sbml.org/sbml/symbols/time"> time </csymbol>
              <cn> 464.4 </cn>
            </apply>
          </math>
        </trigger>
        <listOfEventAssignments>
          <eventAssignment
variable="mw7d178aae_ff78_412a_830f_3be325d22773">
            <math xmlns="http://www.w3.org/1998/Math/MathML">
              <cn type="integer"> 1 </cn>
            </math>
          </eventAssignment>
        </listOfEventAssignments>
      </event>
      <event id="mwf94e8c76_c137_49ca_ada7_cd1207212f0e"
name="event_397">
        <trigger>
          <math xmlns="http://www.w3.org/1998/Math/MathML">
            <apply>
              <geq/>
              <csymbol encoding="text"
definitionURL="http://www.sbml.org/sbml/symbols/time"> time </csymbol>
              <cn> 467.27 </cn>
            </apply>
          </math>
        </trigger>
        <listOfEventAssignments>
          <eventAssignment
variable="mw7d178aae_ff78_412a_830f_3be325d22773">
            <math xmlns="http://www.w3.org/1998/Math/MathML">
              <cn type="integer"> 0 </cn>
            </math>
          </eventAssignment>
        </listOfEventAssignments>
      </event>
      <event id="mw40f40a99_15e3_43f1_b60b_c206bb9ae4dc"
name="event_398">
        <trigger>
          <math xmlns="http://www.w3.org/1998/Math/MathML">
            <apply>
              <geq/>
              <csymbol encoding="text"
definitionURL="http://www.sbml.org/sbml/symbols/time"> time </csymbol>
              <cn> 468.7 </cn>
            </apply>
          </math>
        </trigger>
        <listOfEventAssignments>
          <eventAssignment
variable="mw7d178aae_ff78_412a_830f_3be325d22773">
            <math xmlns="http://www.w3.org/1998/Math/MathML">
```

```
            <cn type="integer"> 1 </cn>
          </math>
        </eventAssignment>
      </listOfEventAssignments>
    </event>
    <event id="mw3441259c_87c3_4f12_b4fe_0a649df1af16"
name="event_399">
      <trigger>
        <math xmlns="http://www.w3.org/1998/Math/MathML">
          <apply>
            <geq/>
            <csymbol encoding="text"
definitionURL="http://www.sbml.org/sbml/symbols/time"> time </csymbol>
            <cn> 471.57 </cn>
          </apply>
        </math>
      </trigger>
      <listOfEventAssignments>
        <eventAssignment
variable="mw7d178aae_ff78_412a_830f_3be325d22773">
          <math xmlns="http://www.w3.org/1998/Math/MathML">
            <cn type="integer"> 0 </cn>
          </math>
        </eventAssignment>
      </listOfEventAssignments>
    </event>
    <event id="mw164c5e44_48bb_4d0c_a036_c57e7b2ef913"
name="event_400">
      <trigger>
        <math xmlns="http://www.w3.org/1998/Math/MathML">
          <apply>
            <geq/>
            <csymbol encoding="text"
definitionURL="http://www.sbml.org/sbml/symbols/time"> time </csymbol>
            <cn type="integer"> 473 </cn>
          </apply>
        </math>
      </trigger>
      <listOfEventAssignments>
        <eventAssignment
variable="mw7d178aae_ff78_412a_830f_3be325d22773">
          <math xmlns="http://www.w3.org/1998/Math/MathML">
            <cn type="integer"> 1 </cn>
          </math>
        </eventAssignment>
      </listOfEventAssignments>
    </event>
    <event id="mwf0870fd1_d7f6_4511_a1b7_fa1e80346593"
name="event_401">
      <trigger>
        <math xmlns="http://www.w3.org/1998/Math/MathML">
          <apply>
```

```
            <geq/>
            <csymbol encoding="text"
definitionURL="http://www.sbml.org/sbml/symbols/time"> time </csymbol>
            <cn> 389.87 </cn>
          </apply>
        </math>
      </trigger>
      <listOfEventAssignments>
        <eventAssignment
variable="mwcedbe49e_2d28_4720_8fcd_207db64228cf">
          <math xmlns="http://www.w3.org/1998/Math/MathML">
            <cn type="integer"> 1 </cn>
          </math>
        </eventAssignment>
      </listOfEventAssignments>
    </event>
    <event id="mw231a51e2_1a4a_4ede_9763_825d6fc43564"
name="event_402">
      <trigger>
        <math xmlns="http://www.w3.org/1998/Math/MathML">
          <apply>
            <geq/>
            <csymbol encoding="text"
definitionURL="http://www.sbml.org/sbml/symbols/time"> time </csymbol>
            <cn> 391.3 </cn>
          </apply>
        </math>
      </trigger>
      <listOfEventAssignments>
        <eventAssignment
variable="mwcedbe49e_2d28_4720_8fcd_207db64228cf">
          <math xmlns="http://www.w3.org/1998/Math/MathML">
            <cn type="integer"> 0 </cn>
          </math>
        </eventAssignment>
      </listOfEventAssignments>
    </event>
    <event id="mw53827592_2340_4123_a1e7_4663d2ee02d2"
name="event_403">
      <trigger>
        <math xmlns="http://www.w3.org/1998/Math/MathML">
          <apply>
            <geq/>
            <csymbol encoding="text"
definitionURL="http://www.sbml.org/sbml/symbols/time"> time </csymbol>
            <cn> 394.17 </cn>
          </apply>
        </math>
      </trigger>
      <listOfEventAssignments>
        <eventAssignment
variable="mwcedbe49e_2d28_4720_8fcd_207db64228cf">
```

```
            <math xmlns="http://www.w3.org/1998/Math/MathML">
              <cn type="integer"> 1 </cn>
            </math>
          </eventAssignment>
        </listOfEventAssignments>
      </event>
      <event id="mw90b71965_a81d_40cb_8913_0e2496d3cd27"
name="event_404">
        <trigger>
          <math xmlns="http://www.w3.org/1998/Math/MathML">
            <apply>
              <geq/>
              <csymbol encoding="text"
definitionURL="http://www.sbml.org/sbml/symbols/time"> time </csymbol>
              <cn> 395.6 </cn>
            </apply>
          </math>
        </trigger>
        <listOfEventAssignments>
          <eventAssignment
variable="mwcedbe49e_2d28_4720_8fcd_207db64228cf">
            <math xmlns="http://www.w3.org/1998/Math/MathML">
              <cn type="integer"> 0 </cn>
            </math>
          </eventAssignment>
        </listOfEventAssignments>
      </event>
      <event id="mwa041cc44_ea6d_47a4_b79a_19a048f0a0ce"
name="event_405">
        <trigger>
          <math xmlns="http://www.w3.org/1998/Math/MathML">
            <apply>
              <geq/>
              <csymbol encoding="text"
definitionURL="http://www.sbml.org/sbml/symbols/time"> time </csymbol>
              <cn> 398.47 </cn>
            </apply>
          </math>
        </trigger>
        <listOfEventAssignments>
          <eventAssignment
variable="mwcedbe49e_2d28_4720_8fcd_207db64228cf">
            <math xmlns="http://www.w3.org/1998/Math/MathML">
              <cn type="integer"> 1 </cn>
            </math>
          </eventAssignment>
        </listOfEventAssignments>
      </event>
      <event id="mw08f1a7d1_89ee_429d_9e58_d84a634e81cb"
name="event_406">
        <trigger>
          <math xmlns="http://www.w3.org/1998/Math/MathML">
```

```
            <apply>
              <geq/>
              <csymbol encoding="text"
definitionURL="http://www.sbml.org/sbml/symbols/time"> time </csymbol>
              <cn> 399.9 </cn>
            </apply>
          </math>
        </trigger>
        <listOfEventAssignments>
          <eventAssignment
variable="mwcedbe49e_2d28_4720_8fcd_207db64228cf">
            <math xmlns="http://www.w3.org/1998/Math/MathML">
              <cn type="integer"> 0 </cn>
            </math>
          </eventAssignment>
        </listOfEventAssignments>
      </event>
      <event id="mw41c10699_0d90_4fff_8168_8786b1d60db3"
name="event_407">
        <trigger>
          <math xmlns="http://www.w3.org/1998/Math/MathML">
            <apply>
              <geq/>
              <csymbol encoding="text"
definitionURL="http://www.sbml.org/sbml/symbols/time"> time </csymbol>
              <cn> 402.77 </cn>
            </apply>
          </math>
        </trigger>
        <listOfEventAssignments>
          <eventAssignment
variable="mwcedbe49e_2d28_4720_8fcd_207db64228cf">
            <math xmlns="http://www.w3.org/1998/Math/MathML">
              <cn type="integer"> 1 </cn>
            </math>
          </eventAssignment>
        </listOfEventAssignments>
      </event>
      <event id="mw85005529_8cc8_4eb0_86b4_20095e25ea2e"
name="event_408">
        <trigger>
          <math xmlns="http://www.w3.org/1998/Math/MathML">
            <apply>
              <geq/>
              <csymbol encoding="text"
definitionURL="http://www.sbml.org/sbml/symbols/time"> time </csymbol>
              <cn> 404.2 </cn>
            </apply>
          </math>
        </trigger>
        <listOfEventAssignments>
```

```
          <eventAssignment
variable="mwcedbe49e_2d28_4720_8fcd_207db64228cf">
            <math xmlns="http://www.w3.org/1998/Math/MathML">
              <cn type="integer"> 0 </cn>
            </math>
          </eventAssignment>
        </listOfEventAssignments>
      </event>
      <event id="mw9eb2367c_5b49_475b_8a4b_d28cbc23668d"
name="event_409">
        <trigger>
          <math xmlns="http://www.w3.org/1998/Math/MathML">
            <apply>
              <geq/>
              <csymbol encoding="text"
definitionURL="http://www.sbml.org/sbml/symbols/time"> time </csymbol>
              <cn> 407.07 </cn>
            </apply>
          </math>
        </trigger>
        <listOfEventAssignments>
          <eventAssignment
variable="mwcedbe49e_2d28_4720_8fcd_207db64228cf">
            <math xmlns="http://www.w3.org/1998/Math/MathML">
              <cn type="integer"> 1 </cn>
            </math>
          </eventAssignment>
        </listOfEventAssignments>
      </event>
      <event id="mw2438f7ab_f819_49f9_b787_a1d1bcc3af00"
name="event_410">
        <trigger>
          <math xmlns="http://www.w3.org/1998/Math/MathML">
            <apply>
              <geq/>
              <csymbol encoding="text"
definitionURL="http://www.sbml.org/sbml/symbols/time"> time </csymbol>
              <cn> 408.5 </cn>
            </apply>
          </math>
        </trigger>
        <listOfEventAssignments>
          <eventAssignment
variable="mwcedbe49e_2d28_4720_8fcd_207db64228cf">
            <math xmlns="http://www.w3.org/1998/Math/MathML">
              <cn type="integer"> 0 </cn>
            </math>
          </eventAssignment>
        </listOfEventAssignments>
      </event>
      <event id="mw2b389df0_b9f4_4915_88d9_fd8ad491d1de"
name="event_411">
```

```
        &lt;trigger&gt;
          &lt;math xmlns="http://www.w3.org/1998/Math/MathML"&gt;
            &lt;apply&gt;
              &lt;geq/&gt;
              <csymbol encoding="text"
definitionURL="http://www.sbml.org/sbml/symbols/time"> time &lt;/csymbol&gt;
              &lt;cn&gt; 411.37 &lt;/cn&gt;
            &lt;/apply&gt;
          &lt;/math&gt;
        &lt;/trigger&gt;
        &lt;listOfEventAssignments&gt;
          <eventAssignment
variable="mwcedbe49e_2d28_4720_8fcd_207db64228cf">
            &lt;math xmlns="http://www.w3.org/1998/Math/MathML"&gt;
              &lt;cn type="integer"&gt; 1 &lt;/cn&gt;
            &lt;/math&gt;
          &lt;/eventAssignment&gt;
        &lt;/listOfEventAssignments&gt;
      &lt;/event&gt;
      <event id="mwfcf78153_15d6_4c21_8090_07aee554c745"
name="event_412">
        &lt;trigger&gt;
          &lt;math xmlns="http://www.w3.org/1998/Math/MathML"&gt;
            &lt;apply&gt;
              &lt;geq/&gt;
              <csymbol encoding="text"
definitionURL="http://www.sbml.org/sbml/symbols/time"> time &lt;/csymbol&gt;
              &lt;cn&gt; 412.8 &lt;/cn&gt;
            &lt;/apply&gt;
          &lt;/math&gt;
        &lt;/trigger&gt;
        &lt;listOfEventAssignments&gt;
          <eventAssignment
variable="mwcedbe49e_2d28_4720_8fcd_207db64228cf">
            &lt;math xmlns="http://www.w3.org/1998/Math/MathML"&gt;
              &lt;cn type="integer"&gt; 0 &lt;/cn&gt;
            &lt;/math&gt;
          &lt;/eventAssignment&gt;
        &lt;/listOfEventAssignments&gt;
      &lt;/event&gt;
      <event id="mw85caf869_e36a_4158_9dba_92aa35961e6b"
name="event_413">
        &lt;trigger&gt;
          &lt;math xmlns="http://www.w3.org/1998/Math/MathML"&gt;
            &lt;apply&gt;
              &lt;geq/&gt;
              <csymbol encoding="text"
definitionURL="http://www.sbml.org/sbml/symbols/time"> time &lt;/csymbol&gt;
              &lt;cn&gt; 415.67 &lt;/cn&gt;
            &lt;/apply&gt;
          &lt;/math&gt;
        &lt;/trigger&gt;
```

```
        <listOfEventAssignments>
          <eventAssignment
variable="mwcedbe49e_2d28_4720_8fcd_207db64228cf">
            <math xmlns="http://www.w3.org/1998/Math/MathML">
              <cn type="integer"> 1 </cn>
            </math>
          </eventAssignment>
        </listOfEventAssignments>
      </event>
      <event id="mw85e490b7_193c_454c_af10_af86204e284a"
name="event_414">
        <trigger>
          <math xmlns="http://www.w3.org/1998/Math/MathML">
            <apply>
              <geq/>
              <csymbol encoding="text"
definitionURL="http://www.sbml.org/sbml/symbols/time"> time </csymbol>
              <cn> 417.1 </cn>
            </apply>
          </math>
        </trigger>
        <listOfEventAssignments>
          <eventAssignment
variable="mwcedbe49e_2d28_4720_8fcd_207db64228cf">
            <math xmlns="http://www.w3.org/1998/Math/MathML">
              <cn type="integer"> 0 </cn>
            </math>
          </eventAssignment>
        </listOfEventAssignments>
      </event>
      <event id="mw14a0e5c7_691c_4f81_9b39_18169ca491ff"
name="event_415">
        <trigger>
          <math xmlns="http://www.w3.org/1998/Math/MathML">
            <apply>
              <geq/>
              <csymbol encoding="text"
definitionURL="http://www.sbml.org/sbml/symbols/time"> time </csymbol>
              <cn> 419.97 </cn>
            </apply>
          </math>
        </trigger>
        <listOfEventAssignments>
          <eventAssignment
variable="mwcedbe49e_2d28_4720_8fcd_207db64228cf">
            <math xmlns="http://www.w3.org/1998/Math/MathML">
              <cn type="integer"> 1 </cn>
            </math>
          </eventAssignment>
        </listOfEventAssignments>
      </event>
```

```
      <event id="mw87fbb6f4_77c2_4063_a5cd_72cb2524c9c2"
name="event_416">
        &lt;trigger&gt;
          &lt;math xmlns="http://www.w3.org/1998/Math/MathML"&gt;
            &lt;apply&gt;
              &lt;geq/&gt;
              <csymbol encoding="text"
definitionURL="http://www.sbml.org/sbml/symbols/time"> time &lt;/csymbol&gt;
              &lt;cn&gt; 421.4 &lt;/cn&gt;
            &lt;/apply&gt;
          &lt;/math&gt;
        &lt;/trigger&gt;
        &lt;listOfEventAssignments&gt;
          <eventAssignment
variable="mwcedbe49e_2d28_4720_8fcd_207db64228cf">
            &lt;math xmlns="http://www.w3.org/1998/Math/MathML"&gt;
              &lt;cn type="integer"&gt; 0 &lt;/cn&gt;
            &lt;/math&gt;
          &lt;/eventAssignment&gt;
        &lt;/listOfEventAssignments&gt;
      &lt;/event&gt;
      <event id="mw614de16b_caef_444d_aae8_64f99681f312"
name="event_417">
        &lt;trigger&gt;
          &lt;math xmlns="http://www.w3.org/1998/Math/MathML"&gt;
            &lt;apply&gt;
              &lt;geq/&gt;
              <csymbol encoding="text"
definitionURL="http://www.sbml.org/sbml/symbols/time"> time &lt;/csymbol&gt;
              &lt;cn&gt; 424.27 &lt;/cn&gt;
            &lt;/apply&gt;
          &lt;/math&gt;
        &lt;/trigger&gt;
        &lt;listOfEventAssignments&gt;
          <eventAssignment
variable="mwcedbe49e_2d28_4720_8fcd_207db64228cf">
            &lt;math xmlns="http://www.w3.org/1998/Math/MathML"&gt;
              &lt;cn type="integer"&gt; 1 &lt;/cn&gt;
            &lt;/math&gt;
          &lt;/eventAssignment&gt;
        &lt;/listOfEventAssignments&gt;
      &lt;/event&gt;
      <event id="mw8af5412f_014c_4b94_a126_acd1943a70c8"
name="event_418">
        &lt;trigger&gt;
          &lt;math xmlns="http://www.w3.org/1998/Math/MathML"&gt;
            &lt;apply&gt;
              &lt;geq/&gt;
              <csymbol encoding="text"
definitionURL="http://www.sbml.org/sbml/symbols/time"> time &lt;/csymbol&gt;
              &lt;cn&gt; 425.7 &lt;/cn&gt;
            &lt;/apply&gt;
```

```
          </math>
        </trigger>
        <listOfEventAssignments>
          <eventAssignment
variable="mwcedbe49e_2d28_4720_8fcd_207db64228cf">
            <math xmlns="http://www.w3.org/1998/Math/MathML">
              <cn type="integer"> 0 </cn>
            </math>
          </eventAssignment>
        </listOfEventAssignments>
      </event>
      <event id="mw340de4a6_3869_4d30_89c9_535caa234725"
name="event_419">
        <trigger>
          <math xmlns="http://www.w3.org/1998/Math/MathML">
            <apply>
              <geq/>
              <csymbol encoding="text"
definitionURL="http://www.sbml.org/sbml/symbols/time"> time </csymbol>
              <cn> 428.57 </cn>
            </apply>
          </math>
        </trigger>
        <listOfEventAssignments>
          <eventAssignment
variable="mwcedbe49e_2d28_4720_8fcd_207db64228cf">
            <math xmlns="http://www.w3.org/1998/Math/MathML">
              <cn type="integer"> 1 </cn>
            </math>
          </eventAssignment>
        </listOfEventAssignments>
      </event>
      <event id="mwcdb9080d_c9a0_4340_aed5_baab45767c6c"
name="event_420">
        <trigger>
          <math xmlns="http://www.w3.org/1998/Math/MathML">
            <apply>
              <geq/>
              <csymbol encoding="text"
definitionURL="http://www.sbml.org/sbml/symbols/time"> time </csymbol>
              <cn type="integer"> 430 </cn>
            </apply>
          </math>
        </trigger>
        <listOfEventAssignments>
          <eventAssignment
variable="mwcedbe49e_2d28_4720_8fcd_207db64228cf">
            <math xmlns="http://www.w3.org/1998/Math/MathML">
              <cn type="integer"> 0 </cn>
            </math>
          </eventAssignment>
        </listOfEventAssignments>
```

```
      &lt;/event&gt;
      <event id="mw6fd06cf1_5665_46dd_91e5_246f7c1d312a"
name="event_421">
        &lt;trigger&gt;
          &lt;math xmlns="http://www.w3.org/1998/Math/MathML"&gt;
            &lt;apply&gt;
              &lt;geq/&gt;
              <csymbol encoding="text"
definitionURL="http://www.sbml.org/sbml/symbols/time"> time &lt;/csymbol&gt;
              &lt;cn&gt; 432.87 &lt;/cn&gt;
            &lt;/apply&gt;
          &lt;/math&gt;
        &lt;/trigger&gt;
        &lt;listOfEventAssignments&gt;
          <eventAssignment
variable="mwcedbe49e_2d28_4720_8fcd_207db64228cf">
            &lt;math xmlns="http://www.w3.org/1998/Math/MathML"&gt;
              &lt;cn type="integer"&gt; 1 &lt;/cn&gt;
            &lt;/math&gt;
          &lt;/eventAssignment&gt;
        &lt;/listOfEventAssignments&gt;
      &lt;/event&gt;
      <event id="mw6b9731d8_654c_4e04_9a27_d7751824c1cd"
name="event_422">
        &lt;trigger&gt;
          &lt;math xmlns="http://www.w3.org/1998/Math/MathML"&gt;
            &lt;apply&gt;
              &lt;geq/&gt;
              <csymbol encoding="text"
definitionURL="http://www.sbml.org/sbml/symbols/time"> time &lt;/csymbol&gt;
              &lt;cn&gt; 434.3 &lt;/cn&gt;
            &lt;/apply&gt;
          &lt;/math&gt;
        &lt;/trigger&gt;
        &lt;listOfEventAssignments&gt;
          <eventAssignment
variable="mwcedbe49e_2d28_4720_8fcd_207db64228cf">
            &lt;math xmlns="http://www.w3.org/1998/Math/MathML"&gt;
              &lt;cn type="integer"&gt; 0 &lt;/cn&gt;
            &lt;/math&gt;
          &lt;/eventAssignment&gt;
        &lt;/listOfEventAssignments&gt;
      &lt;/event&gt;
      <event id="mw5a7222ca_04e6_4298_a74b_b2ac15ac0995"
name="event_423">
        &lt;trigger&gt;
          &lt;math xmlns="http://www.w3.org/1998/Math/MathML"&gt;
            &lt;apply&gt;
              &lt;geq/&gt;
              <csymbol encoding="text"
definitionURL="http://www.sbml.org/sbml/symbols/time"> time &lt;/csymbol&gt;
              &lt;cn&gt; 437.17 &lt;/cn&gt;
```

```
            </apply>
          </math>
        </trigger>
        <listOfEventAssignments>
          <eventAssignment
variable="mwcedbe49e_2d28_4720_8fcd_207db64228cf">
            <math xmlns="http://www.w3.org/1998/Math/MathML">
              <cn type="integer"> 1 </cn>
            </math>
          </eventAssignment>
        </listOfEventAssignments>
      </event>
      <event id="mwa17aabb4_17f3_437f_a4fd_28a4161fd844"
name="event_424">
        <trigger>
          <math xmlns="http://www.w3.org/1998/Math/MathML">
            <apply>
              <geq/>
              <csymbol encoding="text"
definitionURL="http://www.sbml.org/sbml/symbols/time"> time </csymbol>
              <cn> 438.6 </cn>
            </apply>
          </math>
        </trigger>
        <listOfEventAssignments>
          <eventAssignment
variable="mwcedbe49e_2d28_4720_8fcd_207db64228cf">
            <math xmlns="http://www.w3.org/1998/Math/MathML">
              <cn type="integer"> 0 </cn>
            </math>
          </eventAssignment>
        </listOfEventAssignments>
      </event>
      <event id="mw81cf6661_abc1_496e_86c1_c8b97ac2acc3"
name="event_425">
        <trigger>
          <math xmlns="http://www.w3.org/1998/Math/MathML">
            <apply>
              <geq/>
              <csymbol encoding="text"
definitionURL="http://www.sbml.org/sbml/symbols/time"> time </csymbol>
              <cn> 441.47 </cn>
            </apply>
          </math>
        </trigger>
        <listOfEventAssignments>
          <eventAssignment
variable="mwcedbe49e_2d28_4720_8fcd_207db64228cf">
            <math xmlns="http://www.w3.org/1998/Math/MathML">
              <cn type="integer"> 1 </cn>
            </math>
          </eventAssignment>
```

```
        </listOfEventAssignments>
      </event>
      <event id="mw022515f7_4863_4291_8a4d_3d713fc0aa5a"
name="event_426">
        <trigger>
          <math xmlns="http://www.w3.org/1998/Math/MathML">
            <apply>
              <geq/>
              <csymbol encoding="text"
definitionURL="http://www.sbml.org/sbml/symbols/time"> time </csymbol>
              <cn> 442.9 </cn>
            </apply>
          </math>
        </trigger>
        <listOfEventAssignments>
          <eventAssignment
variable="mwcedbe49e_2d28_4720_8fcd_207db64228cf">
            <math xmlns="http://www.w3.org/1998/Math/MathML">
              <cn type="integer"> 0 </cn>
            </math>
          </eventAssignment>
        </listOfEventAssignments>
      </event>
      <event id="mwd6a89f28_5829_4896_a2de_58ad18f1f381"
name="event_427">
        <trigger>
          <math xmlns="http://www.w3.org/1998/Math/MathML">
            <apply>
              <geq/>
              <csymbol encoding="text"
definitionURL="http://www.sbml.org/sbml/symbols/time"> time </csymbol>
              <cn> 445.77 </cn>
            </apply>
          </math>
        </trigger>
        <listOfEventAssignments>
          <eventAssignment
variable="mwcedbe49e_2d28_4720_8fcd_207db64228cf">
            <math xmlns="http://www.w3.org/1998/Math/MathML">
              <cn type="integer"> 1 </cn>
            </math>
          </eventAssignment>
        </listOfEventAssignments>
      </event>
      <event id="mw8a38064f_4dc9_4c19_9e3f_d2db3cb520aa"
name="event_428">
        <trigger>
          <math xmlns="http://www.w3.org/1998/Math/MathML">
            <apply>
              <geq/>
              <csymbol encoding="text"
definitionURL="http://www.sbml.org/sbml/symbols/time"> time </csymbol>
```

```
              <cn> 447.2 </cn>
            </apply>
          </math>
        </trigger>
        <listOfEventAssignments>
          <eventAssignment
variable="mwcedbe49e_2d28_4720_8fcd_207db64228cf">
            <math xmlns="http://www.w3.org/1998/Math/MathML">
              <cn type="integer"> 0 </cn>
            </math>
          </eventAssignment>
        </listOfEventAssignments>
      </event>
      <event id="mw9f505ec3_5757_4f60_90a8_9b390cbd8767"
name="event_429">
        <trigger>
          <math xmlns="http://www.w3.org/1998/Math/MathML">
            <apply>
              <geq/>
              <csymbol encoding="text"
definitionURL="http://www.sbml.org/sbml/symbols/time"> time </csymbol>
              <cn> 450.07 </cn>
            </apply>
          </math>
        </trigger>
        <listOfEventAssignments>
          <eventAssignment
variable="mwcedbe49e_2d28_4720_8fcd_207db64228cf">
            <math xmlns="http://www.w3.org/1998/Math/MathML">
              <cn type="integer"> 1 </cn>
            </math>
          </eventAssignment>
        </listOfEventAssignments>
      </event>
      <event id="mwff372d39_d6ef_44c5_a190_41dbe1baa6a7"
name="event_430">
        <trigger>
          <math xmlns="http://www.w3.org/1998/Math/MathML">
            <apply>
              <geq/>
              <csymbol encoding="text"
definitionURL="http://www.sbml.org/sbml/symbols/time"> time </csymbol>
              <cn> 451.5 </cn>
            </apply>
          </math>
        </trigger>
        <listOfEventAssignments>
          <eventAssignment
variable="mwcedbe49e_2d28_4720_8fcd_207db64228cf">
            <math xmlns="http://www.w3.org/1998/Math/MathML">
              <cn type="integer"> 0 </cn>
            </math>
```

```
          </eventAssignment>
        </listOfEventAssignments>
      </event>
      <event id="mwa315ae10_c13b_462c_917c_50594b2a8440"
name="event_431">
        <trigger>
          <math xmlns="http://www.w3.org/1998/Math/MathML">
            <apply>
              <geq/>
              <csymbol encoding="text"
definitionURL="http://www.sbml.org/sbml/symbols/time"> time </csymbol>
              <cn> 454.37 </cn>
            </apply>
          </math>
        </trigger>
        <listOfEventAssignments>
          <eventAssignment
variable="mwcedbe49e_2d28_4720_8fcd_207db64228cf">
            <math xmlns="http://www.w3.org/1998/Math/MathML">
              <cn type="integer"> 1 </cn>
            </math>
          </eventAssignment>
        </listOfEventAssignments>
      </event>
      <event id="mw866784e5_7933_414c_ae78_4cf4d4381473"
name="event_432">
        <trigger>
          <math xmlns="http://www.w3.org/1998/Math/MathML">
            <apply>
              <geq/>
              <csymbol encoding="text"
definitionURL="http://www.sbml.org/sbml/symbols/time"> time </csymbol>
              <cn> 455.8 </cn>
            </apply>
          </math>
        </trigger>
        <listOfEventAssignments>
          <eventAssignment
variable="mwcedbe49e_2d28_4720_8fcd_207db64228cf">
            <math xmlns="http://www.w3.org/1998/Math/MathML">
              <cn type="integer"> 0 </cn>
            </math>
          </eventAssignment>
        </listOfEventAssignments>
      </event>
      <event id="mw1dc0fccc_9c9b_4b03_8801_feee5c77e3e7"
name="event_433">
        <trigger>
          <math xmlns="http://www.w3.org/1998/Math/MathML">
            <apply>
              <geq/>
```

```
              <csymbol encoding="text"
definitionURL="http://www.sbml.org/sbml/symbols/time"> time &lt;/csymbol&gt;
              &lt;cn&gt; 458.67 &lt;/cn&gt;
            &lt;/apply&gt;
          &lt;/math&gt;
        &lt;/trigger&gt;
        &lt;listOfEventAssignments&gt;
          <eventAssignment
variable="mwcedbe49e_2d28_4720_8fcd_207db64228cf">
            &lt;math xmlns="http://www.w3.org/1998/Math/MathML"&gt;
              &lt;cn type="integer"&gt; 1 &lt;/cn&gt;
            &lt;/math&gt;
          &lt;/eventAssignment&gt;
        &lt;/listOfEventAssignments&gt;
      &lt;/event&gt;
      <event id="mw6d031e97_d724_4ae8_9808_fe97d4740a44"
name="event_434">
        &lt;trigger&gt;
          &lt;math xmlns="http://www.w3.org/1998/Math/MathML"&gt;
            &lt;apply&gt;
              &lt;geq/&gt;
              <csymbol encoding="text"
definitionURL="http://www.sbml.org/sbml/symbols/time"> time &lt;/csymbol&gt;
              &lt;cn&gt; 460.1 &lt;/cn&gt;
            &lt;/apply&gt;
          &lt;/math&gt;
        &lt;/trigger&gt;
        &lt;listOfEventAssignments&gt;
          <eventAssignment
variable="mwcedbe49e_2d28_4720_8fcd_207db64228cf">
            &lt;math xmlns="http://www.w3.org/1998/Math/MathML"&gt;
              &lt;cn type="integer"&gt; 0 &lt;/cn&gt;
            &lt;/math&gt;
          &lt;/eventAssignment&gt;
        &lt;/listOfEventAssignments&gt;
      &lt;/event&gt;
      <event id="mwafd6d9b6_9d6d_46c8_9ba1_1af1dfb22b81"
name="event_435">
        &lt;trigger&gt;
          &lt;math xmlns="http://www.w3.org/1998/Math/MathML"&gt;
            &lt;apply&gt;
              &lt;geq/&gt;
              <csymbol encoding="text"
definitionURL="http://www.sbml.org/sbml/symbols/time"> time &lt;/csymbol&gt;
              &lt;cn&gt; 462.97 &lt;/cn&gt;
            &lt;/apply&gt;
          &lt;/math&gt;
        &lt;/trigger&gt;
        &lt;listOfEventAssignments&gt;
          <eventAssignment
variable="mwcedbe49e_2d28_4720_8fcd_207db64228cf">
            &lt;math xmlns="http://www.w3.org/1998/Math/MathML"&gt;
```

```
            &lt;cn type="integer"&gt; 1 &lt;/cn&gt;
          &lt;/math&gt;
        &lt;/eventAssignment&gt;
      &lt;/listOfEventAssignments&gt;
    &lt;/event&gt;
    <event id="mw12c03681_ec33_4a67_a47e_c9aaca313e09"
name="event_436">
      &lt;trigger&gt;
        &lt;math xmlns="http://www.w3.org/1998/Math/MathML"&gt;
          &lt;apply&gt;
            &lt;geq/&gt;
            <csymbol encoding="text"
definitionURL="http://www.sbml.org/sbml/symbols/time"> time &lt;/csymbol&gt;
            &lt;cn&gt; 464.4 &lt;/cn&gt;
          &lt;/apply&gt;
        &lt;/math&gt;
      &lt;/trigger&gt;
      &lt;listOfEventAssignments&gt;
        <eventAssignment
variable="mwcedbe49e_2d28_4720_8fcd_207db64228cf">
          &lt;math xmlns="http://www.w3.org/1998/Math/MathML"&gt;
            &lt;cn type="integer"&gt; 0 &lt;/cn&gt;
          &lt;/math&gt;
        &lt;/eventAssignment&gt;
      &lt;/listOfEventAssignments&gt;
    &lt;/event&gt;
    <event id="mwcac36643_f559_4742_a8c9_5410757a3531"
name="event_437">
      &lt;trigger&gt;
        &lt;math xmlns="http://www.w3.org/1998/Math/MathML"&gt;
          &lt;apply&gt;
            &lt;geq/&gt;
            <csymbol encoding="text"
definitionURL="http://www.sbml.org/sbml/symbols/time"> time &lt;/csymbol&gt;
            &lt;cn&gt; 467.27 &lt;/cn&gt;
          &lt;/apply&gt;
        &lt;/math&gt;
      &lt;/trigger&gt;
      &lt;listOfEventAssignments&gt;
        <eventAssignment
variable="mwcedbe49e_2d28_4720_8fcd_207db64228cf">
          &lt;math xmlns="http://www.w3.org/1998/Math/MathML"&gt;
            &lt;cn type="integer"&gt; 1 &lt;/cn&gt;
          &lt;/math&gt;
        &lt;/eventAssignment&gt;
      &lt;/listOfEventAssignments&gt;
    &lt;/event&gt;
    <event id="mw4e6cf717_98a8_41d3_ae69_09b1dfe607b4"
name="event_438">
      &lt;trigger&gt;
        &lt;math xmlns="http://www.w3.org/1998/Math/MathML"&gt;
          &lt;apply&gt;
```

```
              <geq/>
              <csymbol encoding="text"
definitionURL="http://www.sbml.org/sbml/symbols/time"> time </csymbol>
              <cn> 468.7 </cn>
            </apply>
          </math>
        </trigger>
        <listOfEventAssignments>
          <eventAssignment
variable="mwcedbe49e_2d28_4720_8fcd_207db64228cf">
            <math xmlns="http://www.w3.org/1998/Math/MathML">
              <cn type="integer"> 0 </cn>
            </math>
          </eventAssignment>
        </listOfEventAssignments>
      </event>
      <event id="mw6b138527_4d90_4e98_8023_590189a302eb"
name="event_439">
        <trigger>
          <math xmlns="http://www.w3.org/1998/Math/MathML">
            <apply>
              <geq/>
              <csymbol encoding="text"
definitionURL="http://www.sbml.org/sbml/symbols/time"> time </csymbol>
              <cn> 471.57 </cn>
            </apply>
          </math>
        </trigger>
        <listOfEventAssignments>
          <eventAssignment
variable="mwcedbe49e_2d28_4720_8fcd_207db64228cf">
            <math xmlns="http://www.w3.org/1998/Math/MathML">
              <cn type="integer"> 1 </cn>
            </math>
          </eventAssignment>
        </listOfEventAssignments>
      </event>
      <event id="mw7cc5ffc2_a427_4a40_8ebc_d9afeb6b8865"
name="event_440">
        <trigger>
          <math xmlns="http://www.w3.org/1998/Math/MathML">
            <apply>
              <geq/>
              <csymbol encoding="text"
definitionURL="http://www.sbml.org/sbml/symbols/time"> time </csymbol>
              <cn type="integer"> 473 </cn>
            </apply>
          </math>
        </trigger>
        <listOfEventAssignments>
          <eventAssignment
variable="mwcedbe49e_2d28_4720_8fcd_207db64228cf">
```

```
            &lt;math xmlns="http://www.w3.org/1998/Math/MathML"&gt;
              &lt;cn type="integer"&gt; 0 &lt;/cn&gt;
            &lt;/math&gt;
          &lt;/eventAssignment&gt;
        &lt;/listOfEventAssignments&gt;
      &lt;/event&gt;
      <event id="mw0b91af8a_b5eb_422e_a0f8_5f58c3e8be0a"
name="event_441">
        &lt;trigger&gt;
          &lt;math xmlns="http://www.w3.org/1998/Math/MathML"&gt;
            &lt;apply&gt;
              &lt;geq/&gt;
              <csymbol encoding="text"
definitionURL="http://www.sbml.org/sbml/symbols/time"> time &lt;/csymbol&gt;
              &lt;cn&gt; 475.87 &lt;/cn&gt;
            &lt;/apply&gt;
          &lt;/math&gt;
        &lt;/trigger&gt;
        &lt;listOfEventAssignments&gt;
          <eventAssignment
variable="mwcedbe49e_2d28_4720_8fcd_207db64228cf">
            &lt;math xmlns="http://www.w3.org/1998/Math/MathML"&gt;
              &lt;cn type="integer"&gt; 1 &lt;/cn&gt;
            &lt;/math&gt;
          &lt;/eventAssignment&gt;
        &lt;/listOfEventAssignments&gt;
      &lt;/event&gt;
      <event id="mw5f3ae539_4000_4e84_a0ca_26138619c003"
name="event_442">
        &lt;trigger&gt;
          &lt;math xmlns="http://www.w3.org/1998/Math/MathML"&gt;
            &lt;apply&gt;
              &lt;geq/&gt;
              <csymbol encoding="text"
definitionURL="http://www.sbml.org/sbml/symbols/time"> time &lt;/csymbol&gt;
              &lt;cn&gt; 477.3 &lt;/cn&gt;
            &lt;/apply&gt;
          &lt;/math&gt;
        &lt;/trigger&gt;
        &lt;listOfEventAssignments&gt;
          <eventAssignment
variable="mwcedbe49e_2d28_4720_8fcd_207db64228cf">
            &lt;math xmlns="http://www.w3.org/1998/Math/MathML"&gt;
              &lt;cn type="integer"&gt; 0 &lt;/cn&gt;
            &lt;/math&gt;
          &lt;/eventAssignment&gt;
        &lt;/listOfEventAssignments&gt;
      &lt;/event&gt;
      <event id="mw061eff91_ade1_4a00_b473_bd080a0dc06c"
name="event_443">
        &lt;trigger&gt;
          &lt;math xmlns="http://www.w3.org/1998/Math/MathML"&gt;
```

```
            &lt;apply&gt;
              &lt;geq/&gt;
              <csymbol encoding="text"
definitionURL="http://www.sbml.org/sbml/symbols/time"> time &lt;/csymbol&gt;
              &lt;cn&gt; 480.17 &lt;/cn&gt;
            &lt;/apply&gt;
          &lt;/math&gt;
        &lt;/trigger&gt;
        &lt;listOfEventAssignments&gt;
          <eventAssignment
variable="mwcedbe49e_2d28_4720_8fcd_207db64228cf">
            &lt;math xmlns="http://www.w3.org/1998/Math/MathML"&gt;
              &lt;cn type="integer"&gt; 1 &lt;/cn&gt;
            &lt;/math&gt;
          &lt;/eventAssignment&gt;
        &lt;/listOfEventAssignments&gt;
      &lt;/event&gt;
      <event id="mw83c9144e_b65a_4a42_b1b9_a50e0a373266"
name="event_444">
        &lt;trigger&gt;
          &lt;math xmlns="http://www.w3.org/1998/Math/MathML"&gt;
            &lt;apply&gt;
              &lt;geq/&gt;
              <csymbol encoding="text"
definitionURL="http://www.sbml.org/sbml/symbols/time"> time &lt;/csymbol&gt;
              &lt;cn&gt; 481.6 &lt;/cn&gt;
            &lt;/apply&gt;
          &lt;/math&gt;
        &lt;/trigger&gt;
        &lt;listOfEventAssignments&gt;
          <eventAssignment
variable="mwcedbe49e_2d28_4720_8fcd_207db64228cf">
            &lt;math xmlns="http://www.w3.org/1998/Math/MathML"&gt;
              &lt;cn type="integer"&gt; 0 &lt;/cn&gt;
            &lt;/math&gt;
          &lt;/eventAssignment&gt;
        &lt;/listOfEventAssignments&gt;
      &lt;/event&gt;
      <event id="mwb32bd45b_a5f5_4340_891e_cc05d06a2462"
name="event_445">
        &lt;trigger&gt;
          &lt;math xmlns="http://www.w3.org/1998/Math/MathML"&gt;
            &lt;apply&gt;
              &lt;geq/&gt;
              <csymbol encoding="text"
definitionURL="http://www.sbml.org/sbml/symbols/time"> time &lt;/csymbol&gt;
              &lt;cn&gt; 484.47 &lt;/cn&gt;
            &lt;/apply&gt;
          &lt;/math&gt;
        &lt;/trigger&gt;
        &lt;listOfEventAssignments&gt;
```

```
          <eventAssignment
variable="mwcedbe49e_2d28_4720_8fcd_207db64228cf">
            <math xmlns="http://www.w3.org/1998/Math/MathML">
              <cn type="integer"> 1 </cn>
            </math>
          </eventAssignment>
        </listOfEventAssignments>
      </event>
      <event id="mwac668489_1e3a_4cc0_9360_e89de4a2c5a0"
name="event_446">
        <trigger>
          <math xmlns="http://www.w3.org/1998/Math/MathML">
            <apply>
              <geq/>
              <csymbol encoding="text"
definitionURL="http://www.sbml.org/sbml/symbols/time"> time </csymbol>
              <cn> 485.9 </cn>
            </apply>
          </math>
        </trigger>
        <listOfEventAssignments>
          <eventAssignment
variable="mwcedbe49e_2d28_4720_8fcd_207db64228cf">
            <math xmlns="http://www.w3.org/1998/Math/MathML">
              <cn type="integer"> 0 </cn>
            </math>
          </eventAssignment>
        </listOfEventAssignments>
      </event>
      <event id="mwef4adb42_11f2_46f0_8820_96607b46f3df"
name="event_447">
        <trigger>
          <math xmlns="http://www.w3.org/1998/Math/MathML">
            <apply>
              <geq/>
              <csymbol encoding="text"
definitionURL="http://www.sbml.org/sbml/symbols/time"> time </csymbol>
              <cn> 488.77 </cn>
            </apply>
          </math>
        </trigger>
        <listOfEventAssignments>
          <eventAssignment
variable="mwcedbe49e_2d28_4720_8fcd_207db64228cf">
            <math xmlns="http://www.w3.org/1998/Math/MathML">
              <cn type="integer"> 1 </cn>
            </math>
          </eventAssignment>
        </listOfEventAssignments>
      </event>
      <event id="mw40268f58_7b15_4243_a327_b76f4e019fcf"
name="event_448">
```

```
        &lt;trigger&gt;
          &lt;math xmlns="http://www.w3.org/1998/Math/MathML"&gt;
            &lt;apply&gt;
              &lt;geq/&gt;
              <csymbol encoding="text"
definitionURL="http://www.sbml.org/sbml/symbols/time"> time &lt;/csymbol&gt;
              &lt;cn&gt; 490.2 &lt;/cn&gt;
            &lt;/apply&gt;
          &lt;/math&gt;
        &lt;/trigger&gt;
        &lt;listOfEventAssignments&gt;
          <eventAssignment
variable="mwcedbe49e_2d28_4720_8fcd_207db64228cf">
            &lt;math xmlns="http://www.w3.org/1998/Math/MathML"&gt;
              &lt;cn type="integer"&gt; 0 &lt;/cn&gt;
            &lt;/math&gt;
          &lt;/eventAssignment&gt;
        &lt;/listOfEventAssignments&gt;
      &lt;/event&gt;
      <event id="mwacca7d4e_37cb_426a_8a47_fc080ecc8c1c"
name="event_449">
        &lt;trigger&gt;
          &lt;math xmlns="http://www.w3.org/1998/Math/MathML"&gt;
            &lt;apply&gt;
              &lt;geq/&gt;
              <csymbol encoding="text"
definitionURL="http://www.sbml.org/sbml/symbols/time"> time &lt;/csymbol&gt;
              &lt;cn&gt; 493.07 &lt;/cn&gt;
            &lt;/apply&gt;
          &lt;/math&gt;
        &lt;/trigger&gt;
        &lt;listOfEventAssignments&gt;
          <eventAssignment
variable="mwcedbe49e_2d28_4720_8fcd_207db64228cf">
            &lt;math xmlns="http://www.w3.org/1998/Math/MathML"&gt;
              &lt;cn type="integer"&gt; 1 &lt;/cn&gt;
            &lt;/math&gt;
          &lt;/eventAssignment&gt;
        &lt;/listOfEventAssignments&gt;
      &lt;/event&gt;
      <event id="mw5df48145_f7be_455f_bff8_a0cb4ae99366"
name="event_450">
        &lt;trigger&gt;
          &lt;math xmlns="http://www.w3.org/1998/Math/MathML"&gt;
            &lt;apply&gt;
              &lt;geq/&gt;
              <csymbol encoding="text"
definitionURL="http://www.sbml.org/sbml/symbols/time"> time &lt;/csymbol&gt;
              &lt;cn&gt; 494.5 &lt;/cn&gt;
            &lt;/apply&gt;
          &lt;/math&gt;
        &lt;/trigger&gt;
```

```
        <listOfEventAssignments>
          <eventAssignment
variable="mwcedbe49e_2d28_4720_8fcd_207db64228cf">
            <math xmlns="http://www.w3.org/1998/Math/MathML">
              <cn type="integer"> 0 </cn>
            </math>
          </eventAssignment>
        </listOfEventAssignments>
      </event>
      <event id="mw71bc821c_b535_4642_a01b_b0404ab4860d"
name="event_451">
        <trigger>
          <math xmlns="http://www.w3.org/1998/Math/MathML">
            <apply>
              <geq/>
              <csymbol encoding="text"
definitionURL="http://www.sbml.org/sbml/symbols/time"> time </csymbol>
              <cn> 497.37 </cn>
            </apply>
          </math>
        </trigger>
        <listOfEventAssignments>
          <eventAssignment
variable="mwcedbe49e_2d28_4720_8fcd_207db64228cf">
            <math xmlns="http://www.w3.org/1998/Math/MathML">
              <cn type="integer"> 1 </cn>
            </math>
          </eventAssignment>
        </listOfEventAssignments>
      </event>
      <event id="mw158cebaf_c8cc_474c_8a38_aca43392fe5c"
name="event_452">
        <trigger>
          <math xmlns="http://www.w3.org/1998/Math/MathML">
            <apply>
              <geq/>
              <csymbol encoding="text"
definitionURL="http://www.sbml.org/sbml/symbols/time"> time </csymbol>
              <cn> 498.8 </cn>
            </apply>
          </math>
        </trigger>
        <listOfEventAssignments>
          <eventAssignment
variable="mwcedbe49e_2d28_4720_8fcd_207db64228cf">
            <math xmlns="http://www.w3.org/1998/Math/MathML">
              <cn type="integer"> 0 </cn>
            </math>
          </eventAssignment>
        </listOfEventAssignments>
      </event>
```

```
      <event id="mwa5a373d2_9d35_4291_a81b_60165da78daa"
name="event_453">
        <trigger>
          <math xmlns="http://www.w3.org/1998/Math/MathML">
            <apply>
              <geq/>
              <csymbol encoding="text"
definitionURL="http://www.sbml.org/sbml/symbols/time"> time </csymbol>
              <cn> 501.67 </cn>
            </apply>
          </math>
        </trigger>
        <listOfEventAssignments>
          <eventAssignment
variable="mwcedbe49e_2d28_4720_8fcd_207db64228cf">
            <math xmlns="http://www.w3.org/1998/Math/MathML">
              <cn type="integer"> 1 </cn>
            </math>
          </eventAssignment>
        </listOfEventAssignments>
      </event>
      <event id="mwe2381f2e_b96b_46a0_9378_58ef14f6c1be"
name="event_454">
        <trigger>
          <math xmlns="http://www.w3.org/1998/Math/MathML">
            <apply>
              <geq/>
              <csymbol encoding="text"
definitionURL="http://www.sbml.org/sbml/symbols/time"> time </csymbol>
              <cn> 503.1 </cn>
            </apply>
          </math>
        </trigger>
        <listOfEventAssignments>
          <eventAssignment
variable="mwcedbe49e_2d28_4720_8fcd_207db64228cf">
            <math xmlns="http://www.w3.org/1998/Math/MathML">
              <cn type="integer"> 0 </cn>
            </math>
          </eventAssignment>
        </listOfEventAssignments>
      </event>
      <event id="mw7e1a9345_df67_4094_ab47_77413a524102"
name="event_455">
        <trigger>
          <math xmlns="http://www.w3.org/1998/Math/MathML">
            <apply>
              <geq/>
              <csymbol encoding="text"
definitionURL="http://www.sbml.org/sbml/symbols/time"> time </csymbol>
              <cn> 505.97 </cn>
            </apply>
```

```
          </math>
        </trigger>
        <listOfEventAssignments>
          <eventAssignment
variable="mwcedbe49e_2d28_4720_8fcd_207db64228cf">
            <math xmlns="http://www.w3.org/1998/Math/MathML">
              <cn type="integer"> 1 </cn>
            </math>
          </eventAssignment>
        </listOfEventAssignments>
      </event>
      <event id="mw37788c18_5572_47ab_9373_572df5185aa3"
name="event_456">
        <trigger>
          <math xmlns="http://www.w3.org/1998/Math/MathML">
            <apply>
              <geq/>
              <csymbol encoding="text"
definitionURL="http://www.sbml.org/sbml/symbols/time"> time </csymbol>
              <cn> 507.4 </cn>
            </apply>
          </math>
        </trigger>
        <listOfEventAssignments>
          <eventAssignment
variable="mwcedbe49e_2d28_4720_8fcd_207db64228cf">
            <math xmlns="http://www.w3.org/1998/Math/MathML">
              <cn type="integer"> 0 </cn>
            </math>
          </eventAssignment>
        </listOfEventAssignments>
      </event>
      <event id="mw813c7e9e_6aa2_47d5_8432_ff2069688fb6"
name="event_457">
        <trigger>
          <math xmlns="http://www.w3.org/1998/Math/MathML">
            <apply>
              <geq/>
              <csymbol encoding="text"
definitionURL="http://www.sbml.org/sbml/symbols/time"> time </csymbol>
              <cn> 510.27 </cn>
            </apply>
          </math>
        </trigger>
        <listOfEventAssignments>
          <eventAssignment
variable="mwcedbe49e_2d28_4720_8fcd_207db64228cf">
            <math xmlns="http://www.w3.org/1998/Math/MathML">
              <cn type="integer"> 1 </cn>
            </math>
          </eventAssignment>
        </listOfEventAssignments>
```

```
      </event>
      <event id="mw612880a2_ac01_490b_83a6_42a2a7e51e95"
name="event_458">
        <trigger>
          <math xmlns="http://www.w3.org/1998/Math/MathML">
            <apply>
              <geq/>
              <csymbol encoding="text"
definitionURL="http://www.sbml.org/sbml/symbols/time"> time </csymbol>
              <cn> 511.7 </cn>
            </apply>
          </math>
        </trigger>
        <listOfEventAssignments>
          <eventAssignment
variable="mwcedbe49e_2d28_4720_8fcd_207db64228cf">
            <math xmlns="http://www.w3.org/1998/Math/MathML">
              <cn type="integer"> 0 </cn>
            </math>
          </eventAssignment>
        </listOfEventAssignments>
      </event>
      <event id="mw7d1138c0_8e22_4f07_a598_8dce9258800b"
name="event_459">
        <trigger>
          <math xmlns="http://www.w3.org/1998/Math/MathML">
            <apply>
              <geq/>
              <csymbol encoding="text"
definitionURL="http://www.sbml.org/sbml/symbols/time"> time </csymbol>
              <cn> 514.57 </cn>
            </apply>
          </math>
        </trigger>
        <listOfEventAssignments>
          <eventAssignment
variable="mwcedbe49e_2d28_4720_8fcd_207db64228cf">
            <math xmlns="http://www.w3.org/1998/Math/MathML">
              <cn type="integer"> 1 </cn>
            </math>
          </eventAssignment>
        </listOfEventAssignments>
      </event>
      <event id="mwa23e9c1a_6ca5_45ac_a06c_65016c97f6c2"
name="event_460">
        <trigger>
          <math xmlns="http://www.w3.org/1998/Math/MathML">
            <apply>
              <geq/>
              <csymbol encoding="text"
definitionURL="http://www.sbml.org/sbml/symbols/time"> time </csymbol>
              <cn type="integer"> 516 </cn>
```

```
          </apply>
        </math>
      </trigger>
      <listOfEventAssignments>
        <eventAssignment
variable="mwcedbe49e_2d28_4720_8fcd_207db64228cf">
          <math xmlns="http://www.w3.org/1998/Math/MathML">
            <cn type="integer"> 0 </cn>
          </math>
        </eventAssignment>
      </listOfEventAssignments>
    </event>
    <event id="mwdb67f605_cc28_478b_b243_80e331ac1ae3"
name="event_461">
      <trigger>
        <math xmlns="http://www.w3.org/1998/Math/MathML">
          <apply>
            <geq/>
            <csymbol encoding="text"
definitionURL="http://www.sbml.org/sbml/symbols/time"> time </csymbol>
            <cn> 475.87 </cn>
          </apply>
        </math>
      </trigger>
      <listOfEventAssignments>
        <eventAssignment
variable="mw7d178aae_ff78_412a_830f_3be325d22773">
          <math xmlns="http://www.w3.org/1998/Math/MathML">
            <cn type="integer"> 0 </cn>
          </math>
        </eventAssignment>
      </listOfEventAssignments>
    </event>
    <event id="mwe2c442e1_875d_484b_8076_9c846caa9a12"
name="event_462">
      <trigger>
        <math xmlns="http://www.w3.org/1998/Math/MathML">
          <apply>
            <geq/>
            <csymbol encoding="text"
definitionURL="http://www.sbml.org/sbml/symbols/time"> time </csymbol>
            <cn> 477.3 </cn>
          </apply>
        </math>
      </trigger>
      <listOfEventAssignments>
        <eventAssignment
variable="mw7d178aae_ff78_412a_830f_3be325d22773">
          <math xmlns="http://www.w3.org/1998/Math/MathML">
            <cn type="integer"> 1 </cn>
          </math>
        </eventAssignment>
```

```
        </listOfEventAssignments>
      </event>
      <event id="mw07168eec_36bc_4cbc_a639_7434cf23d7ce"
name="event_463">
        <trigger>
          <math xmlns="http://www.w3.org/1998/Math/MathML">
            <apply>
              <geq/>
              <csymbol encoding="text"
definitionURL="http://www.sbml.org/sbml/symbols/time"> time </csymbol>
              <cn> 480.17 </cn>
            </apply>
          </math>
        </trigger>
        <listOfEventAssignments>
          <eventAssignment
variable="mw7d178aae_ff78_412a_830f_3be325d22773">
            <math xmlns="http://www.w3.org/1998/Math/MathML">
              <cn type="integer"> 0 </cn>
            </math>
          </eventAssignment>
        </listOfEventAssignments>
      </event>
      <event id="mw3731b39b_8e22_40e1_8d0d_10723345eb68"
name="event_464">
        <trigger>
          <math xmlns="http://www.w3.org/1998/Math/MathML">
            <apply>
              <geq/>
              <csymbol encoding="text"
definitionURL="http://www.sbml.org/sbml/symbols/time"> time </csymbol>
              <cn> 481.6 </cn>
            </apply>
          </math>
        </trigger>
        <listOfEventAssignments>
          <eventAssignment
variable="mw7d178aae_ff78_412a_830f_3be325d22773">
            <math xmlns="http://www.w3.org/1998/Math/MathML">
              <cn type="integer"> 1 </cn>
            </math>
          </eventAssignment>
        </listOfEventAssignments>
      </event>
      <event id="mw5bda1ccf_2d21_4336_a4b6_e4139553230c"
name="event_465">
        <trigger>
          <math xmlns="http://www.w3.org/1998/Math/MathML">
            <apply>
              <geq/>
              <csymbol encoding="text"
definitionURL="http://www.sbml.org/sbml/symbols/time"> time </csymbol>
```

```
              &lt;cn&gt; 484.47 &lt;/cn&gt;
            &lt;/apply&gt;
          &lt;/math&gt;
        &lt;/trigger&gt;
        &lt;listOfEventAssignments&gt;
          <eventAssignment
variable="mw7d178aae_ff78_412a_830f_3be325d22773">
            &lt;math xmlns="http://www.w3.org/1998/Math/MathML"&gt;
              &lt;cn type="integer"&gt; 0 &lt;/cn&gt;
            &lt;/math&gt;
          &lt;/eventAssignment&gt;
        &lt;/listOfEventAssignments&gt;
      &lt;/event&gt;
      <event id="mwa353adb8_7847_4ee1_838f_d51907d4a28c"
name="event_466">
        &lt;trigger&gt;
          &lt;math xmlns="http://www.w3.org/1998/Math/MathML"&gt;
            &lt;apply&gt;
              &lt;geq/&gt;
              <csymbol encoding="text"
definitionURL="http://www.sbml.org/sbml/symbols/time"> time &lt;/csymbol&gt;
              &lt;cn&gt; 485.9 &lt;/cn&gt;
            &lt;/apply&gt;
          &lt;/math&gt;
        &lt;/trigger&gt;
        &lt;listOfEventAssignments&gt;
          <eventAssignment
variable="mw7d178aae_ff78_412a_830f_3be325d22773">
            &lt;math xmlns="http://www.w3.org/1998/Math/MathML"&gt;
              &lt;cn type="integer"&gt; 1 &lt;/cn&gt;
            &lt;/math&gt;
          &lt;/eventAssignment&gt;
        &lt;/listOfEventAssignments&gt;
      &lt;/event&gt;
      <event id="mw3bb2bcf8_f1e7_4611_aa69_515d7d731d62"
name="event_467">
        &lt;trigger&gt;
          &lt;math xmlns="http://www.w3.org/1998/Math/MathML"&gt;
            &lt;apply&gt;
              &lt;geq/&gt;
              <csymbol encoding="text"
definitionURL="http://www.sbml.org/sbml/symbols/time"> time &lt;/csymbol&gt;
              &lt;cn&gt; 488.77 &lt;/cn&gt;
            &lt;/apply&gt;
          &lt;/math&gt;
        &lt;/trigger&gt;
        &lt;listOfEventAssignments&gt;
          <eventAssignment
variable="mw7d178aae_ff78_412a_830f_3be325d22773">
            &lt;math xmlns="http://www.w3.org/1998/Math/MathML"&gt;
              &lt;cn type="integer"&gt; 0 &lt;/cn&gt;
            &lt;/math&gt;
```

```
          &lt;/eventAssignment&gt;
        &lt;/listOfEventAssignments&gt;
      &lt;/event&gt;
      <event id="mw9bb81041_74f6_42bb_a58b_c9b4ea72d403"
name="event_468">
        &lt;trigger&gt;
          &lt;math xmlns="http://www.w3.org/1998/Math/MathML"&gt;
            &lt;apply&gt;
              &lt;geq/&gt;
              <csymbol encoding="text"
definitionURL="http://www.sbml.org/sbml/symbols/time"> time &lt;/csymbol&gt;
              &lt;cn&gt; 490.2 &lt;/cn&gt;
            &lt;/apply&gt;
          &lt;/math&gt;
        &lt;/trigger&gt;
        &lt;listOfEventAssignments&gt;
          <eventAssignment
variable="mw7d178aae_ff78_412a_830f_3be325d22773">
            &lt;math xmlns="http://www.w3.org/1998/Math/MathML"&gt;
              &lt;cn type="integer"&gt; 1 &lt;/cn&gt;
            &lt;/math&gt;
          &lt;/eventAssignment&gt;
        &lt;/listOfEventAssignments&gt;
      &lt;/event&gt;
      <event id="mw0aa0edde_5504_49e4_9d5e_3a23c95e329d"
name="event_469">
        &lt;trigger&gt;
          &lt;math xmlns="http://www.w3.org/1998/Math/MathML"&gt;
            &lt;apply&gt;
              &lt;geq/&gt;
              <csymbol encoding="text"
definitionURL="http://www.sbml.org/sbml/symbols/time"> time &lt;/csymbol&gt;
              &lt;cn&gt; 493.07 &lt;/cn&gt;
            &lt;/apply&gt;
          &lt;/math&gt;
        &lt;/trigger&gt;
        &lt;listOfEventAssignments&gt;
          <eventAssignment
variable="mw7d178aae_ff78_412a_830f_3be325d22773">
            &lt;math xmlns="http://www.w3.org/1998/Math/MathML"&gt;
              &lt;cn type="integer"&gt; 0 &lt;/cn&gt;
            &lt;/math&gt;
          &lt;/eventAssignment&gt;
        &lt;/listOfEventAssignments&gt;
      &lt;/event&gt;
      <event id="mw0223b5b3_fed0_47b4_9eca_b321d6a61a8e"
name="event_470">
        &lt;trigger&gt;
          &lt;math xmlns="http://www.w3.org/1998/Math/MathML"&gt;
            &lt;apply&gt;
              &lt;geq/&gt;
```

```
              <csymbol encoding="text"
definitionURL="http://www.sbml.org/sbml/symbols/time"> time </csymbol>
              <cn> 494.5 </cn>
            </apply>
          </math>
        </trigger>
        <listOfEventAssignments>
          <eventAssignment
variable="mw7d178aae_ff78_412a_830f_3be325d22773">
            <math xmlns="http://www.w3.org/1998/Math/MathML">
              <cn type="integer"> 1 </cn>
            </math>
          </eventAssignment>
        </listOfEventAssignments>
      </event>
      <event id="mwc3df1827_bcc3_4ac5_b728_c86f577b0605"
name="event_471">
        <trigger>
          <math xmlns="http://www.w3.org/1998/Math/MathML">
            <apply>
              <geq/>
              <csymbol encoding="text"
definitionURL="http://www.sbml.org/sbml/symbols/time"> time </csymbol>
              <cn> 497.37 </cn>
            </apply>
          </math>
        </trigger>
        <listOfEventAssignments>
          <eventAssignment
variable="mw7d178aae_ff78_412a_830f_3be325d22773">
            <math xmlns="http://www.w3.org/1998/Math/MathML">
              <cn type="integer"> 0 </cn>
            </math>
          </eventAssignment>
        </listOfEventAssignments>
      </event>
      <event id="mwf3f59e99_6d0c_44bc_94e9_559df027fa61"
name="event_472">
        <trigger>
          <math xmlns="http://www.w3.org/1998/Math/MathML">
            <apply>
              <geq/>
              <csymbol encoding="text"
definitionURL="http://www.sbml.org/sbml/symbols/time"> time </csymbol>
              <cn> 498.8 </cn>
            </apply>
          </math>
        </trigger>
        <listOfEventAssignments>
          <eventAssignment
variable="mw7d178aae_ff78_412a_830f_3be325d22773">
            <math xmlns="http://www.w3.org/1998/Math/MathML">
```

```
            <cn type="integer"> 1 </cn>
          </math>
        </eventAssignment>
      </listOfEventAssignments>
    </event>
    <event id="mw019a24a7_bf6d_422a_81d6_e049b33c86fd"
name="event_473">
      <trigger>
        <math xmlns="http://www.w3.org/1998/Math/MathML">
          <apply>
            <geq/>
            <csymbol encoding="text"
definitionURL="http://www.sbml.org/sbml/symbols/time"> time </csymbol>
            <cn> 501.67 </cn>
          </apply>
        </math>
      </trigger>
      <listOfEventAssignments>
        <eventAssignment
variable="mw7d178aae_ff78_412a_830f_3be325d22773">
          <math xmlns="http://www.w3.org/1998/Math/MathML">
            <cn type="integer"> 0 </cn>
          </math>
        </eventAssignment>
      </listOfEventAssignments>
    </event>
    <event id="mw4f3c165f_4888_4f1e_9f2a_d71fa5ff7b4b"
name="event_474">
      <trigger>
        <math xmlns="http://www.w3.org/1998/Math/MathML">
          <apply>
            <geq/>
            <csymbol encoding="text"
definitionURL="http://www.sbml.org/sbml/symbols/time"> time </csymbol>
            <cn> 503.1 </cn>
          </apply>
        </math>
      </trigger>
      <listOfEventAssignments>
        <eventAssignment
variable="mw7d178aae_ff78_412a_830f_3be325d22773">
          <math xmlns="http://www.w3.org/1998/Math/MathML">
            <cn type="integer"> 1 </cn>
          </math>
        </eventAssignment>
      </listOfEventAssignments>
    </event>
    <event id="mwc92a78a1_0853_4dae_a71d_ae6e402dc5eb"
name="event_475">
      <trigger>
        <math xmlns="http://www.w3.org/1998/Math/MathML">
          <apply>
```

```
              <geq/>
              <csymbol encoding="text"
definitionURL="http://www.sbml.org/sbml/symbols/time"> time </csymbol>
              <cn> 505.97 </cn>
            </apply>
          </math>
        </trigger>
        <listOfEventAssignments>
          <eventAssignment
variable="mw7d178aae_ff78_412a_830f_3be325d22773">
            <math xmlns="http://www.w3.org/1998/Math/MathML">
              <cn type="integer"> 0 </cn>
            </math>
          </eventAssignment>
        </listOfEventAssignments>
      </event>
      <event id="mw692d8282_c559_4896_8b6d_450bebe600ca"
name="event_476">
        <trigger>
          <math xmlns="http://www.w3.org/1998/Math/MathML">
            <apply>
              <geq/>
              <csymbol encoding="text"
definitionURL="http://www.sbml.org/sbml/symbols/time"> time </csymbol>
              <cn> 507.4 </cn>
            </apply>
          </math>
        </trigger>
        <listOfEventAssignments>
          <eventAssignment
variable="mw7d178aae_ff78_412a_830f_3be325d22773">
            <math xmlns="http://www.w3.org/1998/Math/MathML">
              <cn type="integer"> 1 </cn>
            </math>
          </eventAssignment>
        </listOfEventAssignments>
      </event>
      <event id="mw4792a881_4742_45a6_816e_8e21c317cdc1"
name="event_477">
        <trigger>
          <math xmlns="http://www.w3.org/1998/Math/MathML">
            <apply>
              <geq/>
              <csymbol encoding="text"
definitionURL="http://www.sbml.org/sbml/symbols/time"> time </csymbol>
              <cn> 510.27 </cn>
            </apply>
          </math>
        </trigger>
        <listOfEventAssignments>
          <eventAssignment
variable="mw7d178aae_ff78_412a_830f_3be325d22773">
```

```
            <math xmlns="http://www.w3.org/1998/Math/MathML">
              <cn type="integer"> 0 </cn>
            </math>
          </eventAssignment>
        </listOfEventAssignments>
      </event>
      <event id="mwdf2adf8b_39c4_40f8_932e_a91cf54c95d0"
name="event_478">
        <trigger>
          <math xmlns="http://www.w3.org/1998/Math/MathML">
            <apply>
              <geq/>
              <csymbol encoding="text"
definitionURL="http://www.sbml.org/sbml/symbols/time"> time </csymbol>
              <cn> 511.7 </cn>
            </apply>
          </math>
        </trigger>
        <listOfEventAssignments>
          <eventAssignment
variable="mw7d178aae_ff78_412a_830f_3be325d22773">
            <math xmlns="http://www.w3.org/1998/Math/MathML">
              <cn type="integer"> 1 </cn>
            </math>
          </eventAssignment>
        </listOfEventAssignments>
      </event>
      <event id="mwdb5e66ed_4bba_4dea_be72_b0aa86240fa0"
name="event_479">
        <trigger>
          <math xmlns="http://www.w3.org/1998/Math/MathML">
            <apply>
              <geq/>
              <csymbol encoding="text"
definitionURL="http://www.sbml.org/sbml/symbols/time"> time </csymbol>
              <cn> 514.57 </cn>
            </apply>
          </math>
        </trigger>
        <listOfEventAssignments>
          <eventAssignment
variable="mw7d178aae_ff78_412a_830f_3be325d22773">
            <math xmlns="http://www.w3.org/1998/Math/MathML">
              <cn type="integer"> 0 </cn>
            </math>
          </eventAssignment>
        </listOfEventAssignments>
      </event>
      <event id="mw6527e621_93b1_4f3c_acb2_842348c1621b"
name="event_480">
        <trigger>
          <math xmlns="http://www.w3.org/1998/Math/MathML">
```

```
            <apply>
              <geq/>
              <csymbol encoding="text"
definitionURL="http://www.sbml.org/sbml/symbols/time"> time </csymbol>
              <cn type="integer"> 516 </cn>
            </apply>
          </math>
        </trigger>
        <listOfEventAssignments>
          <eventAssignment
variable="mw7d178aae_ff78_412a_830f_3be325d22773">
            <math xmlns="http://www.w3.org/1998/Math/MathML">
              <cn type="integer"> 1 </cn>
            </math>
          </eventAssignment>
        </listOfEventAssignments>
      </event>
      <event id="mwd0f994f4_56ba_409c_b073_f02a9d8bdf67"
name="event_481">
        <trigger>
          <math xmlns="http://www.w3.org/1998/Math/MathML">
            <apply>
              <geq/>
              <csymbol encoding="text"
definitionURL="http://www.sbml.org/sbml/symbols/time"> time </csymbol>
              <cn> 518.87 </cn>
            </apply>
          </math>
        </trigger>
        <listOfEventAssignments>
          <eventAssignment
variable="mw7d178aae_ff78_412a_830f_3be325d22773">
            <math xmlns="http://www.w3.org/1998/Math/MathML">
              <cn type="integer"> 0 </cn>
            </math>
          </eventAssignment>
        </listOfEventAssignments>
      </event>
      <event id="mw550f9fdb_eaf8_44aa_bb25_9c63dccdbba4"
name="event_482">
        <trigger>
          <math xmlns="http://www.w3.org/1998/Math/MathML">
            <apply>
              <geq/>
              <csymbol encoding="text"
definitionURL="http://www.sbml.org/sbml/symbols/time"> time </csymbol>
              <cn> 520.3 </cn>
            </apply>
          </math>
        </trigger>
        <listOfEventAssignments>
```

```
          <eventAssignment
variable="mw7d178aae_ff78_412a_830f_3be325d22773">
            &lt;math xmlns="http://www.w3.org/1998/Math/MathML"&gt;
              &lt;cn type="integer"&gt; 1 &lt;/cn&gt;
            &lt;/math&gt;
          &lt;/eventAssignment&gt;
        &lt;/listOfEventAssignments&gt;
      &lt;/event&gt;
      <event id="mw63d3f502_ce8e_4905_ba76_44abfd1a766e"
name="event_483">
        &lt;trigger&gt;
          &lt;math xmlns="http://www.w3.org/1998/Math/MathML"&gt;
            &lt;apply&gt;
              &lt;geq/&gt;
              <csymbol encoding="text"
definitionURL="http://www.sbml.org/sbml/symbols/time"> time &lt;/csymbol&gt;
              &lt;cn&gt; 523.17 &lt;/cn&gt;
            &lt;/apply&gt;
          &lt;/math&gt;
        &lt;/trigger&gt;
        &lt;listOfEventAssignments&gt;
          <eventAssignment
variable="mw7d178aae_ff78_412a_830f_3be325d22773">
            &lt;math xmlns="http://www.w3.org/1998/Math/MathML"&gt;
              &lt;cn type="integer"&gt; 0 &lt;/cn&gt;
            &lt;/math&gt;
          &lt;/eventAssignment&gt;
        &lt;/listOfEventAssignments&gt;
      &lt;/event&gt;
      <event id="mw50a4254f_3b16_40db_81df_1f87aec40ad2"
name="event_484">
        &lt;trigger&gt;
          &lt;math xmlns="http://www.w3.org/1998/Math/MathML"&gt;
            &lt;apply&gt;
              &lt;geq/&gt;
              <csymbol encoding="text"
definitionURL="http://www.sbml.org/sbml/symbols/time"> time &lt;/csymbol&gt;
              &lt;cn&gt; 524.6 &lt;/cn&gt;
            &lt;/apply&gt;
          &lt;/math&gt;
        &lt;/trigger&gt;
        &lt;listOfEventAssignments&gt;
          <eventAssignment
variable="mw7d178aae_ff78_412a_830f_3be325d22773">
            &lt;math xmlns="http://www.w3.org/1998/Math/MathML"&gt;
              &lt;cn type="integer"&gt; 1 &lt;/cn&gt;
            &lt;/math&gt;
          &lt;/eventAssignment&gt;
        &lt;/listOfEventAssignments&gt;
      &lt;/event&gt;
      <event id="mw7cd79289_5340_489c_817f_f9281fc70fca"
name="event_485">
```

```
        &lt;trigger&gt;
          &lt;math xmlns="http://www.w3.org/1998/Math/MathML"&gt;
            &lt;apply&gt;
              &lt;geq/&gt;
              <csymbol encoding="text"
definitionURL="http://www.sbml.org/sbml/symbols/time"> time &lt;/csymbol&gt;
              &lt;cn&gt; 527.47 &lt;/cn&gt;
            &lt;/apply&gt;
          &lt;/math&gt;
        &lt;/trigger&gt;
        &lt;listOfEventAssignments&gt;
          <eventAssignment
variable="mw7d178aae_ff78_412a_830f_3be325d22773">
            &lt;math xmlns="http://www.w3.org/1998/Math/MathML"&gt;
              &lt;cn type="integer"&gt; 0 &lt;/cn&gt;
            &lt;/math&gt;
          &lt;/eventAssignment&gt;
        &lt;/listOfEventAssignments&gt;
      &lt;/event&gt;
      <event id="mw7a784c16_ed13_481e_b8a4_03174108afc6"
name="event_486">
        &lt;trigger&gt;
          &lt;math xmlns="http://www.w3.org/1998/Math/MathML"&gt;
            &lt;apply&gt;
              &lt;geq/&gt;
              <csymbol encoding="text"
definitionURL="http://www.sbml.org/sbml/symbols/time"> time &lt;/csymbol&gt;
              &lt;cn&gt; 528.9 &lt;/cn&gt;
            &lt;/apply&gt;
          &lt;/math&gt;
        &lt;/trigger&gt;
        &lt;listOfEventAssignments&gt;
          <eventAssignment
variable="mw7d178aae_ff78_412a_830f_3be325d22773">
            &lt;math xmlns="http://www.w3.org/1998/Math/MathML"&gt;
              &lt;cn type="integer"&gt; 1 &lt;/cn&gt;
            &lt;/math&gt;
          &lt;/eventAssignment&gt;
        &lt;/listOfEventAssignments&gt;
      &lt;/event&gt;
      <event id="mwef4c07f0_eb21_4116_af69_8e98accbf710"
name="event_487">
        &lt;trigger&gt;
          &lt;math xmlns="http://www.w3.org/1998/Math/MathML"&gt;
            &lt;apply&gt;
              &lt;geq/&gt;
              <csymbol encoding="text"
definitionURL="http://www.sbml.org/sbml/symbols/time"> time &lt;/csymbol&gt;
              &lt;cn&gt; 531.77 &lt;/cn&gt;
            &lt;/apply&gt;
          &lt;/math&gt;
        &lt;/trigger&gt;
```

```
        <listOfEventAssignments>
          <eventAssignment
variable="mw7d178aae_ff78_412a_830f_3be325d22773">
            <math xmlns="http://www.w3.org/1998/Math/MathML">
              <cn type="integer"> 0 </cn>
            </math>
          </eventAssignment>
        </listOfEventAssignments>
      </event>
      <event id="mw9e087644_c2ce_4056_8773_f9f6687784d5"
name="event_488">
        <trigger>
          <math xmlns="http://www.w3.org/1998/Math/MathML">
            <apply>
              <geq/>
              <csymbol encoding="text"
definitionURL="http://www.sbml.org/sbml/symbols/time"> time </csymbol>
              <cn> 533.2 </cn>
            </apply>
          </math>
        </trigger>
        <listOfEventAssignments>
          <eventAssignment
variable="mw7d178aae_ff78_412a_830f_3be325d22773">
            <math xmlns="http://www.w3.org/1998/Math/MathML">
              <cn type="integer"> 1 </cn>
            </math>
          </eventAssignment>
        </listOfEventAssignments>
      </event>
      <event id="mw15f3c05e_2542_40dc_8e95_082b2c41e0c2"
name="event_489">
        <trigger>
          <math xmlns="http://www.w3.org/1998/Math/MathML">
            <apply>
              <geq/>
              <csymbol encoding="text"
definitionURL="http://www.sbml.org/sbml/symbols/time"> time </csymbol>
              <cn> 536.07 </cn>
            </apply>
          </math>
        </trigger>
        <listOfEventAssignments>
          <eventAssignment
variable="mw7d178aae_ff78_412a_830f_3be325d22773">
            <math xmlns="http://www.w3.org/1998/Math/MathML">
              <cn type="integer"> 0 </cn>
            </math>
          </eventAssignment>
        </listOfEventAssignments>
      </event>
```

```
      <event id="mwfe8d38c7_2264_437b_a7c9_935995ec6fcf"
name="event_490">
        &lt;trigger&gt;
          &lt;math xmlns="http://www.w3.org/1998/Math/MathML"&gt;
            &lt;apply&gt;
              &lt;geq/&gt;
              <csymbol encoding="text"
definitionURL="http://www.sbml.org/sbml/symbols/time"> time &lt;/csymbol&gt;
              &lt;cn&gt; 537.5 &lt;/cn&gt;
            &lt;/apply&gt;
          &lt;/math&gt;
        &lt;/trigger&gt;
        &lt;listOfEventAssignments&gt;
          <eventAssignment
variable="mw7d178aae_ff78_412a_830f_3be325d22773">
            &lt;math xmlns="http://www.w3.org/1998/Math/MathML"&gt;
              &lt;cn type="integer"&gt; 1 &lt;/cn&gt;
            &lt;/math&gt;
          &lt;/eventAssignment&gt;
        &lt;/listOfEventAssignments&gt;
      &lt;/event&gt;
      <event id="mw4857c608_9fc3_49ac_88a4_49dc29887164"
name="event_491">
        &lt;trigger&gt;
          &lt;math xmlns="http://www.w3.org/1998/Math/MathML"&gt;
            &lt;apply&gt;
              &lt;geq/&gt;
              <csymbol encoding="text"
definitionURL="http://www.sbml.org/sbml/symbols/time"> time &lt;/csymbol&gt;
              &lt;cn&gt; 540.37 &lt;/cn&gt;
            &lt;/apply&gt;
          &lt;/math&gt;
        &lt;/trigger&gt;
        &lt;listOfEventAssignments&gt;
          <eventAssignment
variable="mw7d178aae_ff78_412a_830f_3be325d22773">
            &lt;math xmlns="http://www.w3.org/1998/Math/MathML"&gt;
              &lt;cn type="integer"&gt; 0 &lt;/cn&gt;
            &lt;/math&gt;
          &lt;/eventAssignment&gt;
        &lt;/listOfEventAssignments&gt;
      &lt;/event&gt;
      <event id="mw21c10446_81f4_4ffb_9806_aa0f21aafd01"
name="event_492">
        &lt;trigger&gt;
          &lt;math xmlns="http://www.w3.org/1998/Math/MathML"&gt;
            &lt;apply&gt;
              &lt;geq/&gt;
              <csymbol encoding="text"
definitionURL="http://www.sbml.org/sbml/symbols/time"> time &lt;/csymbol&gt;
              &lt;cn&gt; 541.8 &lt;/cn&gt;
            &lt;/apply&gt;
```

```
          </math>
        </trigger>
        <listOfEventAssignments>
          <eventAssignment
variable="mw7d178aae_ff78_412a_830f_3be325d22773">
            <math xmlns="http://www.w3.org/1998/Math/MathML">
              <cn type="integer"> 1 </cn>
            </math>
          </eventAssignment>
        </listOfEventAssignments>
      </event>
      <event id="mwa3ea9cbe_364e_43cd_985b_986717010b00"
name="event_493">
        <trigger>
          <math xmlns="http://www.w3.org/1998/Math/MathML">
            <apply>
              <geq/>
              <csymbol encoding="text"
definitionURL="http://www.sbml.org/sbml/symbols/time"> time </csymbol>
              <cn> 544.67 </cn>
            </apply>
          </math>
        </trigger>
        <listOfEventAssignments>
          <eventAssignment
variable="mw7d178aae_ff78_412a_830f_3be325d22773">
            <math xmlns="http://www.w3.org/1998/Math/MathML">
              <cn type="integer"> 0 </cn>
            </math>
          </eventAssignment>
        </listOfEventAssignments>
      </event>
      <event id="mwbc21fd5e_4e3f_4464_b1df_5f04dc022a8a"
name="event_494">
        <trigger>
          <math xmlns="http://www.w3.org/1998/Math/MathML">
            <apply>
              <geq/>
              <csymbol encoding="text"
definitionURL="http://www.sbml.org/sbml/symbols/time"> time </csymbol>
              <cn> 546.1 </cn>
            </apply>
          </math>
        </trigger>
        <listOfEventAssignments>
          <eventAssignment
variable="mw7d178aae_ff78_412a_830f_3be325d22773">
            <math xmlns="http://www.w3.org/1998/Math/MathML">
              <cn type="integer"> 1 </cn>
            </math>
          </eventAssignment>
        </listOfEventAssignments>
```

```
      &lt;/event&gt;
      <event id="mw2eab3d49_9925_4479_9202_ba0fc0168d09"
name="event_495">
        &lt;trigger&gt;
          &lt;math xmlns="http://www.w3.org/1998/Math/MathML"&gt;
            &lt;apply&gt;
              &lt;geq/&gt;
              <csymbol encoding="text"
definitionURL="http://www.sbml.org/sbml/symbols/time"> time &lt;/csymbol&gt;
              &lt;cn&gt; 548.97 &lt;/cn&gt;
            &lt;/apply&gt;
          &lt;/math&gt;
        &lt;/trigger&gt;
        &lt;listOfEventAssignments&gt;
          <eventAssignment
variable="mw7d178aae_ff78_412a_830f_3be325d22773">
            &lt;math xmlns="http://www.w3.org/1998/Math/MathML"&gt;
              &lt;cn type="integer"&gt; 0 &lt;/cn&gt;
            &lt;/math&gt;
          &lt;/eventAssignment&gt;
        &lt;/listOfEventAssignments&gt;
      &lt;/event&gt;
      <event id="mw0e8637a5_0794_4e2a_b612_3283f0b7cb05"
name="event_496">
        &lt;trigger&gt;
          &lt;math xmlns="http://www.w3.org/1998/Math/MathML"&gt;
            &lt;apply&gt;
              &lt;geq/&gt;
              <csymbol encoding="text"
definitionURL="http://www.sbml.org/sbml/symbols/time"> time &lt;/csymbol&gt;
              &lt;cn&gt; 550.4 &lt;/cn&gt;
            &lt;/apply&gt;
          &lt;/math&gt;
        &lt;/trigger&gt;
        &lt;listOfEventAssignments&gt;
          <eventAssignment
variable="mw7d178aae_ff78_412a_830f_3be325d22773">
            &lt;math xmlns="http://www.w3.org/1998/Math/MathML"&gt;
              &lt;cn type="integer"&gt; 1 &lt;/cn&gt;
            &lt;/math&gt;
          &lt;/eventAssignment&gt;
        &lt;/listOfEventAssignments&gt;
      &lt;/event&gt;
      <event id="mwabeb71dc_83f2_49a7_a4fb_54743ae5af36"
name="event_497">
        &lt;trigger&gt;
          &lt;math xmlns="http://www.w3.org/1998/Math/MathML"&gt;
            &lt;apply&gt;
              &lt;geq/&gt;
              <csymbol encoding="text"
definitionURL="http://www.sbml.org/sbml/symbols/time"> time &lt;/csymbol&gt;
              &lt;cn&gt; 553.27 &lt;/cn&gt;
```

```
          </apply>
        </math>
      </trigger>
      <listOfEventAssignments>
        <eventAssignment
variable="mw7d178aae_ff78_412a_830f_3be325d22773">
          <math xmlns="http://www.w3.org/1998/Math/MathML">
            <cn type="integer"> 0 </cn>
          </math>
        </eventAssignment>
      </listOfEventAssignments>
    </event>
    <event id="mw17e095fe_d560_4d63_8354_1c24bae3aeba"
name="event_498">
      <trigger>
        <math xmlns="http://www.w3.org/1998/Math/MathML">
          <apply>
            <geq/>
            <csymbol encoding="text"
definitionURL="http://www.sbml.org/sbml/symbols/time"> time </csymbol>
            <cn> 554.7 </cn>
          </apply>
        </math>
      </trigger>
      <listOfEventAssignments>
        <eventAssignment
variable="mw7d178aae_ff78_412a_830f_3be325d22773">
          <math xmlns="http://www.w3.org/1998/Math/MathML">
            <cn type="integer"> 1 </cn>
          </math>
        </eventAssignment>
      </listOfEventAssignments>
    </event>
    <event id="mw0e9de967_7dc1_4e92_a9fa_03bfde2525d0"
name="event_499">
      <trigger>
        <math xmlns="http://www.w3.org/1998/Math/MathML">
          <apply>
            <geq/>
            <csymbol encoding="text"
definitionURL="http://www.sbml.org/sbml/symbols/time"> time </csymbol>
            <cn> 557.57 </cn>
          </apply>
        </math>
      </trigger>
      <listOfEventAssignments>
        <eventAssignment
variable="mw7d178aae_ff78_412a_830f_3be325d22773">
          <math xmlns="http://www.w3.org/1998/Math/MathML">
            <cn type="integer"> 0 </cn>
          </math>
        </eventAssignment>
```

```
        </listOfEventAssignments>
      </event>
      <event id="mw8369d6e5_8dce_4c69_aea0_1b144cc29a0f"
name="event_500">
        <trigger>
          <math xmlns="http://www.w3.org/1998/Math/MathML">
            <apply>
              <geq/>
              <csymbol encoding="text"
definitionURL="http://www.sbml.org/sbml/symbols/time"> time </csymbol>
              <cn type="integer"> 559 </cn>
            </apply>
          </math>
        </trigger>
        <listOfEventAssignments>
          <eventAssignment
variable="mw7d178aae_ff78_412a_830f_3be325d22773">
            <math xmlns="http://www.w3.org/1998/Math/MathML">
              <cn type="integer"> 1 </cn>
            </math>
          </eventAssignment>
        </listOfEventAssignments>
      </event>
      <event id="mwebb07220_60e0_49e6_a008_863b4a262c1d"
name="event_501">
        <trigger>
          <math xmlns="http://www.w3.org/1998/Math/MathML">
            <apply>
              <geq/>
              <csymbol encoding="text"
definitionURL="http://www.sbml.org/sbml/symbols/time"> time </csymbol>
              <cn> 561.87 </cn>
            </apply>
          </math>
        </trigger>
        <listOfEventAssignments>
          <eventAssignment
variable="mw7d178aae_ff78_412a_830f_3be325d22773">
            <math xmlns="http://www.w3.org/1998/Math/MathML">
              <cn type="integer"> 0 </cn>
            </math>
          </eventAssignment>
        </listOfEventAssignments>
      </event>
      <event id="mw6841196d_4e80_4cd6_abb7_18ebdbb48c7e"
name="event_502">
        <trigger>
          <math xmlns="http://www.w3.org/1998/Math/MathML">
            <apply>
              <geq/>
              <csymbol encoding="text"
definitionURL="http://www.sbml.org/sbml/symbols/time"> time </csymbol>
```

```
              <cn> 563.3 </cn>
            </apply>
          </math>
        </trigger>
        <listOfEventAssignments>
          <eventAssignment
variable="mw7d178aae_ff78_412a_830f_3be325d22773">
            <math xmlns="http://www.w3.org/1998/Math/MathML">
              <cn type="integer"> 1 </cn>
            </math>
          </eventAssignment>
        </listOfEventAssignments>
      </event>
      <event id="mw80cf9830_6c00_45bd_ae68_960c7a9f1214"
name="event_503">
        <trigger>
          <math xmlns="http://www.w3.org/1998/Math/MathML">
            <apply>
              <geq/>
              <csymbol encoding="text"
definitionURL="http://www.sbml.org/sbml/symbols/time"> time </csymbol>
              <cn> 566.17 </cn>
            </apply>
          </math>
        </trigger>
        <listOfEventAssignments>
          <eventAssignment
variable="mw7d178aae_ff78_412a_830f_3be325d22773">
            <math xmlns="http://www.w3.org/1998/Math/MathML">
              <cn type="integer"> 0 </cn>
            </math>
          </eventAssignment>
        </listOfEventAssignments>
      </event>
      <event id="mw5dbc43e3_e948_4613_a6a0_591ada874a9f"
name="event_504">
        <trigger>
          <math xmlns="http://www.w3.org/1998/Math/MathML">
            <apply>
              <geq/>
              <csymbol encoding="text"
definitionURL="http://www.sbml.org/sbml/symbols/time"> time </csymbol>
              <cn> 567.6 </cn>
            </apply>
          </math>
        </trigger>
        <listOfEventAssignments>
          <eventAssignment
variable="mw7d178aae_ff78_412a_830f_3be325d22773">
            <math xmlns="http://www.w3.org/1998/Math/MathML">
              <cn type="integer"> 1 </cn>
            </math>
```

```
          </eventAssignment>
        </listOfEventAssignments>
      </event>
      <event id="mwd83675f3_1818_481e_8ec4_97b0aa894163"
name="event_505">
        <trigger>
          <math xmlns="http://www.w3.org/1998/Math/MathML">
            <apply>
              <geq/>
              <csymbol encoding="text"
definitionURL="http://www.sbml.org/sbml/symbols/time"> time </csymbol>
              <cn> 570.47 </cn>
            </apply>
          </math>
        </trigger>
        <listOfEventAssignments>
          <eventAssignment
variable="mw7d178aae_ff78_412a_830f_3be325d22773">
            <math xmlns="http://www.w3.org/1998/Math/MathML">
              <cn type="integer"> 0 </cn>
            </math>
          </eventAssignment>
        </listOfEventAssignments>
      </event>
      <event id="mwb54a9eee_4466_4128_bc13_1ef7d2115f12"
name="event_506">
        <trigger>
          <math xmlns="http://www.w3.org/1998/Math/MathML">
            <apply>
              <geq/>
              <csymbol encoding="text"
definitionURL="http://www.sbml.org/sbml/symbols/time"> time </csymbol>
              <cn> 571.9 </cn>
            </apply>
          </math>
        </trigger>
        <listOfEventAssignments>
          <eventAssignment
variable="mw7d178aae_ff78_412a_830f_3be325d22773">
            <math xmlns="http://www.w3.org/1998/Math/MathML">
              <cn type="integer"> 1 </cn>
            </math>
          </eventAssignment>
        </listOfEventAssignments>
      </event>
      <event id="mwd9477bfc_d85c_492d_a481_fb322738a386"
name="event_507">
        <trigger>
          <math xmlns="http://www.w3.org/1998/Math/MathML">
            <apply>
              <geq/>
```

```
              <csymbol encoding="text" 
definitionURL="http://www.sbml.org/sbml/symbols/time"> time &lt;/csymbol&gt;
              &lt;cn&gt; 574.77 &lt;/cn&gt;
            &lt;/apply&gt;
          &lt;/math&gt;
        &lt;/trigger&gt;
        &lt;listOfEventAssignments&gt;
          <eventAssignment 
variable="mw7d178aae_ff78_412a_830f_3be325d22773">
            &lt;math xmlns="http://www.w3.org/1998/Math/MathML"&gt;
              &lt;cn type="integer"&gt; 0 &lt;/cn&gt;
            &lt;/math&gt;
          &lt;/eventAssignment&gt;
        &lt;/listOfEventAssignments&gt;
      &lt;/event&gt;
      <event id="mw53b2fab8_990f_40da_96b6_690d8e7c64ab" 
name="event_508">
        &lt;trigger&gt;
          &lt;math xmlns="http://www.w3.org/1998/Math/MathML"&gt;
            &lt;apply&gt;
              &lt;geq/&gt;
              <csymbol encoding="text" 
definitionURL="http://www.sbml.org/sbml/symbols/time"> time &lt;/csymbol&gt;
              &lt;cn&gt; 576.2 &lt;/cn&gt;
            &lt;/apply&gt;
          &lt;/math&gt;
        &lt;/trigger&gt;
        &lt;listOfEventAssignments&gt;
          <eventAssignment 
variable="mw7d178aae_ff78_412a_830f_3be325d22773">
            &lt;math xmlns="http://www.w3.org/1998/Math/MathML"&gt;
              &lt;cn type="integer"&gt; 1 &lt;/cn&gt;
            &lt;/math&gt;
          &lt;/eventAssignment&gt;
        &lt;/listOfEventAssignments&gt;
      &lt;/event&gt;
      <event id="mwf1286fe4_0bf8_47ab_bf33_cac57dcfc0a2" 
name="event_509">
        &lt;trigger&gt;
          &lt;math xmlns="http://www.w3.org/1998/Math/MathML"&gt;
            &lt;apply&gt;
              &lt;geq/&gt;
              <csymbol encoding="text" 
definitionURL="http://www.sbml.org/sbml/symbols/time"> time &lt;/csymbol&gt;
              &lt;cn&gt; 579.07 &lt;/cn&gt;
            &lt;/apply&gt;
          &lt;/math&gt;
        &lt;/trigger&gt;
        &lt;listOfEventAssignments&gt;
          <eventAssignment 
variable="mw7d178aae_ff78_412a_830f_3be325d22773">
            &lt;math xmlns="http://www.w3.org/1998/Math/MathML"&gt;
```

```
            <cn type="integer"> 0 </cn>
          </math>
        </eventAssignment>
      </listOfEventAssignments>
    </event>
    <event id="mwf694ef78_193f_4c00_a602_db1bca1de2ff"
name="event_510">
      <trigger>
        <math xmlns="http://www.w3.org/1998/Math/MathML">
          <apply>
            <geq/>
            <csymbol encoding="text"
definitionURL="http://www.sbml.org/sbml/symbols/time"> time </csymbol>
            <cn> 580.5 </cn>
          </apply>
        </math>
      </trigger>
      <listOfEventAssignments>
        <eventAssignment
variable="mw7d178aae_ff78_412a_830f_3be325d22773">
          <math xmlns="http://www.w3.org/1998/Math/MathML">
            <cn type="integer"> 1 </cn>
          </math>
        </eventAssignment>
      </listOfEventAssignments>
    </event>
    <event id="mw969f0118_e18e_403a_8316_881435ec22a6"
name="event_511">
      <trigger>
        <math xmlns="http://www.w3.org/1998/Math/MathML">
          <apply>
            <geq/>
            <csymbol encoding="text"
definitionURL="http://www.sbml.org/sbml/symbols/time"> time </csymbol>
            <cn> 583.37 </cn>
          </apply>
        </math>
      </trigger>
      <listOfEventAssignments>
        <eventAssignment
variable="mw7d178aae_ff78_412a_830f_3be325d22773">
          <math xmlns="http://www.w3.org/1998/Math/MathML">
            <cn type="integer"> 0 </cn>
          </math>
        </eventAssignment>
      </listOfEventAssignments>
    </event>
    <event id="mw3e1cfbb6_b570_4b0a_8be1_fd09bcb5e843"
name="event_512">
      <trigger>
        <math xmlns="http://www.w3.org/1998/Math/MathML">
          <apply>
```

```
              <geq/>
              <csymbol encoding="text"
definitionURL="http://www.sbml.org/sbml/symbols/time"> time </csymbol>
              <cn> 584.8 </cn>
            </apply>
          </math>
        </trigger>
        <listOfEventAssignments>
          <eventAssignment
variable="mw7d178aae_ff78_412a_830f_3be325d22773">
            <math xmlns="http://www.w3.org/1998/Math/MathML">
              <cn type="integer"> 1 </cn>
            </math>
          </eventAssignment>
        </listOfEventAssignments>
      </event>
      <event id="mwe7d183f4_40cd_4ac0_bd55_1ce88bf5cb65"
name="event_513">
        <trigger>
          <math xmlns="http://www.w3.org/1998/Math/MathML">
            <apply>
              <geq/>
              <csymbol encoding="text"
definitionURL="http://www.sbml.org/sbml/symbols/time"> time </csymbol>
              <cn> 587.67 </cn>
            </apply>
          </math>
        </trigger>
        <listOfEventAssignments>
          <eventAssignment
variable="mw7d178aae_ff78_412a_830f_3be325d22773">
            <math xmlns="http://www.w3.org/1998/Math/MathML">
              <cn type="integer"> 0 </cn>
            </math>
          </eventAssignment>
        </listOfEventAssignments>
      </event>
      <event id="mw8c76e55a_109b_48f4_8884_6fb4074f651b"
name="event_514">
        <trigger>
          <math xmlns="http://www.w3.org/1998/Math/MathML">
            <apply>
              <geq/>
              <csymbol encoding="text"
definitionURL="http://www.sbml.org/sbml/symbols/time"> time </csymbol>
              <cn> 589.1 </cn>
            </apply>
          </math>
        </trigger>
        <listOfEventAssignments>
          <eventAssignment
variable="mw7d178aae_ff78_412a_830f_3be325d22773">
```

```
            <math xmlns="http://www.w3.org/1998/Math/MathML">
              <cn type="integer"> 1 </cn>
            </math>
          </eventAssignment>
        </listOfEventAssignments>
      </event>
      <event id="mw3a5fc9d7_c12b_4b41_a7d0_284406a4d2c6"
name="event_515">
        <trigger>
          <math xmlns="http://www.w3.org/1998/Math/MathML">
            <apply>
              <geq/>
              <csymbol encoding="text"
definitionURL="http://www.sbml.org/sbml/symbols/time"> time </csymbol>
              <cn> 591.97 </cn>
            </apply>
          </math>
        </trigger>
        <listOfEventAssignments>
          <eventAssignment
variable="mw7d178aae_ff78_412a_830f_3be325d22773">
            <math xmlns="http://www.w3.org/1998/Math/MathML">
              <cn type="integer"> 0 </cn>
            </math>
          </eventAssignment>
        </listOfEventAssignments>
      </event>
      <event id="mw0285d77a_22c9_4444_a776_5694449d10d5"
name="event_516">
        <trigger>
          <math xmlns="http://www.w3.org/1998/Math/MathML">
            <apply>
              <geq/>
              <csymbol encoding="text"
definitionURL="http://www.sbml.org/sbml/symbols/time"> time </csymbol>
              <cn> 593.4 </cn>
            </apply>
          </math>
        </trigger>
        <listOfEventAssignments>
          <eventAssignment
variable="mw7d178aae_ff78_412a_830f_3be325d22773">
            <math xmlns="http://www.w3.org/1998/Math/MathML">
              <cn type="integer"> 1 </cn>
            </math>
          </eventAssignment>
        </listOfEventAssignments>
      </event>
      <event id="mwecd71b76_fd7c_420d_b377_7134252fd1f4"
name="event_517">
        <trigger>
          <math xmlns="http://www.w3.org/1998/Math/MathML">
```

```
            <apply>
              <geq/>
              <csymbol encoding="text"
definitionURL="http://www.sbml.org/sbml/symbols/time"> time </csymbol>
              <cn> 596.27 </cn>
            </apply>
          </math>
        </trigger>
        <listOfEventAssignments>
          <eventAssignment
variable="mw7d178aae_ff78_412a_830f_3be325d22773">
            <math xmlns="http://www.w3.org/1998/Math/MathML">
              <cn type="integer"> 0 </cn>
            </math>
          </eventAssignment>
        </listOfEventAssignments>
      </event>
      <event id="mw3242b5bd_83ad_4973_b1c2_4cf3ece78b52"
name="event_518">
        <trigger>
          <math xmlns="http://www.w3.org/1998/Math/MathML">
            <apply>
              <geq/>
              <csymbol encoding="text"
definitionURL="http://www.sbml.org/sbml/symbols/time"> time </csymbol>
              <cn> 597.7 </cn>
            </apply>
          </math>
        </trigger>
        <listOfEventAssignments>
          <eventAssignment
variable="mw7d178aae_ff78_412a_830f_3be325d22773">
            <math xmlns="http://www.w3.org/1998/Math/MathML">
              <cn type="integer"> 1 </cn>
            </math>
          </eventAssignment>
        </listOfEventAssignments>
      </event>
      <event id="mw8c0f1d34_4ad7_465f_8586_b6e71c676db2"
name="event_519">
        <trigger>
          <math xmlns="http://www.w3.org/1998/Math/MathML">
            <apply>
              <geq/>
              <csymbol encoding="text"
definitionURL="http://www.sbml.org/sbml/symbols/time"> time </csymbol>
              <cn> 600.57 </cn>
            </apply>
          </math>
        </trigger>
        <listOfEventAssignments>
```

```
          <eventAssignment
variable="mw7d178aae_ff78_412a_830f_3be325d22773">
            <math xmlns="http://www.w3.org/1998/Math/MathML">
              <cn type="integer"> 0 </cn>
            </math>
          </eventAssignment>
        </listOfEventAssignments>
      </event>
      <event id="mwd3d7145a_aea1_4ff1_80d0_724d188c73de"
name="event_520">
        <trigger>
          <math xmlns="http://www.w3.org/1998/Math/MathML">
            <apply>
              <geq/>
              <csymbol encoding="text"
definitionURL="http://www.sbml.org/sbml/symbols/time"> time </csymbol>
              <cn type="integer"> 602 </cn>
            </apply>
          </math>
        </trigger>
        <listOfEventAssignments>
          <eventAssignment
variable="mw7d178aae_ff78_412a_830f_3be325d22773">
            <math xmlns="http://www.w3.org/1998/Math/MathML">
              <cn type="integer"> 1 </cn>
            </math>
          </eventAssignment>
        </listOfEventAssignments>
      </event>
      <event id="mw851536df_3302_44a0_b5c7_870619965db1"
name="event_521">
        <trigger>
          <math xmlns="http://www.w3.org/1998/Math/MathML">
            <apply>
              <geq/>
              <csymbol encoding="text"
definitionURL="http://www.sbml.org/sbml/symbols/time"> time </csymbol>
              <cn> 518.87 </cn>
            </apply>
          </math>
        </trigger>
        <listOfEventAssignments>
          <eventAssignment
variable="mwcedbe49e_2d28_4720_8fcd_207db64228cf">
            <math xmlns="http://www.w3.org/1998/Math/MathML">
              <cn type="integer"> 1 </cn>
            </math>
          </eventAssignment>
        </listOfEventAssignments>
      </event>
      <event id="mw879cbd43_513c_4afe_a66b_63c28263d633"
name="event_522">
```

```
        &lt;trigger&gt;
          &lt;math xmlns="http://www.w3.org/1998/Math/MathML"&gt;
            &lt;apply&gt;
              &lt;geq/&gt;
              <csymbol encoding="text"
definitionURL="http://www.sbml.org/sbml/symbols/time"> time &lt;/csymbol&gt;
              &lt;cn&gt; 520.3 &lt;/cn&gt;
            &lt;/apply&gt;
          &lt;/math&gt;
        &lt;/trigger&gt;
        &lt;listOfEventAssignments&gt;
          <eventAssignment
variable="mwcedbe49e_2d28_4720_8fcd_207db64228cf">
            &lt;math xmlns="http://www.w3.org/1998/Math/MathML"&gt;
              &lt;cn type="integer"&gt; 0 &lt;/cn&gt;
            &lt;/math&gt;
          &lt;/eventAssignment&gt;
        &lt;/listOfEventAssignments&gt;
      &lt;/event&gt;
      <event id="mw55f6b33a_8488_4163_a828_149df3dd15f3"
name="event_523">
        &lt;trigger&gt;
          &lt;math xmlns="http://www.w3.org/1998/Math/MathML"&gt;
            &lt;apply&gt;
              &lt;geq/&gt;
              <csymbol encoding="text"
definitionURL="http://www.sbml.org/sbml/symbols/time"> time &lt;/csymbol&gt;
              &lt;cn&gt; 523.17 &lt;/cn&gt;
            &lt;/apply&gt;
          &lt;/math&gt;
        &lt;/trigger&gt;
        &lt;listOfEventAssignments&gt;
          <eventAssignment
variable="mwcedbe49e_2d28_4720_8fcd_207db64228cf">
            &lt;math xmlns="http://www.w3.org/1998/Math/MathML"&gt;
              &lt;cn type="integer"&gt; 1 &lt;/cn&gt;
            &lt;/math&gt;
          &lt;/eventAssignment&gt;
        &lt;/listOfEventAssignments&gt;
      &lt;/event&gt;
      <event id="mw4116a2f7_94e2_457e_a4a3_b1dacfb402b0"
name="event_524">
        &lt;trigger&gt;
          &lt;math xmlns="http://www.w3.org/1998/Math/MathML"&gt;
            &lt;apply&gt;
              &lt;geq/&gt;
              <csymbol encoding="text"
definitionURL="http://www.sbml.org/sbml/symbols/time"> time &lt;/csymbol&gt;
              &lt;cn&gt; 524.6 &lt;/cn&gt;
            &lt;/apply&gt;
          &lt;/math&gt;
        &lt;/trigger&gt;
```

```
        <listOfEventAssignments>
          <eventAssignment
variable="mwcedbe49e_2d28_4720_8fcd_207db64228cf">
            <math xmlns="http://www.w3.org/1998/Math/MathML">
              <cn type="integer"> 0 </cn>
            </math>
          </eventAssignment>
        </listOfEventAssignments>
      </event>
      <event id="mw57c5cffe_0a3b_42d3_8a1c_12ea2f651f24"
name="event_525">
        <trigger>
          <math xmlns="http://www.w3.org/1998/Math/MathML">
            <apply>
              <geq/>
              <csymbol encoding="text"
definitionURL="http://www.sbml.org/sbml/symbols/time"> time </csymbol>
              <cn> 527.47 </cn>
            </apply>
          </math>
        </trigger>
        <listOfEventAssignments>
          <eventAssignment
variable="mwcedbe49e_2d28_4720_8fcd_207db64228cf">
            <math xmlns="http://www.w3.org/1998/Math/MathML">
              <cn type="integer"> 1 </cn>
            </math>
          </eventAssignment>
        </listOfEventAssignments>
      </event>
      <event id="mw9acc2395_6ea0_48f8_af21_3a8b1d535662"
name="event_526">
        <trigger>
          <math xmlns="http://www.w3.org/1998/Math/MathML">
            <apply>
              <geq/>
              <csymbol encoding="text"
definitionURL="http://www.sbml.org/sbml/symbols/time"> time </csymbol>
              <cn> 528.9 </cn>
            </apply>
          </math>
        </trigger>
        <listOfEventAssignments>
          <eventAssignment
variable="mwcedbe49e_2d28_4720_8fcd_207db64228cf">
            <math xmlns="http://www.w3.org/1998/Math/MathML">
              <cn type="integer"> 0 </cn>
            </math>
          </eventAssignment>
        </listOfEventAssignments>
      </event>
```

```
      <event id="mwf8835fe4_216e_4f8a_acb2_75e16c5e54f3"
name="event_527">
        &lt;trigger&gt;
          &lt;math xmlns="http://www.w3.org/1998/Math/MathML"&gt;
            &lt;apply&gt;
              &lt;geq/&gt;
              <csymbol encoding="text"
definitionURL="http://www.sbml.org/sbml/symbols/time"> time &lt;/csymbol&gt;
              &lt;cn&gt; 531.77 &lt;/cn&gt;
            &lt;/apply&gt;
          &lt;/math&gt;
        &lt;/trigger&gt;
        &lt;listOfEventAssignments&gt;
          <eventAssignment
variable="mwcedbe49e_2d28_4720_8fcd_207db64228cf">
            &lt;math xmlns="http://www.w3.org/1998/Math/MathML"&gt;
              &lt;cn type="integer"&gt; 1 &lt;/cn&gt;
            &lt;/math&gt;
          &lt;/eventAssignment&gt;
        &lt;/listOfEventAssignments&gt;
      &lt;/event&gt;
      <event id="mw27d72e75_bfad_4b8a_b2b2_eb8320b70c19"
name="event_528">
        &lt;trigger&gt;
          &lt;math xmlns="http://www.w3.org/1998/Math/MathML"&gt;
            &lt;apply&gt;
              &lt;geq/&gt;
              <csymbol encoding="text"
definitionURL="http://www.sbml.org/sbml/symbols/time"> time &lt;/csymbol&gt;
              &lt;cn&gt; 533.2 &lt;/cn&gt;
            &lt;/apply&gt;
          &lt;/math&gt;
        &lt;/trigger&gt;
        &lt;listOfEventAssignments&gt;
          <eventAssignment
variable="mwcedbe49e_2d28_4720_8fcd_207db64228cf">
            &lt;math xmlns="http://www.w3.org/1998/Math/MathML"&gt;
              &lt;cn type="integer"&gt; 0 &lt;/cn&gt;
            &lt;/math&gt;
          &lt;/eventAssignment&gt;
        &lt;/listOfEventAssignments&gt;
      &lt;/event&gt;
      <event id="mwa9065b97_a376_4e0f_89ef_add56dc88808"
name="event_529">
        &lt;trigger&gt;
          &lt;math xmlns="http://www.w3.org/1998/Math/MathML"&gt;
            &lt;apply&gt;
              &lt;geq/&gt;
              <csymbol encoding="text"
definitionURL="http://www.sbml.org/sbml/symbols/time"> time &lt;/csymbol&gt;
              &lt;cn&gt; 536.07 &lt;/cn&gt;
            &lt;/apply&gt;
```

```
          </math>
        </trigger>
        <listOfEventAssignments>
          <eventAssignment
variable="mwcedbe49e_2d28_4720_8fcd_207db64228cf">
            <math xmlns="http://www.w3.org/1998/Math/MathML">
              <cn type="integer"> 1 </cn>
            </math>
          </eventAssignment>
        </listOfEventAssignments>
      </event>
      <event id="mwd97d2a63_5c5f_40cb_861e_c62ff05cb90a"
name="event_530">
        <trigger>
          <math xmlns="http://www.w3.org/1998/Math/MathML">
            <apply>
              <geq/>
              <csymbol encoding="text"
definitionURL="http://www.sbml.org/sbml/symbols/time"> time </csymbol>
              <cn> 537.5 </cn>
            </apply>
          </math>
        </trigger>
        <listOfEventAssignments>
          <eventAssignment
variable="mwcedbe49e_2d28_4720_8fcd_207db64228cf">
            <math xmlns="http://www.w3.org/1998/Math/MathML">
              <cn type="integer"> 0 </cn>
            </math>
          </eventAssignment>
        </listOfEventAssignments>
      </event>
      <event id="mwc29547a5_ed73_4d97_804b_3057547ea7ba"
name="event_531">
        <trigger>
          <math xmlns="http://www.w3.org/1998/Math/MathML">
            <apply>
              <geq/>
              <csymbol encoding="text"
definitionURL="http://www.sbml.org/sbml/symbols/time"> time </csymbol>
              <cn> 540.37 </cn>
            </apply>
          </math>
        </trigger>
        <listOfEventAssignments>
          <eventAssignment
variable="mwcedbe49e_2d28_4720_8fcd_207db64228cf">
            <math xmlns="http://www.w3.org/1998/Math/MathML">
              <cn type="integer"> 1 </cn>
            </math>
          </eventAssignment>
        </listOfEventAssignments>
```

```
      </event>
      <event id="mw5a4b9230_1af5_4883_95af_2a1228bcd0d0"
name="event_532">
        <trigger>
          <math xmlns="http://www.w3.org/1998/Math/MathML">
            <apply>
              <geq/>
              <csymbol encoding="text"
definitionURL="http://www.sbml.org/sbml/symbols/time"> time </csymbol>
              <cn> 541.8 </cn>
            </apply>
          </math>
        </trigger>
        <listOfEventAssignments>
          <eventAssignment
variable="mwcedbe49e_2d28_4720_8fcd_207db64228cf">
            <math xmlns="http://www.w3.org/1998/Math/MathML">
              <cn type="integer"> 0 </cn>
            </math>
          </eventAssignment>
        </listOfEventAssignments>
      </event>
      <event id="mw8d0add51_f0df_43ad_95e8_1e430bb1f187"
name="event_533">
        <trigger>
          <math xmlns="http://www.w3.org/1998/Math/MathML">
            <apply>
              <geq/>
              <csymbol encoding="text"
definitionURL="http://www.sbml.org/sbml/symbols/time"> time </csymbol>
              <cn> 544.67 </cn>
            </apply>
          </math>
        </trigger>
        <listOfEventAssignments>
          <eventAssignment
variable="mwcedbe49e_2d28_4720_8fcd_207db64228cf">
            <math xmlns="http://www.w3.org/1998/Math/MathML">
              <cn type="integer"> 1 </cn>
            </math>
          </eventAssignment>
        </listOfEventAssignments>
      </event>
      <event id="mwe9c74166_318b_46c7_a58e_4e8003847b04"
name="event_534">
        <trigger>
          <math xmlns="http://www.w3.org/1998/Math/MathML">
            <apply>
              <geq/>
              <csymbol encoding="text"
definitionURL="http://www.sbml.org/sbml/symbols/time"> time </csymbol>
              <cn> 546.1 </cn>
```

```
          </apply>
        </math>
      </trigger>
      <listOfEventAssignments>
        <eventAssignment
variable="mwcedbe49e_2d28_4720_8fcd_207db64228cf">
          <math xmlns="http://www.w3.org/1998/Math/MathML">
            <cn type="integer"> 0 </cn>
          </math>
        </eventAssignment>
      </listOfEventAssignments>
    </event>
    <event id="mw94c6f8b5_11b2_4d3a_bd92_ef123ef4caf1"
name="event_535">
      <trigger>
        <math xmlns="http://www.w3.org/1998/Math/MathML">
          <apply>
            <geq/>
            <csymbol encoding="text"
definitionURL="http://www.sbml.org/sbml/symbols/time"> time </csymbol>
            <cn> 548.97 </cn>
          </apply>
        </math>
      </trigger>
      <listOfEventAssignments>
        <eventAssignment
variable="mwcedbe49e_2d28_4720_8fcd_207db64228cf">
          <math xmlns="http://www.w3.org/1998/Math/MathML">
            <cn type="integer"> 1 </cn>
          </math>
        </eventAssignment>
      </listOfEventAssignments>
    </event>
    <event id="mw2c4b4030_fc0d_439b_bfb7_bcb3eda4cbb9"
name="event_536">
      <trigger>
        <math xmlns="http://www.w3.org/1998/Math/MathML">
          <apply>
            <geq/>
            <csymbol encoding="text"
definitionURL="http://www.sbml.org/sbml/symbols/time"> time </csymbol>
            <cn> 550.4 </cn>
          </apply>
        </math>
      </trigger>
      <listOfEventAssignments>
        <eventAssignment
variable="mwcedbe49e_2d28_4720_8fcd_207db64228cf">
          <math xmlns="http://www.w3.org/1998/Math/MathML">
            <cn type="integer"> 0 </cn>
          </math>
        </eventAssignment>
```

```
        &lt;/listOfEventAssignments&gt;
      &lt;/event&gt;
      <event id="mwcece09c0_31f1_4220_aeba_ec0a4090815a"
name="event_537">
        &lt;trigger&gt;
          &lt;math xmlns="http://www.w3.org/1998/Math/MathML"&gt;
            &lt;apply&gt;
              &lt;geq/&gt;
              <csymbol encoding="text"
definitionURL="http://www.sbml.org/sbml/symbols/time"> time &lt;/csymbol&gt;
              &lt;cn&gt; 553.27 &lt;/cn&gt;
            &lt;/apply&gt;
          &lt;/math&gt;
        &lt;/trigger&gt;
        &lt;listOfEventAssignments&gt;
          <eventAssignment
variable="mwcedbe49e_2d28_4720_8fcd_207db64228cf">
            &lt;math xmlns="http://www.w3.org/1998/Math/MathML"&gt;
              &lt;cn type="integer"&gt; 1 &lt;/cn&gt;
            &lt;/math&gt;
          &lt;/eventAssignment&gt;
        &lt;/listOfEventAssignments&gt;
      &lt;/event&gt;
      <event id="mwc89c5f90_4f01_4111_a2d6_5dcdb4afb851"
name="event_538">
        &lt;trigger&gt;
          &lt;math xmlns="http://www.w3.org/1998/Math/MathML"&gt;
            &lt;apply&gt;
              &lt;geq/&gt;
              <csymbol encoding="text"
definitionURL="http://www.sbml.org/sbml/symbols/time"> time &lt;/csymbol&gt;
              &lt;cn&gt; 554.7 &lt;/cn&gt;
            &lt;/apply&gt;
          &lt;/math&gt;
        &lt;/trigger&gt;
        &lt;listOfEventAssignments&gt;
          <eventAssignment
variable="mwcedbe49e_2d28_4720_8fcd_207db64228cf">
            &lt;math xmlns="http://www.w3.org/1998/Math/MathML"&gt;
              &lt;cn type="integer"&gt; 0 &lt;/cn&gt;
            &lt;/math&gt;
          &lt;/eventAssignment&gt;
        &lt;/listOfEventAssignments&gt;
      &lt;/event&gt;
      <event id="mw3ed9c346_0919_4fce_b961_fa549469a1aa"
name="event_539">
        &lt;trigger&gt;
          &lt;math xmlns="http://www.w3.org/1998/Math/MathML"&gt;
            &lt;apply&gt;
              &lt;geq/&gt;
              <csymbol encoding="text"
definitionURL="http://www.sbml.org/sbml/symbols/time"> time &lt;/csymbol&gt;
```

```
              &lt;cn&gt; 557.57 &lt;/cn&gt;
            &lt;/apply&gt;
          &lt;/math&gt;
        &lt;/trigger&gt;
        &lt;listOfEventAssignments&gt;
          <eventAssignment
variable="mwcedbe49e_2d28_4720_8fcd_207db64228cf">
            &lt;math xmlns="http://www.w3.org/1998/Math/MathML"&gt;
              &lt;cn type="integer"&gt; 1 &lt;/cn&gt;
            &lt;/math&gt;
          &lt;/eventAssignment&gt;
        &lt;/listOfEventAssignments&gt;
      &lt;/event&gt;
      <event id="mw52b4752f_0d8f_4f00_981b_6f13e8fa8b04"
name="event_540">
        &lt;trigger&gt;
          &lt;math xmlns="http://www.w3.org/1998/Math/MathML"&gt;
            &lt;apply&gt;
              &lt;geq/&gt;
              <csymbol encoding="text"
definitionURL="http://www.sbml.org/sbml/symbols/time"> time &lt;/csymbol&gt;
              &lt;cn type="integer"&gt; 559 &lt;/cn&gt;
            &lt;/apply&gt;
          &lt;/math&gt;
        &lt;/trigger&gt;
        &lt;listOfEventAssignments&gt;
          <eventAssignment
variable="mwcedbe49e_2d28_4720_8fcd_207db64228cf">
            &lt;math xmlns="http://www.w3.org/1998/Math/MathML"&gt;
              &lt;cn type="integer"&gt; 0 &lt;/cn&gt;
            &lt;/math&gt;
          &lt;/eventAssignment&gt;
        &lt;/listOfEventAssignments&gt;
      &lt;/event&gt;
      <event id="mw48676acf_1ca1_456b_957c_57dd7f48d57b"
name="event_541">
        &lt;trigger&gt;
          &lt;math xmlns="http://www.w3.org/1998/Math/MathML"&gt;
            &lt;apply&gt;
              &lt;geq/&gt;
              <csymbol encoding="text"
definitionURL="http://www.sbml.org/sbml/symbols/time"> time &lt;/csymbol&gt;
              &lt;cn&gt; 561.87 &lt;/cn&gt;
            &lt;/apply&gt;
          &lt;/math&gt;
        &lt;/trigger&gt;
        &lt;listOfEventAssignments&gt;
          <eventAssignment
variable="mwcedbe49e_2d28_4720_8fcd_207db64228cf">
            &lt;math xmlns="http://www.w3.org/1998/Math/MathML"&gt;
              &lt;cn type="integer"&gt; 1 &lt;/cn&gt;
            &lt;/math&gt;
```

```
          </eventAssignment>
        </listOfEventAssignments>
      </event>
      <event id="mw95be0464_b9a4_47b3_98a4_a6d8ad148843"
name="event_542">
        <trigger>
          <math xmlns="http://www.w3.org/1998/Math/MathML">
            <apply>
              <geq/>
              <csymbol encoding="text"
definitionURL="http://www.sbml.org/sbml/symbols/time"> time </csymbol>
              <cn> 563.3 </cn>
            </apply>
          </math>
        </trigger>
        <listOfEventAssignments>
          <eventAssignment
variable="mwcedbe49e_2d28_4720_8fcd_207db64228cf">
            <math xmlns="http://www.w3.org/1998/Math/MathML">
              <cn type="integer"> 0 </cn>
            </math>
          </eventAssignment>
        </listOfEventAssignments>
      </event>
      <event id="mwcac26a1c_6175_45be_ae71_6528aaf00d06"
name="event_543">
        <trigger>
          <math xmlns="http://www.w3.org/1998/Math/MathML">
            <apply>
              <geq/>
              <csymbol encoding="text"
definitionURL="http://www.sbml.org/sbml/symbols/time"> time </csymbol>
              <cn> 566.17 </cn>
            </apply>
          </math>
        </trigger>
        <listOfEventAssignments>
          <eventAssignment
variable="mwcedbe49e_2d28_4720_8fcd_207db64228cf">
            <math xmlns="http://www.w3.org/1998/Math/MathML">
              <cn type="integer"> 1 </cn>
            </math>
          </eventAssignment>
        </listOfEventAssignments>
      </event>
      <event id="mwe02793f2_b925_45f7_b5aa_f664559769bc"
name="event_544">
        <trigger>
          <math xmlns="http://www.w3.org/1998/Math/MathML">
            <apply>
              <geq/>
```

```
              <csymbol encoding="text" 
definitionURL="http://www.sbml.org/sbml/symbols/time"> time &lt;/csymbol&gt;
              &lt;cn&gt; 567.6 &lt;/cn&gt;
            &lt;/apply&gt;
          &lt;/math&gt;
        &lt;/trigger&gt;
        &lt;listOfEventAssignments&gt;
          <eventAssignment 
variable="mwcedbe49e_2d28_4720_8fcd_207db64228cf">
            &lt;math xmlns="http://www.w3.org/1998/Math/MathML"&gt;
              &lt;cn type="integer"&gt; 0 &lt;/cn&gt;
            &lt;/math&gt;
          &lt;/eventAssignment&gt;
        &lt;/listOfEventAssignments&gt;
      &lt;/event&gt;
      <event id="mw346d1189_f052_4be2_a482_bdf086b8120c" 
name="event_545">
        &lt;trigger&gt;
          &lt;math xmlns="http://www.w3.org/1998/Math/MathML"&gt;
            &lt;apply&gt;
              &lt;geq/&gt;
              <csymbol encoding="text" 
definitionURL="http://www.sbml.org/sbml/symbols/time"> time &lt;/csymbol&gt;
              &lt;cn&gt; 570.47 &lt;/cn&gt;
            &lt;/apply&gt;
          &lt;/math&gt;
        &lt;/trigger&gt;
        &lt;listOfEventAssignments&gt;
          <eventAssignment 
variable="mwcedbe49e_2d28_4720_8fcd_207db64228cf">
            &lt;math xmlns="http://www.w3.org/1998/Math/MathML"&gt;
              &lt;cn type="integer"&gt; 1 &lt;/cn&gt;
            &lt;/math&gt;
          &lt;/eventAssignment&gt;
        &lt;/listOfEventAssignments&gt;
      &lt;/event&gt;
      <event id="mw5ccbb564_854b_4a67_a4cb_a75633e1cd36" 
name="event_546">
        &lt;trigger&gt;
          &lt;math xmlns="http://www.w3.org/1998/Math/MathML"&gt;
            &lt;apply&gt;
              &lt;geq/&gt;
              <csymbol encoding="text" 
definitionURL="http://www.sbml.org/sbml/symbols/time"> time &lt;/csymbol&gt;
              &lt;cn&gt; 571.9 &lt;/cn&gt;
            &lt;/apply&gt;
          &lt;/math&gt;
        &lt;/trigger&gt;
        &lt;listOfEventAssignments&gt;
          <eventAssignment 
variable="mwcedbe49e_2d28_4720_8fcd_207db64228cf">
            &lt;math xmlns="http://www.w3.org/1998/Math/MathML"&gt;
```

```
              &lt;cn type="integer"&gt; 0 &lt;/cn&gt;
            &lt;/math&gt;
          &lt;/eventAssignment&gt;
        &lt;/listOfEventAssignments&gt;
      &lt;/event&gt;
      <event id="mwf1c37c54_5906_4305_8462_a63ea0f06baf"
name="event_547">
        &lt;trigger&gt;
          &lt;math xmlns="http://www.w3.org/1998/Math/MathML"&gt;
            &lt;apply&gt;
              &lt;geq/&gt;
              <csymbol encoding="text"
definitionURL="http://www.sbml.org/sbml/symbols/time"> time &lt;/csymbol&gt;
              &lt;cn&gt; 574.77 &lt;/cn&gt;
            &lt;/apply&gt;
          &lt;/math&gt;
        &lt;/trigger&gt;
        &lt;listOfEventAssignments&gt;
          <eventAssignment
variable="mwcedbe49e_2d28_4720_8fcd_207db64228cf">
            &lt;math xmlns="http://www.w3.org/1998/Math/MathML"&gt;
              &lt;cn type="integer"&gt; 1 &lt;/cn&gt;
            &lt;/math&gt;
          &lt;/eventAssignment&gt;
        &lt;/listOfEventAssignments&gt;
      &lt;/event&gt;
      <event id="mw0accde73_779a_4b94_8149_7a71597e899e"
name="event_548">
        &lt;trigger&gt;
          &lt;math xmlns="http://www.w3.org/1998/Math/MathML"&gt;
            &lt;apply&gt;
              &lt;geq/&gt;
              <csymbol encoding="text"
definitionURL="http://www.sbml.org/sbml/symbols/time"> time &lt;/csymbol&gt;
              &lt;cn&gt; 576.2 &lt;/cn&gt;
            &lt;/apply&gt;
          &lt;/math&gt;
        &lt;/trigger&gt;
        &lt;listOfEventAssignments&gt;
          <eventAssignment
variable="mwcedbe49e_2d28_4720_8fcd_207db64228cf">
            &lt;math xmlns="http://www.w3.org/1998/Math/MathML"&gt;
              &lt;cn type="integer"&gt; 0 &lt;/cn&gt;
            &lt;/math&gt;
          &lt;/eventAssignment&gt;
        &lt;/listOfEventAssignments&gt;
      &lt;/event&gt;
      <event id="mw38d3703e_71a9_43e2_9496_e39c6b433652"
name="event_549">
        &lt;trigger&gt;
          &lt;math xmlns="http://www.w3.org/1998/Math/MathML"&gt;
            &lt;apply&gt;
```

```
            <geq/>
            <csymbol encoding="text"
definitionURL="http://www.sbml.org/sbml/symbols/time"> time </csymbol>
            <cn> 579.07 </cn>
          </apply>
        </math>
      </trigger>
      <listOfEventAssignments>
        <eventAssignment
variable="mwcedbe49e_2d28_4720_8fcd_207db64228cf">
          <math xmlns="http://www.w3.org/1998/Math/MathML">
            <cn type="integer"> 1 </cn>
          </math>
        </eventAssignment>
      </listOfEventAssignments>
    </event>
    <event id="mwa6bdabe4_1d1d_421b_9c84_97cf8b8f5b58"
name="event_550">
      <trigger>
        <math xmlns="http://www.w3.org/1998/Math/MathML">
          <apply>
            <geq/>
            <csymbol encoding="text"
definitionURL="http://www.sbml.org/sbml/symbols/time"> time </csymbol>
            <cn> 580.5 </cn>
          </apply>
        </math>
      </trigger>
      <listOfEventAssignments>
        <eventAssignment
variable="mwcedbe49e_2d28_4720_8fcd_207db64228cf">
          <math xmlns="http://www.w3.org/1998/Math/MathML">
            <cn type="integer"> 0 </cn>
          </math>
        </eventAssignment>
      </listOfEventAssignments>
    </event>
    <event id="mwac47c258_84f1_4f1e_b977_8789f260fefd"
name="event_551">
      <trigger>
        <math xmlns="http://www.w3.org/1998/Math/MathML">
          <apply>
            <geq/>
            <csymbol encoding="text"
definitionURL="http://www.sbml.org/sbml/symbols/time"> time </csymbol>
            <cn> 583.37 </cn>
          </apply>
        </math>
      </trigger>
      <listOfEventAssignments>
        <eventAssignment
variable="mwcedbe49e_2d28_4720_8fcd_207db64228cf">
```

```
            <math xmlns="http://www.w3.org/1998/Math/MathML">
              <cn type="integer"> 1 </cn>
            </math>
          </eventAssignment>
        </listOfEventAssignments>
      </event>
      <event id="mw2009e446_731d_47d5_807d_29e6eb6ae48f"
name="event_552">
        <trigger>
          <math xmlns="http://www.w3.org/1998/Math/MathML">
            <apply>
              <geq/>
              <csymbol encoding="text"
definitionURL="http://www.sbml.org/sbml/symbols/time"> time </csymbol>
              <cn> 584.8 </cn>
            </apply>
          </math>
        </trigger>
        <listOfEventAssignments>
          <eventAssignment
variable="mwcedbe49e_2d28_4720_8fcd_207db64228cf">
            <math xmlns="http://www.w3.org/1998/Math/MathML">
              <cn type="integer"> 0 </cn>
            </math>
          </eventAssignment>
        </listOfEventAssignments>
      </event>
      <event id="mwc7728026_c0c7_4bee_a4d9_e756feb5a3eb"
name="event_553">
        <trigger>
          <math xmlns="http://www.w3.org/1998/Math/MathML">
            <apply>
              <geq/>
              <csymbol encoding="text"
definitionURL="http://www.sbml.org/sbml/symbols/time"> time </csymbol>
              <cn> 587.67 </cn>
            </apply>
          </math>
        </trigger>
        <listOfEventAssignments>
          <eventAssignment
variable="mwcedbe49e_2d28_4720_8fcd_207db64228cf">
            <math xmlns="http://www.w3.org/1998/Math/MathML">
              <cn type="integer"> 1 </cn>
            </math>
          </eventAssignment>
        </listOfEventAssignments>
      </event>
      <event id="mw677617d6_d52e_47d2_afd3_7ad58f188a52"
name="event_554">
        <trigger>
          <math xmlns="http://www.w3.org/1998/Math/MathML">
```

```
            &lt;apply&gt;
              &lt;geq/&gt;
              <csymbol encoding="text"
definitionURL="http://www.sbml.org/sbml/symbols/time"> time &lt;/csymbol&gt;
              &lt;cn&gt; 589.1 &lt;/cn&gt;
            &lt;/apply&gt;
          &lt;/math&gt;
        &lt;/trigger&gt;
        &lt;listOfEventAssignments&gt;
          <eventAssignment
variable="mwcedbe49e_2d28_4720_8fcd_207db64228cf">
            &lt;math xmlns="http://www.w3.org/1998/Math/MathML"&gt;
              &lt;cn type="integer"&gt; 0 &lt;/cn&gt;
            &lt;/math&gt;
          &lt;/eventAssignment&gt;
        &lt;/listOfEventAssignments&gt;
      &lt;/event&gt;
      <event id="mw1bef227c_44ac_4c77_9df2_baa7c273fff8"
name="event_555">
        &lt;trigger&gt;
          &lt;math xmlns="http://www.w3.org/1998/Math/MathML"&gt;
            &lt;apply&gt;
              &lt;geq/&gt;
              <csymbol encoding="text"
definitionURL="http://www.sbml.org/sbml/symbols/time"> time &lt;/csymbol&gt;
              &lt;cn&gt; 591.97 &lt;/cn&gt;
            &lt;/apply&gt;
          &lt;/math&gt;
        &lt;/trigger&gt;
        &lt;listOfEventAssignments&gt;
          <eventAssignment
variable="mwcedbe49e_2d28_4720_8fcd_207db64228cf">
            &lt;math xmlns="http://www.w3.org/1998/Math/MathML"&gt;
              &lt;cn type="integer"&gt; 1 &lt;/cn&gt;
            &lt;/math&gt;
          &lt;/eventAssignment&gt;
        &lt;/listOfEventAssignments&gt;
      &lt;/event&gt;
      <event id="mw47a88f79_70ce_4c08_81e0_f271e8d18c28"
name="event_556">
        &lt;trigger&gt;
          &lt;math xmlns="http://www.w3.org/1998/Math/MathML"&gt;
            &lt;apply&gt;
              &lt;geq/&gt;
              <csymbol encoding="text"
definitionURL="http://www.sbml.org/sbml/symbols/time"> time &lt;/csymbol&gt;
              &lt;cn&gt; 593.4 &lt;/cn&gt;
            &lt;/apply&gt;
          &lt;/math&gt;
        &lt;/trigger&gt;
        &lt;listOfEventAssignments&gt;
```

```
          <eventAssignment
variable="mwcedbe49e_2d28_4720_8fcd_207db64228cf">
            <math xmlns="http://www.w3.org/1998/Math/MathML">
              <cn type="integer"> 0 </cn>
            </math>
          </eventAssignment>
        </listOfEventAssignments>
      </event>
      <event id="mw1fc3bba9_88c1_4cfd_a3f5_d5826139ee15"
name="event_557">
        <trigger>
          <math xmlns="http://www.w3.org/1998/Math/MathML">
            <apply>
              <geq/>
              <csymbol encoding="text"
definitionURL="http://www.sbml.org/sbml/symbols/time"> time </csymbol>
              <cn> 596.27 </cn>
            </apply>
          </math>
        </trigger>
        <listOfEventAssignments>
          <eventAssignment
variable="mwcedbe49e_2d28_4720_8fcd_207db64228cf">
            <math xmlns="http://www.w3.org/1998/Math/MathML">
              <cn type="integer"> 1 </cn>
            </math>
          </eventAssignment>
        </listOfEventAssignments>
      </event>
      <event id="mwb9fd544c_370b_4e02_8e9d_22db439ed140"
name="event_558">
        <trigger>
          <math xmlns="http://www.w3.org/1998/Math/MathML">
            <apply>
              <geq/>
              <csymbol encoding="text"
definitionURL="http://www.sbml.org/sbml/symbols/time"> time </csymbol>
              <cn> 597.7 </cn>
            </apply>
          </math>
        </trigger>
        <listOfEventAssignments>
          <eventAssignment
variable="mwcedbe49e_2d28_4720_8fcd_207db64228cf">
            <math xmlns="http://www.w3.org/1998/Math/MathML">
              <cn type="integer"> 0 </cn>
            </math>
          </eventAssignment>
        </listOfEventAssignments>
      </event>
      <event id="mw088133c0_29c3_49cc_b5f1_1cacebc0f219"
name="event_559">
```

```
        <trigger>
          <math xmlns="http://www.w3.org/1998/Math/MathML">
            <apply>
              <geq/>
              <csymbol encoding="text"
definitionURL="http://www.sbml.org/sbml/symbols/time"> time </csymbol>
              <cn> 600.57 </cn>
            </apply>
          </math>
        </trigger>
        <listOfEventAssignments>
          <eventAssignment
variable="mwcedbe49e_2d28_4720_8fcd_207db64228cf">
            <math xmlns="http://www.w3.org/1998/Math/MathML">
              <cn type="integer"> 1 </cn>
            </math>
          </eventAssignment>
        </listOfEventAssignments>
      </event>
      <event id="mw080f7329_0b36_4276_ab0b_ae58223b591e"
name="event_560">
        <trigger>
          <math xmlns="http://www.w3.org/1998/Math/MathML">
            <apply>
              <geq/>
              <csymbol encoding="text"
definitionURL="http://www.sbml.org/sbml/symbols/time"> time </csymbol>
              <cn type="integer"> 602 </cn>
            </apply>
          </math>
        </trigger>
        <listOfEventAssignments>
          <eventAssignment
variable="mwcedbe49e_2d28_4720_8fcd_207db64228cf">
            <math xmlns="http://www.w3.org/1998/Math/MathML">
              <cn type="integer"> 0 </cn>
            </math>
          </eventAssignment>
        </listOfEventAssignments>
      </event>
      <event id="mw2d9e4999_96e6_4c0f_9da2_29970ff6b9bf"
name="event_561">
        <trigger>
          <math xmlns="http://www.w3.org/1998/Math/MathML">
            <apply>
              <geq/>
              <csymbol encoding="text"
definitionURL="http://www.sbml.org/sbml/symbols/time"> time </csymbol>
              <cn> 604.87 </cn>
            </apply>
          </math>
        </trigger>
```

```
        &lt;listOfEventAssignments&gt;
          <eventAssignment
variable="mwcedbe49e_2d28_4720_8fcd_207db64228cf">
            &lt;math xmlns="http://www.w3.org/1998/Math/MathML"&gt;
              &lt;cn type="integer"&gt; 1 &lt;/cn&gt;
            &lt;/math&gt;
          &lt;/eventAssignment&gt;
        &lt;/listOfEventAssignments&gt;
      &lt;/event&gt;
      <event id="mw7e2c9adf_5674_42fb_b623_fd3e023ad593"
name="event_562">
        &lt;trigger&gt;
          &lt;math xmlns="http://www.w3.org/1998/Math/MathML"&gt;
            &lt;apply&gt;
              &lt;geq/&gt;
              <csymbol encoding="text"
definitionURL="http://www.sbml.org/sbml/symbols/time"> time &lt;/csymbol&gt;
              &lt;cn&gt; 606.3 &lt;/cn&gt;
            &lt;/apply&gt;
          &lt;/math&gt;
        &lt;/trigger&gt;
        &lt;listOfEventAssignments&gt;
          <eventAssignment
variable="mwcedbe49e_2d28_4720_8fcd_207db64228cf">
            &lt;math xmlns="http://www.w3.org/1998/Math/MathML"&gt;
              &lt;cn type="integer"&gt; 0 &lt;/cn&gt;
            &lt;/math&gt;
          &lt;/eventAssignment&gt;
        &lt;/listOfEventAssignments&gt;
      &lt;/event&gt;
      <event id="mw6473a513_426b_4720_a015_603670fc3494"
name="event_563">
        &lt;trigger&gt;
          &lt;math xmlns="http://www.w3.org/1998/Math/MathML"&gt;
            &lt;apply&gt;
              &lt;geq/&gt;
              <csymbol encoding="text"
definitionURL="http://www.sbml.org/sbml/symbols/time"> time &lt;/csymbol&gt;
              &lt;cn&gt; 609.17 &lt;/cn&gt;
            &lt;/apply&gt;
          &lt;/math&gt;
        &lt;/trigger&gt;
        &lt;listOfEventAssignments&gt;
          <eventAssignment
variable="mwcedbe49e_2d28_4720_8fcd_207db64228cf">
            &lt;math xmlns="http://www.w3.org/1998/Math/MathML"&gt;
              &lt;cn type="integer"&gt; 1 &lt;/cn&gt;
            &lt;/math&gt;
          &lt;/eventAssignment&gt;
        &lt;/listOfEventAssignments&gt;
      &lt;/event&gt;
```

```
      <event id="mw3fd6834d_3780_411a_b8e9_d303a4d548de"
name="event_564">
        <trigger>
          <math xmlns="http://www.w3.org/1998/Math/MathML">
            <apply>
              <geq/>
              <csymbol encoding="text"
definitionURL="http://www.sbml.org/sbml/symbols/time"> time </csymbol>
              <cn> 610.6 </cn>
            </apply>
          </math>
        </trigger>
        <listOfEventAssignments>
          <eventAssignment
variable="mwcedbe49e_2d28_4720_8fcd_207db64228cf">
            <math xmlns="http://www.w3.org/1998/Math/MathML">
              <cn type="integer"> 0 </cn>
            </math>
          </eventAssignment>
        </listOfEventAssignments>
      </event>
      <event id="mw90b01465_fb39_435e_9288_d2dbcc9f65c8"
name="event_565">
        <trigger>
          <math xmlns="http://www.w3.org/1998/Math/MathML">
            <apply>
              <geq/>
              <csymbol encoding="text"
definitionURL="http://www.sbml.org/sbml/symbols/time"> time </csymbol>
              <cn> 613.47 </cn>
            </apply>
          </math>
        </trigger>
        <listOfEventAssignments>
          <eventAssignment
variable="mwcedbe49e_2d28_4720_8fcd_207db64228cf">
            <math xmlns="http://www.w3.org/1998/Math/MathML">
              <cn type="integer"> 1 </cn>
            </math>
          </eventAssignment>
        </listOfEventAssignments>
      </event>
      <event id="mw79a042d3_13f5_4e47_8fad_235d527378cd"
name="event_566">
        <trigger>
          <math xmlns="http://www.w3.org/1998/Math/MathML">
            <apply>
              <geq/>
              <csymbol encoding="text"
definitionURL="http://www.sbml.org/sbml/symbols/time"> time </csymbol>
              <cn> 614.9 </cn>
            </apply>
```

```
          </math>
        </trigger>
        <listOfEventAssignments>
          <eventAssignment
variable="mwcedbe49e_2d28_4720_8fcd_207db64228cf">
            <math xmlns="http://www.w3.org/1998/Math/MathML">
              <cn type="integer"> 0 </cn>
            </math>
          </eventAssignment>
        </listOfEventAssignments>
      </event>
      <event id="mwf6f919d2_31ab_4d7d_8213_ec98a06fc68e"
name="event_567">
        <trigger>
          <math xmlns="http://www.w3.org/1998/Math/MathML">
            <apply>
              <geq/>
              <csymbol encoding="text"
definitionURL="http://www.sbml.org/sbml/symbols/time"> time </csymbol>
              <cn> 617.77 </cn>
            </apply>
          </math>
        </trigger>
        <listOfEventAssignments>
          <eventAssignment
variable="mwcedbe49e_2d28_4720_8fcd_207db64228cf">
            <math xmlns="http://www.w3.org/1998/Math/MathML">
              <cn type="integer"> 1 </cn>
            </math>
          </eventAssignment>
        </listOfEventAssignments>
      </event>
      <event id="mwf124c170_5721_4fda_8812_6be059352fa0"
name="event_568">
        <trigger>
          <math xmlns="http://www.w3.org/1998/Math/MathML">
            <apply>
              <geq/>
              <csymbol encoding="text"
definitionURL="http://www.sbml.org/sbml/symbols/time"> time </csymbol>
              <cn> 619.2 </cn>
            </apply>
          </math>
        </trigger>
        <listOfEventAssignments>
          <eventAssignment
variable="mwcedbe49e_2d28_4720_8fcd_207db64228cf">
            <math xmlns="http://www.w3.org/1998/Math/MathML">
              <cn type="integer"> 0 </cn>
            </math>
          </eventAssignment>
        </listOfEventAssignments>
```

```
      &lt;/event&gt;
      <event id="mw0fe08c59_849d_4d6d_835e_49faed0eb7c4"
name="event_569">
        &lt;trigger&gt;
          &lt;math xmlns="http://www.w3.org/1998/Math/MathML"&gt;
            &lt;apply&gt;
              &lt;geq/&gt;
              <csymbol encoding="text"
definitionURL="http://www.sbml.org/sbml/symbols/time"> time &lt;/csymbol&gt;
              &lt;cn&gt; 622.07 &lt;/cn&gt;
            &lt;/apply&gt;
          &lt;/math&gt;
        &lt;/trigger&gt;
        &lt;listOfEventAssignments&gt;
          <eventAssignment
variable="mwcedbe49e_2d28_4720_8fcd_207db64228cf">
            &lt;math xmlns="http://www.w3.org/1998/Math/MathML"&gt;
              &lt;cn type="integer"&gt; 1 &lt;/cn&gt;
            &lt;/math&gt;
          &lt;/eventAssignment&gt;
        &lt;/listOfEventAssignments&gt;
      &lt;/event&gt;
      <event id="mwbf0c576b_3ccd_4fab_b361_da5da79ffd80"
name="event_570">
        &lt;trigger&gt;
          &lt;math xmlns="http://www.w3.org/1998/Math/MathML"&gt;
            &lt;apply&gt;
              &lt;geq/&gt;
              <csymbol encoding="text"
definitionURL="http://www.sbml.org/sbml/symbols/time"> time &lt;/csymbol&gt;
              &lt;cn&gt; 623.5 &lt;/cn&gt;
            &lt;/apply&gt;
          &lt;/math&gt;
        &lt;/trigger&gt;
        &lt;listOfEventAssignments&gt;
          <eventAssignment
variable="mwcedbe49e_2d28_4720_8fcd_207db64228cf">
            &lt;math xmlns="http://www.w3.org/1998/Math/MathML"&gt;
              &lt;cn type="integer"&gt; 0 &lt;/cn&gt;
            &lt;/math&gt;
          &lt;/eventAssignment&gt;
        &lt;/listOfEventAssignments&gt;
      &lt;/event&gt;
      <event id="mwa1aa3b11_bc01_434b_9473_5f5e67347037"
name="event_571">
        &lt;trigger&gt;
          &lt;math xmlns="http://www.w3.org/1998/Math/MathML"&gt;
            &lt;apply&gt;
              &lt;geq/&gt;
              <csymbol encoding="text"
definitionURL="http://www.sbml.org/sbml/symbols/time"> time &lt;/csymbol&gt;
              &lt;cn&gt; 626.37 &lt;/cn&gt;
```

```
          &lt;/apply&gt;
        &lt;/math&gt;
      &lt;/trigger&gt;
      &lt;listOfEventAssignments&gt;
        <eventAssignment
variable="mwcedbe49e_2d28_4720_8fcd_207db64228cf">
          &lt;math xmlns="http://www.w3.org/1998/Math/MathML"&gt;
            &lt;cn type="integer"&gt; 1 &lt;/cn&gt;
          &lt;/math&gt;
        &lt;/eventAssignment&gt;
      &lt;/listOfEventAssignments&gt;
    &lt;/event&gt;
    <event id="mw7bbe5049_6937_40d2_ba27_4de2599dc57c"
name="event_572">
      &lt;trigger&gt;
        &lt;math xmlns="http://www.w3.org/1998/Math/MathML"&gt;
          &lt;apply&gt;
            &lt;geq/&gt;
            <csymbol encoding="text"
definitionURL="http://www.sbml.org/sbml/symbols/time"> time &lt;/csymbol&gt;
            &lt;cn&gt; 627.8 &lt;/cn&gt;
          &lt;/apply&gt;
        &lt;/math&gt;
      &lt;/trigger&gt;
      &lt;listOfEventAssignments&gt;
        <eventAssignment
variable="mwcedbe49e_2d28_4720_8fcd_207db64228cf">
          &lt;math xmlns="http://www.w3.org/1998/Math/MathML"&gt;
            &lt;cn type="integer"&gt; 0 &lt;/cn&gt;
          &lt;/math&gt;
        &lt;/eventAssignment&gt;
      &lt;/listOfEventAssignments&gt;
    &lt;/event&gt;
    <event id="mwe651753a_e756_4209_90cd_0f50142edd32"
name="event_573">
      &lt;trigger&gt;
        &lt;math xmlns="http://www.w3.org/1998/Math/MathML"&gt;
          &lt;apply&gt;
            &lt;geq/&gt;
            <csymbol encoding="text"
definitionURL="http://www.sbml.org/sbml/symbols/time"> time &lt;/csymbol&gt;
            &lt;cn&gt; 630.67 &lt;/cn&gt;
          &lt;/apply&gt;
        &lt;/math&gt;
      &lt;/trigger&gt;
      &lt;listOfEventAssignments&gt;
        <eventAssignment
variable="mwcedbe49e_2d28_4720_8fcd_207db64228cf">
          &lt;math xmlns="http://www.w3.org/1998/Math/MathML"&gt;
            &lt;cn type="integer"&gt; 1 &lt;/cn&gt;
          &lt;/math&gt;
        &lt;/eventAssignment&gt;
```

```
        &lt;/listOfEventAssignments&gt;
      &lt;/event&gt;
      <event id="mwc152724e_a79b_484c_bcca_af45e962cf00"
name="event_574">
        &lt;trigger&gt;
          &lt;math xmlns="http://www.w3.org/1998/Math/MathML"&gt;
            &lt;apply&gt;
              &lt;geq/&gt;
              <csymbol encoding="text"
definitionURL="http://www.sbml.org/sbml/symbols/time"> time &lt;/csymbol&gt;
              &lt;cn&gt; 632.1 &lt;/cn&gt;
            &lt;/apply&gt;
          &lt;/math&gt;
        &lt;/trigger&gt;
        &lt;listOfEventAssignments&gt;
          <eventAssignment
variable="mwcedbe49e_2d28_4720_8fcd_207db64228cf">
            &lt;math xmlns="http://www.w3.org/1998/Math/MathML"&gt;
              &lt;cn type="integer"&gt; 0 &lt;/cn&gt;
            &lt;/math&gt;
          &lt;/eventAssignment&gt;
        &lt;/listOfEventAssignments&gt;
      &lt;/event&gt;
      <event id="mw034bc2e7_aa31_491d_b350_5f2c3d518be0"
name="event_575">
        &lt;trigger&gt;
          &lt;math xmlns="http://www.w3.org/1998/Math/MathML"&gt;
            &lt;apply&gt;
              &lt;geq/&gt;
              <csymbol encoding="text"
definitionURL="http://www.sbml.org/sbml/symbols/time"> time &lt;/csymbol&gt;
              &lt;cn&gt; 634.97 &lt;/cn&gt;
            &lt;/apply&gt;
          &lt;/math&gt;
        &lt;/trigger&gt;
        &lt;listOfEventAssignments&gt;
          <eventAssignment
variable="mwcedbe49e_2d28_4720_8fcd_207db64228cf">
            &lt;math xmlns="http://www.w3.org/1998/Math/MathML"&gt;
              &lt;cn type="integer"&gt; 1 &lt;/cn&gt;
            &lt;/math&gt;
          &lt;/eventAssignment&gt;
        &lt;/listOfEventAssignments&gt;
      &lt;/event&gt;
      <event id="mwb6cda4cf_5d87_4d4b_8d00_65a1060f186f"
name="event_576">
        &lt;trigger&gt;
          &lt;math xmlns="http://www.w3.org/1998/Math/MathML"&gt;
            &lt;apply&gt;
              &lt;geq/&gt;
              <csymbol encoding="text"
definitionURL="http://www.sbml.org/sbml/symbols/time"> time &lt;/csymbol&gt;
```

```
              <cn> 636.4 </cn>
            </apply>
          </math>
        </trigger>
        <listOfEventAssignments>
          <eventAssignment
variable="mwcedbe49e_2d28_4720_8fcd_207db64228cf">
            <math xmlns="http://www.w3.org/1998/Math/MathML">
              <cn type="integer"> 0 </cn>
            </math>
          </eventAssignment>
        </listOfEventAssignments>
      </event>
      <event id="mw60d2b9ba_2e4e_4518_a03b_e38dae90b37b"
name="event_577">
        <trigger>
          <math xmlns="http://www.w3.org/1998/Math/MathML">
            <apply>
              <geq/>
              <csymbol encoding="text"
definitionURL="http://www.sbml.org/sbml/symbols/time"> time </csymbol>
              <cn> 639.27 </cn>
            </apply>
          </math>
        </trigger>
        <listOfEventAssignments>
          <eventAssignment
variable="mwcedbe49e_2d28_4720_8fcd_207db64228cf">
            <math xmlns="http://www.w3.org/1998/Math/MathML">
              <cn type="integer"> 1 </cn>
            </math>
          </eventAssignment>
        </listOfEventAssignments>
      </event>
      <event id="mw255ce6a7_08a7_4dc5_a4af_aebce02129a0"
name="event_578">
        <trigger>
          <math xmlns="http://www.w3.org/1998/Math/MathML">
            <apply>
              <geq/>
              <csymbol encoding="text"
definitionURL="http://www.sbml.org/sbml/symbols/time"> time </csymbol>
              <cn> 640.7 </cn>
            </apply>
          </math>
        </trigger>
        <listOfEventAssignments>
          <eventAssignment
variable="mwcedbe49e_2d28_4720_8fcd_207db64228cf">
            <math xmlns="http://www.w3.org/1998/Math/MathML">
              <cn type="integer"> 0 </cn>
            </math>
```

```
          &lt;/eventAssignment&gt;
        &lt;/listOfEventAssignments&gt;
      &lt;/event&gt;
      <event id="mw684c1909_3ad0_4926_8a1c_4ce82bdf2835"
name="event_579">
        &lt;trigger&gt;
          &lt;math xmlns="http://www.w3.org/1998/Math/MathML"&gt;
            &lt;apply&gt;
              &lt;geq/&gt;
              <csymbol encoding="text"
definitionURL="http://www.sbml.org/sbml/symbols/time"> time &lt;/csymbol&gt;
              &lt;cn&gt; 643.57 &lt;/cn&gt;
            &lt;/apply&gt;
          &lt;/math&gt;
        &lt;/trigger&gt;
        &lt;listOfEventAssignments&gt;
          <eventAssignment
variable="mwcedbe49e_2d28_4720_8fcd_207db64228cf">
            &lt;math xmlns="http://www.w3.org/1998/Math/MathML"&gt;
              &lt;cn type="integer"&gt; 1 &lt;/cn&gt;
            &lt;/math&gt;
          &lt;/eventAssignment&gt;
        &lt;/listOfEventAssignments&gt;
      &lt;/event&gt;
      <event id="mwd59265f8_b2a6_4bf5_b8fd_dbfb6c7a595b"
name="event_580">
        &lt;trigger&gt;
          &lt;math xmlns="http://www.w3.org/1998/Math/MathML"&gt;
            &lt;apply&gt;
              &lt;geq/&gt;
              <csymbol encoding="text"
definitionURL="http://www.sbml.org/sbml/symbols/time"> time &lt;/csymbol&gt;
              &lt;cn type="integer"&gt; 645 &lt;/cn&gt;
            &lt;/apply&gt;
          &lt;/math&gt;
        &lt;/trigger&gt;
        &lt;listOfEventAssignments&gt;
          <eventAssignment
variable="mwcedbe49e_2d28_4720_8fcd_207db64228cf">
            &lt;math xmlns="http://www.w3.org/1998/Math/MathML"&gt;
              &lt;cn type="integer"&gt; 0 &lt;/cn&gt;
            &lt;/math&gt;
          &lt;/eventAssignment&gt;
        &lt;/listOfEventAssignments&gt;
      &lt;/event&gt;
      <event id="mw0754a7c0_cad5_433d_a472_bbfdb5cb1630"
name="event_581">
        &lt;trigger&gt;
          &lt;math xmlns="http://www.w3.org/1998/Math/MathML"&gt;
            &lt;apply&gt;
              &lt;geq/&gt;
```

```
              <csymbol encoding="text"
definitionURL="http://www.sbml.org/sbml/symbols/time"> time </csymbol>
              <cn> 647.87 </cn>
            </apply>
          </math>
        </trigger>
        <listOfEventAssignments>
          <eventAssignment
variable="mwcedbe49e_2d28_4720_8fcd_207db64228cf">
            <math xmlns="http://www.w3.org/1998/Math/MathML">
              <cn type="integer"> 1 </cn>
            </math>
          </eventAssignment>
        </listOfEventAssignments>
      </event>
      <event id="mwb17a7532_f9c6_48b1_b4a3_9e2a2e7b9dfc"
name="event_582">
        <trigger>
          <math xmlns="http://www.w3.org/1998/Math/MathML">
            <apply>
              <geq/>
              <csymbol encoding="text"
definitionURL="http://www.sbml.org/sbml/symbols/time"> time </csymbol>
              <cn> 649.3 </cn>
            </apply>
          </math>
        </trigger>
        <listOfEventAssignments>
          <eventAssignment
variable="mwcedbe49e_2d28_4720_8fcd_207db64228cf">
            <math xmlns="http://www.w3.org/1998/Math/MathML">
              <cn type="integer"> 0 </cn>
            </math>
          </eventAssignment>
        </listOfEventAssignments>
      </event>
      <event id="mw81d1f927_9e9c_4826_b14d_221c07f617b8"
name="event_583">
        <trigger>
          <math xmlns="http://www.w3.org/1998/Math/MathML">
            <apply>
              <geq/>
              <csymbol encoding="text"
definitionURL="http://www.sbml.org/sbml/symbols/time"> time </csymbol>
              <cn> 652.17 </cn>
            </apply>
          </math>
        </trigger>
        <listOfEventAssignments>
          <eventAssignment
variable="mwcedbe49e_2d28_4720_8fcd_207db64228cf">
            <math xmlns="http://www.w3.org/1998/Math/MathML">
```

```
              &lt;cn type="integer"&gt; 1 &lt;/cn&gt;
            &lt;/math&gt;
          &lt;/eventAssignment&gt;
        &lt;/listOfEventAssignments&gt;
      &lt;/event&gt;
      <event id="mw8a673b47_8432_4bc4_826e_498f192a9f6a"
name="event_584">
        &lt;trigger&gt;
          &lt;math xmlns="http://www.w3.org/1998/Math/MathML"&gt;
            &lt;apply&gt;
              &lt;geq/&gt;
              <csymbol encoding="text"
definitionURL="http://www.sbml.org/sbml/symbols/time"> time &lt;/csymbol&gt;
              &lt;cn&gt; 653.6 &lt;/cn&gt;
            &lt;/apply&gt;
          &lt;/math&gt;
        &lt;/trigger&gt;
        &lt;listOfEventAssignments&gt;
          <eventAssignment
variable="mwcedbe49e_2d28_4720_8fcd_207db64228cf">
            &lt;math xmlns="http://www.w3.org/1998/Math/MathML"&gt;
              &lt;cn type="integer"&gt; 0 &lt;/cn&gt;
            &lt;/math&gt;
          &lt;/eventAssignment&gt;
        &lt;/listOfEventAssignments&gt;
      &lt;/event&gt;
      <event id="mwadf15b99_dee7_4d5d_9b01_bd8f9bfd71a0"
name="event_585">
        &lt;trigger&gt;
          &lt;math xmlns="http://www.w3.org/1998/Math/MathML"&gt;
            &lt;apply&gt;
              &lt;geq/&gt;
              <csymbol encoding="text"
definitionURL="http://www.sbml.org/sbml/symbols/time"> time &lt;/csymbol&gt;
              &lt;cn&gt; 656.47 &lt;/cn&gt;
            &lt;/apply&gt;
          &lt;/math&gt;
        &lt;/trigger&gt;
        &lt;listOfEventAssignments&gt;
          <eventAssignment
variable="mwcedbe49e_2d28_4720_8fcd_207db64228cf">
            &lt;math xmlns="http://www.w3.org/1998/Math/MathML"&gt;
              &lt;cn type="integer"&gt; 1 &lt;/cn&gt;
            &lt;/math&gt;
          &lt;/eventAssignment&gt;
        &lt;/listOfEventAssignments&gt;
      &lt;/event&gt;
      <event id="mwc1526e48_7395_413a_bb55_d24da505a58a"
name="event_586">
        &lt;trigger&gt;
          &lt;math xmlns="http://www.w3.org/1998/Math/MathML"&gt;
            &lt;apply&gt;
```

```
              <geq/>
              <csymbol encoding="text"
definitionURL="http://www.sbml.org/sbml/symbols/time"> time </csymbol>
              <cn> 657.9 </cn>
            </apply>
          </math>
        </trigger>
        <listOfEventAssignments>
          <eventAssignment
variable="mwcedbe49e_2d28_4720_8fcd_207db64228cf">
            <math xmlns="http://www.w3.org/1998/Math/MathML">
              <cn type="integer"> 0 </cn>
            </math>
          </eventAssignment>
        </listOfEventAssignments>
      </event>
      <event id="mw46dfa013_034d_4b87_b447_ba447c26b7ea"
name="event_587">
        <trigger>
          <math xmlns="http://www.w3.org/1998/Math/MathML">
            <apply>
              <geq/>
              <csymbol encoding="text"
definitionURL="http://www.sbml.org/sbml/symbols/time"> time </csymbol>
              <cn> 660.77 </cn>
            </apply>
          </math>
        </trigger>
        <listOfEventAssignments>
          <eventAssignment
variable="mwcedbe49e_2d28_4720_8fcd_207db64228cf">
            <math xmlns="http://www.w3.org/1998/Math/MathML">
              <cn type="integer"> 1 </cn>
            </math>
          </eventAssignment>
        </listOfEventAssignments>
      </event>
      <event id="mwd672f48e_e563_41dc_88f6_4fa4cbf2bdc4"
name="event_588">
        <trigger>
          <math xmlns="http://www.w3.org/1998/Math/MathML">
            <apply>
              <geq/>
              <csymbol encoding="text"
definitionURL="http://www.sbml.org/sbml/symbols/time"> time </csymbol>
              <cn> 662.2 </cn>
            </apply>
          </math>
        </trigger>
        <listOfEventAssignments>
          <eventAssignment
variable="mwcedbe49e_2d28_4720_8fcd_207db64228cf">
```

```
            &lt;math xmlns="http://www.w3.org/1998/Math/MathML"&gt;
              &lt;cn type="integer"&gt; 0 &lt;/cn&gt;
            &lt;/math&gt;
          &lt;/eventAssignment&gt;
        &lt;/listOfEventAssignments&gt;
      &lt;/event&gt;
      <event id="mwd7e9a224_baa6_4d55_bbf5_b5de298e207e"
name="event_589">
        &lt;trigger&gt;
          &lt;math xmlns="http://www.w3.org/1998/Math/MathML"&gt;
            &lt;apply&gt;
              &lt;geq/&gt;
              <csymbol encoding="text"
definitionURL="http://www.sbml.org/sbml/symbols/time"> time &lt;/csymbol&gt;
              &lt;cn&gt; 665.07 &lt;/cn&gt;
            &lt;/apply&gt;
          &lt;/math&gt;
        &lt;/trigger&gt;
        &lt;listOfEventAssignments&gt;
          <eventAssignment
variable="mwcedbe49e_2d28_4720_8fcd_207db64228cf">
            &lt;math xmlns="http://www.w3.org/1998/Math/MathML"&gt;
              &lt;cn type="integer"&gt; 1 &lt;/cn&gt;
            &lt;/math&gt;
          &lt;/eventAssignment&gt;
        &lt;/listOfEventAssignments&gt;
      &lt;/event&gt;
      <event id="mw9cfd2086_4b9a_40c9_a953_a2791de934ee"
name="event_590">
        &lt;trigger&gt;
          &lt;math xmlns="http://www.w3.org/1998/Math/MathML"&gt;
            &lt;apply&gt;
              &lt;geq/&gt;
              <csymbol encoding="text"
definitionURL="http://www.sbml.org/sbml/symbols/time"> time &lt;/csymbol&gt;
              &lt;cn&gt; 666.5 &lt;/cn&gt;
            &lt;/apply&gt;
          &lt;/math&gt;
        &lt;/trigger&gt;
        &lt;listOfEventAssignments&gt;
          <eventAssignment
variable="mwcedbe49e_2d28_4720_8fcd_207db64228cf">
            &lt;math xmlns="http://www.w3.org/1998/Math/MathML"&gt;
              &lt;cn type="integer"&gt; 0 &lt;/cn&gt;
            &lt;/math&gt;
          &lt;/eventAssignment&gt;
        &lt;/listOfEventAssignments&gt;
      &lt;/event&gt;
      <event id="mw0d926d04_f372_48e3_a59f_b9c52a6f2fed"
name="event_591">
        &lt;trigger&gt;
          &lt;math xmlns="http://www.w3.org/1998/Math/MathML"&gt;
```

```
            <apply>
              <geq/>
              <csymbol encoding="text"
definitionURL="http://www.sbml.org/sbml/symbols/time"> time </csymbol>
              <cn> 669.37 </cn>
            </apply>
          </math>
        </trigger>
        <listOfEventAssignments>
          <eventAssignment
variable="mwcedbe49e_2d28_4720_8fcd_207db64228cf">
            <math xmlns="http://www.w3.org/1998/Math/MathML">
              <cn type="integer"> 1 </cn>
            </math>
          </eventAssignment>
        </listOfEventAssignments>
      </event>
      <event id="mw8864786d_c638_4fc3_bfda_2694ea599dcf"
name="event_592">
        <trigger>
          <math xmlns="http://www.w3.org/1998/Math/MathML">
            <apply>
              <geq/>
              <csymbol encoding="text"
definitionURL="http://www.sbml.org/sbml/symbols/time"> time </csymbol>
              <cn> 670.8 </cn>
            </apply>
          </math>
        </trigger>
        <listOfEventAssignments>
          <eventAssignment
variable="mwcedbe49e_2d28_4720_8fcd_207db64228cf">
            <math xmlns="http://www.w3.org/1998/Math/MathML">
              <cn type="integer"> 0 </cn>
            </math>
          </eventAssignment>
        </listOfEventAssignments>
      </event>
      <event id="mwd0fcc21b_2e29_4d60_a0a4_2203bc855dc6"
name="event_593">
        <trigger>
          <math xmlns="http://www.w3.org/1998/Math/MathML">
            <apply>
              <geq/>
              <csymbol encoding="text"
definitionURL="http://www.sbml.org/sbml/symbols/time"> time </csymbol>
              <cn> 673.67 </cn>
            </apply>
          </math>
        </trigger>
        <listOfEventAssignments>
```

```
          <eventAssignment
variable="mwcedbe49e_2d28_4720_8fcd_207db64228cf">
            &lt;math xmlns="http://www.w3.org/1998/Math/MathML"&gt;
              &lt;cn type="integer"&gt; 1 &lt;/cn&gt;
            &lt;/math&gt;
          &lt;/eventAssignment&gt;
        &lt;/listOfEventAssignments&gt;
      &lt;/event&gt;
      <event id="mw58cc16a8_60ce_43b2_aa28_44eafc9d47cd"
name="event_594">
        &lt;trigger&gt;
          &lt;math xmlns="http://www.w3.org/1998/Math/MathML"&gt;
            &lt;apply&gt;
              &lt;geq/&gt;
              <csymbol encoding="text"
definitionURL="http://www.sbml.org/sbml/symbols/time"> time &lt;/csymbol&gt;
              &lt;cn&gt; 675.1 &lt;/cn&gt;
            &lt;/apply&gt;
          &lt;/math&gt;
        &lt;/trigger&gt;
        &lt;listOfEventAssignments&gt;
          <eventAssignment
variable="mwcedbe49e_2d28_4720_8fcd_207db64228cf">
            &lt;math xmlns="http://www.w3.org/1998/Math/MathML"&gt;
              &lt;cn type="integer"&gt; 0 &lt;/cn&gt;
            &lt;/math&gt;
          &lt;/eventAssignment&gt;
        &lt;/listOfEventAssignments&gt;
      &lt;/event&gt;
      <event id="mwa24e3b91_8064_419e_a925_ae17b0a3084e"
name="event_595">
        &lt;trigger&gt;
          &lt;math xmlns="http://www.w3.org/1998/Math/MathML"&gt;
            &lt;apply&gt;
              &lt;geq/&gt;
              <csymbol encoding="text"
definitionURL="http://www.sbml.org/sbml/symbols/time"> time &lt;/csymbol&gt;
              &lt;cn&gt; 677.97 &lt;/cn&gt;
            &lt;/apply&gt;
          &lt;/math&gt;
        &lt;/trigger&gt;
        &lt;listOfEventAssignments&gt;
          <eventAssignment
variable="mwcedbe49e_2d28_4720_8fcd_207db64228cf">
            &lt;math xmlns="http://www.w3.org/1998/Math/MathML"&gt;
              &lt;cn type="integer"&gt; 1 &lt;/cn&gt;
            &lt;/math&gt;
          &lt;/eventAssignment&gt;
        &lt;/listOfEventAssignments&gt;
      &lt;/event&gt;
      <event id="mwe16ce66d_ed7e_4fbd_a799_8b7b43222e81"
name="event_596">
```

```
        &lt;trigger&gt;
          &lt;math xmlns="http://www.w3.org/1998/Math/MathML"&gt;
            &lt;apply&gt;
              &lt;geq/&gt;
              <csymbol encoding="text"
definitionURL="http://www.sbml.org/sbml/symbols/time"> time &lt;/csymbol&gt;
              &lt;cn&gt; 679.4 &lt;/cn&gt;
            &lt;/apply&gt;
          &lt;/math&gt;
        &lt;/trigger&gt;
        &lt;listOfEventAssignments&gt;
          <eventAssignment
variable="mwcedbe49e_2d28_4720_8fcd_207db64228cf">
            &lt;math xmlns="http://www.w3.org/1998/Math/MathML"&gt;
              &lt;cn type="integer"&gt; 0 &lt;/cn&gt;
            &lt;/math&gt;
          &lt;/eventAssignment&gt;
        &lt;/listOfEventAssignments&gt;
      &lt;/event&gt;
      <event id="mwba26417f_5a62_4a22_8b52_8517bd29ec70"
name="event_597">
        &lt;trigger&gt;
          &lt;math xmlns="http://www.w3.org/1998/Math/MathML"&gt;
            &lt;apply&gt;
              &lt;geq/&gt;
              <csymbol encoding="text"
definitionURL="http://www.sbml.org/sbml/symbols/time"> time &lt;/csymbol&gt;
              &lt;cn&gt; 682.27 &lt;/cn&gt;
            &lt;/apply&gt;
          &lt;/math&gt;
        &lt;/trigger&gt;
        &lt;listOfEventAssignments&gt;
          <eventAssignment
variable="mwcedbe49e_2d28_4720_8fcd_207db64228cf">
            &lt;math xmlns="http://www.w3.org/1998/Math/MathML"&gt;
              &lt;cn type="integer"&gt; 1 &lt;/cn&gt;
            &lt;/math&gt;
          &lt;/eventAssignment&gt;
        &lt;/listOfEventAssignments&gt;
      &lt;/event&gt;
      <event id="mw10da28bc_f02f_4143_94ab_b658b00adce4"
name="event_598">
        &lt;trigger&gt;
          &lt;math xmlns="http://www.w3.org/1998/Math/MathML"&gt;
            &lt;apply&gt;
              &lt;geq/&gt;
              <csymbol encoding="text"
definitionURL="http://www.sbml.org/sbml/symbols/time"> time &lt;/csymbol&gt;
              &lt;cn&gt; 683.7 &lt;/cn&gt;
            &lt;/apply&gt;
          &lt;/math&gt;
        &lt;/trigger&gt;
```

```
        &lt;listOfEventAssignments&gt;
          <eventAssignment
variable="mwcedbe49e_2d28_4720_8fcd_207db64228cf">
            &lt;math xmlns="http://www.w3.org/1998/Math/MathML"&gt;
              &lt;cn type="integer"&gt; 0 &lt;/cn&gt;
            &lt;/math&gt;
          &lt;/eventAssignment&gt;
        &lt;/listOfEventAssignments&gt;
      &lt;/event&gt;
      <event id="mw782bbb16_e921_4c98_b349_5120ac6a0622"
name="event_599">
        &lt;trigger&gt;
          &lt;math xmlns="http://www.w3.org/1998/Math/MathML"&gt;
            &lt;apply&gt;
              &lt;geq/&gt;
              <csymbol encoding="text"
definitionURL="http://www.sbml.org/sbml/symbols/time"> time &lt;/csymbol&gt;
              &lt;cn&gt; 686.57 &lt;/cn&gt;
            &lt;/apply&gt;
          &lt;/math&gt;
        &lt;/trigger&gt;
        &lt;listOfEventAssignments&gt;
          <eventAssignment
variable="mwcedbe49e_2d28_4720_8fcd_207db64228cf">
            &lt;math xmlns="http://www.w3.org/1998/Math/MathML"&gt;
              &lt;cn type="integer"&gt; 1 &lt;/cn&gt;
            &lt;/math&gt;
          &lt;/eventAssignment&gt;
        &lt;/listOfEventAssignments&gt;
      &lt;/event&gt;
      <event id="mwdc81acb1_fb53_4549_84a3_3a890734e814"
name="event_600">
        &lt;trigger&gt;
          &lt;math xmlns="http://www.w3.org/1998/Math/MathML"&gt;
            &lt;apply&gt;
              &lt;geq/&gt;
              <csymbol encoding="text"
definitionURL="http://www.sbml.org/sbml/symbols/time"> time &lt;/csymbol&gt;
              &lt;cn type="integer"&gt; 688 &lt;/cn&gt;
            &lt;/apply&gt;
          &lt;/math&gt;
        &lt;/trigger&gt;
        &lt;listOfEventAssignments&gt;
          <eventAssignment
variable="mwcedbe49e_2d28_4720_8fcd_207db64228cf">
            &lt;math xmlns="http://www.w3.org/1998/Math/MathML"&gt;
              &lt;cn type="integer"&gt; 0 &lt;/cn&gt;
            &lt;/math&gt;
          &lt;/eventAssignment&gt;
        &lt;/listOfEventAssignments&gt;
      &lt;/event&gt;
```

```
      <event id="mw2453635a_a6a6_4786_8790_a9c96052922f"
name="event_601">
        &lt;trigger&gt;
          &lt;math xmlns="http://www.w3.org/1998/Math/MathML"&gt;
            &lt;apply&gt;
              &lt;geq/&gt;
              <csymbol encoding="text"
definitionURL="http://www.sbml.org/sbml/symbols/time"> time &lt;/csymbol&gt;
              &lt;cn&gt; 604.87 &lt;/cn&gt;
            &lt;/apply&gt;
          &lt;/math&gt;
        &lt;/trigger&gt;
        &lt;listOfEventAssignments&gt;
          <eventAssignment
variable="mw7d178aae_ff78_412a_830f_3be325d22773">
            &lt;math xmlns="http://www.w3.org/1998/Math/MathML"&gt;
              &lt;cn type="integer"&gt; 0 &lt;/cn&gt;
            &lt;/math&gt;
          &lt;/eventAssignment&gt;
        &lt;/listOfEventAssignments&gt;
      &lt;/event&gt;
      <event id="mw714a5a3c_b750_4687_83cb_951da3e9aead"
name="event_602">
        &lt;trigger&gt;
          &lt;math xmlns="http://www.w3.org/1998/Math/MathML"&gt;
            &lt;apply&gt;
              &lt;geq/&gt;
              <csymbol encoding="text"
definitionURL="http://www.sbml.org/sbml/symbols/time"> time &lt;/csymbol&gt;
              &lt;cn&gt; 606.3 &lt;/cn&gt;
            &lt;/apply&gt;
          &lt;/math&gt;
        &lt;/trigger&gt;
        &lt;listOfEventAssignments&gt;
          <eventAssignment
variable="mw7d178aae_ff78_412a_830f_3be325d22773">
            &lt;math xmlns="http://www.w3.org/1998/Math/MathML"&gt;
              &lt;cn type="integer"&gt; 1 &lt;/cn&gt;
            &lt;/math&gt;
          &lt;/eventAssignment&gt;
        &lt;/listOfEventAssignments&gt;
      &lt;/event&gt;
      <event id="mw61c1cf72_cbfc_426e_9567_08232cdb1109"
name="event_603">
        &lt;trigger&gt;
          &lt;math xmlns="http://www.w3.org/1998/Math/MathML"&gt;
            &lt;apply&gt;
              &lt;geq/&gt;
              <csymbol encoding="text"
definitionURL="http://www.sbml.org/sbml/symbols/time"> time &lt;/csymbol&gt;
              &lt;cn&gt; 609.17 &lt;/cn&gt;
            &lt;/apply&gt;
```

```
          </math>
        </trigger>
        <listOfEventAssignments>
          <eventAssignment
variable="mw7d178aae_ff78_412a_830f_3be325d22773">
            <math xmlns="http://www.w3.org/1998/Math/MathML">
              <cn type="integer"> 0 </cn>
            </math>
          </eventAssignment>
        </listOfEventAssignments>
      </event>
      <event id="mwbb9cea47_4f2a_42a6_863c_5be56649f8e3"
name="event_604">
        <trigger>
          <math xmlns="http://www.w3.org/1998/Math/MathML">
            <apply>
              <geq/>
              <csymbol encoding="text"
definitionURL="http://www.sbml.org/sbml/symbols/time"> time </csymbol>
              <cn> 610.6 </cn>
            </apply>
          </math>
        </trigger>
        <listOfEventAssignments>
          <eventAssignment
variable="mw7d178aae_ff78_412a_830f_3be325d22773">
            <math xmlns="http://www.w3.org/1998/Math/MathML">
              <cn type="integer"> 1 </cn>
            </math>
          </eventAssignment>
        </listOfEventAssignments>
      </event>
      <event id="mwa0b4e2e0_1e99_4297_a198_4f3955d15b24"
name="event_605">
        <trigger>
          <math xmlns="http://www.w3.org/1998/Math/MathML">
            <apply>
              <geq/>
              <csymbol encoding="text"
definitionURL="http://www.sbml.org/sbml/symbols/time"> time </csymbol>
              <cn> 613.47 </cn>
            </apply>
          </math>
        </trigger>
        <listOfEventAssignments>
          <eventAssignment
variable="mw7d178aae_ff78_412a_830f_3be325d22773">
            <math xmlns="http://www.w3.org/1998/Math/MathML">
              <cn type="integer"> 0 </cn>
            </math>
          </eventAssignment>
        </listOfEventAssignments>
```

```
      &lt;/event&gt;
      <event id="mw9f406382_01ac_4aae_b7a9_61af7f61cacf"
name="event_606">
        &lt;trigger&gt;
          &lt;math xmlns="http://www.w3.org/1998/Math/MathML"&gt;
            &lt;apply&gt;
              &lt;geq/&gt;
              <csymbol encoding="text"
definitionURL="http://www.sbml.org/sbml/symbols/time"> time &lt;/csymbol&gt;
              &lt;cn&gt; 614.9 &lt;/cn&gt;
            &lt;/apply&gt;
          &lt;/math&gt;
        &lt;/trigger&gt;
        &lt;listOfEventAssignments&gt;
          <eventAssignment
variable="mw7d178aae_ff78_412a_830f_3be325d22773">
            &lt;math xmlns="http://www.w3.org/1998/Math/MathML"&gt;
              &lt;cn type="integer"&gt; 1 &lt;/cn&gt;
            &lt;/math&gt;
          &lt;/eventAssignment&gt;
        &lt;/listOfEventAssignments&gt;
      &lt;/event&gt;
      <event id="mw636639f9_ec92_4dff_b02b_4bb8c5e011de"
name="event_607">
        &lt;trigger&gt;
          &lt;math xmlns="http://www.w3.org/1998/Math/MathML"&gt;
            &lt;apply&gt;
              &lt;geq/&gt;
              <csymbol encoding="text"
definitionURL="http://www.sbml.org/sbml/symbols/time"> time &lt;/csymbol&gt;
              &lt;cn&gt; 617.77 &lt;/cn&gt;
            &lt;/apply&gt;
          &lt;/math&gt;
        &lt;/trigger&gt;
        &lt;listOfEventAssignments&gt;
          <eventAssignment
variable="mw7d178aae_ff78_412a_830f_3be325d22773">
            &lt;math xmlns="http://www.w3.org/1998/Math/MathML"&gt;
              &lt;cn type="integer"&gt; 0 &lt;/cn&gt;
            &lt;/math&gt;
          &lt;/eventAssignment&gt;
        &lt;/listOfEventAssignments&gt;
      &lt;/event&gt;
      <event id="mw83e1c6ad_060f_4e45_ac85_9f46251ae10e"
name="event_608">
        &lt;trigger&gt;
          &lt;math xmlns="http://www.w3.org/1998/Math/MathML"&gt;
            &lt;apply&gt;
              &lt;geq/&gt;
              <csymbol encoding="text"
definitionURL="http://www.sbml.org/sbml/symbols/time"> time &lt;/csymbol&gt;
              &lt;cn&gt; 619.2 &lt;/cn&gt;
```

```
          </apply>
        </math>
      </trigger>
      <listOfEventAssignments>
        <eventAssignment
variable="mw7d178aae_ff78_412a_830f_3be325d22773">
          <math xmlns="http://www.w3.org/1998/Math/MathML">
            <cn type="integer"> 1 </cn>
          </math>
        </eventAssignment>
      </listOfEventAssignments>
    </event>
    <event id="mwdfa3c163_c3ef_4a31_9d6b_eb41063b72b2"
name="event_609">
      <trigger>
        <math xmlns="http://www.w3.org/1998/Math/MathML">
          <apply>
            <geq/>
            <csymbol encoding="text"
definitionURL="http://www.sbml.org/sbml/symbols/time"> time </csymbol>
            <cn> 622.07 </cn>
          </apply>
        </math>
      </trigger>
      <listOfEventAssignments>
        <eventAssignment
variable="mw7d178aae_ff78_412a_830f_3be325d22773">
          <math xmlns="http://www.w3.org/1998/Math/MathML">
            <cn type="integer"> 0 </cn>
          </math>
        </eventAssignment>
      </listOfEventAssignments>
    </event>
    <event id="mw2c463421_e9df_4d8f_b2e1_9bc1f554cd74"
name="event_610">
      <trigger>
        <math xmlns="http://www.w3.org/1998/Math/MathML">
          <apply>
            <geq/>
            <csymbol encoding="text"
definitionURL="http://www.sbml.org/sbml/symbols/time"> time </csymbol>
            <cn> 623.5 </cn>
          </apply>
        </math>
      </trigger>
      <listOfEventAssignments>
        <eventAssignment
variable="mw7d178aae_ff78_412a_830f_3be325d22773">
          <math xmlns="http://www.w3.org/1998/Math/MathML">
            <cn type="integer"> 1 </cn>
          </math>
        </eventAssignment>
```

```
        </listOfEventAssignments>
      </event>
      <event id="mw63022dab_0ee3_4096_916e_5b0f930f0391"
name="event_611">
        <trigger>
          <math xmlns="http://www.w3.org/1998/Math/MathML">
            <apply>
              <geq/>
              <csymbol encoding="text"
definitionURL="http://www.sbml.org/sbml/symbols/time"> time </csymbol>
              <cn> 626.37 </cn>
            </apply>
          </math>
        </trigger>
        <listOfEventAssignments>
          <eventAssignment
variable="mw7d178aae_ff78_412a_830f_3be325d22773">
            <math xmlns="http://www.w3.org/1998/Math/MathML">
              <cn type="integer"> 0 </cn>
            </math>
          </eventAssignment>
        </listOfEventAssignments>
      </event>
      <event id="mw4c0ab82a_396c_4185_8ed8_86ddead9dfa4"
name="event_612">
        <trigger>
          <math xmlns="http://www.w3.org/1998/Math/MathML">
            <apply>
              <geq/>
              <csymbol encoding="text"
definitionURL="http://www.sbml.org/sbml/symbols/time"> time </csymbol>
              <cn> 627.8 </cn>
            </apply>
          </math>
        </trigger>
        <listOfEventAssignments>
          <eventAssignment
variable="mw7d178aae_ff78_412a_830f_3be325d22773">
            <math xmlns="http://www.w3.org/1998/Math/MathML">
              <cn type="integer"> 1 </cn>
            </math>
          </eventAssignment>
        </listOfEventAssignments>
      </event>
      <event id="mw51622fd2_ae70_4f6a_afc8_8161468d7235"
name="event_613">
        <trigger>
          <math xmlns="http://www.w3.org/1998/Math/MathML">
            <apply>
              <geq/>
              <csymbol encoding="text"
definitionURL="http://www.sbml.org/sbml/symbols/time"> time </csymbol>
```

```
            <cn> 630.67 </cn>
          </apply>
        </math>
      </trigger>
      <listOfEventAssignments>
        <eventAssignment
variable="mw7d178aae_ff78_412a_830f_3be325d22773">
          <math xmlns="http://www.w3.org/1998/Math/MathML">
            <cn type="integer"> 0 </cn>
          </math>
        </eventAssignment>
      </listOfEventAssignments>
    </event>
    <event id="mwa64e50a1_a143_49e6_965f_ee95b894a9c3"
name="event_614">
      <trigger>
        <math xmlns="http://www.w3.org/1998/Math/MathML">
          <apply>
            <geq/>
            <csymbol encoding="text"
definitionURL="http://www.sbml.org/sbml/symbols/time"> time </csymbol>
            <cn> 632.1 </cn>
          </apply>
        </math>
      </trigger>
      <listOfEventAssignments>
        <eventAssignment
variable="mw7d178aae_ff78_412a_830f_3be325d22773">
          <math xmlns="http://www.w3.org/1998/Math/MathML">
            <cn type="integer"> 1 </cn>
          </math>
        </eventAssignment>
      </listOfEventAssignments>
    </event>
    <event id="mw50ba0075_25dd_4502_ba55_0440924906a8"
name="event_615">
      <trigger>
        <math xmlns="http://www.w3.org/1998/Math/MathML">
          <apply>
            <geq/>
            <csymbol encoding="text"
definitionURL="http://www.sbml.org/sbml/symbols/time"> time </csymbol>
            <cn> 634.97 </cn>
          </apply>
        </math>
      </trigger>
      <listOfEventAssignments>
        <eventAssignment
variable="mw7d178aae_ff78_412a_830f_3be325d22773">
          <math xmlns="http://www.w3.org/1998/Math/MathML">
            <cn type="integer"> 0 </cn>
          </math>
```

```
          </eventAssignment>
        </listOfEventAssignments>
      </event>
      <event id="mwca8025f9_d063_4944_8f70_66b180e3c8f8"
name="event_616">
        <trigger>
          <math xmlns="http://www.w3.org/1998/Math/MathML">
            <apply>
              <geq/>
              <csymbol encoding="text"
definitionURL="http://www.sbml.org/sbml/symbols/time"> time </csymbol>
              <cn> 636.4 </cn>
            </apply>
          </math>
        </trigger>
        <listOfEventAssignments>
          <eventAssignment
variable="mw7d178aae_ff78_412a_830f_3be325d22773">
            <math xmlns="http://www.w3.org/1998/Math/MathML">
              <cn type="integer"> 1 </cn>
            </math>
          </eventAssignment>
        </listOfEventAssignments>
      </event>
      <event id="mw5f1c5038_791b_47bc_ad2b_3c972691eab0"
name="event_617">
        <trigger>
          <math xmlns="http://www.w3.org/1998/Math/MathML">
            <apply>
              <geq/>
              <csymbol encoding="text"
definitionURL="http://www.sbml.org/sbml/symbols/time"> time </csymbol>
              <cn> 639.27 </cn>
            </apply>
          </math>
        </trigger>
        <listOfEventAssignments>
          <eventAssignment
variable="mw7d178aae_ff78_412a_830f_3be325d22773">
            <math xmlns="http://www.w3.org/1998/Math/MathML">
              <cn type="integer"> 0 </cn>
            </math>
          </eventAssignment>
        </listOfEventAssignments>
      </event>
      <event id="mw1631a552_e74a_455c_955f_569f4666be52"
name="event_618">
        <trigger>
          <math xmlns="http://www.w3.org/1998/Math/MathML">
            <apply>
              <geq/>
```

```
              <csymbol encoding="text"
definitionURL="http://www.sbml.org/sbml/symbols/time"> time </csymbol>
              <cn> 640.7 </cn>
            </apply>
          </math>
        </trigger>
        <listOfEventAssignments>
          <eventAssignment
variable="mw7d178aae_ff78_412a_830f_3be325d22773">
            <math xmlns="http://www.w3.org/1998/Math/MathML">
              <cn type="integer"> 1 </cn>
            </math>
          </eventAssignment>
        </listOfEventAssignments>
      </event>
      <event id="mw9eb97cdb_c0fb_4abc_8ec4_2c1713edf475"
name="event_619">
        <trigger>
          <math xmlns="http://www.w3.org/1998/Math/MathML">
            <apply>
              <geq/>
              <csymbol encoding="text"
definitionURL="http://www.sbml.org/sbml/symbols/time"> time </csymbol>
              <cn> 643.57 </cn>
            </apply>
          </math>
        </trigger>
        <listOfEventAssignments>
          <eventAssignment
variable="mw7d178aae_ff78_412a_830f_3be325d22773">
            <math xmlns="http://www.w3.org/1998/Math/MathML">
              <cn type="integer"> 0 </cn>
            </math>
          </eventAssignment>
        </listOfEventAssignments>
      </event>
      <event id="mw97311332_592c_4de2_b6dc_2d3ee8480845"
name="event_620">
        <trigger>
          <math xmlns="http://www.w3.org/1998/Math/MathML">
            <apply>
              <geq/>
              <csymbol encoding="text"
definitionURL="http://www.sbml.org/sbml/symbols/time"> time </csymbol>
              <cn type="integer"> 645 </cn>
            </apply>
          </math>
        </trigger>
        <listOfEventAssignments>
          <eventAssignment
variable="mw7d178aae_ff78_412a_830f_3be325d22773">
            <math xmlns="http://www.w3.org/1998/Math/MathML">
```

```
              <cn type="integer"> 1 </cn>
            </math>
          </eventAssignment>
        </listOfEventAssignments>
      </event>
      <event id="mw534fab3f_e1df_4803_aacf_b7f4fd0d5a60"
name="event_621">
        <trigger>
          <math xmlns="http://www.w3.org/1998/Math/MathML">
            <apply>
              <geq/>
              <csymbol encoding="text"
definitionURL="http://www.sbml.org/sbml/symbols/time"> time </csymbol>
              <cn> 647.87 </cn>
            </apply>
          </math>
        </trigger>
        <listOfEventAssignments>
          <eventAssignment
variable="mw7d178aae_ff78_412a_830f_3be325d22773">
            <math xmlns="http://www.w3.org/1998/Math/MathML">
              <cn type="integer"> 0 </cn>
            </math>
          </eventAssignment>
        </listOfEventAssignments>
      </event>
      <event id="mw5c51cbc6_2cad_4409_a626_c23afe6292b7"
name="event_622">
        <trigger>
          <math xmlns="http://www.w3.org/1998/Math/MathML">
            <apply>
              <geq/>
              <csymbol encoding="text"
definitionURL="http://www.sbml.org/sbml/symbols/time"> time </csymbol>
              <cn> 649.3 </cn>
            </apply>
          </math>
        </trigger>
        <listOfEventAssignments>
          <eventAssignment
variable="mw7d178aae_ff78_412a_830f_3be325d22773">
            <math xmlns="http://www.w3.org/1998/Math/MathML">
              <cn type="integer"> 1 </cn>
            </math>
          </eventAssignment>
        </listOfEventAssignments>
      </event>
      <event id="mw91ebbb27_90eb_4ec1_ace9_7aef31087d11"
name="event_623">
        <trigger>
          <math xmlns="http://www.w3.org/1998/Math/MathML">
            <apply>
```

```
              <geq/>
              <csymbol encoding="text"
definitionURL="http://www.sbml.org/sbml/symbols/time"> time </csymbol>
              <cn> 652.17 </cn>
            </apply>
          </math>
        </trigger>
        <listOfEventAssignments>
          <eventAssignment
variable="mw7d178aae_ff78_412a_830f_3be325d22773">
            <math xmlns="http://www.w3.org/1998/Math/MathML">
              <cn type="integer"> 0 </cn>
            </math>
          </eventAssignment>
        </listOfEventAssignments>
      </event>
      <event id="mw1c81b862_01df_4203_a146_e368d1ff4dc7"
name="event_624">
        <trigger>
          <math xmlns="http://www.w3.org/1998/Math/MathML">
            <apply>
              <geq/>
              <csymbol encoding="text"
definitionURL="http://www.sbml.org/sbml/symbols/time"> time </csymbol>
              <cn> 653.6 </cn>
            </apply>
          </math>
        </trigger>
        <listOfEventAssignments>
          <eventAssignment
variable="mw7d178aae_ff78_412a_830f_3be325d22773">
            <math xmlns="http://www.w3.org/1998/Math/MathML">
              <cn type="integer"> 1 </cn>
            </math>
          </eventAssignment>
        </listOfEventAssignments>
      </event>
      <event id="mwde8ab415_9807_402c_93e0_48188cedee55"
name="event_625">
        <trigger>
          <math xmlns="http://www.w3.org/1998/Math/MathML">
            <apply>
              <geq/>
              <csymbol encoding="text"
definitionURL="http://www.sbml.org/sbml/symbols/time"> time </csymbol>
              <cn> 656.47 </cn>
            </apply>
          </math>
        </trigger>
        <listOfEventAssignments>
          <eventAssignment
variable="mw7d178aae_ff78_412a_830f_3be325d22773">
```

```
            <math xmlns="http://www.w3.org/1998/Math/MathML">
              <cn type="integer"> 0 </cn>
            </math>
          </eventAssignment>
        </listOfEventAssignments>
      </event>
      <event id="mw1e88d4cd_31a1_4862_b6f9_9a594b522c06"
name="event_626">
        <trigger>
          <math xmlns="http://www.w3.org/1998/Math/MathML">
            <apply>
              <geq/>
              <csymbol encoding="text"
definitionURL="http://www.sbml.org/sbml/symbols/time"> time </csymbol>
              <cn> 657.9 </cn>
            </apply>
          </math>
        </trigger>
        <listOfEventAssignments>
          <eventAssignment
variable="mw7d178aae_ff78_412a_830f_3be325d22773">
            <math xmlns="http://www.w3.org/1998/Math/MathML">
              <cn type="integer"> 1 </cn>
            </math>
          </eventAssignment>
        </listOfEventAssignments>
      </event>
      <event id="mw4b312ef9_95e9_43ba_afff_eae2c4dd9539"
name="event_627">
        <trigger>
          <math xmlns="http://www.w3.org/1998/Math/MathML">
            <apply>
              <geq/>
              <csymbol encoding="text"
definitionURL="http://www.sbml.org/sbml/symbols/time"> time </csymbol>
              <cn> 660.77 </cn>
            </apply>
          </math>
        </trigger>
        <listOfEventAssignments>
          <eventAssignment
variable="mw7d178aae_ff78_412a_830f_3be325d22773">
            <math xmlns="http://www.w3.org/1998/Math/MathML">
              <cn type="integer"> 0 </cn>
            </math>
          </eventAssignment>
        </listOfEventAssignments>
      </event>
      <event id="mwdb12357e_bd2d_4775_ad57_0b43a419ac1c"
name="event_628">
        <trigger>
          <math xmlns="http://www.w3.org/1998/Math/MathML">
```

```
            <apply>
              <geq/>
              <csymbol encoding="text"
definitionURL="http://www.sbml.org/sbml/symbols/time"> time </csymbol>
              <cn> 662.2 </cn>
            </apply>
          </math>
        </trigger>
        <listOfEventAssignments>
          <eventAssignment
variable="mw7d178aae_ff78_412a_830f_3be325d22773">
            <math xmlns="http://www.w3.org/1998/Math/MathML">
              <cn type="integer"> 1 </cn>
            </math>
          </eventAssignment>
        </listOfEventAssignments>
      </event>
      <event id="mw8e0b6f6b_c8ef_4c8b_8d75_76f407e03800"
name="event_629">
        <trigger>
          <math xmlns="http://www.w3.org/1998/Math/MathML">
            <apply>
              <geq/>
              <csymbol encoding="text"
definitionURL="http://www.sbml.org/sbml/symbols/time"> time </csymbol>
              <cn> 665.07 </cn>
            </apply>
          </math>
        </trigger>
        <listOfEventAssignments>
          <eventAssignment
variable="mw7d178aae_ff78_412a_830f_3be325d22773">
            <math xmlns="http://www.w3.org/1998/Math/MathML">
              <cn type="integer"> 0 </cn>
            </math>
          </eventAssignment>
        </listOfEventAssignments>
      </event>
      <event id="mwae679f21_c53b_413e_94c6_7f5d5f29bb2d"
name="event_630">
        <trigger>
          <math xmlns="http://www.w3.org/1998/Math/MathML">
            <apply>
              <geq/>
              <csymbol encoding="text"
definitionURL="http://www.sbml.org/sbml/symbols/time"> time </csymbol>
              <cn> 666.5 </cn>
            </apply>
          </math>
        </trigger>
        <listOfEventAssignments>
```

```
          <eventAssignment
variable="mw7d178aae_ff78_412a_830f_3be325d22773">
            <math xmlns="http://www.w3.org/1998/Math/MathML">
              <cn type="integer"> 1 </cn>
            </math>
          </eventAssignment>
        </listOfEventAssignments>
      </event>
      <event id="mw87f02197_ecd5_460b_899f_ef5adbffdbc1"
name="event_631">
        <trigger>
          <math xmlns="http://www.w3.org/1998/Math/MathML">
            <apply>
              <geq/>
              <csymbol encoding="text"
definitionURL="http://www.sbml.org/sbml/symbols/time"> time </csymbol>
              <cn> 669.37 </cn>
            </apply>
          </math>
        </trigger>
        <listOfEventAssignments>
          <eventAssignment
variable="mw7d178aae_ff78_412a_830f_3be325d22773">
            <math xmlns="http://www.w3.org/1998/Math/MathML">
              <cn type="integer"> 0 </cn>
            </math>
          </eventAssignment>
        </listOfEventAssignments>
      </event>
      <event id="mw50130d15_62da_4bca_94f3_adc4709a26ae"
name="event_632">
        <trigger>
          <math xmlns="http://www.w3.org/1998/Math/MathML">
            <apply>
              <geq/>
              <csymbol encoding="text"
definitionURL="http://www.sbml.org/sbml/symbols/time"> time </csymbol>
              <cn> 670.8 </cn>
            </apply>
          </math>
        </trigger>
        <listOfEventAssignments>
          <eventAssignment
variable="mw7d178aae_ff78_412a_830f_3be325d22773">
            <math xmlns="http://www.w3.org/1998/Math/MathML">
              <cn type="integer"> 1 </cn>
            </math>
          </eventAssignment>
        </listOfEventAssignments>
      </event>
      <event id="mwc843b820_0994_4142_86be_1b18fecda250"
name="event_633">
```

```
        &lt;trigger&gt;
          &lt;math xmlns="http://www.w3.org/1998/Math/MathML"&gt;
            &lt;apply&gt;
              &lt;geq/&gt;
              <csymbol encoding="text"
definitionURL="http://www.sbml.org/sbml/symbols/time"> time &lt;/csymbol&gt;
              &lt;cn&gt; 673.67 &lt;/cn&gt;
            &lt;/apply&gt;
          &lt;/math&gt;
        &lt;/trigger&gt;
        &lt;listOfEventAssignments&gt;
          <eventAssignment
variable="mw7d178aae_ff78_412a_830f_3be325d22773">
            &lt;math xmlns="http://www.w3.org/1998/Math/MathML"&gt;
              &lt;cn type="integer"&gt; 0 &lt;/cn&gt;
            &lt;/math&gt;
          &lt;/eventAssignment&gt;
        &lt;/listOfEventAssignments&gt;
      &lt;/event&gt;
      <event id="mwb44d3d46_095f_4d69_b04a_d9a85f85ad4e"
name="event_634">
        &lt;trigger&gt;
          &lt;math xmlns="http://www.w3.org/1998/Math/MathML"&gt;
            &lt;apply&gt;
              &lt;geq/&gt;
              <csymbol encoding="text"
definitionURL="http://www.sbml.org/sbml/symbols/time"> time &lt;/csymbol&gt;
              &lt;cn&gt; 675.1 &lt;/cn&gt;
            &lt;/apply&gt;
          &lt;/math&gt;
        &lt;/trigger&gt;
        &lt;listOfEventAssignments&gt;
          <eventAssignment
variable="mw7d178aae_ff78_412a_830f_3be325d22773">
            &lt;math xmlns="http://www.w3.org/1998/Math/MathML"&gt;
              &lt;cn type="integer"&gt; 1 &lt;/cn&gt;
            &lt;/math&gt;
          &lt;/eventAssignment&gt;
        &lt;/listOfEventAssignments&gt;
      &lt;/event&gt;
      <event id="mw115d7a76_14c0_4ada_9a41_14745437d1e3"
name="event_635">
        &lt;trigger&gt;
          &lt;math xmlns="http://www.w3.org/1998/Math/MathML"&gt;
            &lt;apply&gt;
              &lt;geq/&gt;
              <csymbol encoding="text"
definitionURL="http://www.sbml.org/sbml/symbols/time"> time &lt;/csymbol&gt;
              &lt;cn&gt; 677.97 &lt;/cn&gt;
            &lt;/apply&gt;
          &lt;/math&gt;
        &lt;/trigger&gt;
```

```
        &lt;listOfEventAssignments&gt;
          <eventAssignment
variable="mw7d178aae_ff78_412a_830f_3be325d22773">
            &lt;math xmlns="http://www.w3.org/1998/Math/MathML"&gt;
              &lt;cn type="integer"&gt; 0 &lt;/cn&gt;
            &lt;/math&gt;
          &lt;/eventAssignment&gt;
        &lt;/listOfEventAssignments&gt;
      &lt;/event&gt;
      <event id="mw6c7586ed_0941_4392_8c07_5a603c7b1dfa"
name="event_636">
        &lt;trigger&gt;
          &lt;math xmlns="http://www.w3.org/1998/Math/MathML"&gt;
            &lt;apply&gt;
              &lt;geq/&gt;
              <csymbol encoding="text"
definitionURL="http://www.sbml.org/sbml/symbols/time"> time &lt;/csymbol&gt;
              &lt;cn&gt; 679.4 &lt;/cn&gt;
            &lt;/apply&gt;
          &lt;/math&gt;
        &lt;/trigger&gt;
        &lt;listOfEventAssignments&gt;
          <eventAssignment
variable="mw7d178aae_ff78_412a_830f_3be325d22773">
            &lt;math xmlns="http://www.w3.org/1998/Math/MathML"&gt;
              &lt;cn type="integer"&gt; 1 &lt;/cn&gt;
            &lt;/math&gt;
          &lt;/eventAssignment&gt;
        &lt;/listOfEventAssignments&gt;
      &lt;/event&gt;
      <event id="mw7c523b41_08ed_4310_957a_b36181add757"
name="event_637">
        &lt;trigger&gt;
          &lt;math xmlns="http://www.w3.org/1998/Math/MathML"&gt;
            &lt;apply&gt;
              &lt;geq/&gt;
              <csymbol encoding="text"
definitionURL="http://www.sbml.org/sbml/symbols/time"> time &lt;/csymbol&gt;
              &lt;cn&gt; 682.27 &lt;/cn&gt;
            &lt;/apply&gt;
          &lt;/math&gt;
        &lt;/trigger&gt;
        &lt;listOfEventAssignments&gt;
          <eventAssignment
variable="mw7d178aae_ff78_412a_830f_3be325d22773">
            &lt;math xmlns="http://www.w3.org/1998/Math/MathML"&gt;
              &lt;cn type="integer"&gt; 0 &lt;/cn&gt;
            &lt;/math&gt;
          &lt;/eventAssignment&gt;
        &lt;/listOfEventAssignments&gt;
      &lt;/event&gt;
```

```
      <event id="mwcc2fac97_caa8_4bca_b5c2_8885e78c2848"
name="event_638">
        <trigger>
          <math xmlns="http://www.w3.org/1998/Math/MathML">
            <apply>
              <geq/>
              <csymbol encoding="text"
definitionURL="http://www.sbml.org/sbml/symbols/time"> time </csymbol>
              <cn> 683.7 </cn>
            </apply>
          </math>
        </trigger>
        <listOfEventAssignments>
          <eventAssignment
variable="mw7d178aae_ff78_412a_830f_3be325d22773">
            <math xmlns="http://www.w3.org/1998/Math/MathML">
              <cn type="integer"> 1 </cn>
            </math>
          </eventAssignment>
        </listOfEventAssignments>
      </event>
      <event id="mw00987ccb_638e_4f52_995d_b85cc3feeed7"
name="event_639">
        <trigger>
          <math xmlns="http://www.w3.org/1998/Math/MathML">
            <apply>
              <geq/>
              <csymbol encoding="text"
definitionURL="http://www.sbml.org/sbml/symbols/time"> time </csymbol>
              <cn> 686.57 </cn>
            </apply>
          </math>
        </trigger>
        <listOfEventAssignments>
          <eventAssignment
variable="mw7d178aae_ff78_412a_830f_3be325d22773">
            <math xmlns="http://www.w3.org/1998/Math/MathML">
              <cn type="integer"> 0 </cn>
            </math>
          </eventAssignment>
        </listOfEventAssignments>
      </event>
      <event id="mw3852cf18_4cad_4377_9baa_5abb3dee391a"
name="event_640">
        <trigger>
          <math xmlns="http://www.w3.org/1998/Math/MathML">
            <apply>
              <geq/>
              <csymbol encoding="text"
definitionURL="http://www.sbml.org/sbml/symbols/time"> time </csymbol>
              <cn type="integer"> 688 </cn>
            </apply>
```

```
          </math>
        </trigger>
        <listOfEventAssignments>
          <eventAssignment
variable="mw7d178aae_ff78_412a_830f_3be325d22773">
            <math xmlns="http://www.w3.org/1998/Math/MathML">
              <cn type="integer"> 1 </cn>
            </math>
          </eventAssignment>
        </listOfEventAssignments>
      </event>
      <event id="mwced55e68_d51f_4b61_aee3_cf63d9cf9de4"
name="event_641">
        <trigger>
          <math xmlns="http://www.w3.org/1998/Math/MathML">
            <apply>
              <geq/>
              <csymbol encoding="text"
definitionURL="http://www.sbml.org/sbml/symbols/time"> time </csymbol>
              <cn> 690.87 </cn>
            </apply>
          </math>
        </trigger>
        <listOfEventAssignments>
          <eventAssignment
variable="mwcedbe49e_2d28_4720_8fcd_207db64228cf">
            <math xmlns="http://www.w3.org/1998/Math/MathML">
              <cn type="integer"> 1 </cn>
            </math>
          </eventAssignment>
        </listOfEventAssignments>
      </event>
      <event id="mw8d99183c_af97_4ce9_93f4_c539b0da25d8"
name="event_642">
        <trigger>
          <math xmlns="http://www.w3.org/1998/Math/MathML">
            <apply>
              <geq/>
              <csymbol encoding="text"
definitionURL="http://www.sbml.org/sbml/symbols/time"> time </csymbol>
              <cn> 692.3 </cn>
            </apply>
          </math>
        </trigger>
        <listOfEventAssignments>
          <eventAssignment
variable="mwcedbe49e_2d28_4720_8fcd_207db64228cf">
            <math xmlns="http://www.w3.org/1998/Math/MathML">
              <cn type="integer"> 0 </cn>
            </math>
          </eventAssignment>
        </listOfEventAssignments>
```

```
      &lt;/event&gt;
      <event id="mw94c1ea22_b7ca_494f_8f3a_021f6ae81daf"
name="event_643">
        &lt;trigger&gt;
          &lt;math xmlns="http://www.w3.org/1998/Math/MathML"&gt;
            &lt;apply&gt;
              &lt;geq/&gt;
              <csymbol encoding="text"
definitionURL="http://www.sbml.org/sbml/symbols/time"> time &lt;/csymbol&gt;
              &lt;cn&gt; 695.17 &lt;/cn&gt;
            &lt;/apply&gt;
          &lt;/math&gt;
        &lt;/trigger&gt;
        &lt;listOfEventAssignments&gt;
          <eventAssignment
variable="mwcedbe49e_2d28_4720_8fcd_207db64228cf">
            &lt;math xmlns="http://www.w3.org/1998/Math/MathML"&gt;
              &lt;cn type="integer"&gt; 1 &lt;/cn&gt;
            &lt;/math&gt;
          &lt;/eventAssignment&gt;
        &lt;/listOfEventAssignments&gt;
      &lt;/event&gt;
      <event id="mw9ba76ca1_7b7d_4841_aa1d_15cc9020752c"
name="event_644">
        &lt;trigger&gt;
          &lt;math xmlns="http://www.w3.org/1998/Math/MathML"&gt;
            &lt;apply&gt;
              &lt;geq/&gt;
              <csymbol encoding="text"
definitionURL="http://www.sbml.org/sbml/symbols/time"> time &lt;/csymbol&gt;
              &lt;cn&gt; 696.6 &lt;/cn&gt;
            &lt;/apply&gt;
          &lt;/math&gt;
        &lt;/trigger&gt;
        &lt;listOfEventAssignments&gt;
          <eventAssignment
variable="mwcedbe49e_2d28_4720_8fcd_207db64228cf">
            &lt;math xmlns="http://www.w3.org/1998/Math/MathML"&gt;
              &lt;cn type="integer"&gt; 0 &lt;/cn&gt;
            &lt;/math&gt;
          &lt;/eventAssignment&gt;
        &lt;/listOfEventAssignments&gt;
      &lt;/event&gt;
      <event id="mw614a0f56_faec_4482_8838_14572a623489"
name="event_645">
        &lt;trigger&gt;
          &lt;math xmlns="http://www.w3.org/1998/Math/MathML"&gt;
            &lt;apply&gt;
              &lt;geq/&gt;
              <csymbol encoding="text"
definitionURL="http://www.sbml.org/sbml/symbols/time"> time &lt;/csymbol&gt;
              &lt;cn&gt; 699.47 &lt;/cn&gt;
```

```
            </apply>
          </math>
        </trigger>
        <listOfEventAssignments>
          <eventAssignment
variable="mwcedbe49e_2d28_4720_8fcd_207db64228cf">
            <math xmlns="http://www.w3.org/1998/Math/MathML">
              <cn type="integer"> 1 </cn>
            </math>
          </eventAssignment>
        </listOfEventAssignments>
      </event>
      <event id="mw7a6e4de1_0aaf_4061_b33e_29d9f0ae6075"
name="event_646">
        <trigger>
          <math xmlns="http://www.w3.org/1998/Math/MathML">
            <apply>
              <geq/>
              <csymbol encoding="text"
definitionURL="http://www.sbml.org/sbml/symbols/time"> time </csymbol>
              <cn> 700.9 </cn>
            </apply>
          </math>
        </trigger>
        <listOfEventAssignments>
          <eventAssignment
variable="mwcedbe49e_2d28_4720_8fcd_207db64228cf">
            <math xmlns="http://www.w3.org/1998/Math/MathML">
              <cn type="integer"> 0 </cn>
            </math>
          </eventAssignment>
        </listOfEventAssignments>
      </event>
      <event id="mwc5087fe6_11c7_47b9_9d32_959a41fbd523"
name="event_647">
        <trigger>
          <math xmlns="http://www.w3.org/1998/Math/MathML">
            <apply>
              <geq/>
              <csymbol encoding="text"
definitionURL="http://www.sbml.org/sbml/symbols/time"> time </csymbol>
              <cn> 703.77 </cn>
            </apply>
          </math>
        </trigger>
        <listOfEventAssignments>
          <eventAssignment
variable="mwcedbe49e_2d28_4720_8fcd_207db64228cf">
            <math xmlns="http://www.w3.org/1998/Math/MathML">
              <cn type="integer"> 1 </cn>
            </math>
          </eventAssignment>
```

```
        </listOfEventAssignments>
      </event>
      <event id="mw1351257b_9de8_4c5d_99dd_3028a421a5c8"
name="event_648">
        <trigger>
          <math xmlns="http://www.w3.org/1998/Math/MathML">
            <apply>
              <geq/>
              <csymbol encoding="text"
definitionURL="http://www.sbml.org/sbml/symbols/time"> time </csymbol>
              <cn> 705.2 </cn>
            </apply>
          </math>
        </trigger>
        <listOfEventAssignments>
          <eventAssignment
variable="mwcedbe49e_2d28_4720_8fcd_207db64228cf">
            <math xmlns="http://www.w3.org/1998/Math/MathML">
              <cn type="integer"> 0 </cn>
            </math>
          </eventAssignment>
        </listOfEventAssignments>
      </event>
      <event id="mw01a5f621_b1e4_44e7_8fb6_8f4c9cfbba89"
name="event_649">
        <trigger>
          <math xmlns="http://www.w3.org/1998/Math/MathML">
            <apply>
              <geq/>
              <csymbol encoding="text"
definitionURL="http://www.sbml.org/sbml/symbols/time"> time </csymbol>
              <cn> 708.07 </cn>
            </apply>
          </math>
        </trigger>
        <listOfEventAssignments>
          <eventAssignment
variable="mwcedbe49e_2d28_4720_8fcd_207db64228cf">
            <math xmlns="http://www.w3.org/1998/Math/MathML">
              <cn type="integer"> 1 </cn>
            </math>
          </eventAssignment>
        </listOfEventAssignments>
      </event>
      <event id="mw5c16237f_0b37_4cdd_a1bf_c7995e48e25c"
name="event_650">
        <trigger>
          <math xmlns="http://www.w3.org/1998/Math/MathML">
            <apply>
              <geq/>
              <csymbol encoding="text"
definitionURL="http://www.sbml.org/sbml/symbols/time"> time </csymbol>
```

```
            &lt;cn&gt; 709.5 &lt;/cn&gt;
          &lt;/apply&gt;
        &lt;/math&gt;
      &lt;/trigger&gt;
      &lt;listOfEventAssignments&gt;
        <eventAssignment
variable="mwcedbe49e_2d28_4720_8fcd_207db64228cf">
          &lt;math xmlns="http://www.w3.org/1998/Math/MathML"&gt;
            &lt;cn type="integer"&gt; 0 &lt;/cn&gt;
          &lt;/math&gt;
        &lt;/eventAssignment&gt;
      &lt;/listOfEventAssignments&gt;
    &lt;/event&gt;
    <event id="mw20e4589c_74da_44d9_ae12_42f002e25126"
name="event_655">
      &lt;trigger&gt;
        &lt;math xmlns="http://www.w3.org/1998/Math/MathML"&gt;
          &lt;apply&gt;
            &lt;geq/&gt;
            <csymbol encoding="text"
definitionURL="http://www.sbml.org/sbml/symbols/time"> time &lt;/csymbol&gt;
            &lt;cn&gt; 712.37 &lt;/cn&gt;
          &lt;/apply&gt;
        &lt;/math&gt;
      &lt;/trigger&gt;
      &lt;listOfEventAssignments&gt;
        <eventAssignment
variable="mwcedbe49e_2d28_4720_8fcd_207db64228cf">
          &lt;math xmlns="http://www.w3.org/1998/Math/MathML"&gt;
            &lt;cn type="integer"&gt; 1 &lt;/cn&gt;
          &lt;/math&gt;
        &lt;/eventAssignment&gt;
      &lt;/listOfEventAssignments&gt;
    &lt;/event&gt;
    <event id="mw3dda5102_b788_4a67_802a_b81b8a172609"
name="event_656">
      &lt;trigger&gt;
        &lt;math xmlns="http://www.w3.org/1998/Math/MathML"&gt;
          &lt;apply&gt;
            &lt;geq/&gt;
            <csymbol encoding="text"
definitionURL="http://www.sbml.org/sbml/symbols/time"> time &lt;/csymbol&gt;
            &lt;cn&gt; 713.8 &lt;/cn&gt;
          &lt;/apply&gt;
        &lt;/math&gt;
      &lt;/trigger&gt;
      &lt;listOfEventAssignments&gt;
        <eventAssignment
variable="mwcedbe49e_2d28_4720_8fcd_207db64228cf">
          &lt;math xmlns="http://www.w3.org/1998/Math/MathML"&gt;
            &lt;cn type="integer"&gt; 0 &lt;/cn&gt;
          &lt;/math&gt;
```

```
          </eventAssignment>
        </listOfEventAssignments>
      </event>
      <event id="mwed2272eb_ee59_403d_a765_eb1485e0ed78"
name="event_657">
        <trigger>
          <math xmlns="http://www.w3.org/1998/Math/MathML">
            <apply>
              <geq/>
              <csymbol encoding="text"
definitionURL="http://www.sbml.org/sbml/symbols/time"> time </csymbol>
              <cn> 716.67 </cn>
            </apply>
          </math>
        </trigger>
        <listOfEventAssignments>
          <eventAssignment
variable="mwcedbe49e_2d28_4720_8fcd_207db64228cf">
            <math xmlns="http://www.w3.org/1998/Math/MathML">
              <cn type="integer"> 1 </cn>
            </math>
          </eventAssignment>
        </listOfEventAssignments>
      </event>
      <event id="mw9224412c_9c23_4dec_b64b_73532eb448e7"
name="event_658">
        <trigger>
          <math xmlns="http://www.w3.org/1998/Math/MathML">
            <apply>
              <geq/>
              <csymbol encoding="text"
definitionURL="http://www.sbml.org/sbml/symbols/time"> time </csymbol>
              <cn> 718.1 </cn>
            </apply>
          </math>
        </trigger>
        <listOfEventAssignments>
          <eventAssignment
variable="mwcedbe49e_2d28_4720_8fcd_207db64228cf">
            <math xmlns="http://www.w3.org/1998/Math/MathML">
              <cn type="integer"> 0 </cn>
            </math>
          </eventAssignment>
        </listOfEventAssignments>
      </event>
      <event id="mwa0c1c630_8fd3_4a67_837d_a76e9379bced"
name="event_659">
        <trigger>
          <math xmlns="http://www.w3.org/1998/Math/MathML">
            <apply>
              <geq/>
```

```
              <csymbol encoding="text"
definitionURL="http://www.sbml.org/sbml/symbols/time"> time </csymbol>
              <cn> 720.97 </cn>
            </apply>
          </math>
        </trigger>
        <listOfEventAssignments>
          <eventAssignment
variable="mwcedbe49e_2d28_4720_8fcd_207db64228cf">
            <math xmlns="http://www.w3.org/1998/Math/MathML">
              <cn type="integer"> 1 </cn>
            </math>
          </eventAssignment>
        </listOfEventAssignments>
      </event>
      <event id="mwb2b13bbb_963e_4c12_ab93_f9fce9342435"
name="event_660">
        <trigger>
          <math xmlns="http://www.w3.org/1998/Math/MathML">
            <apply>
              <geq/>
              <csymbol encoding="text"
definitionURL="http://www.sbml.org/sbml/symbols/time"> time </csymbol>
              <cn> 722.4 </cn>
            </apply>
          </math>
        </trigger>
        <listOfEventAssignments>
          <eventAssignment
variable="mwcedbe49e_2d28_4720_8fcd_207db64228cf">
            <math xmlns="http://www.w3.org/1998/Math/MathML">
              <cn type="integer"> 0 </cn>
            </math>
          </eventAssignment>
        </listOfEventAssignments>
      </event>
      <event id="mwe158810e_1601_4f07_acaa_0750592f2af9"
name="event_661">
        <trigger>
          <math xmlns="http://www.w3.org/1998/Math/MathML">
            <apply>
              <geq/>
              <csymbol encoding="text"
definitionURL="http://www.sbml.org/sbml/symbols/time"> time </csymbol>
              <cn> 725.27 </cn>
            </apply>
          </math>
        </trigger>
        <listOfEventAssignments>
          <eventAssignment
variable="mwcedbe49e_2d28_4720_8fcd_207db64228cf">
            <math xmlns="http://www.w3.org/1998/Math/MathML">
```

```
            &lt;cn type="integer"&gt; 1 &lt;/cn&gt;
          &lt;/math&gt;
        &lt;/eventAssignment&gt;
      &lt;/listOfEventAssignments&gt;
    &lt;/event&gt;
    <event id="mwbf18075e_1a9c_477b_a99d_6be83fa74bce"
name="event_662">
      &lt;trigger&gt;
        &lt;math xmlns="http://www.w3.org/1998/Math/MathML"&gt;
          &lt;apply&gt;
            &lt;geq/&gt;
            <csymbol encoding="text"
definitionURL="http://www.sbml.org/sbml/symbols/time"> time &lt;/csymbol&gt;
            &lt;cn&gt; 726.7 &lt;/cn&gt;
          &lt;/apply&gt;
        &lt;/math&gt;
      &lt;/trigger&gt;
      &lt;listOfEventAssignments&gt;
        <eventAssignment
variable="mwcedbe49e_2d28_4720_8fcd_207db64228cf">
          &lt;math xmlns="http://www.w3.org/1998/Math/MathML"&gt;
            &lt;cn type="integer"&gt; 0 &lt;/cn&gt;
          &lt;/math&gt;
        &lt;/eventAssignment&gt;
      &lt;/listOfEventAssignments&gt;
    &lt;/event&gt;
    <event id="mw831fe706_fdfd_4454_b62e_7c1de3fb6d9f"
name="event_663">
      &lt;trigger&gt;
        &lt;math xmlns="http://www.w3.org/1998/Math/MathML"&gt;
          &lt;apply&gt;
            &lt;geq/&gt;
            <csymbol encoding="text"
definitionURL="http://www.sbml.org/sbml/symbols/time"> time &lt;/csymbol&gt;
            &lt;cn&gt; 729.57 &lt;/cn&gt;
          &lt;/apply&gt;
        &lt;/math&gt;
      &lt;/trigger&gt;
      &lt;listOfEventAssignments&gt;
        <eventAssignment
variable="mwcedbe49e_2d28_4720_8fcd_207db64228cf">
          &lt;math xmlns="http://www.w3.org/1998/Math/MathML"&gt;
            &lt;cn type="integer"&gt; 1 &lt;/cn&gt;
          &lt;/math&gt;
        &lt;/eventAssignment&gt;
      &lt;/listOfEventAssignments&gt;
    &lt;/event&gt;
    <event id="mw49032030_782f_459f_b540_38ef17e2563e"
name="event_664">
      &lt;trigger&gt;
        &lt;math xmlns="http://www.w3.org/1998/Math/MathML"&gt;
          &lt;apply&gt;
```

```
            <geq/>
            <csymbol encoding="text"
definitionURL="http://www.sbml.org/sbml/symbols/time"> time </csymbol>
            <cn type="integer"> 731 </cn>
          </apply>
        </math>
      </trigger>
      <listOfEventAssignments>
        <eventAssignment
variable="mwcedbe49e_2d28_4720_8fcd_207db64228cf">
          <math xmlns="http://www.w3.org/1998/Math/MathML">
            <cn type="integer"> 0 </cn>
          </math>
        </eventAssignment>
      </listOfEventAssignments>
    </event>
    <event id="mwba917d28_34bc_4b53_8464_1cdf3599f8c5"
name="event_665">
      <trigger>
        <math xmlns="http://www.w3.org/1998/Math/MathML">
          <apply>
            <geq/>
            <csymbol encoding="text"
definitionURL="http://www.sbml.org/sbml/symbols/time"> time </csymbol>
            <cn> 733.87 </cn>
          </apply>
        </math>
      </trigger>
      <listOfEventAssignments>
        <eventAssignment
variable="mwcedbe49e_2d28_4720_8fcd_207db64228cf">
          <math xmlns="http://www.w3.org/1998/Math/MathML">
            <cn type="integer"> 1 </cn>
          </math>
        </eventAssignment>
      </listOfEventAssignments>
    </event>
    <event id="mwc5bf5a04_6b90_4d01_8fbc_5039f7b2e18c"
name="event_666">
      <trigger>
        <math xmlns="http://www.w3.org/1998/Math/MathML">
          <apply>
            <geq/>
            <csymbol encoding="text"
definitionURL="http://www.sbml.org/sbml/symbols/time"> time </csymbol>
            <cn> 735.3 </cn>
          </apply>
        </math>
      </trigger>
      <listOfEventAssignments>
        <eventAssignment
variable="mwcedbe49e_2d28_4720_8fcd_207db64228cf">
```

```
            <math xmlns="http://www.w3.org/1998/Math/MathML">
              <cn type="integer"> 0 </cn>
            </math>
          </eventAssignment>
        </listOfEventAssignments>
      </event>
      <event id="mw13412a01_cbc8_4546_9d12_5f88c7451533"
name="event_667">
        <trigger>
          <math xmlns="http://www.w3.org/1998/Math/MathML">
            <apply>
              <geq/>
              <csymbol encoding="text"
definitionURL="http://www.sbml.org/sbml/symbols/time"> time </csymbol>
              <cn> 738.17 </cn>
            </apply>
          </math>
        </trigger>
        <listOfEventAssignments>
          <eventAssignment
variable="mwcedbe49e_2d28_4720_8fcd_207db64228cf">
            <math xmlns="http://www.w3.org/1998/Math/MathML">
              <cn type="integer"> 1 </cn>
            </math>
          </eventAssignment>
        </listOfEventAssignments>
      </event>
      <event id="mw9cf5a4e6_0004_493a_b5c8_7a72f1fe48a6"
name="event_668">
        <trigger>
          <math xmlns="http://www.w3.org/1998/Math/MathML">
            <apply>
              <geq/>
              <csymbol encoding="text"
definitionURL="http://www.sbml.org/sbml/symbols/time"> time </csymbol>
              <cn> 739.6 </cn>
            </apply>
          </math>
        </trigger>
        <listOfEventAssignments>
          <eventAssignment
variable="mwcedbe49e_2d28_4720_8fcd_207db64228cf">
            <math xmlns="http://www.w3.org/1998/Math/MathML">
              <cn type="integer"> 0 </cn>
            </math>
          </eventAssignment>
        </listOfEventAssignments>
      </event>
      <event id="mw8f6d028d_06fb_48d2_881b_42d815bc12dd"
name="event_669">
        <trigger>
          <math xmlns="http://www.w3.org/1998/Math/MathML">
```

```
            &lt;apply&gt;
              &lt;geq/&gt;
              <csymbol encoding="text"
definitionURL="http://www.sbml.org/sbml/symbols/time"> time &lt;/csymbol&gt;
              &lt;cn&gt; 742.47 &lt;/cn&gt;
            &lt;/apply&gt;
          &lt;/math&gt;
        &lt;/trigger&gt;
        &lt;listOfEventAssignments&gt;
          <eventAssignment
variable="mwcedbe49e_2d28_4720_8fcd_207db64228cf">
            &lt;math xmlns="http://www.w3.org/1998/Math/MathML"&gt;
              &lt;cn type="integer"&gt; 1 &lt;/cn&gt;
            &lt;/math&gt;
          &lt;/eventAssignment&gt;
        &lt;/listOfEventAssignments&gt;
      &lt;/event&gt;
      <event id="mw46f091a6_4ba5_4c24_a7d8_ba6e3484a08e"
name="event_670">
        &lt;trigger&gt;
          &lt;math xmlns="http://www.w3.org/1998/Math/MathML"&gt;
            &lt;apply&gt;
              &lt;geq/&gt;
              <csymbol encoding="text"
definitionURL="http://www.sbml.org/sbml/symbols/time"> time &lt;/csymbol&gt;
              &lt;cn&gt; 743.9 &lt;/cn&gt;
            &lt;/apply&gt;
          &lt;/math&gt;
        &lt;/trigger&gt;
        &lt;listOfEventAssignments&gt;
          <eventAssignment
variable="mwcedbe49e_2d28_4720_8fcd_207db64228cf">
            &lt;math xmlns="http://www.w3.org/1998/Math/MathML"&gt;
              &lt;cn type="integer"&gt; 0 &lt;/cn&gt;
            &lt;/math&gt;
          &lt;/eventAssignment&gt;
        &lt;/listOfEventAssignments&gt;
      &lt;/event&gt;
      <event id="mw51805935_b47c_4e00_9414_db59da518f13"
name="event_671">
        &lt;trigger&gt;
          &lt;math xmlns="http://www.w3.org/1998/Math/MathML"&gt;
            &lt;apply&gt;
              &lt;geq/&gt;
              <csymbol encoding="text"
definitionURL="http://www.sbml.org/sbml/symbols/time"> time &lt;/csymbol&gt;
              &lt;cn&gt; 746.77 &lt;/cn&gt;
            &lt;/apply&gt;
          &lt;/math&gt;
        &lt;/trigger&gt;
        &lt;listOfEventAssignments&gt;
```

```
          <eventAssignment
variable="mwcedbe49e_2d28_4720_8fcd_207db64228cf">
            <math xmlns="http://www.w3.org/1998/Math/MathML">
              <cn type="integer"> 1 </cn>
            </math>
          </eventAssignment>
        </listOfEventAssignments>
      </event>
      <event id="mw936724d8_c967_48c6_87a4_e94ee58e3417"
name="event_672">
        <trigger>
          <math xmlns="http://www.w3.org/1998/Math/MathML">
            <apply>
              <geq/>
              <csymbol encoding="text"
definitionURL="http://www.sbml.org/sbml/symbols/time"> time </csymbol>
              <cn> 748.2 </cn>
            </apply>
          </math>
        </trigger>
        <listOfEventAssignments>
          <eventAssignment
variable="mwcedbe49e_2d28_4720_8fcd_207db64228cf">
            <math xmlns="http://www.w3.org/1998/Math/MathML">
              <cn type="integer"> 0 </cn>
            </math>
          </eventAssignment>
        </listOfEventAssignments>
      </event>
      <event id="mw214d3683_39d5_428e_a444_5ad2b92b4347"
name="event_673">
        <trigger>
          <math xmlns="http://www.w3.org/1998/Math/MathML">
            <apply>
              <geq/>
              <csymbol encoding="text"
definitionURL="http://www.sbml.org/sbml/symbols/time"> time </csymbol>
              <cn> 751.07 </cn>
            </apply>
          </math>
        </trigger>
        <listOfEventAssignments>
          <eventAssignment
variable="mwcedbe49e_2d28_4720_8fcd_207db64228cf">
            <math xmlns="http://www.w3.org/1998/Math/MathML">
              <cn type="integer"> 1 </cn>
            </math>
          </eventAssignment>
        </listOfEventAssignments>
      </event>
      <event id="mwfa72225a_67ee_474c_95ab_78e8957b405e"
name="event_674">
```

```
        &lt;trigger&gt;
          &lt;math xmlns="http://www.w3.org/1998/Math/MathML"&gt;
            &lt;apply&gt;
              &lt;geq/&gt;
              <csymbol encoding="text"
definitionURL="http://www.sbml.org/sbml/symbols/time"> time &lt;/csymbol&gt;
              &lt;cn&gt; 752.5 &lt;/cn&gt;
            &lt;/apply&gt;
          &lt;/math&gt;
        &lt;/trigger&gt;
        &lt;listOfEventAssignments&gt;
          <eventAssignment
variable="mwcedbe49e_2d28_4720_8fcd_207db64228cf">
            &lt;math xmlns="http://www.w3.org/1998/Math/MathML"&gt;
              &lt;cn type="integer"&gt; 0 &lt;/cn&gt;
            &lt;/math&gt;
          &lt;/eventAssignment&gt;
        &lt;/listOfEventAssignments&gt;
      &lt;/event&gt;
      <event id="mwede5b678_3f28_49c1_9b32_3e8bbbfe7336"
name="event_675">
        &lt;trigger&gt;
          &lt;math xmlns="http://www.w3.org/1998/Math/MathML"&gt;
            &lt;apply&gt;
              &lt;geq/&gt;
              <csymbol encoding="text"
definitionURL="http://www.sbml.org/sbml/symbols/time"> time &lt;/csymbol&gt;
              &lt;cn&gt; 755.37 &lt;/cn&gt;
            &lt;/apply&gt;
          &lt;/math&gt;
        &lt;/trigger&gt;
        &lt;listOfEventAssignments&gt;
          <eventAssignment
variable="mwcedbe49e_2d28_4720_8fcd_207db64228cf">
            &lt;math xmlns="http://www.w3.org/1998/Math/MathML"&gt;
              &lt;cn type="integer"&gt; 1 &lt;/cn&gt;
            &lt;/math&gt;
          &lt;/eventAssignment&gt;
        &lt;/listOfEventAssignments&gt;
      &lt;/event&gt;
      <event id="mw2020427a_3342_45e8_9e97_d99ae81eaf1f"
name="event_676">
        &lt;trigger&gt;
          &lt;math xmlns="http://www.w3.org/1998/Math/MathML"&gt;
            &lt;apply&gt;
              &lt;geq/&gt;
              <csymbol encoding="text"
definitionURL="http://www.sbml.org/sbml/symbols/time"> time &lt;/csymbol&gt;
              &lt;cn&gt; 756.8 &lt;/cn&gt;
            &lt;/apply&gt;
          &lt;/math&gt;
        &lt;/trigger&gt;
```

```
        <listOfEventAssignments>
          <eventAssignment
variable="mwcedbe49e_2d28_4720_8fcd_207db64228cf">
            <math xmlns="http://www.w3.org/1998/Math/MathML">
              <cn type="integer"> 0 </cn>
            </math>
          </eventAssignment>
        </listOfEventAssignments>
      </event>
      <event id="mwae600586_99aa_422f_9183_b6035ed1d1e2"
name="event_677">
        <trigger>
          <math xmlns="http://www.w3.org/1998/Math/MathML">
            <apply>
              <geq/>
              <csymbol encoding="text"
definitionURL="http://www.sbml.org/sbml/symbols/time"> time </csymbol>
              <cn> 759.67 </cn>
            </apply>
          </math>
        </trigger>
        <listOfEventAssignments>
          <eventAssignment
variable="mwcedbe49e_2d28_4720_8fcd_207db64228cf">
            <math xmlns="http://www.w3.org/1998/Math/MathML">
              <cn type="integer"> 1 </cn>
            </math>
          </eventAssignment>
        </listOfEventAssignments>
      </event>
      <event id="mw8a71c271_29be_4a4d_96d4_8ceb9d789ab0"
name="event_678">
        <trigger>
          <math xmlns="http://www.w3.org/1998/Math/MathML">
            <apply>
              <geq/>
              <csymbol encoding="text"
definitionURL="http://www.sbml.org/sbml/symbols/time"> time </csymbol>
              <cn> 761.1 </cn>
            </apply>
          </math>
        </trigger>
        <listOfEventAssignments>
          <eventAssignment
variable="mwcedbe49e_2d28_4720_8fcd_207db64228cf">
            <math xmlns="http://www.w3.org/1998/Math/MathML">
              <cn type="integer"> 0 </cn>
            </math>
          </eventAssignment>
        </listOfEventAssignments>
      </event>
```

```
      <event id="mwc5965871_71a3_468f_bda6_d855e3280b63"
name="event_679">
        &lt;trigger&gt;
          &lt;math xmlns="http://www.w3.org/1998/Math/MathML"&gt;
            &lt;apply&gt;
              &lt;geq/&gt;
              <csymbol encoding="text"
definitionURL="http://www.sbml.org/sbml/symbols/time"> time &lt;/csymbol&gt;
              &lt;cn&gt; 763.97 &lt;/cn&gt;
            &lt;/apply&gt;
          &lt;/math&gt;
        &lt;/trigger&gt;
        &lt;listOfEventAssignments&gt;
          <eventAssignment
variable="mwcedbe49e_2d28_4720_8fcd_207db64228cf">
            &lt;math xmlns="http://www.w3.org/1998/Math/MathML"&gt;
              &lt;cn type="integer"&gt; 1 &lt;/cn&gt;
            &lt;/math&gt;
          &lt;/eventAssignment&gt;
        &lt;/listOfEventAssignments&gt;
      &lt;/event&gt;
      <event id="mwe1728ff1_a392_4f42_b4f0_88a57fe9441b"
name="event_680">
        &lt;trigger&gt;
          &lt;math xmlns="http://www.w3.org/1998/Math/MathML"&gt;
            &lt;apply&gt;
              &lt;geq/&gt;
              <csymbol encoding="text"
definitionURL="http://www.sbml.org/sbml/symbols/time"> time &lt;/csymbol&gt;
              &lt;cn&gt; 765.4 &lt;/cn&gt;
            &lt;/apply&gt;
          &lt;/math&gt;
        &lt;/trigger&gt;
        &lt;listOfEventAssignments&gt;
          <eventAssignment
variable="mwcedbe49e_2d28_4720_8fcd_207db64228cf">
            &lt;math xmlns="http://www.w3.org/1998/Math/MathML"&gt;
              &lt;cn type="integer"&gt; 0 &lt;/cn&gt;
            &lt;/math&gt;
          &lt;/eventAssignment&gt;
        &lt;/listOfEventAssignments&gt;
      &lt;/event&gt;
      <event id="mwceb81a46_ea2c_4cc7_95d1_b2ecdb14be79"
name="event_681">
        &lt;trigger&gt;
          &lt;math xmlns="http://www.w3.org/1998/Math/MathML"&gt;
            &lt;apply&gt;
              &lt;geq/&gt;
              <csymbol encoding="text"
definitionURL="http://www.sbml.org/sbml/symbols/time"> time &lt;/csymbol&gt;
              &lt;cn&gt; 768.27 &lt;/cn&gt;
            &lt;/apply&gt;
```

```
          &lt;/math&gt;
        &lt;/trigger&gt;
        &lt;listOfEventAssignments&gt;
          <eventAssignment
variable="mwcedbe49e_2d28_4720_8fcd_207db64228cf">
            &lt;math xmlns="http://www.w3.org/1998/Math/MathML"&gt;
              &lt;cn type="integer"&gt; 1 &lt;/cn&gt;
            &lt;/math&gt;
          &lt;/eventAssignment&gt;
        &lt;/listOfEventAssignments&gt;
      &lt;/event&gt;
      <event id="mw367febb8_d01b_4b33_b421_8bee637e8205"
name="event_682">
        &lt;trigger&gt;
          &lt;math xmlns="http://www.w3.org/1998/Math/MathML"&gt;
            &lt;apply&gt;
              &lt;geq/&gt;
              <csymbol encoding="text"
definitionURL="http://www.sbml.org/sbml/symbols/time"> time &lt;/csymbol&gt;
              &lt;cn&gt; 769.7 &lt;/cn&gt;
            &lt;/apply&gt;
          &lt;/math&gt;
        &lt;/trigger&gt;
        &lt;listOfEventAssignments&gt;
          <eventAssignment
variable="mwcedbe49e_2d28_4720_8fcd_207db64228cf">
            &lt;math xmlns="http://www.w3.org/1998/Math/MathML"&gt;
              &lt;cn type="integer"&gt; 0 &lt;/cn&gt;
            &lt;/math&gt;
          &lt;/eventAssignment&gt;
        &lt;/listOfEventAssignments&gt;
      &lt;/event&gt;
      <event id="mwb06c1ee9_7e64_48b1_b96e_b0c120e5f98d"
name="event_683">
        &lt;trigger&gt;
          &lt;math xmlns="http://www.w3.org/1998/Math/MathML"&gt;
            &lt;apply&gt;
              &lt;geq/&gt;
              <csymbol encoding="text"
definitionURL="http://www.sbml.org/sbml/symbols/time"> time &lt;/csymbol&gt;
              &lt;cn&gt; 772.57 &lt;/cn&gt;
            &lt;/apply&gt;
          &lt;/math&gt;
        &lt;/trigger&gt;
        &lt;listOfEventAssignments&gt;
          <eventAssignment
variable="mwcedbe49e_2d28_4720_8fcd_207db64228cf">
            &lt;math xmlns="http://www.w3.org/1998/Math/MathML"&gt;
              &lt;cn type="integer"&gt; 1 &lt;/cn&gt;
            &lt;/math&gt;
          &lt;/eventAssignment&gt;
        &lt;/listOfEventAssignments&gt;
```

```
      &lt;/event&gt;
      <event id="mwb4e845e7_776c_461a_b016_47d757360776"
name="event_684">
        &lt;trigger&gt;
          &lt;math xmlns="http://www.w3.org/1998/Math/MathML"&gt;
            &lt;apply&gt;
              &lt;geq/&gt;
              <csymbol encoding="text"
definitionURL="http://www.sbml.org/sbml/symbols/time"> time &lt;/csymbol&gt;
              &lt;cn type="integer"&gt; 774 &lt;/cn&gt;
            &lt;/apply&gt;
          &lt;/math&gt;
        &lt;/trigger&gt;
        &lt;listOfEventAssignments&gt;
          <eventAssignment
variable="mwcedbe49e_2d28_4720_8fcd_207db64228cf">
            &lt;math xmlns="http://www.w3.org/1998/Math/MathML"&gt;
              &lt;cn type="integer"&gt; 0 &lt;/cn&gt;
            &lt;/math&gt;
          &lt;/eventAssignment&gt;
        &lt;/listOfEventAssignments&gt;
      &lt;/event&gt;
      <event id="mwf8703e92_c609_4c1d_a626_045fca8cf9d9"
name="event_651">
        &lt;trigger&gt;
          &lt;math xmlns="http://www.w3.org/1998/Math/MathML"&gt;
            &lt;apply&gt;
              &lt;geq/&gt;
              <csymbol encoding="text"
definitionURL="http://www.sbml.org/sbml/symbols/time"> time &lt;/csymbol&gt;
              &lt;cn&gt; 690.87 &lt;/cn&gt;
            &lt;/apply&gt;
          &lt;/math&gt;
        &lt;/trigger&gt;
        &lt;listOfEventAssignments&gt;
          <eventAssignment
variable="mw7d178aae_ff78_412a_830f_3be325d22773">
            &lt;math xmlns="http://www.w3.org/1998/Math/MathML"&gt;
              &lt;cn type="integer"&gt; 0 &lt;/cn&gt;
            &lt;/math&gt;
          &lt;/eventAssignment&gt;
        &lt;/listOfEventAssignments&gt;
      &lt;/event&gt;
      <event id="mw4712397a_0c76_49ad_a5cd_16f2279e9142"
name="event_652">
        &lt;trigger&gt;
          &lt;math xmlns="http://www.w3.org/1998/Math/MathML"&gt;
            &lt;apply&gt;
              &lt;geq/&gt;
              <csymbol encoding="text"
definitionURL="http://www.sbml.org/sbml/symbols/time"> time &lt;/csymbol&gt;
              &lt;cn&gt; 692.3 &lt;/cn&gt;
```

```
          &lt;/apply&gt;
        &lt;/math&gt;
      &lt;/trigger&gt;
      &lt;listOfEventAssignments&gt;
        <eventAssignment
variable="mw7d178aae_ff78_412a_830f_3be325d22773">
          &lt;math xmlns="http://www.w3.org/1998/Math/MathML"&gt;
            &lt;cn type="integer"&gt; 1 &lt;/cn&gt;
          &lt;/math&gt;
        &lt;/eventAssignment&gt;
      &lt;/listOfEventAssignments&gt;
    &lt;/event&gt;
    <event id="mw7b2f0226_9d06_46d7_b4f1_c9a5139d7b50"
name="event_653">
      &lt;trigger&gt;
        &lt;math xmlns="http://www.w3.org/1998/Math/MathML"&gt;
          &lt;apply&gt;
            &lt;geq/&gt;
            <csymbol encoding="text"
definitionURL="http://www.sbml.org/sbml/symbols/time"> time &lt;/csymbol&gt;
            &lt;cn&gt; 695.17 &lt;/cn&gt;
          &lt;/apply&gt;
        &lt;/math&gt;
      &lt;/trigger&gt;
      &lt;listOfEventAssignments&gt;
        <eventAssignment
variable="mw7d178aae_ff78_412a_830f_3be325d22773">
          &lt;math xmlns="http://www.w3.org/1998/Math/MathML"&gt;
            &lt;cn type="integer"&gt; 0 &lt;/cn&gt;
          &lt;/math&gt;
        &lt;/eventAssignment&gt;
      &lt;/listOfEventAssignments&gt;
    &lt;/event&gt;
    <event id="mwa1de6387_556d_4865_bc66_0dd259bb3641"
name="event_654">
      &lt;trigger&gt;
        &lt;math xmlns="http://www.w3.org/1998/Math/MathML"&gt;
          &lt;apply&gt;
            &lt;geq/&gt;
            <csymbol encoding="text"
definitionURL="http://www.sbml.org/sbml/symbols/time"> time &lt;/csymbol&gt;
            &lt;cn&gt; 696.6 &lt;/cn&gt;
          &lt;/apply&gt;
        &lt;/math&gt;
      &lt;/trigger&gt;
      &lt;listOfEventAssignments&gt;
        <eventAssignment
variable="mw7d178aae_ff78_412a_830f_3be325d22773">
          &lt;math xmlns="http://www.w3.org/1998/Math/MathML"&gt;
            &lt;cn type="integer"&gt; 1 &lt;/cn&gt;
          &lt;/math&gt;
        &lt;/eventAssignment&gt;
```

```
        </listOfEventAssignments>
      </event>
      <event id="mw14628cd0_39c4_4a93_ac4d_b1cdea690fdd"
name="event_685">
        <trigger>
          <math xmlns="http://www.w3.org/1998/Math/MathML">
            <apply>
              <geq/>
              <csymbol encoding="text"
definitionURL="http://www.sbml.org/sbml/symbols/time"> time </csymbol>
              <cn> 699.47 </cn>
            </apply>
          </math>
        </trigger>
        <listOfEventAssignments>
          <eventAssignment
variable="mw7d178aae_ff78_412a_830f_3be325d22773">
            <math xmlns="http://www.w3.org/1998/Math/MathML">
              <cn type="integer"> 0 </cn>
            </math>
          </eventAssignment>
        </listOfEventAssignments>
      </event>
      <event id="mw9c72a661_4d72_403f_8acf_4c701585cc79"
name="event_686">
        <trigger>
          <math xmlns="http://www.w3.org/1998/Math/MathML">
            <apply>
              <geq/>
              <csymbol encoding="text"
definitionURL="http://www.sbml.org/sbml/symbols/time"> time </csymbol>
              <cn> 700.9 </cn>
            </apply>
          </math>
        </trigger>
        <listOfEventAssignments>
          <eventAssignment
variable="mw7d178aae_ff78_412a_830f_3be325d22773">
            <math xmlns="http://www.w3.org/1998/Math/MathML">
              <cn type="integer"> 1 </cn>
            </math>
          </eventAssignment>
        </listOfEventAssignments>
      </event>
      <event id="mw6fc7f2f1_553c_443e_a6a3_c66df1ae50d2"
name="event_687">
        <trigger>
          <math xmlns="http://www.w3.org/1998/Math/MathML">
            <apply>
              <geq/>
              <csymbol encoding="text"
definitionURL="http://www.sbml.org/sbml/symbols/time"> time </csymbol>
```

```
              <cn> 703.77 </cn>
            </apply>
          </math>
        </trigger>
        <listOfEventAssignments>
          <eventAssignment
variable="mw7d178aae_ff78_412a_830f_3be325d22773">
            <math xmlns="http://www.w3.org/1998/Math/MathML">
              <cn type="integer"> 0 </cn>
            </math>
          </eventAssignment>
        </listOfEventAssignments>
      </event>
      <event id="mw68e816e7_e3a5_4a34_bb28_5440723b0f07"
name="event_688">
        <trigger>
          <math xmlns="http://www.w3.org/1998/Math/MathML">
            <apply>
              <geq/>
              <csymbol encoding="text"
definitionURL="http://www.sbml.org/sbml/symbols/time"> time </csymbol>
              <cn> 705.2 </cn>
            </apply>
          </math>
        </trigger>
        <listOfEventAssignments>
          <eventAssignment
variable="mw7d178aae_ff78_412a_830f_3be325d22773">
            <math xmlns="http://www.w3.org/1998/Math/MathML">
              <cn type="integer"> 1 </cn>
            </math>
          </eventAssignment>
        </listOfEventAssignments>
      </event>
      <event id="mw50423240_5217_42e9_9517_0cff157a2c85"
name="event_689">
        <trigger>
          <math xmlns="http://www.w3.org/1998/Math/MathML">
            <apply>
              <geq/>
              <csymbol encoding="text"
definitionURL="http://www.sbml.org/sbml/symbols/time"> time </csymbol>
              <cn> 708.07 </cn>
            </apply>
          </math>
        </trigger>
        <listOfEventAssignments>
          <eventAssignment
variable="mw7d178aae_ff78_412a_830f_3be325d22773">
            <math xmlns="http://www.w3.org/1998/Math/MathML">
              <cn type="integer"> 0 </cn>
            </math>
```

```
          &lt;/eventAssignment&gt;
        &lt;/listOfEventAssignments&gt;
      &lt;/event&gt;
      <event id="mw14b498e8_738f_465f_a91b_460ccabd4878"
name="event_690">
        &lt;trigger&gt;
          &lt;math xmlns="http://www.w3.org/1998/Math/MathML"&gt;
            &lt;apply&gt;
              &lt;geq/&gt;
              <csymbol encoding="text"
definitionURL="http://www.sbml.org/sbml/symbols/time"> time &lt;/csymbol&gt;
              &lt;cn&gt; 709.5 &lt;/cn&gt;
            &lt;/apply&gt;
          &lt;/math&gt;
        &lt;/trigger&gt;
        &lt;listOfEventAssignments&gt;
          <eventAssignment
variable="mw7d178aae_ff78_412a_830f_3be325d22773">
            &lt;math xmlns="http://www.w3.org/1998/Math/MathML"&gt;
              &lt;cn type="integer"&gt; 1 &lt;/cn&gt;
            &lt;/math&gt;
          &lt;/eventAssignment&gt;
        &lt;/listOfEventAssignments&gt;
      &lt;/event&gt;
      <event id="mw895171e7_c852_4a50_a9dc_16f6132ff45f"
name="event_691">
        &lt;trigger&gt;
          &lt;math xmlns="http://www.w3.org/1998/Math/MathML"&gt;
            &lt;apply&gt;
              &lt;geq/&gt;
              <csymbol encoding="text"
definitionURL="http://www.sbml.org/sbml/symbols/time"> time &lt;/csymbol&gt;
              &lt;cn&gt; 712.37 &lt;/cn&gt;
            &lt;/apply&gt;
          &lt;/math&gt;
        &lt;/trigger&gt;
        &lt;listOfEventAssignments&gt;
          <eventAssignment
variable="mw7d178aae_ff78_412a_830f_3be325d22773">
            &lt;math xmlns="http://www.w3.org/1998/Math/MathML"&gt;
              &lt;cn type="integer"&gt; 0 &lt;/cn&gt;
            &lt;/math&gt;
          &lt;/eventAssignment&gt;
        &lt;/listOfEventAssignments&gt;
      &lt;/event&gt;
      <event id="mw7c5d16d3_1662_4681_99a6_19a84676d693"
name="event_692">
        &lt;trigger&gt;
          &lt;math xmlns="http://www.w3.org/1998/Math/MathML"&gt;
            &lt;apply&gt;
              &lt;geq/&gt;
```

```
              <csymbol encoding="text"
definitionURL="http://www.sbml.org/sbml/symbols/time"> time &lt;/csymbol&gt;
              &lt;cn&gt; 713.8 &lt;/cn&gt;
            &lt;/apply&gt;
          &lt;/math&gt;
        &lt;/trigger&gt;
        &lt;listOfEventAssignments&gt;
          <eventAssignment
variable="mw7d178aae_ff78_412a_830f_3be325d22773">
            &lt;math xmlns="http://www.w3.org/1998/Math/MathML"&gt;
              &lt;cn type="integer"&gt; 1 &lt;/cn&gt;
            &lt;/math&gt;
          &lt;/eventAssignment&gt;
        &lt;/listOfEventAssignments&gt;
      &lt;/event&gt;
      <event id="mwd056ff6d_eb1f_4807_af1b_b7d7b6c7c551"
name="event_693">
        &lt;trigger&gt;
          &lt;math xmlns="http://www.w3.org/1998/Math/MathML"&gt;
            &lt;apply&gt;
              &lt;geq/&gt;
              <csymbol encoding="text"
definitionURL="http://www.sbml.org/sbml/symbols/time"> time &lt;/csymbol&gt;
              &lt;cn&gt; 716.67 &lt;/cn&gt;
            &lt;/apply&gt;
          &lt;/math&gt;
        &lt;/trigger&gt;
        &lt;listOfEventAssignments&gt;
          <eventAssignment
variable="mw7d178aae_ff78_412a_830f_3be325d22773">
            &lt;math xmlns="http://www.w3.org/1998/Math/MathML"&gt;
              &lt;cn type="integer"&gt; 0 &lt;/cn&gt;
            &lt;/math&gt;
          &lt;/eventAssignment&gt;
        &lt;/listOfEventAssignments&gt;
      &lt;/event&gt;
      <event id="mw38b81fcd_1837_4788_89e8_f679d892aa5b"
name="event_694">
        &lt;trigger&gt;
          &lt;math xmlns="http://www.w3.org/1998/Math/MathML"&gt;
            &lt;apply&gt;
              &lt;geq/&gt;
              <csymbol encoding="text"
definitionURL="http://www.sbml.org/sbml/symbols/time"> time &lt;/csymbol&gt;
              &lt;cn&gt; 718.1 &lt;/cn&gt;
            &lt;/apply&gt;
          &lt;/math&gt;
        &lt;/trigger&gt;
        &lt;listOfEventAssignments&gt;
          <eventAssignment
variable="mw7d178aae_ff78_412a_830f_3be325d22773">
            &lt;math xmlns="http://www.w3.org/1998/Math/MathML"&gt;
```

```
            &lt;cn type="integer"&gt; 1 &lt;/cn&gt;
          &lt;/math&gt;
        &lt;/eventAssignment&gt;
      &lt;/listOfEventAssignments&gt;
    &lt;/event&gt;
    <event id="mw33d00e5a_3942_48ac_8379_f436376a9e5f"
name="event_695">
      &lt;trigger&gt;
        &lt;math xmlns="http://www.w3.org/1998/Math/MathML"&gt;
          &lt;apply&gt;
            &lt;geq/&gt;
            <csymbol encoding="text"
definitionURL="http://www.sbml.org/sbml/symbols/time"> time &lt;/csymbol&gt;
            &lt;cn&gt; 720.97 &lt;/cn&gt;
          &lt;/apply&gt;
        &lt;/math&gt;
      &lt;/trigger&gt;
      &lt;listOfEventAssignments&gt;
        <eventAssignment
variable="mw7d178aae_ff78_412a_830f_3be325d22773">
          &lt;math xmlns="http://www.w3.org/1998/Math/MathML"&gt;
            &lt;cn type="integer"&gt; 0 &lt;/cn&gt;
          &lt;/math&gt;
        &lt;/eventAssignment&gt;
      &lt;/listOfEventAssignments&gt;
    &lt;/event&gt;
    <event id="mw7ea5a350_6feb_4722_a751_c97b2e021ba6"
name="event_696">
      &lt;trigger&gt;
        &lt;math xmlns="http://www.w3.org/1998/Math/MathML"&gt;
          &lt;apply&gt;
            &lt;geq/&gt;
            <csymbol encoding="text"
definitionURL="http://www.sbml.org/sbml/symbols/time"> time &lt;/csymbol&gt;
            &lt;cn&gt; 722.4 &lt;/cn&gt;
          &lt;/apply&gt;
        &lt;/math&gt;
      &lt;/trigger&gt;
      &lt;listOfEventAssignments&gt;
        <eventAssignment
variable="mw7d178aae_ff78_412a_830f_3be325d22773">
          &lt;math xmlns="http://www.w3.org/1998/Math/MathML"&gt;
            &lt;cn type="integer"&gt; 1 &lt;/cn&gt;
          &lt;/math&gt;
        &lt;/eventAssignment&gt;
      &lt;/listOfEventAssignments&gt;
    &lt;/event&gt;
    <event id="mwdc268e7e_a2af_4d84_923a_b7fbd0d8b44a"
name="event_697">
      &lt;trigger&gt;
        &lt;math xmlns="http://www.w3.org/1998/Math/MathML"&gt;
          &lt;apply&gt;
```

```
              &lt;geq/&gt;
              <csymbol encoding="text"
definitionURL="http://www.sbml.org/sbml/symbols/time"> time &lt;/csymbol&gt;
              &lt;cn&gt; 725.27 &lt;/cn&gt;
            &lt;/apply&gt;
          &lt;/math&gt;
        &lt;/trigger&gt;
        &lt;listOfEventAssignments&gt;
          <eventAssignment
variable="mw7d178aae_ff78_412a_830f_3be325d22773">
            &lt;math xmlns="http://www.w3.org/1998/Math/MathML"&gt;
              &lt;cn type="integer"&gt; 0 &lt;/cn&gt;
            &lt;/math&gt;
          &lt;/eventAssignment&gt;
        &lt;/listOfEventAssignments&gt;
      &lt;/event&gt;
      <event id="mwb1bbd18a_dbaa_4ede_92cb_60ed821fe5b2"
name="event_698">
        &lt;trigger&gt;
          &lt;math xmlns="http://www.w3.org/1998/Math/MathML"&gt;
            &lt;apply&gt;
              &lt;geq/&gt;
              <csymbol encoding="text"
definitionURL="http://www.sbml.org/sbml/symbols/time"> time &lt;/csymbol&gt;
              &lt;cn&gt; 726.7 &lt;/cn&gt;
            &lt;/apply&gt;
          &lt;/math&gt;
        &lt;/trigger&gt;
        &lt;listOfEventAssignments&gt;
          <eventAssignment
variable="mw7d178aae_ff78_412a_830f_3be325d22773">
            &lt;math xmlns="http://www.w3.org/1998/Math/MathML"&gt;
              &lt;cn type="integer"&gt; 1 &lt;/cn&gt;
            &lt;/math&gt;
          &lt;/eventAssignment&gt;
        &lt;/listOfEventAssignments&gt;
      &lt;/event&gt;
      <event id="mwf9d72adb_c327_44a0_9c3b_da25c1375fd3"
name="event_699">
        &lt;trigger&gt;
          &lt;math xmlns="http://www.w3.org/1998/Math/MathML"&gt;
            &lt;apply&gt;
              &lt;geq/&gt;
              <csymbol encoding="text"
definitionURL="http://www.sbml.org/sbml/symbols/time"> time &lt;/csymbol&gt;
              &lt;cn&gt; 729.57 &lt;/cn&gt;
            &lt;/apply&gt;
          &lt;/math&gt;
        &lt;/trigger&gt;
        &lt;listOfEventAssignments&gt;
          <eventAssignment
variable="mw7d178aae_ff78_412a_830f_3be325d22773">
```

```
            <math xmlns="http://www.w3.org/1998/Math/MathML">
              <cn type="integer"> 0 </cn>
            </math>
          </eventAssignment>
        </listOfEventAssignments>
      </event>
      <event id="mw356337f2_c069_42d3_99f8_3e1f42be0a97"
name="event_700">
        <trigger>
          <math xmlns="http://www.w3.org/1998/Math/MathML">
            <apply>
              <geq/>
              <csymbol encoding="text"
definitionURL="http://www.sbml.org/sbml/symbols/time"> time </csymbol>
              <cn type="integer"> 731 </cn>
            </apply>
          </math>
        </trigger>
        <listOfEventAssignments>
          <eventAssignment
variable="mw7d178aae_ff78_412a_830f_3be325d22773">
            <math xmlns="http://www.w3.org/1998/Math/MathML">
              <cn type="integer"> 1 </cn>
            </math>
          </eventAssignment>
        </listOfEventAssignments>
      </event>
      <event id="mwfd7d18a2_ee67_40be_a8fa_5850b41fc7b8"
name="event_701">
        <trigger>
          <math xmlns="http://www.w3.org/1998/Math/MathML">
            <apply>
              <geq/>
              <csymbol encoding="text"
definitionURL="http://www.sbml.org/sbml/symbols/time"> time </csymbol>
              <cn> 733.87 </cn>
            </apply>
          </math>
        </trigger>
        <listOfEventAssignments>
          <eventAssignment
variable="mw7d178aae_ff78_412a_830f_3be325d22773">
            <math xmlns="http://www.w3.org/1998/Math/MathML">
              <cn type="integer"> 0 </cn>
            </math>
          </eventAssignment>
        </listOfEventAssignments>
      </event>
      <event id="mw78bd2c1f_f978_4025_afc1_f61e52fa12f1"
name="event_702">
        <trigger>
          <math xmlns="http://www.w3.org/1998/Math/MathML">
```

```
          <apply>
            <geq/>
            <csymbol encoding="text"
definitionURL="http://www.sbml.org/sbml/symbols/time"> time </csymbol>
            <cn> 735.3 </cn>
          </apply>
        </math>
      </trigger>
      <listOfEventAssignments>
        <eventAssignment
variable="mw7d178aae_ff78_412a_830f_3be325d22773">
          <math xmlns="http://www.w3.org/1998/Math/MathML">
            <cn type="integer"> 1 </cn>
          </math>
        </eventAssignment>
      </listOfEventAssignments>
    </event>
    <event id="mwcfad3bdf_0015_4a23_942b_a9a105199974"
name="event_703">
      <trigger>
        <math xmlns="http://www.w3.org/1998/Math/MathML">
          <apply>
            <geq/>
            <csymbol encoding="text"
definitionURL="http://www.sbml.org/sbml/symbols/time"> time </csymbol>
            <cn> 738.17 </cn>
          </apply>
        </math>
      </trigger>
      <listOfEventAssignments>
        <eventAssignment
variable="mw7d178aae_ff78_412a_830f_3be325d22773">
          <math xmlns="http://www.w3.org/1998/Math/MathML">
            <cn type="integer"> 0 </cn>
          </math>
        </eventAssignment>
      </listOfEventAssignments>
    </event>
    <event id="mwfe8e4f78_29a5_441c_94a0_e004fb03847c"
name="event_704">
      <trigger>
        <math xmlns="http://www.w3.org/1998/Math/MathML">
          <apply>
            <geq/>
            <csymbol encoding="text"
definitionURL="http://www.sbml.org/sbml/symbols/time"> time </csymbol>
            <cn> 739.6 </cn>
          </apply>
        </math>
      </trigger>
      <listOfEventAssignments>
```

```
          <eventAssignment
variable="mw7d178aae_ff78_412a_830f_3be325d22773">
            <math xmlns="http://www.w3.org/1998/Math/MathML">
              <cn type="integer"> 1 </cn>
            </math>
          </eventAssignment>
        </listOfEventAssignments>
      </event>
      <event id="mw6853725f_34da_464d_80e4_6af2bfe5d7e1"
name="event_705">
        <trigger>
          <math xmlns="http://www.w3.org/1998/Math/MathML">
            <apply>
              <geq/>
              <csymbol encoding="text"
definitionURL="http://www.sbml.org/sbml/symbols/time"> time </csymbol>
              <cn> 742.47 </cn>
            </apply>
          </math>
        </trigger>
        <listOfEventAssignments>
          <eventAssignment
variable="mw7d178aae_ff78_412a_830f_3be325d22773">
            <math xmlns="http://www.w3.org/1998/Math/MathML">
              <cn type="integer"> 0 </cn>
            </math>
          </eventAssignment>
        </listOfEventAssignments>
      </event>
      <event id="mwf6ab0567_0a6b_467c_b58e_0e331e6c6a70"
name="event_706">
        <trigger>
          <math xmlns="http://www.w3.org/1998/Math/MathML">
            <apply>
              <geq/>
              <csymbol encoding="text"
definitionURL="http://www.sbml.org/sbml/symbols/time"> time </csymbol>
              <cn> 743.9 </cn>
            </apply>
          </math>
        </trigger>
        <listOfEventAssignments>
          <eventAssignment
variable="mw7d178aae_ff78_412a_830f_3be325d22773">
            <math xmlns="http://www.w3.org/1998/Math/MathML">
              <cn type="integer"> 1 </cn>
            </math>
          </eventAssignment>
        </listOfEventAssignments>
      </event>
      <event id="mw409f74a2_d543_4112_a658_8299303c408b"
name="event_707">
```

```
        <trigger>
          <math xmlns="http://www.w3.org/1998/Math/MathML">
            <apply>
              <geq/>
              <csymbol encoding="text"
definitionURL="http://www.sbml.org/sbml/symbols/time"> time </csymbol>
              <cn> 746.77 </cn>
            </apply>
          </math>
        </trigger>
        <listOfEventAssignments>
          <eventAssignment
variable="mw7d178aae_ff78_412a_830f_3be325d22773">
            <math xmlns="http://www.w3.org/1998/Math/MathML">
              <cn type="integer"> 0 </cn>
            </math>
          </eventAssignment>
        </listOfEventAssignments>
      </event>
      <event id="mwba24101e_6523_4de2_9d33_4b94029fc1f0"
name="event_708">
        <trigger>
          <math xmlns="http://www.w3.org/1998/Math/MathML">
            <apply>
              <geq/>
              <csymbol encoding="text"
definitionURL="http://www.sbml.org/sbml/symbols/time"> time </csymbol>
              <cn> 748.2 </cn>
            </apply>
          </math>
        </trigger>
        <listOfEventAssignments>
          <eventAssignment
variable="mw7d178aae_ff78_412a_830f_3be325d22773">
            <math xmlns="http://www.w3.org/1998/Math/MathML">
              <cn type="integer"> 1 </cn>
            </math>
          </eventAssignment>
        </listOfEventAssignments>
      </event>
      <event id="mw5a98c5d3_7b2b_4d70_8d9a_a68a2a8563e6"
name="event_709">
        <trigger>
          <math xmlns="http://www.w3.org/1998/Math/MathML">
            <apply>
              <geq/>
              <csymbol encoding="text"
definitionURL="http://www.sbml.org/sbml/symbols/time"> time </csymbol>
              <cn> 751.07 </cn>
            </apply>
          </math>
        </trigger>
```

```
        <listOfEventAssignments>
          <eventAssignment
variable="mw7d178aae_ff78_412a_830f_3be325d22773">
            <math xmlns="http://www.w3.org/1998/Math/MathML">
              <cn type="integer"> 0 </cn>
            </math>
          </eventAssignment>
        </listOfEventAssignments>
      </event>
      <event id="mwfabf33a8_6732_4be5_8045_8de99921943d"
name="event_710">
        <trigger>
          <math xmlns="http://www.w3.org/1998/Math/MathML">
            <apply>
              <geq/>
              <csymbol encoding="text"
definitionURL="http://www.sbml.org/sbml/symbols/time"> time </csymbol>
              <cn> 752.5 </cn>
            </apply>
          </math>
        </trigger>
        <listOfEventAssignments>
          <eventAssignment
variable="mw7d178aae_ff78_412a_830f_3be325d22773">
            <math xmlns="http://www.w3.org/1998/Math/MathML">
              <cn type="integer"> 1 </cn>
            </math>
          </eventAssignment>
        </listOfEventAssignments>
      </event>
      <event id="mw13324e2b_ab10_4b4f_9ccd_860d7418402d"
name="event_711">
        <trigger>
          <math xmlns="http://www.w3.org/1998/Math/MathML">
            <apply>
              <geq/>
              <csymbol encoding="text"
definitionURL="http://www.sbml.org/sbml/symbols/time"> time </csymbol>
              <cn> 755.37 </cn>
            </apply>
          </math>
        </trigger>
        <listOfEventAssignments>
          <eventAssignment
variable="mw7d178aae_ff78_412a_830f_3be325d22773">
            <math xmlns="http://www.w3.org/1998/Math/MathML">
              <cn type="integer"> 0 </cn>
            </math>
          </eventAssignment>
        </listOfEventAssignments>
      </event>
```

```
      <event id="mw80d17127_867f_4472_8c3a_418c3395e73d"
name="event_712">
        <trigger>
          <math xmlns="http://www.w3.org/1998/Math/MathML">
            <apply>
              <geq/>
              <csymbol encoding="text"
definitionURL="http://www.sbml.org/sbml/symbols/time"> time </csymbol>
              <cn> 756.8 </cn>
            </apply>
          </math>
        </trigger>
        <listOfEventAssignments>
          <eventAssignment
variable="mw7d178aae_ff78_412a_830f_3be325d22773">
            <math xmlns="http://www.w3.org/1998/Math/MathML">
              <cn type="integer"> 1 </cn>
            </math>
          </eventAssignment>
        </listOfEventAssignments>
      </event>
      <event id="mw788c4ed8_c736_43ec_b5ac_506e542e32f2"
name="event_713">
        <trigger>
          <math xmlns="http://www.w3.org/1998/Math/MathML">
            <apply>
              <geq/>
              <csymbol encoding="text"
definitionURL="http://www.sbml.org/sbml/symbols/time"> time </csymbol>
              <cn> 759.67 </cn>
            </apply>
          </math>
        </trigger>
        <listOfEventAssignments>
          <eventAssignment
variable="mw7d178aae_ff78_412a_830f_3be325d22773">
            <math xmlns="http://www.w3.org/1998/Math/MathML">
              <cn type="integer"> 0 </cn>
            </math>
          </eventAssignment>
        </listOfEventAssignments>
      </event>
      <event id="mw91983427_a9dd_4e91_b527_31271d999b39"
name="event_714">
        <trigger>
          <math xmlns="http://www.w3.org/1998/Math/MathML">
            <apply>
              <geq/>
              <csymbol encoding="text"
definitionURL="http://www.sbml.org/sbml/symbols/time"> time </csymbol>
              <cn> 761.1 </cn>
            </apply>
```

```
          </math>
        </trigger>
        <listOfEventAssignments>
          <eventAssignment
variable="mw7d178aae_ff78_412a_830f_3be325d22773">
            <math xmlns="http://www.w3.org/1998/Math/MathML">
              <cn type="integer"> 1 </cn>
            </math>
          </eventAssignment>
        </listOfEventAssignments>
      </event>
      <event id="mwd959cc26_55f3_47e8_9c27_f2d176180ccc"
name="event_715">
        <trigger>
          <math xmlns="http://www.w3.org/1998/Math/MathML">
            <apply>
              <geq/>
              <csymbol encoding="text"
definitionURL="http://www.sbml.org/sbml/symbols/time"> time </csymbol>
              <cn> 763.97 </cn>
            </apply>
          </math>
        </trigger>
        <listOfEventAssignments>
          <eventAssignment
variable="mw7d178aae_ff78_412a_830f_3be325d22773">
            <math xmlns="http://www.w3.org/1998/Math/MathML">
              <cn type="integer"> 0 </cn>
            </math>
          </eventAssignment>
        </listOfEventAssignments>
      </event>
      <event id="mw64c4dad3_d495_41e8_9da4_4d576c29948e"
name="event_716">
        <trigger>
          <math xmlns="http://www.w3.org/1998/Math/MathML">
            <apply>
              <geq/>
              <csymbol encoding="text"
definitionURL="http://www.sbml.org/sbml/symbols/time"> time </csymbol>
              <cn> 765.4 </cn>
            </apply>
          </math>
        </trigger>
        <listOfEventAssignments>
          <eventAssignment
variable="mw7d178aae_ff78_412a_830f_3be325d22773">
            <math xmlns="http://www.w3.org/1998/Math/MathML">
              <cn type="integer"> 1 </cn>
            </math>
          </eventAssignment>
        </listOfEventAssignments>
```

```
      </event>
      <event id="mw379a62e4_7aeb_4792_8ece_533411787e33"
name="event_717">
        <trigger>
          <math xmlns="http://www.w3.org/1998/Math/MathML">
            <apply>
              <geq/>
              <csymbol encoding="text"
definitionURL="http://www.sbml.org/sbml/symbols/time"> time </csymbol>
              <cn> 768.27 </cn>
            </apply>
          </math>
        </trigger>
        <listOfEventAssignments>
          <eventAssignment
variable="mw7d178aae_ff78_412a_830f_3be325d22773">
            <math xmlns="http://www.w3.org/1998/Math/MathML">
              <cn type="integer"> 0 </cn>
            </math>
          </eventAssignment>
        </listOfEventAssignments>
      </event>
      <event id="mw128874c0_45be_4b3a_b440_c23b59e8a5bb"
name="event_718">
        <trigger>
          <math xmlns="http://www.w3.org/1998/Math/MathML">
            <apply>
              <geq/>
              <csymbol encoding="text"
definitionURL="http://www.sbml.org/sbml/symbols/time"> time </csymbol>
              <cn> 769.7 </cn>
            </apply>
          </math>
        </trigger>
        <listOfEventAssignments>
          <eventAssignment
variable="mw7d178aae_ff78_412a_830f_3be325d22773">
            <math xmlns="http://www.w3.org/1998/Math/MathML">
              <cn type="integer"> 1 </cn>
            </math>
          </eventAssignment>
        </listOfEventAssignments>
      </event>
      <event id="mw477be1cc_ec98_4fba_8e98_6489306a64ce"
name="event_719">
        <trigger>
          <math xmlns="http://www.w3.org/1998/Math/MathML">
            <apply>
              <geq/>
              <csymbol encoding="text"
definitionURL="http://www.sbml.org/sbml/symbols/time"> time </csymbol>
              <cn> 772.57 </cn>
```

```
            </apply>
          </math>
        </trigger>
        <listOfEventAssignments>
          <eventAssignment
variable="mw7d178aae_ff78_412a_830f_3be325d22773">
            <math xmlns="http://www.w3.org/1998/Math/MathML">
              <cn type="integer"> 0 </cn>
            </math>
          </eventAssignment>
        </listOfEventAssignments>
      </event>
      <event id="mwe52ee8a8_dbbf_4d74_a8f1_09e5dc47cacc"
name="event_720">
        <trigger>
          <math xmlns="http://www.w3.org/1998/Math/MathML">
            <apply>
              <geq/>
              <csymbol encoding="text"
definitionURL="http://www.sbml.org/sbml/symbols/time"> time </csymbol>
              <cn type="integer"> 774 </cn>
            </apply>
          </math>
        </trigger>
        <listOfEventAssignments>
          <eventAssignment
variable="mw7d178aae_ff78_412a_830f_3be325d22773">
            <math xmlns="http://www.w3.org/1998/Math/MathML">
              <cn type="integer"> 1 </cn>
            </math>
          </eventAssignment>
        </listOfEventAssignments>
      </event>
    </listOfEvents>
  </model>
</sbml>
```
